# Size and Geometry Impact the Chiroptical Properties of Double Nanohoops

**DOI:** 10.1021/jacs.5c12590

**Published:** 2025-11-03

**Authors:** Philipp Seitz, Luisa Rzesny, Darleen Busse, Xiaoshuang Xiang, Mathias Hermann, Lilian Estaque, Grégory Pieters, Birgit Esser

**Affiliations:** † Institute of Organic Chemistry II and Advanced Materials, 9189 Ulm University, Albert-Einstein-Allee 11, 89081 Ulm, Germany; ‡ Université Paris-Saclay, CEA, Département Médicaments et Technologies pour la Santé (DMTS), SCBM, F-91191 Gif-sur-Yvette, France

## Abstract

Conjugated nanohoops have attracted much attention in recent years due to their unique optoelectronic properties. A more complex geometry, in which two nanohoops are covalently linked to form a so-called double nanohoop, can have a significant influence on the morphology, (chir)­optical properties and supramolecular interactions compared to a single nanohoop. Herein, we present the systematic design, synthesis, structural and chiroptical analysis of a novel series of three chiral double nanohoops together with their single hoop reference compounds. They each incorporate a tetrahydroindeno­[2,1-*a*]­indene-5,10-diol unit as an asymmetric bridge with central chirality. Notably, they display high photoluminescence quantum yields of 79–95%, along with distinct trends in absorption, emission, and energy transfer dynamics. Enantiomers of the double and reference single nanohoops were successfully separated by HPLC using a chiral stationary phase, and their chiroptical properties were investigated through electronic circular dichroism (ECD) and circularly polarized luminescence (CPL) spectroscopy revealing increased asymmetry factors (*g*_abs_) compared to their reference compounds. Our study provides a systematic exploration of how size, geometry, and asymmetry impact the optoelectronic behavior of chiral double nanohoops and offers valuable insight for the development of high-performance chiroptical materials.

## Graphical abstract

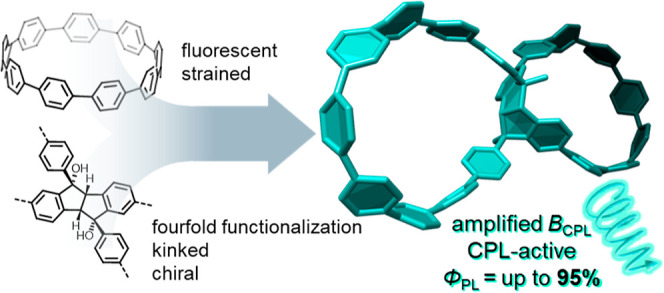

## Introduction

Curved aromatic hydrocarbons have garnered significant attention since the first [*n*]­cycloparaphenylene (CPP) synthesis.^1^ Their bent π-system leads to unique optoelectronic properties,^2–4^ influences aromaticity,^5,6^ enables rich supramolecular chemistry,^7–9^ and makes such molecules of interest for application in organic electronics.^10–14^ Last but not least, curved aromatic hydrocarbons have aesthetic appeal, and they present a significant challenge to the synthetic chemist.^15^ The ability to modulate the optoelectronic properties of conjugated nanohoops by varying their hoop size^2,16^ or the nature of the π-systems they are composed of^17,18^ has opened new avenues for research. Incorporating symmetry-breaking units, such as *meta*-phenylene moieties, can lead to significant alterations in their optoelectronic properties, including increased photoluminescence quantum yield and a change in emission behavior, as well as introducing chirality.^3,4,19,20^

In recent years, molecules containing two or more covalently linked nanohoops (so-called double nanohoops) have attracted significant attention to further investigate the structure–property relationships of these curved π-systems.^21^ These structures range from phenylene-only double nanohoops^22–24^ to more complex architectures with diverse linking units,^25–30^ leading to unexpected properties such as anti-Kasha emission behavior^31^ and enhanced analyte sensing^32^ through morphology manipulation. Depending on the geometry of the linking unit, such double nanohoops can be chiral. In most reported (double) nanohoops, chirality arises from bending the linking unit, typically giving rise to axial chirality in the form of atropisomers.

The exploration of chiral nanohoops is vital, as their asymmetry greatly influences their optical properties, such as electronic circular dichroism (ECD) and circularly polarized luminescence (CPL), enabling their use in chiroptical devices.^7,33–38^ However, a limitation of many previously synthesized chiral nanohoops is their low CPL brightness (*B*_CPL_ = ε × Φ_PL_ × *g*_lum_/2),^39^ where one or more factors are often insufficient to achieve optimal performance.^40^

Herein we report on the systematic design, synthesis and analysis of a novel series of chiral (double) nanohoops and their trends in structural and chiroptical properties (Figure 1). They consist of two CPPs or—more correctly—oligo­(paraphenylene) loops connected through a central tetrahydroindeno­[2,1-*a*]­indene-5,10-diol unit as asymmetric bridge. With four stereocenters this bridge introduces the rare form of central chirality into the double nanohoops. This distinguishes our molecules from other single and double nanohoop chiroptical molecules, in which chirality is often introduced by axial chirality. By contrast, our system derives chirality from four stereogenic centers, formed exclusively in all-*S* or all-*R* configuration, due to the kinked nature of the precursor diketo-nanohoop geometry and the inherently convex nature of the Grignard attack (see Figure 1 and Scheme 1). We varied the hoop sizes on both side of this unit (Figure 1) to investigate optoelectronic trends, and synthesized the respective single nanohoops as reference compounds. The two loops of the double nanohoops are differentiated by their connection with the central unit: one through the five-membered ring (purple, n amount of phenylene units) and the other through the six-membered ring (orange, m amount of conjugated phenylene units in the final nanohoop) of the central tetrahydroindenoindene connecting unit. Note that we chose to color the six-membered rings that are part of the central linking unit in orange as well, as they are part of the conjugated system in the final nanohoops.

**Figure 1:**
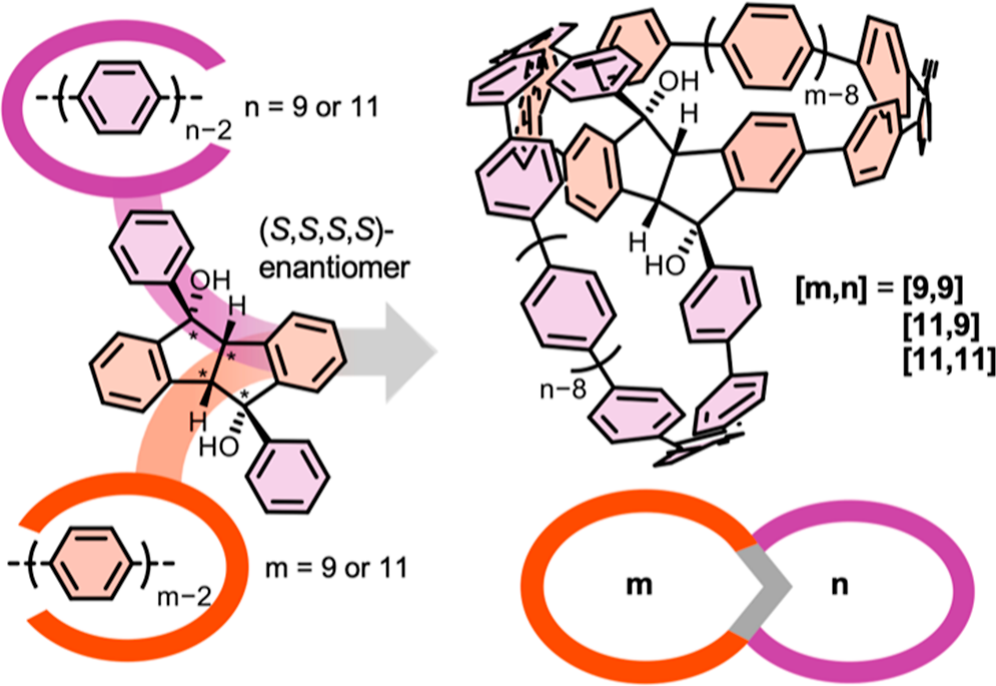
Chiral double nanohoops [*m*,*n*] (**[9,9]**, **[11,9]** and **[11,11]**, right) reported herein with a chiral tetrahydroindeno­[2,1-*a*]­indene-5,10-diol unit (left) as asymmetric bridge with four stereocenters. *m* and *n* indicate the number of conjugated phenylene units in each hoop. Only one enantiomer is shown for simplicity.

**Scheme 1:**
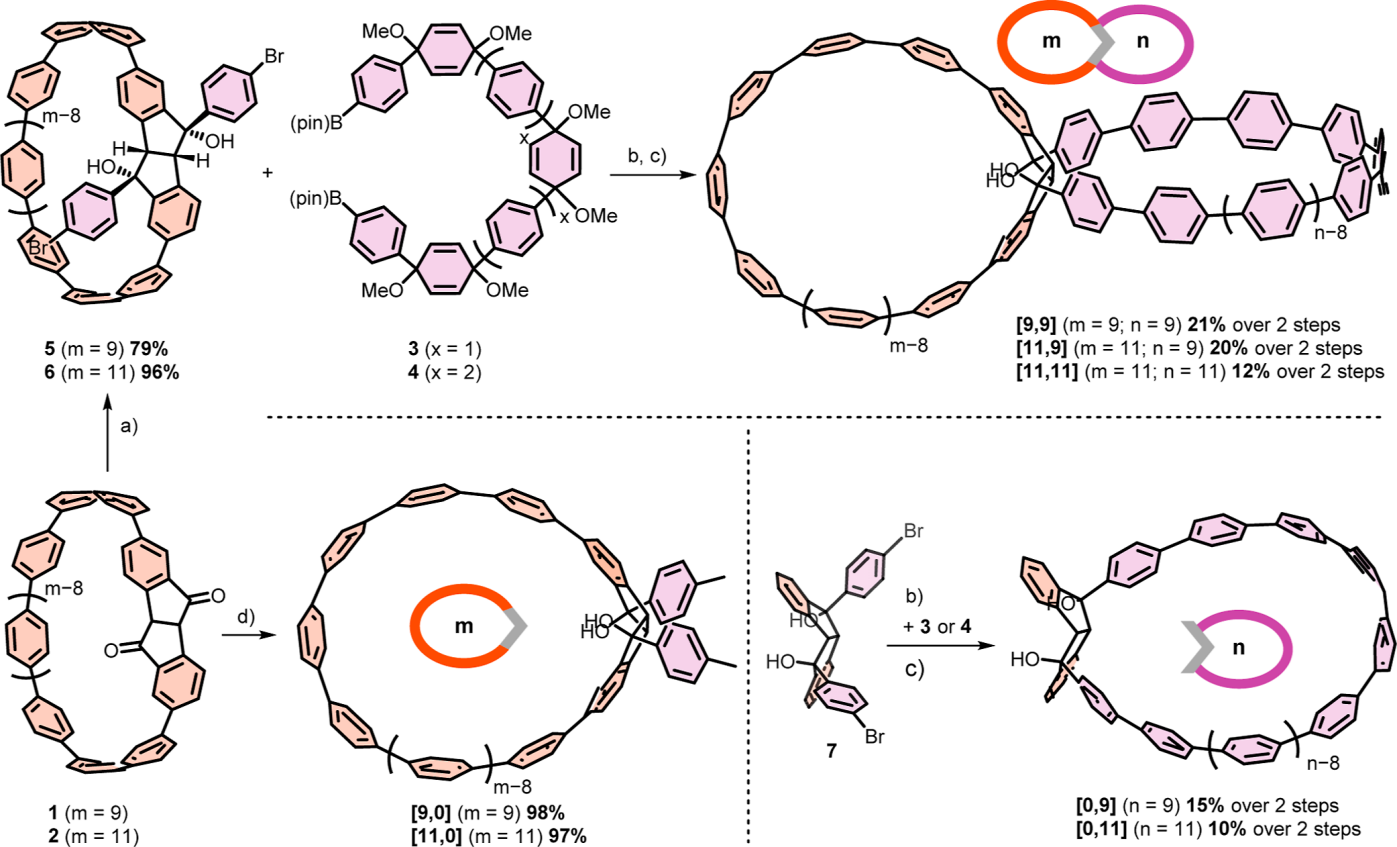
Synthesis of Double Nanohoops **[9,9]**, **[11,9]**, and **[11,11]** and of Reference Single Nanohoops **[9,0]**, **[11,0]**, **[0,9]**, and **[0,11]** ^a^

a For clarity, only one enantiomer of each hoop is shown. Reagents and conditions: (a) 1,4-Dibromobenzene, Mg, LiCl, CeCl_3_, anh. THF, −20 °C to rt, 18 h; (b) Pd­(OAc)_2_, K_3_PO_4_, SPhos, 1,4-dioxane, H_2_O, 80 °C, 1 day; (c) SnCl_2_ × 2H_2_O, HCl, THF, rt, 1 day; (d) *p*-TolMgBr·LiCl, CeCl_3_, anh. THF, −20 °C to rt, 18 h.

Following this design approach, we synthesized three double nanohoops with different amounts of phenylene units in each hoop, resulting in the general acronyms [*m*,*n*] (**[9,9]**; **[11,9]**; **[11,11]**, Figure 1). In addition to these double nanohoops, we also synthesized and investigated four single nanohoops as reference compounds, representing one side of each double nanohoop, namely **[0,9]**, **[0,11]**, **[9,0]**, and **[11,0]** (Scheme 1).

Incorporating a novel linking unit that introduces chirality and asymmetry presents both a synthetic challenge and an opportunity to explore new properties. The resulting double nanohoops **[9,9]**, **[11,9]** and **[11,11]** as well as their reference compounds **[0,9]**, **[0,11]**, **[9,0]**, and **[11,0]** are interesting both synthetically and aesthetically. More importantly, they serve as a platform to study hoop strain and their unique optoelectronic properties. This is possible because we systematically varied the loop size, allowing us to explore these effects in detail. Understanding the energy transfer dynamics within these strained systems reveals fundamental insights into their optoelectronic behavior.

## Results and Discussion

Our synthetic strategy to the double nanohoops starts from diketo[7]­CPP **1** and diketo[9]­CPP **2**, whose syntheses we previously reported.^14,19,41^ Both were first reacted with a Turbo-Grignard reagent made from 1,4-dibromobenzene and one equivalent of Mg in the presence of LiCl under CeCl_3_-mediation to furnish **5** and **6** in 79% and 96% yield, respectively.^42–44^ In these dibromides, the kinked structure of the central tetrahydroindenoindene diol unit (Figure 1) facilitates hoop formation through its five-membered rings in the following. Reaction with two different sizes of the C-shaped oligo­(paraphenylene) precursors **3** or **4** followed by aromatization furnished the double hoops [*m*,*n*]. C-shaped units **3** and **4** were synthesized following literature protocols,^14,45^ where we developed an improved synthesis for **3** furnishing higher yields than previously reported (see Supporting Information for details). Ring closure was achieved using Suzuki–Miyaura cross-coupling conditions, followed by H_2_SnCl_4_-mediated aromatization,^46^ which led to double nanohoops **[9,9]**, **[11,9]**, and **[11,11]**. Due to the extreme sensitivity of the intermediates toward slightly acidic conditions, no purification was performed after the ring-closing step. Instead, the crude products were directly used in the reductive aromatization, yielding 12–21% over two steps for the three double nanohoops. Inspite of repeated attempts, the **[9,11]** double nanohoop was synthetically not accessible, as it almost completely decomposed during workup, and was only visible with major impurities in ^1^H NMR and high-resolution mass spectra. Water elimination using Burgess reagent or under acidic conditions did not successfully yield the respective dibenzopentalene-based double nanohoops, as they could only be detected in trace amounts.^19,41^

Reference single nanohoops **[9,0]** and **[11,0]** were obtained from the respective diketoCPP **1** or **2** via a CeCl_3_-mediated Grignard addition using *p*-tolylmagnesium bromide. To synthesize the reference single nanohoops **[0,9]** and **[0,11]**, we reacted **7** with the C-shaped units **3** or **4** under Suzuki–MiyauraA cross-coupling conditions, followed by reductive aromatization, as described above.

The synthesized double nanohoop structures represent a novel class of CPP derivatives, incorporating a symmetry-breaking linking unit that not only enhances the photoluminescence quantum yield but also introduces chirality, making them potentially interesting as CPL materials (see below).

### Structural Properties

The structures and purity of the double nanohoops **[9,9]**, **[11,9]**, and **[11,11]** as well as of reference single nanohoops **[9,0]**, **[11,0]**, **[0,9]** and **[0,11]** were unambiguously confirmed using one- and two-dimensional NMR spectroscopy, mass spectrometry and single-crystal X-ray diffraction (XRD) for **[11,9]**. Owing to the structural similarity among all the novel double nanohoops, they exhibit comparable NMR spectra (Figure 2). In the ^1^H NMR spectra of double nanohoops **[9,9]**, **[11,9]**, and **[11,11]**, nearly all signals exhibit a downfield shift of the proton resonances with increasing hoop size. This effect is particularly notable for the bridge protons of the tetrahydroindenoindene unit (marked blue in Figure 2), which shift from 3.83 to 3.97 ppm with increasing the hoop size from **[9,9]** to **[11,9]** to **[11,11]**. Enlarging the “purple-colored hoop” (*n*) has a slightly stronger effect than increasing the size of the “orange-colored hoop” (*m*), as these protons point inside the former. The same trend is observed for the purple-marked aromatic protons. The chemical shift of the hydroxy protons (green), on the other hand, is only affected by the size of the orange-colored hoop with a downfield shift by 0.10 ppm from **[9,9]** to **[11,9]**, and not by the size of the purple-colored hoop. A similar observation can be made for the aromatic protons highlighted in orange and red.

**Figure 2:**
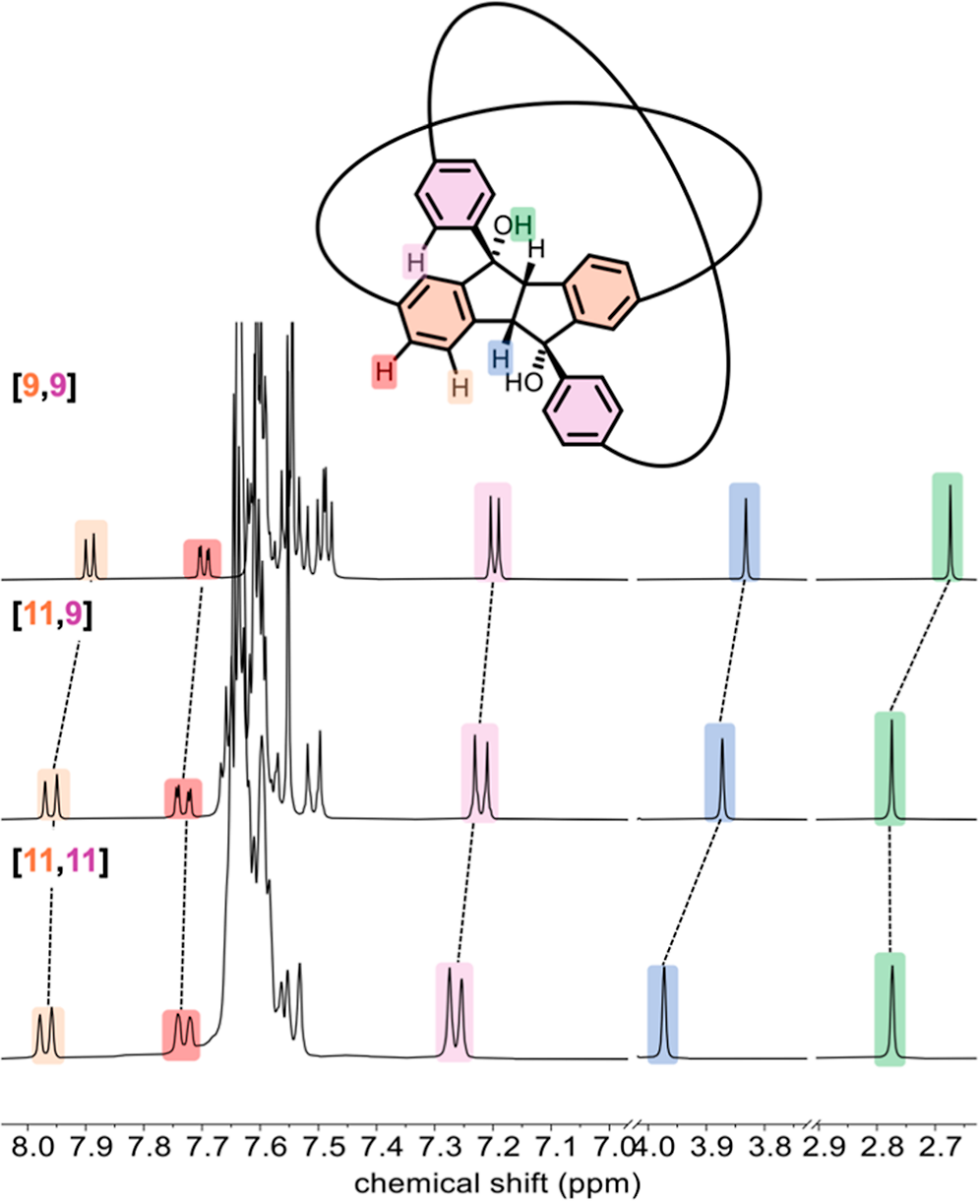
Selected regions of the ^1^H NMR spectra (600 MHz, CD_2_Cl_2_) of double nanohoops **[9,9]**, **[11,9]** and **[11,11]**.

When comparing the ^1^H NMR spectra of the reference single nanohoops **[9,0]** with **[11,0]**, and **[0,9]** with **[0,11]**, the same trend is observed: as the hoop size increases, the NMR signals are shifted downfield (see Supporting Information; Figures S55–S60). This trend is in line with what has been reported for larger [*n*]­CPPs (*n* = 8–12).^2^

A single crystal suitable for XRD could exclusively be obtained for the **[11,9]** double nanohoop (Figure 3A,B). Structure solution and refinement proved to be difficult, even with a data set recorded with synchrotron radiation. The large solvent accessible voids of the hoops’ cavities lead to highly disordered solvent molecules, which hindered the quality of the obtained data set and its refinement. Despite this drawback, a stable refinement clearly showed the core structure of the hoop. Crystals of the other double nanohoops and reference single nanohoops turned opaque upon exposure to air, likely due to solvent loss resulting from their high porosity, which induced defects in the crystal lattice and made their structures unresolvable.

**Figure 3:**
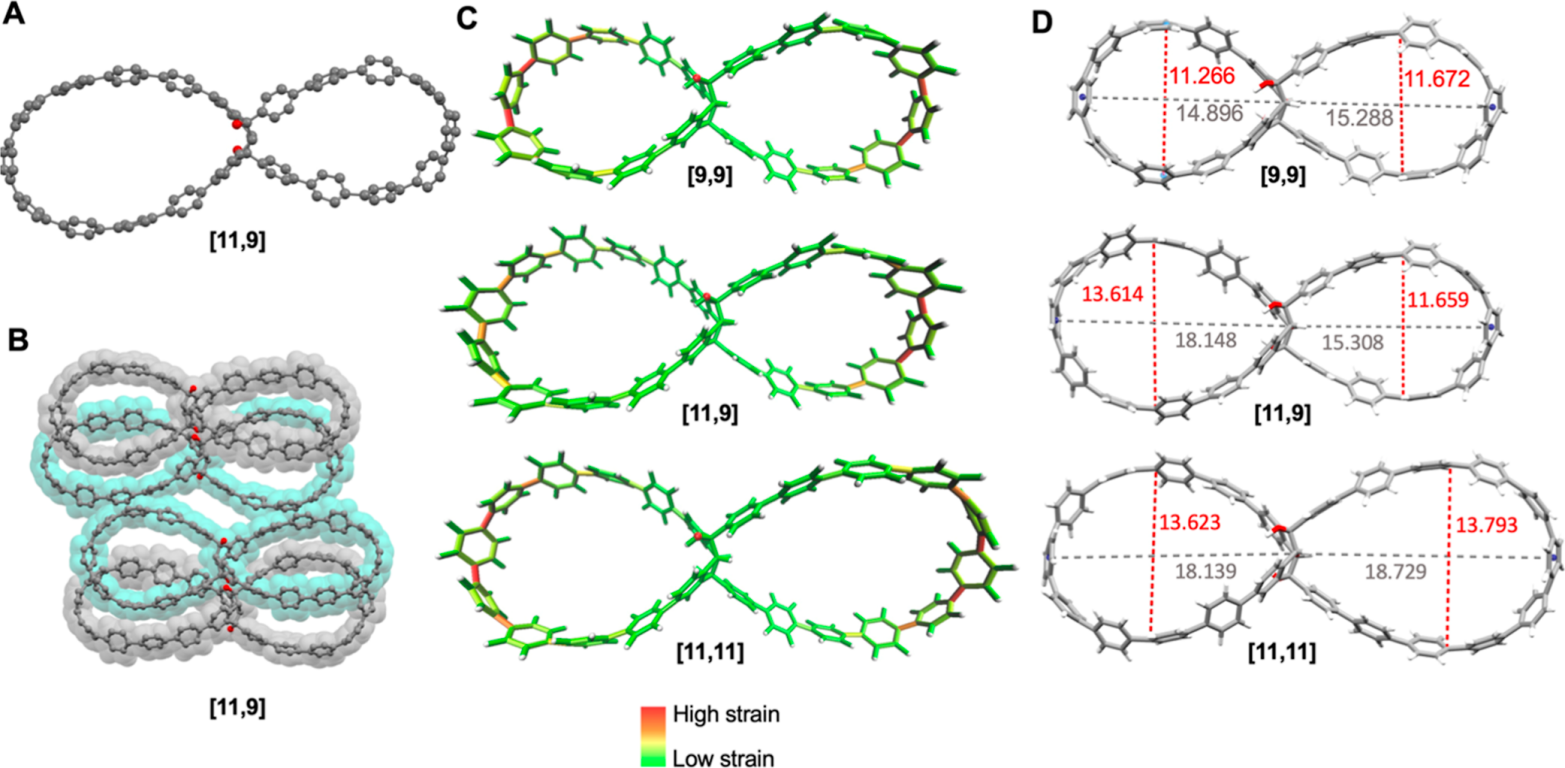
(A) Molecular structure of double nanohoop **[11,9]** in the solid state with (B) molecular packing. All hydrogen atoms and the solvent molecules are omitted for clarity; (C) StrainViz analysis (B3LYP/6-31G­(d)) of double nanohoops **[9,9]**, **[11,9]**, and **[11,11]**. (D) Geometric dimensions of double nanohoops **[9,9]**, **[11,9]**, and **[11,11]**, optimized on the PBEh-3c level of theory, given in Å.

The geometry of the tetrahydroindenoindene unit in the **[11,9]** double nanohoop in the solid state causes both hoop sides to adopt a conformation where they are neither coplanar nor perpendicular to each other. Defining a plane for each hoop segment reveals a mutual twist of approximately 40°. However, the crystal quality is insufficient to reliably analyze bond lengths or determine the precise dihedral angle between adjacent phenyl rings. Nonetheless, the experimentally obtained structure closely aligns with the calculated structure (see Supporting Information, Figures S73 and S80).

We optimized the geometries of all three double nanohoops as well as those of the four reference single nanohoops using density functional theory at the PBEh-3c level of theory,^47–49^ following a conformer search using the global geometry optimization and ensemble generator (GOAT) implemented in ORCA.^50–53^ These calculations show that each hoop takes on a more ellipsoidal rather than round shape, which is caused by the unique geometry of the central kinked tetrahydroindenoindene unit. The double nanohoops have significant strain energies of 82 kcal mol^–1^ for **[9,9]**, 76 kcal mol^–1^ for **[11,9]** and 70 kcal mol^–1^ for **[11,11]** (calculated by using StrainViz^54^ at the B3LYP/6-31G­(d) level of theory).^55–61^ In comparison to the reference single nanohoops with strain energies of 32–45 kcal mol^–1^, these strain energies roughly amount to the sum of the two-halves. Most of the strain is located in the outer part of the paraphenylene loops, which experience the strongest bend, and in particular in the CC-bonds connecting the phenylene units (Figure 3C). This so-called dihedral strain accounts to 85% of the strain energy of **[9,9]**, for example. The central tetrahydroindenoindene experiences only slight deformations relative to its relaxed geometry (see Supporting Information, Figure S114), which accounts for ca. 0.9–3.5 kcal mol^–1^ of the strain energy. The sizes of the cavities in the double nanohoops can be estimated by their maximum height and length (see Figure 3D). The sizes range from 1.13 nm height and 1.49 nm length for **[9,9]** to 1.38 nm height and 1.87 nm length for **[11,11]** (structures optimized at the PBEh-3c level of theory).

### Optoelectronic Properties

The optical properties of the double nanohoops, as evidenced by the UV/vis absorption and emission spectra, reflect the presence of the oligo­(paraphenylene) loops and show therefore similarities to those of [*n*]­CPPs (Table 1 and Figure 4A). All double nanohoops **[9,9]**, **[11,9]**, and **[11,11]** exhibit a similar absorption maximum (between 330 and 335 nm), which can be attributed to the oligo­(paraphenylene) moieties. The energy of this transition is largely unaffected by variations in size, due to limited conjugation lengths imposed by the torsional angles between adjacent phenyl groups, and is similar to the absorption maxima in [*n*]­CPPs of 335–340 nm.^2^ The reference single nanohoops **[11,0]**, **[9,0]**, **[0,11]** and **[0,9]** exhibit the same trends in their absorption behavior due to their similarity to the double nanohoops (Table 1 and Figure 4B). Figure 4C shows a comparison between the double nanohoop **[11,9]** as well as its reference compounds **[0,9]** and **[11,0]**. The calculated absorption spectra match well in shape with the experimental ones (calculated using TDDFT at the PBE0/def2-TZVP level of theory, Figures S103–S105). An evaluation of the orbitals involved in the most important transition shows that for all four reference compounds, the HOMO → LUMO transition is the one most involved in the S_0_ → S_1_ transition, while for the three double nanohoops, the HOMO → LUMO transition is not only higher in energy, but also the oscillator strength calculated for the HOMO → LUMO transition is smaller by 1 order of magnitude.

**Table 1:** Optoelectronic and Chiroptical Data of Double Nanohoops and Reference Single Nanohoops^a^

compound	λ_max,abs_/nm	λ_max,em_/nm	ε/10^4^ cm^–1^ M^–1^	*E*_opt_ ^b^/eV	Stokes shift/eV	Φ_PL_	*g*_abs_ ^c^×10^–4^	Δε^d^/cm^–1^ M^–1^	*g*_lum_ ^e^× 10^–4^	*B*_CPL_ ^f^/M^–1^ cm^–1^	*E*_HOMO_ ^g^/eV	*E*_LUMO_ ^g^/eV
**[9,9]**	335	464	15.9	2.94	1.03	0.80	6.3	100	6.7	42.6	–5.89	–1.76
**[11,9]**	334	456	16.2	2.99	0.99	0.80	8.6	140	3.4	23.3	–5.88	–1.72
**[11,11]**	335	437	16.8	3.03	0.86	0.95	11.1	184	7.8	62.3	–5.92	–1.72
**[9,0]**	331	446	7.4	2.91	1.09	0.80	7.3	65	8.0	23.7	–5.90	–1.71
**[11,0]**	330	452	9.0	2.94	1.01	0.95	6.9	61	4.0	14.5	–5.93	–1.69
**[0,9]**	328	460	8.0	2.95	1.08	0.79	2.6	20	2.0	3.2	–5.87	–1.66
**[0,11]**	330	438	9.4	3.03	0.93	0.95	6.8	30	2.3	9.4	–5.91	–1.65

a All optical measurements were performed in CH_2_Cl_2_. Chiroptical properties from *(R*,*R*,*R*,*R)*-enantiomers.

b Optical bandgap, determined from the onset of the longest wavelength absorption.

c At the absorption maximum.

d Δε = molar extinction from ECD measurement.

e At the emission maximum.

f *B*_CPL_ = ε × ϕ_PL_ × *g*_lum_/2.

g Calculated at the PW6B95/def2-QZVP//PBEh-3c level of theory. For more information see Supporting Information.

**Figure 4:**
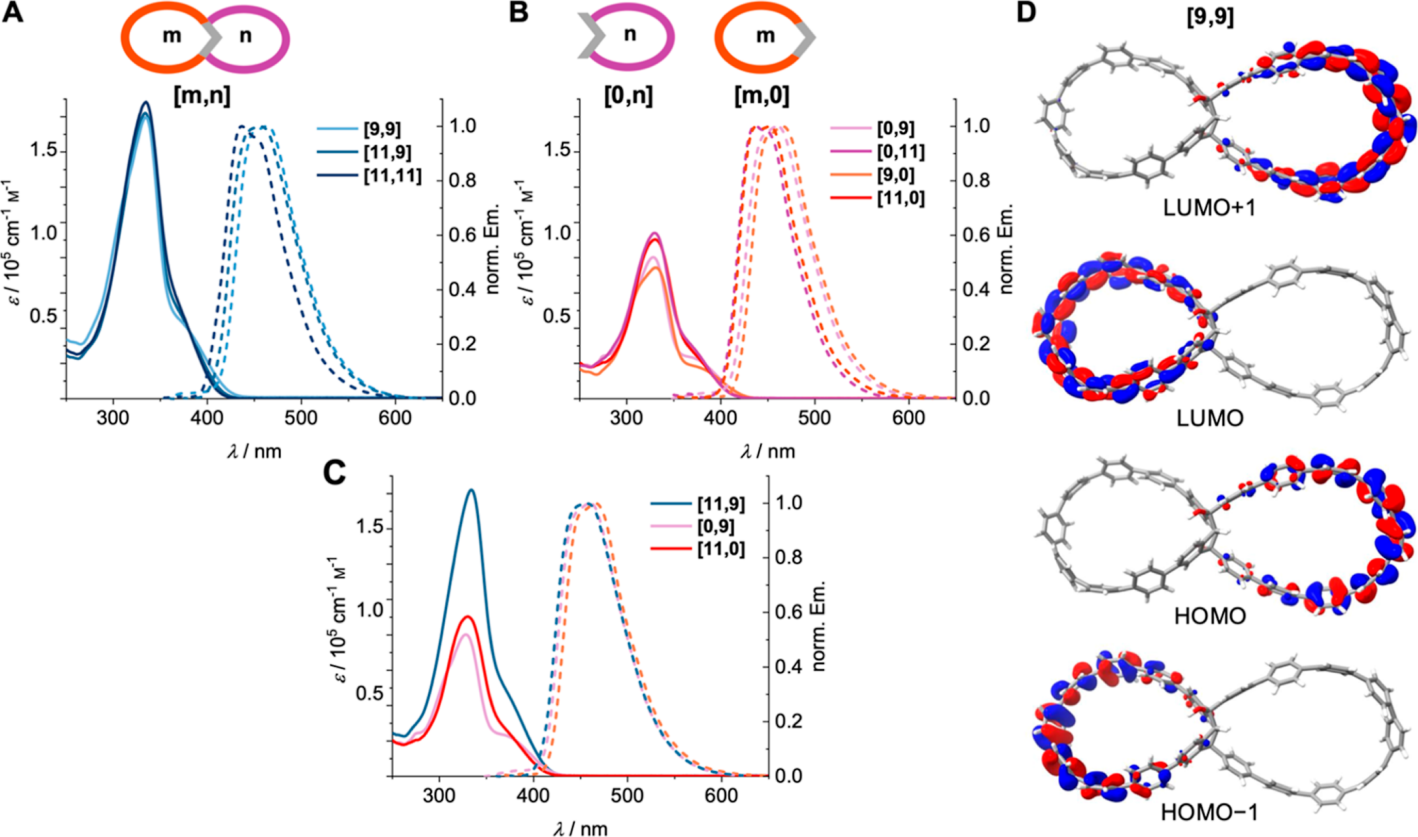
(A–C) Absorption (10^–5^ to 10^–6^ m in CH_2_Cl_2_, solid lines) and emission (10^–6^ to 10^–7^ m in CH_2_Cl_2_, dashed lines, λ_exc_ = 333 nm) spectra of the double nanohoops (A), of the single nanohoop reference compounds (B), and of double nanohoop **[11,9]** together with its two respective reference compounds **[0,9]** and **[11,0]** (C). (D) Calculated molecular orbitals of double nanohoop **[0,9]** (PW6B95/def2-QZVP//PBEh-3c level of theory).

For **[9,9]** and **[11,11]**, the S_0_ → S_1_ transition mostly consists of the HOMO–1 → LUMO transition, while for **[11,9]**, it is the HOMO → LUMO+1 transition that contributes to the S_0_ → S_1_ transition. In all simulated spectra, one excitation is particularly strong, which reflects in the oscillator strength and the maximum of the experimental spectra. The main transitions involved in the absorption maxima are those from HOMO – 1 → LUMO and HOMO → LUMO + 1 for the reference single nanohoops. In the double nanohoops orbitals from HOMO – 3 to LUMO + 3 contribute to this band with oscillator strengths amounting to more than the sum of the single reference systems. Interestingly, in the frontier molecular orbitals of all double nanohoops the electron density is located either on the left loop “*n*” or on the right loop “*m*”.

In the strongest calculated excitation, most of the transitions take place between orbitals of the same type—*n* → *n* or *m* → *m*. A single transition with more than 10% of contribution is of the *m* → *n* (HOMO – 2 → LUMO) type and is only observed for the smaller double nanohoops **[9,9]** and **[11,9]**. A selection of molecular orbitals of one representative double nanohoop are shown in Figure 4D, and calculated HOMO and LUMO energies are given in Table 1.

The molar attenuation coefficients ε are significantly higher in the double nanohoops, consisting of two loops as chromophores, than in the single nanohoops as reference compounds (Table 1). This reflects the (close to) cumulative contribution of the oligo­(paraphenylene) subunits within the structure. The molar attenuation coefficients ε in the double nanohoops **[9,9]**, **[11,9]** and **[11,11]** range from (15.9–16.8) × 10^4^ m ^–1^ cm^–1^, while in the single nanohoops the values are significantly smaller ranging from (7.4–9.0) × 10^4^ m ^–1^ cm^–1^ for **[9,0]** and **[11,0]** and from (8.0–9.4) × 10^4^ m ^–1^ cm^–1^ for **[0,9]** and **[0,11]**. A comparison shows that the values for [9]–[11]­CPP with (12–13) × 10^4^ m ^–1^ cm^–1^ lie in between those of the double nanohoops and their single nanohoop reference compounds.^2^ This suggests that in the oligo­(paraphenylene) loops of the single and double nanohoops fewer conjugation paths exist that absorb in this region compared to in [*n*]­CPPs with cyclic conjugation. The same observation has been made for *meta*-[*n*]­CPPs (*m*[*n*]­CPPs) with broken conjugation, that also exhibit smaller ε values compared to their [*n*]­CPP relatives (i.e., 7.1 × 10^4^ m ^–1^ cm^–1^ for *m*[10]­CPP).^4^

All of the double nanohoops **[9,9]**, **[11,9]** and **[11,11]** and reference single nanohoops **[11,0]**, **[9,0]**, **[0,9]** and **[0,11]** exhibit strong fluorescence with emission maxima between 437 and 464 nm (Figure 4A–C and Table 1). A typical blue shift with increasing hoop size is observed from **[9,9]** to **[11,11]** and from **[9,0]** to **[0,11]**, which is known from [*n*]­CPPs^2^ and distinguishes such nanohoops from their linear (noncyclic) analogous, where an increase in length of the π-system usually causes a bathochromic shift of the emission. Interesting to note is that the photoluminescence quantum yield (Φ_PL_) for all double nanohoops and single hoop reference compounds containing a segment of nine conjugated phenylene rings (**[9,0]**, **[0,9]**, **[9,9]** and **[11,9]**) amounts to approximately 80%, while 95% are measured for all single and double nanohoops with a loop of 11 conjugated phenylene rings (**[11,11]**, **[11,0]** and **[0,11]**). This is in accordance with the size dependency observed for [*n*]­CPPs and *m*[*n*]­CPPs, where larger hoops show higher Φ_PL_ values because fluorescence is the major deactivation process from the S_1_-excited state, whereas the internal conversion is more dominant for the smaller ones.^4,62^ To the best of our knowledge, the quantum yields of 95% measured for **[11,11]**, **[11,0]** and **[0,11]** are among the highest values achieved to date for various types of nanohoops. Notably, the prior record-holding structure featured a triptycene unit, it shares notable structural (kinked) and electronic (aliphatic core of linking unit) similarities with the tetrahydroindenoindene unit.^63^

Although **[11,9]** contains two distinct hoops with different emission maxima (cf. the emission spectra of **[11,9]**, **[11,0]** and **[0,9]** in Figure 4C as well as the values in Table 1), only one emission band is visible that well overlaps with the emission of the smaller **[0,9]** single nanohoop. This might indicate an intramolecular energy transfer occurring from the larger to the smaller nanohoop in the double nanohoop **[11,9]**.

The excitation wavelength has no significant impact on the emission bands, and the excitation spectra closely mirror the absorption spectra (Figure S15). Additionally, the existence of a solvatochromic effect was probed for **[11,9]**, revealing no significant influence of solvent polarity (Figure S13), therefore an intramolecular charge transfer character can be excluded. These results suggest that both nanohoops may absorb light, with an excited-state energy transfer occurring from the larger to the smaller oligo­(paraphenylene) loop. A similar behavior was previously observed in triphenylene-bridged double nanohoops and CPP based polymers.^29,64^ In contrast, asymmetrically phenylene-bridged CPP double nanohoops exhibit an entirely different behavior, displaying anti-Kasha excited-state luminescence^31^ or a dual emission. However, such behavior is extremely rare and has not been observed in any other (double) nanohoops and is therefore most likely unique to phenylene-only double nanohoops.^23^

### Chiroptical Properties

Due to the central chirality of the connecting tetrahydroindenoindene unit with four stereocenters (see Figure 1), all double nanohoops as well as the reference single nanohoops are chiral. Racemization through rotation is not possible, only (hypothetically) by breaking and new-forming of CC-bonds. After separation of the enantiomers of all double and single nanohoops by HPLC using a chiral stationary phase (Chiralpak IG), we investigated their chiroptical properties by ECD and CPL spectroscopy. To assist in the evaluation of the spectra and assignment to enantiomers, we performed TDDFT calculations (PBE0/def2-TZVP)^65–70^ (Figures S106–S112) based on the PBEh-3c optimized geometries.

The ECD spectra of all the double nanohoop pairs of enantiomers display a mirror image relationship (Figure 5A) with three pronounced Cotton effects between 297–303 nm, 336 nm and 382–389 nm. The absorption maxima of the double nanohoops (cf. Figure 4A) with maxima at around 334 nm overlap with the strongest Cotton effect at 336 nm for all three double nanohoops.

**Figure 5:**
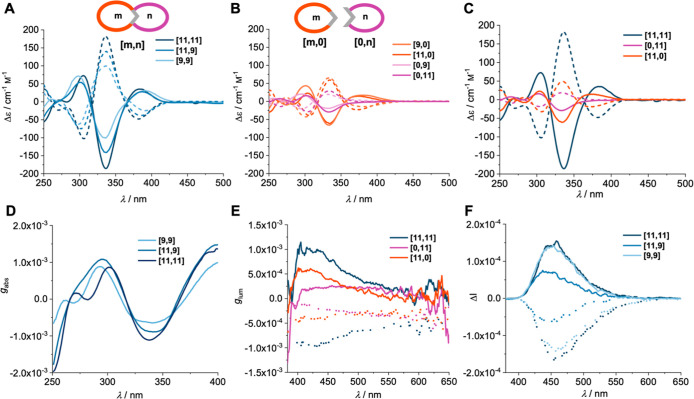
ECD spectra of (A) double nanohoops, (B) reference single nanohoops and (C) double nanohoop **[11,11]** in comparison to its single reference nanohoops (all in CH_2_Cl_2_ at rt; (*R*,*R*,*R*,*R*)-enantiomers in solid lines, (*S*,*S*,*S*,*S*)-enantiomers in dashed lines). (D) *g*_abs_ values of (*R*,*R*,*R*,*R*) double nanohoops. (E) *g*_lum_ values of (*R*,*R*,*R*,*R*) double nanohoops and single nanohoop reference compounds. (F) CPL spectra of the double nanohoops (10^–4^ m in CH_2_Cl_2,_ λ_exc._ = 320 nm.).

Based on TDDFT calculations we assigned the enantiomers to the respective (*R*,*R*,*R*,*R*)- (solid line) and (*S*,*S*,*S*,*S*)-enantiomers (dashed lines) (see Figures S105–S111; for configuration, see Figure 1). Interestingly, the molar extinction values (Table 1) increase with growing size of the double nanohoops from **[9,9]** with *g*_abs_ (Δε/ε) = 6.3 × 10^–4^ over **[11,9]** with 8.6 × 10^–4^ to **[11,11]** with 1.1 × 10^–3^ to an extend that exceeds that of a simple linear addition of the fragments (Figure 5D). This becomes particularly apparent when comparing the ECD spectra of the double nanohoops with those of their single nanohoop reference compounds (Figure 5B). Figure 5C shows the ECD spectra of double nanohoop **[11,11]** as representative examples and its reference single nanohoops **[11,0]** and **[0,11]**. The advantageous influence of the double nanohoop geometry, relative to the corresponding reference compounds, is clearly evident. As previously discussed, the enhanced molar attenuation coefficient ε primarily results from the cumulative contributions of both nanohoop components.

However, in the case of chiroptical properties, this effect significantly surpasses the additive contributions, strongly suggesting that an increase in hoop size amplifies the chiroptical response. For instance, the added Δε of **[9,0]** and **[0,9]** would amount to 84 cm^–1^ M^–1^, while **[9,9]** exhibits a Δε of 100 cm^–1^ M^–1^, corresponding to an “excess” of 16 cm^–1^ M^–1^. This amplification effect gets stronger with increasing double nanohoop size, where **[11,9]** shows an “excess” of 59 cm^–1^ M^–1^, and **[11,11]** demonstrates an even greater increase of 93 cm^–1^ M^–1^ over the cumulative values of their respective single nanohoop references. Thus, two trends can be observed: first, the double nanohoop geometry amplifies the ECD signal compared to the single nanohoop reference compounds, and, second, increasing the hoop size leads to a further amplification, as the comparison between the three double nanohoops in Figure 5A shows. These noncumulative amplifications with increasing size have been reported before in helicenes and have been explained by exciton-like interactions between helicene moieties.^71^ It can be expected that an even greater chiroptic enhancement will result from further elongation of the hoop size. Therefore, our approach demonstrates a novel method to enhance chiroptical absorption properties through geometric adaptation.

We next investigated the chiroptical emission properties by collecting CPL data from all double nanohoops and the respective reference single nanohoops (Figure 5E,F). A slight red shift of the Δ*I* maxima in the CPL spectra of the (*S*,*S*,*S*,*S*)-enantiomers in the studied series of nanohoops (Figure 5F) is likely due to an intrinsic spectrometer response, as we verified that the fluorescence emission spectra of both enantiomers were superimposable. When evaluating the CPL performance of the double nanohoops, the CPL brightness *B*_CPL_ serves as a more informative unit than the dissymmetry factor *g*_lum_, as it accounts for the chiral emission, the molar attenuation coefficient and Φ_PL_. All double nanohoops exhibit a clear enhancement in *B*_CPL_ compared to their corresponding reference single nanohoops (Table 1). This effect is especially pronounced in the **[11,11]** double nanohoop, which reaches a brightness of 62.3 m ^–1^ cm^–1^, substantially higher than those of its reference compounds **[0,11]** and **[11,0]**, which show brightness values of only 9.4 and 14.5 m ^–1^ cm^–1^, respectively. While these values are notably high, they are not unprecedented for curved π-systems. Moreover, the mention of CPL brightness is often omitted in the literature.^39,63^ This trend underscores a consistent amplification of chiroptical responses due to the double nanohoop architecture, likely driven by increased ε values that are not captured when considering *g*_lum_ alone.

## Conclusions

In this work, we introduced a novel class of chiral double nanohoops with a tetrahydroindeno­[2,1-*a*]­indene-5,10-diol linking unit that introduces central chirality through the presence of four stereocenters. By systematically varying the sizes of the nanohoop parts, we investigated their structural, optoelectronic and chiroptical properties. Our results show that the double nanohoops as well as their single nanohoop references exhibit high fluorescence quantum yields up to 95%, a new record among nanohoops, with larger nanohoops leading to an increased emission efficiency. Moreover, we observed an excited-state energy transfer from the larger to the smaller hoop moieties in the asymmetric double nanohoop **[11,9]**. The chiral nature of these nanohoops results in distinct ECD signals, with an enhancement effect that exceeds simple additive contributions from individual hoop components. This enhancement is also evident in the CPL brightness *B*_CPL_, which significantly increases to a value of 62.3 m ^–1^ cm^–1^, highlighting the synergistic effect of the double nanohoop design on chiral light emission. While the observed CPL values do not reach record-breaking levels, they are nonetheless remarkable for nanohoops, and underscore the potential of these double nanohoops as efficient chiral emitters. The modular synthetic path incorporating the chiral tetrahydroindeno­[2,1-*a*]­indene-5,10-diol linking unit can be employed in the future to change the structures of the connecting loops and thereby tune optoelectronic, chiroptical and structural properties.

## Supplementary Material



## Data Availability

The data underlying this study are available in the published article, in its Supporting Information, and openly available in Zenodo at 10.5281/zenodo.15913482.

## Notes

Materials and methods, synthetic manipulations, NMR spectra, mass spectra, ECD and CPL spectra, X-ray diffraction data and details on (TD)­DFT calculations (PDF)
