# Supplementary material for: Size and Geometry Impact the Chiroptical Properties of Double Nanohoops
Source: J Am Chem Soc. 2025 Nov 3;147(45):41610–9. doi: 10.1021/jacs.5c12590 (PMC12616693; doi:10.1021/jacs.5c12590)
Supplement: Supplementary file 1 [file ja5c12590_si_001.pdf]

Supporting Information

Size and Geometry Impact the Chiroptical Properties of Double Nanohoops

Philipp Seitz,[a] Luisa Rzesny,[a] Darleen Busse,[a] Xiaoshuang Xiang,[a] Mathias Hermann,[a] Lilian Estaque,[b] Grégory Pieters[b] and Birgit Esser[a]*

[a]Institute of Organic Chemistry II and Advanced Materials, Ulm University
Albert-Einstein-Allee 11, 89081 Ulm (Germany)
[b] Université Paris-Saclay, CEA, INRAE, Département Médicaments et Technologies pour la Santé (DMTS), SCBM, F-91191 Gif-sur-Yvette, France.

KEYWORDS : nanohoop, cycloparaphenylene, double nanohoop,circularly polarized luminescence, carbon nanomaterials

E-Mail: birgit.esser@uni-ulm.de

Table of Contents

1 Materials and Methods 1

2 Overview of Syntheses 4

3 Synthetic Procedures 6

4 Chiral-Stationary-Phase HPLC 17

5 Optical Properties 22

6 Electronic circular dichroism Spectra and g_{abs} Plots 26

7 Circularly Polarized Luminescence Measurements 34

8 NMR Spectra 41

9 Single Crystal X-Ray Diffraction 54

10 DFT Calculations 59

10.1 Optimized Geometries 59

10.2 Energies of Optimized Geometries 61

10.3 Frontier Molecular Orbitals 62

10.4 StrainViz Calculations 69

10.5 TD-DFT results 76

10.6 Calculated Absorption Spectra 86

10.7 Calculated ECD Spectra 88

10.8 Calculations on the Tetrahydroindenoindene unit 92

10.9 Coordinates of Optimized Geometries 95

11 List of References 113

1 Materials and Methods

Chemicals and solvents were purchased from ABCR, Acros Organics, Alfa Aesar, BLDPharm, Carbolution, Fisher Scientific, Fluorochem, Grüssing, Sigma-Aldrich, Thermo Scientific or VWR Chemicals and used directly without further purification unless otherwise specified. Moisture- or oxygen-sensitive reactions were carried out in dried glassware, heated under vacuum (≤ 10^{-2} mbar), using standard Schlenk techniques in a dry argon atmosphere (Argon 4.6 from MTI Industriegase). Solvents were degassed using the freeze-pump-thaw method or sparging with argon, hereafter denoted as argon-purged.Anhydrous (anh.) THF and toluene were obtained from a M. Braun solvent purification system (MB-SPS-800) and stored over molecular sieves (3 Å). Other anhydrous solvents were obtained by drying over activated molecular sieves (3 Å) for several days.[1] Other solvents were purchased and used in technical or laboratory grade.

Analytical thin layer chromatography (TLC) was carried out using silica gel-coated aluminum plates with a fluorescence indicator (Macherey-Nagel Alugram® Xtra SIL G UV254). Detection was carried out by using UV light (λ_{max} = 254 nm and 366 nm).

Flash column chromatography was carried out using silica gel 60, grain size 40–63 µm (230–400 mesh) from Macherey-Nagel.

Preparative size-exclusion chromatography (SEC) was performed on a JAI *LaboACE LC-7080 Plus* recycling preparative HPLC with a set of two JAI *JAIGEL-2HR Plus* columns. HPLC grade CH_2Cl_2 (amylene stabilized) or THF (inhibitor-free) was used as eluent.

Nuclear magnetic resonance (NMR) spectra were recorded at 298 K, unless otherwise noted, on the following spectrometers: Bruker *Avance Neo 400* (400.1 MHz (1H), 101.6 MHz (13C), 161.9 MHz (31P)) or Bruker *Avance Neo 600* (600.2 MHz (1H), 150.9 MHz (13C)). Chemical shifts are reported in parts per million (ppm, δ scale). ^{1}H NMR spectra are referenced to the residual proton signal of the respective solvent: $CDCl_3$: 7.26 ppm, CD_2Cl_2: 5.32 ppm. ^{13}C NMR spectra are referenced to the following signals: $CDCl_3$: 77.16 ppm, and CD_2Cl_2: 53.84 ppm.[2] Analysis followed first order, and the following abbreviations for multiplets were used: singlet (s), doublet (d), multiplet (m) and combinations therefor, *i. e.*, doublet of doublets (dd). Coupling constants (*J*) are given in Hertz (Hz). For analysis, MESTRENOVA V14.2.0 was used.

High-resolution mass spectra (HRMS) were measured on a BRUKER solarX Hybrid 7T FT-ICR *via* matrix-assisted laser desorption/ionization (MALDI) using *trans*-2-[3-(4-*tert*-butyl-phenyl)-2-methyl-2-propenylidene]malononitrile (DCTB) as matrix.

UV/Vis absorption spectra were measured on a SHIMADZU UV-3600i Plus UV-VIS-NIR spectrophotometer using Quartz (*Suprasil*) cuvettes (1 cm path length) from HELLMA ANALYTICS. Extinction coefficients were measured by serial dillution. Spectra were measured in 10 steps from 10^{-6} to 10^{-5} M.

Fluorescence spectra were measured on a PerkinElmer LS 55 luminescence spectrometer using Quartz (*Suprasil*) cuvettes (1 cm path length) from Hellma Analytics. Analyte concentrations were below 10^{-6} M.

Photoluminescence quantum yields ϕ_{PL} were determined using a Hamamatsu Quantaurus-QY (C11347) (Hamamatsu Photonics, Herrsching am Ammersee, Germany) for CH_2Cl_2 solutions. Analyte concentrations were below 10^{-6} M.

Electronic circular dichroism (ECD) and Circularly Polarized Luminescence (CPL)

ECD spectra were recorded using an OLIS DSM 245 spectrophotometer at 298 K at concentrations of 10^{-5} to 10^{-6} M in CH_2Cl_2. The concentration and hence the molar extinction $\Delta\varepsilon$ was determined by Lambert-Beer relationship and previously measured extinction coefficients. The circularly polarized luminescence (CPL) measurements were performed using a JASCO CPL-300 instrument at room temperature in 10*10 mm cell. Excitation wavelength and instrument parameters were adapted for every sample. Data pitch was set at 1 nm and spectra displayed are mean values of a minimum of 10 accumulations.

Semipreparative HPLC was used to separate the enantiomers on a chiral stationary phase using a Knauer Azura P2.1L Series with a semi-preparative *CHIRALPAK® IG* column (250 x 10 mm, 5 µm) at a flow rate of 2.5 or 5 ml min^{-1} and an injection volume of 1 mg racemate in 1 ml CH_2Cl_2.

Computational Details

All computations were done on the Justus High Performance Computing Cluster (bwForCluster JUSTUS 2 as part of the bwHPC facility).

To identify the low energy conformation, a global geometry optimization was performed for **[9,0]**, **[11,0]**, **[0,9]**, [**0,11]**, [**9,9]**, **[11,9]** and **[11,11]**, using the Global Optimizer Algorithm (GOAT) with the GFN2-xTB[3,4] method within ORCA 6.0.1[5–8]. The global minimum geometry was subsequently optimized with the TURBOMOLE 7.7.1[9] program package through density functional theory (DFT) calculations using the PBEh-3c[10–12] composite method with the Becke-Johnson damping function[13,14], and using the resolution-of-identity approximation (RI)[15–17] for the Coulomb integrals. A frequency calculation confirmed the global minimum and was used to determine the zero-point vibrational energy (ZPVE). To determine the length of the cavities the distance between the center of the tetrahydroindenoindene unit and the central

point of the opposite lying phenylene unit was measured in MERCURY 2025.1.1[18]. The maximum height was measured between atoms or centroids of the same geometrical type. Single Point calculations were performed on PW6B95-D4/def2-QZVP[19–24] level of theory and the electronic energy E_{SP}, as well as the frontier molecular orbitals (FMOs) and their energies were obtained. The UCSF CHIMERAX 1.5[25–27] programm with the SEQCROW 1.8.20[28,29] plugin was used to visualize the FMOs with an isovalue of ±0.02. The thermal corrections for free energy $G_{free,th}$ as well as for the enthalpy $H_{free,th}$ was obtained with the freeh script (with $T = 298.15$ K, $P = 0.1$ MPa and the default scaling factor = 0.9914) implemented in TURBOMOLE.

Absorption spectra and ECD spectra were calculated using time-dependent DFT calculations on PBE0/def2-TZVP[21,30] level of theory with random-phase approximation (RPA) singlet excitation.[31–33] In the 30 calculated excitations, molecular orbital contributions of 10% and higher are considered in the tables below. The outputs were converted to .xy files with a Gaussian band shape using SPECDIS[34,35] and plotted onto the experimental data with OriginPro 2023b[36]. FWHM values (σ/γ) for the UV/vis and ECD spectra were 0.2 and 0.25respectively.

Strain calculations on reference systems as well as on the double hoops were performed using the StrainViz[37] protocol with GAUSSIAN 16[38] on B3LYP/6-31G(d)[39–45] level of theory with a previous geometry optimization using the same functional. In the bond_scripts.py, the minimum was manually defined as 0. Fragmentation of the optimized geometries was done in CHIMERAX. To account for the unsymmetrical double hoop structures, a comparison of the strain in the reference systems with different fragments is shown below (Figure S 94 and Figure S 96). The total strain in all molecules was visualized using VMD 1.9.3[46]. The total_force.tcl was modified to view the oxygen atoms in red (using the colorid 32 for O atoms and thus removing the H atoms) and plotted onto the original total_force.tcl (which shows H atoms in white).

2 Overview of Syntheses

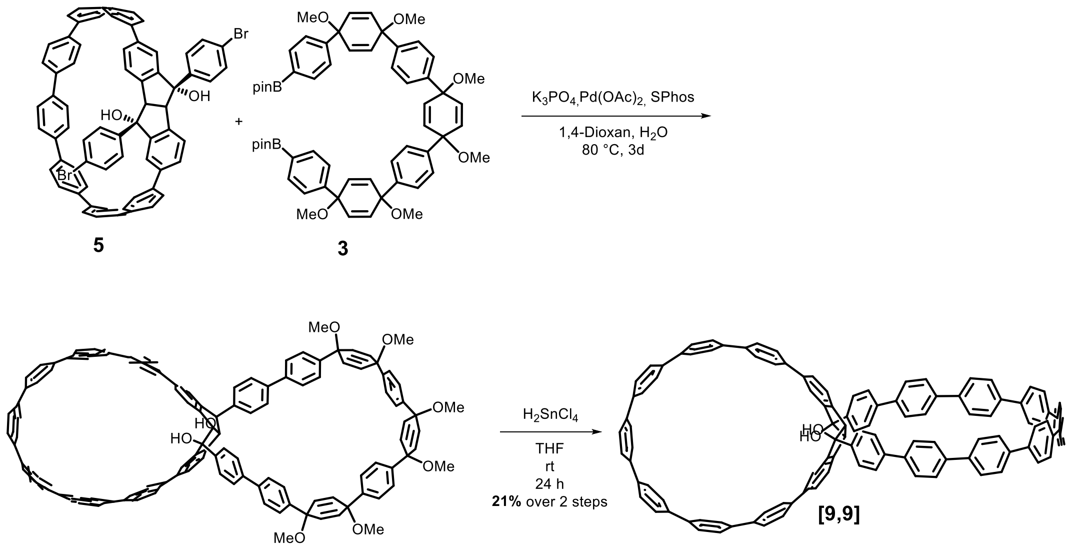

Scheme S 1: Synthetic route to double nanohoop **[9,9]**.

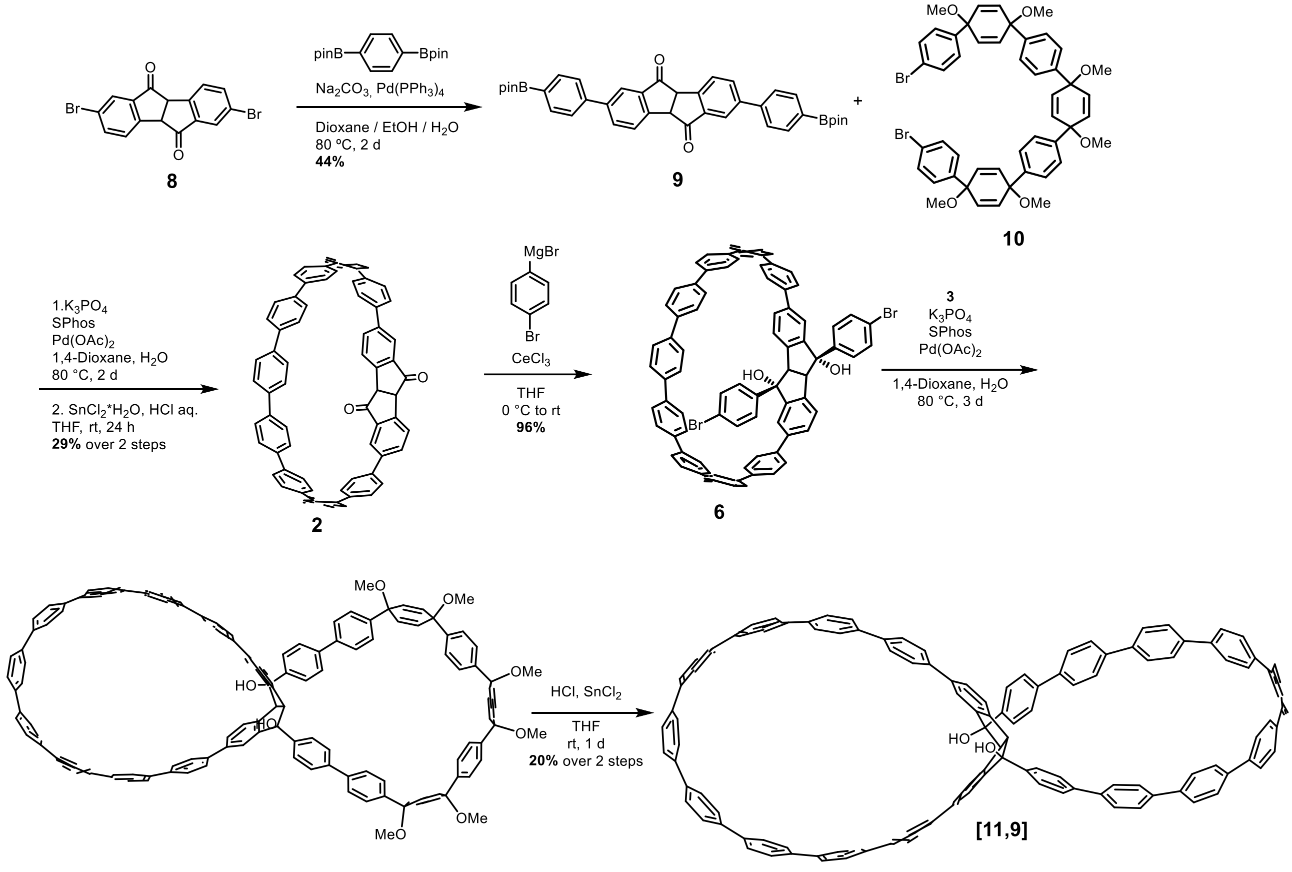

Scheme S 2: Synthetic route to double nanohoop **[11,9]**.

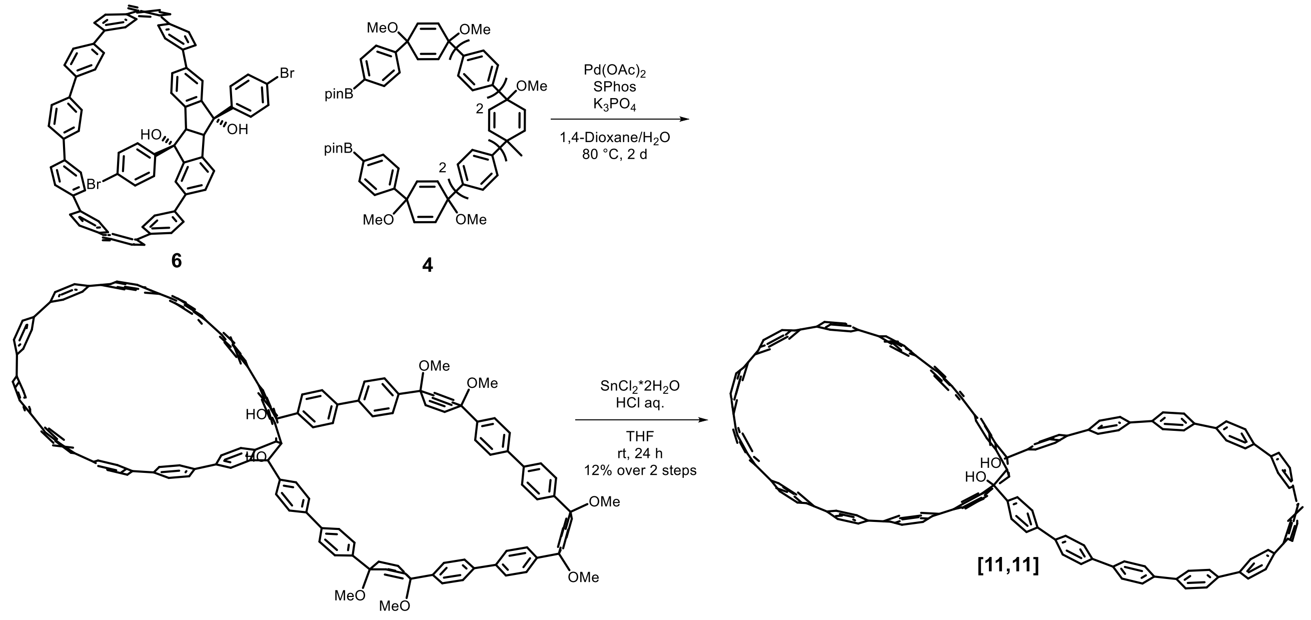

Scheme S 3: Synthesis of double nanohoop **[11,11]**.

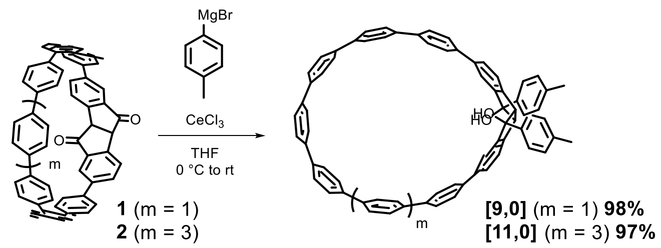

Scheme S 4: Synthesis of reference single nanohoops **[9,0]** and **[11,0]**.

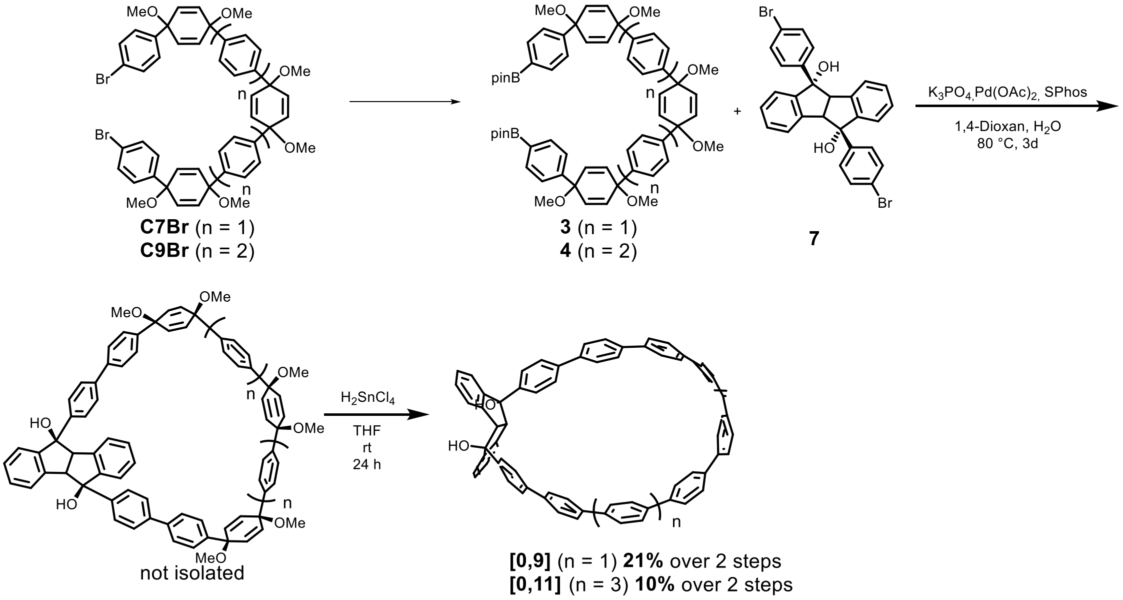

Scheme S 5: Synthesis of reference single nanohoops **[0,9]** and **[0,11]**.

3 Synthetic Procedures

Borylated C-shaped precursor 3

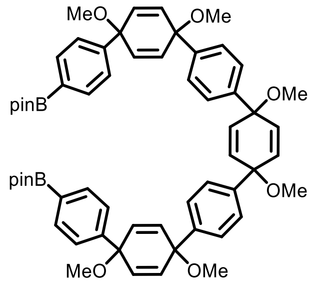

3

We were able to increase the yield to a quantitative conversion into the desired product by slightly changing the reaction times and concentrations and not using column chromatography for purification.

10[1] (220 mg, 250 μmol) was dissolved in anh. THF (4 mL), and the solution was cooled to −85 °C. After 10 min of stirring *n*-BuLi (2.7 M in toluene, 0.20 mL, 0.54 mmol, 2.2 eq.) was added. 5 min after its addition, 2-isopropoxy-4,4,5,5-tetramethyl-1,3,2-dioxaborolane (0.20 mL, 0.98 mmol, 3.9 eq.) was added rapdily, and the yellow solution was stirred between −95 °C and −75 °C for 30 min. The reaction mixture was allowed to warm to room temperature and was stirred for another 2 h, then water (10 mL) was added. CH_2Cl_2 (15 mL) was added to the mixture, the layers were separated, and the aqueous layer was extracted with CH_2Cl_2 (2 × 25 mL). The combined organic layers were washed with brine (50 mL) and dried over Na_2SO_4. The solvents were removed under reduced pressure, and the product (242 mg, 0.25 mmol, quant.) was obtained as a white solid.

***R*f** 0.72 (cyclohexane/ethyl acetate: 2/1); **^{1}H NMR** (500 MHz, $CDCl_3$): *δ* 7.77–7.75 (m, 4H), 7.42–7.40 (m, 4H), 7.36–7.34 (m, 8H), 6.09–6.08 (m, 12H), 3.43–3.42 (m, 18H), 1.34 (s, 24H); **^{13}C NMR** (126 MHz, $CDCl_3$)[2]: *δ* 146.6, 142.9, 142.8, 135.1, 133.5, 133.4, 133.3, 126.2, 125.4, 83.9, 75.0, 74.8, 74.7, 52.09, 52.07, 25.0.

---

[1] Was synthesized according to literature[47]

[2] Many signals overlap due to chemical similarity

2,7-Bis(4-(4,4,5,5-tetramethyl-1,3,2-dioxaborolan-2-yl)phenyl)-4b,9b-dihydroindeno[2,1-*a*]indene-5,10-dione (9)

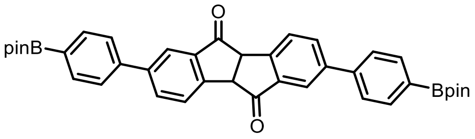

8 (1.94 g, 5.0 mmol, 1.0 eq.) and 1,4-bis(4,4,5,5-tetramethyl-1,3,2-dioxaborolan-2-yl)benzene (8.25 g, 25 mmol, 5.0 eq.) were dissolved in a mixture of 1,4-dioxane (60 mL), ethanol (30 mL), and H_2O (30 mL) in a round-bottom flask (250 mL), the mixture was degassed for 15 min by bubbling argon through the solution. $Pd(PPh_3)_4$ (115 mg, 0.1 mmol, 2.0 mol%) and Na_2CO_3 (5.25 g, 50.0 mmol, 10 eq.) were added, and the mixture was purged with argon for another 15 min. The mixture was stirred at 80 °C for 36 h and was allowed to cool down to rt. It was extracted with CH_2Cl_2 (3 x 50 ml), dried over Na_2SO_4, and the solvents were removed under reduced pressure. The crude product was purified by column chromatography (silica gel, PE/EtOAc: 4/1) and recrystallization from CH_2Cl_2/ethyl acetatr to afford the title compound as a white solid (1.55 g, 2.3 mmol, 47%).

***R*f =** 0.45 (EtOAc/PE: 1/4); **^{1}H NMR** (400 MHz, $CDCl_3$) δ 8.03–7.92 (m, 6H), 7.89–7.86 (m, 4H), 7.60–7.54 (m, 4H), 4.50 (s, 2H), 1.35 (s, 24H). **^{13}C NMR** (100 MHz, $CDCl_3$)[3] δ 201.6, 149.0, 142.6, 142.2, 135.64, 135.59, 135.1, 127.0, 126.6, 123.2, 84.1, 52.9, 25.0. **HRMS** (pos. MALDI): m/z calcd for $C_{40}H_{40}B_2O_6Na^+$ 661.2903 $[M+Na]^+$, found 661.2916.

[3] The signal of the quaternary carbon atom connected to boron has a very low intensity due to coupling with ^{10}B and ^{11}B

Diketo[9]CPP 2

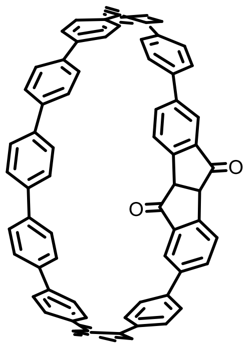

9 (319 mg, 500 µmol), C-shaped precursor **10** (438 mg, 500 µmol, 1.0 eq.) and K_3PO_4 (1.06 g, 5.00 mmol, 10 eq.) were dissolved in a mixture of 1,4-dioxane (300 mL) and H_2O (20 mL), and argon was bubbled through the solution for 1 h. $Pd(OAc)_2$ (58.0 mg, 0.258 mmol, 0.5 eq.) and SPhos (58 mg, 0.25 mmol, 0.5 eq.) were added under an argon atmosphere, and the mixture was stirred at 80 °C for 2 d. The reaction mixture was filtered over a Celite pad, and the solvent was removed under reduced pressure. The mixture was extracted with CH_2Cl_2 (3 x 100 ml), dried over Na_2SO_4. The remaining crude product was used without further purification due to extreme sensitivity towards slightly acidic conditions. $SnCl_2 \times 2H_2O$ (1.68 g, 7.5 mmol, 13.0 eq.) was dissolved in degassed THF (50 mL). Concentrated aq. HCl (37% w/w, 12 M, 0.83 mL, 10 mmol, 20.0 eq.) was added dropwise, and the resulting solution was stirred at rt for 30 min. In a separate flask the crude product (551 mg, 500 µmol) was dissolved in degassed THF (10 mL), and the resulting solution was added dropwise to the H_2SnCl_4 solution. The fluorescent solution was stirred at room temperature for 24 h. The reaction was quenched by addition of aq. KOH (2 M, 400 mL). The mixture was extracted with ethyl acetate (3 × 150 mL). The solvent was removed under reduced pressure. The residue was firstly purified by passing through a short flash silica gel chromatography and then isolated by GPC with CH_2Cl_2 as eluent. **2** was afforded as a yellow solid (35.2 mg, 42 µmol, 32%).

***R*f** = 0.5 (CH_2Cl_2/PE: 2/1); **^{1}H NMR** (400 MHz, $CDCl_3$) δ 7.99 – 7.85 (m, 6H), 7.73 – 7.50 (m, 36H), 4.54 (s, 2H). **HRMS** (pos. MALDI): m/z calcd for $C_{70}H_{44}O_2^+$ 916.3341[M]$^{\bullet+}$, found 916.3337; **^{13}C NMR** (151 MHz, $CDCl_3$)[4] δ 202.3, 148.8, 141.7, 140.2, 139.00, 138.98, 138.8, 138.5, 138.4, 138.22, 138.16, 137.4, 134.6, 134.2, 127.8, 127.7, 127.64, 127.59, 127.5, 127.33, 127.30, 127.28, 127.0, 122.8, 77.34, 77.2, 77.0, 53.6, 53.0.

[4] Signals overlap due to chemical similarity.

Double nanohoop precursor 6

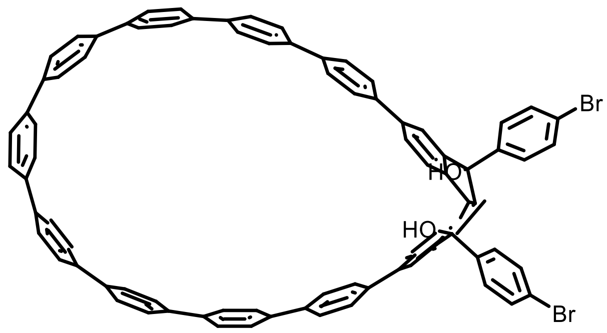

$CeCl_3$ (540 mg, 2.19 mmol, 20.00 eq) was dried at 135 °C for 3 h under vacuum and left to cool down to rt under argon, before anh. THF (5.0 ml) was added under an argon atmosphere. The resulting white suspension was stirred at rt for 18 h. [9]CPP-Diketone **2** (100 mg, 109 µmol) was added, and the resulting dispersion was stirred for 4h at 0 °C. The dibromobenzene-Turbo Grignard-reagent (0.45 M, 3.6 ml, 1.62 mmol, 14.9 eq.) was prepared according to literature[47] and was added to the $CeCl_3$/CPP-diketone-solution at 0 °C. The reaction mixture was stirred at 0 °C for 2 h and was allowed to warm to rt overnight. Subsequently, the reaction was quenched with aq. sat. NH_4Cl (20 ml), extracted with CH_2Cl_2 (3 × 30 ml), washed with brine (30 ml) and dried over $MgSO_4$. Column chromatography (cyclohexane/CH_2Cl_2 : 2/1 to 1/1) yielded the diol-cycle **6** as a yellow-green solid (129 mg, 105 µmol, 96%).

^{1}H NMR (400 MHz, acetone-*d*) δ 7.97 (d, J = 8.0 Hz, 2H), 7.78–7.56 (m, 40H), 7.50–7.42 (m, 4H), 7.36–7.28 (m, 4H), 5.15 (s, 2H), 4.11 (s, 2H); **^{13}C NMR** (151 MHz, CD_2Cl_2)[5] δ 148.5, 147.1, 140.9, 139.7, 139.21, 139.18, 139.1, 138.73, 138.72, 138.6, 138.5, 138.4, 131.8, 128.8, 127.90, 127.88, 127.83, 127.78, 127.76, 127.6, 127.5, 127.43, 127.39, 122.0, 121.5, 85.8, 59.4. **HRMS** (MALDI$^+$) m/z calc. for $C_{82}H_{54}Br_2O_2$ 1230.2470 $[M]^{\bullet+}$, found 1230.2479.

[5] Signals overlap due to chemical similarity.

Reference Hoop [11,0]

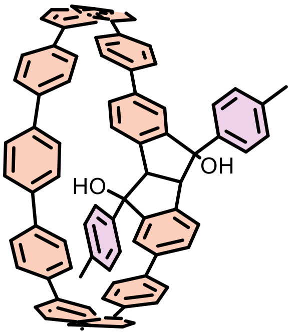

$CeCl_3$ (7.8 mg, 32 µmol, 20.00 eq) was dried at 135 °C for 3 h under vacuum and left to cool down to rt under argon, before anh. THF (4.1 ml) was added under an argon atmosphere. The resulting white suspension was stirred at rt for 18 h. [9]CPP-Diketone **2** (14.7 mg, 16.4 µmol) was added, and the resulting dispersion was stirred for 4h at 0 °C. A solution of *p*-tolylmagnesium bromide (1.0 M in THF, 0.24 ml, 240 µmol, 14.7 eq.) was added to the $CeCl_3$/CPP-diketone-solution at 0 °C. The reaction mixture was stirred at 0 °C for 2 h and was allowed to warm to rt overnight. Subsequently, the reaction was quenched with aq. sat. NH_4Cl (10 ml), extracted with CH_2Cl_2 (3 × 20 ml), washed with brine (30 ml) and dried over $MgSO_4$. Column chromatography (cyclohexane/CH_2Cl_2: 1/0 to 1/3) yielded the diol-cycle **[11,0]** as a yellow-green solid (17.4 mg, 15.8 µmol, 97%).

***R*f** = 0.37 (cyclohexane/CH_2Cl_2; 1/2); **^{1}H NMR** (400 MHz, CD_2Cl_2) δ 7.87 (d, *J* = 8.1 Hz, 2H), 7.70 – 7.52 (m, 40H), 7.21 – 7.09 (m, 8H), 4.20 (s, 2H), 2.59 (s, 2H), 2.29 (s, 6H); **^{13}C NMR** (101 MHz, CD_2Cl_2)[6] δ 149.1, 145.2, 140.4, 139.5, 139.23, 139.17, 139.1, 139.0, 138.9, 138.7, 138.48, 138.46,137.4, 129.3, 128.8, 127.9, 127.83, 127.77, 127.74, 127.71, 127.5, 127.41, 127.38, 125.6, 121.9, 85.9, 59.5; **HRMS** (MALDI$^+$) m/z calc. for $C_{84}H_{60}O_2$ 1100.4593 [M] $^{•+}$ found 1100.4566.

[6] Signals overlap due to chemical similarity.

Reference Hoop [9,0]

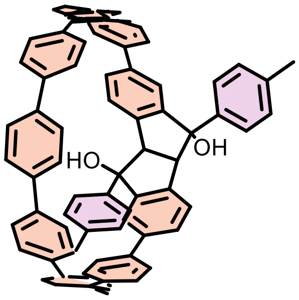

$CeCl_3$ (14.7 mg, 600 µmol, 20.00 eq) was dried at 135 °C for 3h under vacuum, before anh. THF (9.0 ml) was added to the cooled-down $CeCl_3$ under an argon atmosphere. The resulting white suspension was stirred at rt for 18 h. [7]CPP-Diketone **1**[7] (23.0 mg, 30.1 µmol) was added, and the resulting dispersion was stirred for 4 h at 0 °C. A solution of *p*-tolylmagnesium bromide (1.0 M in THF, 0.45 ml, 450 µmol, 15.0 eq) was added to the $CeCl_3$/CPP-diketone-solution at 0 °C. The reaction mixture was stirred at 0 °C for 2 h and was allowed to warm to rt overnight. Subsequently, the reaction was quenched with aq. sat. NH_4Cl (10 ml), extracted with CH_2Cl_2 (3 × 20 ml), washed with brine (30 ml) and dried over $MgSO_4$. Column chromatography (cyclohexane/ CH_2Cl_2 : 2/1 to 1/2) yielded the diol-cycle **[9,0]** as a yellow-green solid (28 mg, 45 µmol, 98%).

***R*f** = 0.31 (cyclohexane/CH_2Cl_2; 1/2); **^{1}H NMR** (400 MHz, CD_2Cl_2) δ 7.82 (d, *J* = 8.1 Hz, 2H), 7.67 – 7.63 (m, 2H), 7.63 – 7.45 (m, 30H), 7.19 – 7.07 (m, 8H), 4.17 (s, 2H), 2.50 (s, 2H), 2.29 (s, 6H); **^{13}C NMR** (101 MHz, CD_2Cl_2)[8] δ 149.0, 145.1, 140.1, 139.6, 138.9, 138.8, 138.61, 138.55, 138.31, 138.27 , 137.8, 137.4, 129.3, 128.6, 128.00, 127.96, 127.95, 127.9, 127.6, 127.5, 127.3, 127.2, 125.6, 121.4, 86.0, 59.3, 21.1; **HRMS** (MALDI$^+$) m/z calc. for $C_{72}H_{52}O_2$ 948.3967 $[M]^{\bullet+}$, found 948.3964.

---

[7] Was synthesized according to literature[59].
[8] Signals overlap due to chemical similarity.

Reference Hoop [0,9]

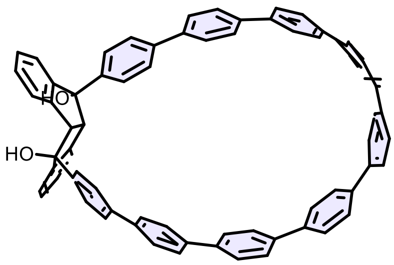

3 (97.3 mg, 100 µmol), diol **7**[9] (54.8 mg, 100 µmol, 1.00 eq.) and K_3PO_4 (212 mg, 1.00 mmol, 10.0 eq.) were added to a Schlenk Flask. SPhos (41.1 mg, 100 µmol, 1.0 eq.) and $Pd(OAc)_2$ (11.2 mg, 50.0 µmol, 0.50 eq.) were added inside a glovebox. Degassed 1,4-dioxane (70 ml) and degassed H_2O (5 ml) were added under inert conditions. The mixture was stirred at 80 °C for 3 d. The reaction mixture was allowed to cool down to rt and was filtered over a celite pad. The 1,4-dioxane was removed under reduced pressure, and the remaining mixture was extracted with CH_2Cl_2 (3 × 40 ml). The combined organic extracts were dried over Na_2SO_4. The solvent was removed under reduced pressure, and the product was used in the next step without further purification.$SnCl_2 \times 2H_2O$ (304 mg, 1.35 mmol, 16.0 eq.) was dissolved in degassed THF (27 ml). Aq. conc. HCl (12 M, 0.19 ml, 2.28 mmol, 26.8 eq.) was added via cannula, and the mixture was stirred for 1 h under an argon atmosphere. A solution of the crude methoxy-protected cycle (93 mg, 84 µmol) in degassed THF (2 ml) was added dropwise to the vigorously stirred solution of H_2SnCl_4. The mixture was stirred at rt for 18 h (the mixture turned yellow-orange over night). H_2O (20 ml) was added, and the crude reaction mixture was extracted with CH_2Cl_2 (3 × 30 ml) and was dried over Na_2SO_4. A short filter column (SiO_2; CH_2Cl_2/ethyl acetate; 1/1) followed by GPC separation (CH_2Cl_2) yielded the **[0,9]** as a yellow-green solid (14.1 mg, 15.3 µmol, 15%).

***R*f** = 0.35 (cyclohexane/ethyl acetate; 2/1); **^{1}H NMR** (400 MHz, CD_2Cl_2) δ 7.90 (d, *J* = 7.6 Hz, 2H), 7.61 – 7.34 (m, 38H), 7.14 – 7.08 (m, 4H), 3.79 (s, 2H), 2.73 (s, 2H); **^{13}C NMR** (151 MHz, CD_2Cl_2)[10] δ 147.8, 140.6, 139.3, 139.1, 139.05, 138.96, 138.72, 138.68, 138.3, 138.1, 132.4, 132.3, 129.3, 129.00, 128.96, 128.9, 128.5, 127.97, 127.96, 127.73, 127.69, 127.64, 127.62, 127.5, 126.9, 126.0, 125.4, 85.8, 60.2; **HRMS** (MALDI$^+$) m/z calcd. for $C_{70}H_{48}O_2$ $[M]^{\bullet+}$ 920.3654, found 920.3642.

[9] Was synthesized according to previously reported literature.[60]i

[10] Signals overlap due to chemical similarity.

Reference Hoop [0,11]

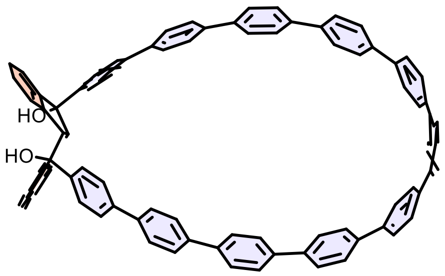

4 (40.5 mg, 36.0 µmol), **7** (19.7 mg, 36.0 µmol, 1 eq.), K_3PO_4 (76.4 mg, 0.36 mmol, 10 eq.), SPhos (14.8 mg, 36.0 µmol, 1 eq.) and $Pd(OAc)_2$ (4.04 mg, 18.0 µmol, 0.5 eq.) were dissolved in degassed 1,4-dioxane (21.6 mL) and degassed H_2O (1.5 mL) and stirred at 110 °C overnight. The mixture was allowed to cool down to rt and was extracted with CH_2Cl_2 (3 × 50 mL). The organic layer was washed with aq. sat. NaCl (20 mL) and dried over $MgSO_4$. The solvent was removed under reduced pressure and the crude product was used in the next step without further purification due to sensitivity towards slightly acidic conditions.

$SnCl_2$ × $2H_2O$ (122 mg, 0.54 mmol, 15.0 eq.) was dissolved in THF (10.8 mL). HCl (12 M, 0.08 mL, 0.90 mol, 25 eq.) was added, and the mixture was stirred for 1 h. The crude substrate mixture was dissolved in THF (2 mL) and the resulting solution was added dropwise to the $SnCl_2$ solution over the cour se of 5 min. The reaction mixture was stirred at rt overnight. The mixture was extracted with CH_2Cl_2 (3 × 30 ml). The organic layer was washed with brine (20 mL) and dried over $MgSO_4$. The solvent was removed under reduced pressure. A short filter column (SiO_2; CH_2Cl_2/ethyl acetate: 1/1) followed by GPC separation (CH_2Cl_2) yielded **[0,11]** (4.70 mg, 3.73 µmol, 10%) as a yellow solid. Chiral HPLC analysis utilizing a Chiralpak IG column with dichloromethane (CH_2Cl_2) as the mobile phase at a flow rate of 5 mL/min effectively achieved enantiomeric separation.

^{1}H NMR (400 MHz, CD_2Cl_2) δ 7.91 (d, *J* = 7.5 Hz, 2H), 7.67 – 7.55 (m, 36H), 7.53 – 7.47 (m, 4H), 7.45 (dd, *J* = 7.5, 1.6 Hz, 2H), 7.42 – 7.32 (m, 4H), 7.20 – 7.15 (m, 4H), 3.92 (s, 2H), 2.74 (s, 2H); **^{13}C NMR** (101 MHz, CD_2Cl_2)[11] δ 148.0, 140.5, 139.5, 139.3, 139.22, 139.16, 138.8, 138.7, 138.60, 138.56, 129.3, 129.0, 128.5, 127.9, 127.83, 127.80, 127.76, 127.61, 127.56, 127.5, 127.01, 126.0, 125.4, 85.7, 60.3; **HRMS** (pos. ESI): m/z calcd. for $C_{82}H_{56}O_2$ 1072.4280 $[M]^{\bullet+}$, found 1072.4281.

[11] Signals overlap due to chemical similarity.

Double Nanohoop [9,9]

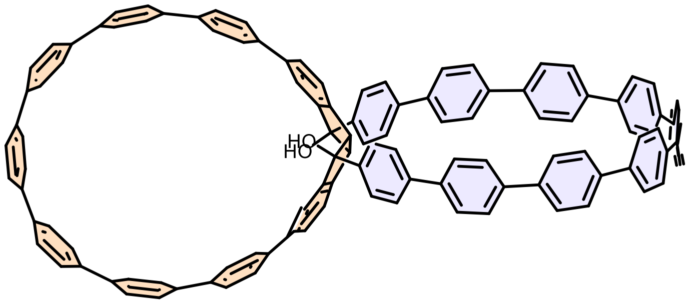

A Schlenk flask was charged with borylated C-shaped precursor **3** (48.4 mg, 49.8 µmol, 1.00 eq.), dibromonanohoop **1** (53.7 mg, 49.8 µmol, 1.00 eq.), K_3PO_4 (106 mg, 498 µmol, 10.0 eq.), SPhos (20.4 mg, 49.9 µmol, 1.00 eq.) and $Pd(OAc)_2$ (5.6 mg, 25 µmol, 0.50 eq.). The mixture was dissolved in a mixture of degassed 1,4-dioxane and H_2O (15/1, v/v, 32 ml) and stirred at 80 °C for 2 d. The reaction mixture was allowed to cool down to rt and brine (300 ml) was added. Subsequently the organic solvent was removed under reduced pressure and the remaining aqueous phase was extracted with EtOAc (3 × 150 ml) and dried over Na_2SO_4. The remaining crude product was used without further purification due to extreme sensitivity towards slightly acidic conditions.

$SnCl_2 \times 2H_2O$ (81.2 mg, 0.360 mmol, 15.0 eq.) was dissolved in degassed THF (6 ml). Aq. conc. HCl (12 M, 0.05 ml, 600 µmol, 24.6 eq.) was added via a cannula and the mixture was stirred for 1 h under an argon atmosphere to obtain H_2SnCl_4. A solution of the crude methoxy-protected doublehoop (40 mg, 24 µmol) in degassed THF (1 ml) was added dropwise over 10 min to the vigorously stirred H_2SnCl_4 solution. The mixture was stirred at rt for 1 d. H_2O (20 ml) and CH_2Cl_2 (20 ml) were added and the organic pase was extracted with CH_2Cl_2 (3 × 20 ml). A short filter column (SiO_2; CH_2Cl_2/ethyl acetate: 1/1) followed by size-exclusion chromatography (CH_2Cl_2) yielded the the double nanohoop **[9,9]** as a yellow-green solid (15.0 mg, 10.3 µmol, 21%).

***R*f** = 0.21 (cyclohexane/CH_2Cl_2: 1/1); **^{1}H NMR** (600 MHz, CD_2Cl_2) δ 7.89 (d, *J* = 8.2 Hz, 2H), 7.70 (dd, *J* = 8.2, 1.9 Hz, 2H), 7.63 – 7.47 (m, 62H), 7.22 – 7.18 (m, 4H), 3.83 (s, 2H), 2.67 (s, 2H); **^{13}C NMR** (151 MHz, CD_2Cl_2)[12] δ 148.1, 146.9, 140.4, 139.7, 139.41, 139.39, 139.3, 138.94, 138.92, 138.85, 138.7, 138.4, 138.58, 138.3, 138.2, 138.1, 137.8, 86.4, 59.7; **HRMS** (MALDI$^+$) m/z calc. for $C_{112}H_{74}O_2$ 1450.5689 $[M]^{\bullet+}$, found 1450.5686.

[12] Many signals overlap due to chemical similarity.

Double Nanohoop [11,9]

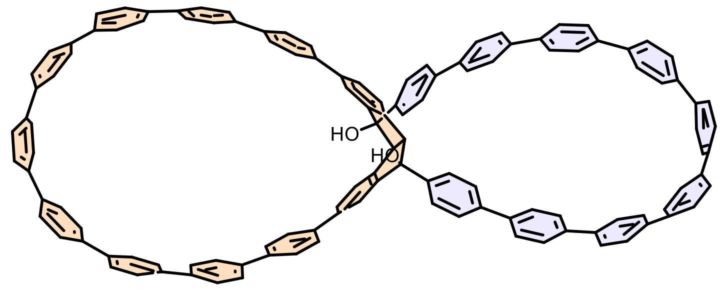

A Schlenk flask was charged with borylated C-shaped precursor **3** (48.4 mg, 49.8 µmol, 1.00 eq.), dibromonanohoop **6** (70 mg, 56.9 µmol, 1.00 eq.), K_3PO_4 (121 mg, 569 µmol, 10.0 eq.), SPhos (23.4 mg, 56.9 µmol, 1.00 eq.) and $Pd(OAc)_2$ (6.4 mg, 29 µmol, 0.50 eq.). The mixture was dissolved in a mixture of degassed 1,4-dioxane and H_2O (15/1, v/v, 36 ml) and stirred at 80 °C for 3 d. The reaction mixture was allowed to cool down to rt and brine (300 ml) was added. Subsequently the organic solvent was removed under reduced pressure and the remaining aqueous phase was extracted with EtOAc (3 × 150 ml) and dried over Na_2SO_4. The remaining crude product was used without further purification due to sensitivity towards slightly acidic conditions.

$SnCl_2$ × $2H_2O$ (193 mg, 854 µmol, 15.2 eq.) was dissolved in degassed THF (16 ml). Aq. conc. HCl (12 M, 0.12 ml, 1.44 mmol, 25.8 eq.) was added via cannula, and the mixture was stirred for 1 h under an argon atmosphere to obtain H_2SnCl_4. A solution of the crude methoxy-protected doublehoop (100 mg, 56 µmol) in degassed THF (2 ml) was added dropwise over 10 min to the vigorously stirred H_2SnCl_4 solution. The mixture was stirred at rt for 1 d. H_2O (20 ml) and CH_2Cl_2 (20 ml) were added, and the organic phase was extracted with CH_2Cl_2 (3 × 20 ml). A short filter column (SiO_2; CH_2Cl_2/ethyl acetate: 1/1) followed by size-exclusion chromatography (CH_2Cl_2) yielded the the double nanohoop **[11,9]** as a yellow-green solid (15.0 mg, 10.3 µmol, 20%).

***R*f** = 0.24 (cyclohexane/CH_2Cl_2: 1/1); **^{1}H NMR** (400 MHz, CD_2Cl_2) δ 7.96 (d, *J* = 8.1 Hz, 2H), 7.73 (dd, *J* = 8.1, 1.9 Hz, 2H), 7.67 – 7.49 (m, 70H), 7.22 (d, *J* = 8.5 Hz, 4H), 3.87 (s, 2H), 2.77 (s, 2H); **^{13}C NMR** (101 MHz, CD_2Cl_2)[13] δ 148.2, 147.0, 140.7, 139.7, 139.6, 139.4, 139.3, 139.21, 139.19, 139.0, 138.92, 138.91, 138.8, 138.7, 138.5, 138.4, 138.3, 138.0, 128.5, 128.0, 127.9, 127.83, 127.77, 127.73, 127.71, 127.66, 127.6, 127.5, 127.4, 126.9, 126.2, 122.1, 86.3, 59.8; **HRMS** (MALDI$^+$) m/z calc. for $C_{124}H_{82}O_2$ 1602.6315 $[M]^{\bullet+}$, found 1602.6303.

[13] Many signals overlap due to chemical similarity.

Double Nanohoop [11,11]

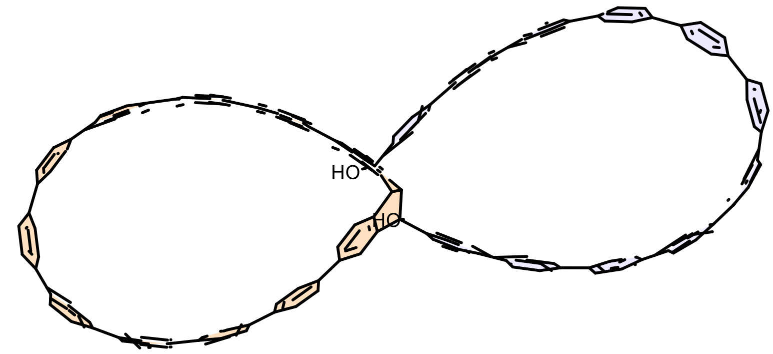

6 (20.5 mg, 19.0 µmol), **4** (21.4 mg, 19.0 µmol, 1 eq.), K_3PO_4 (40.3 mg, 0.190 mmol, 10 eq.), SPhos (7.80 mg, 19.0 µmol, 1 eq.) and $Pd(OAc)_2$ (2.10 mg, 9.50 µmol, 0.5 eq.) were dissolved in a mixture of degassed 1,4-dioxane (11.4 mL) and degassed water (0.76 mL) and stirred at 80 °C overnight. The mixture was extracted with CH_2Cl_2 (3 × 50 mL). The organic layer was washed with aq. sat. NaCl (20 mL) and dried over $MgSO_4$. The solvent was removed under reduced pressure and the crude product mixture was used with no further purification due to instability.

$SnCl_2 \times 2H_2O$ (64.3 mg, 0.29 mmol, 15 eq.) was dissolved in THF (4.5 mL). HCl (12 M, 0.04 mL, 0.48 mol, 25 eq.) was added, and the mixture was stirred at rt for 30 min. The crude mixture was dissolved in THF (2 mL), and the resulting solution was added dropwise to the $SnCl_2$ solution over the course of 5 min. The reaction mixture was stirred at rt overnight The mixture was quenched with aq. sat. $NaHCO_3$ (5 mL) and was extracted with CH_2Cl_2 (3 × 50 mL). The organic layer was washed with aq. sat. NaCl (20 mL) and dried over $MgSO_4$. The solvent was removed under reduced pressure. A short filter column (SiO_2; CH_2Cl_2/ ethyl acetate; 1/1), followed by GPC separation (CH_2Cl_2) yielded **[11,11]** (3.90 mg, 2.22 µmol, 12%) as a yellow solid. Chiral HPLC analysis utilizing a Chiralpak IG column with dichloromethane (CH_2Cl_2) as the mobile phase at a flow rate of 5 mL/min effectively achieved enantiomeric separation.

^{1}H NMR (400 MHz, CD_2Cl_2) δ 7.97 (d, *J* = 8.1 Hz, 2H), 7.73 (dd, *J* = 8.1, 1.7 Hz, 2H), 7.68 – 7.58 (m, 74H), 7.57 – 7.53 (m, 4H), 7.26 (d, *J* = 8.1 Hz, 4H), 3.97 (s, 2H), 2.77 (s, 2H). **^{13}C NMR** (151 MHz, CD_2Cl_2)[14] δ 148.4, 147.1, 140.7, 139.64, 139.58, 139.54, 139.45, 139.4, 139.3, 139.23, 139.20, 139.1, 138.9, 138.8, 138.7, 138.63, 138.56, 138.50, 138.46, 128.5, 127.9, 127.84, 127.82, 127.78, 127.64, 127.61, 127.60, 127.54, 127.52, 127.44, 127.0, 126.3, 122.1, 86.3, 59.8; **HRMS** (pos. ESI): m/z calcd. for $C_{136}H_{90}O_2$ 1754.6941 $[M]^{\bullet+}$, found 1754.6926

[14] Many signals overlap due to chemical similarity.

4 Chiral-Stationary-Phase HPLC

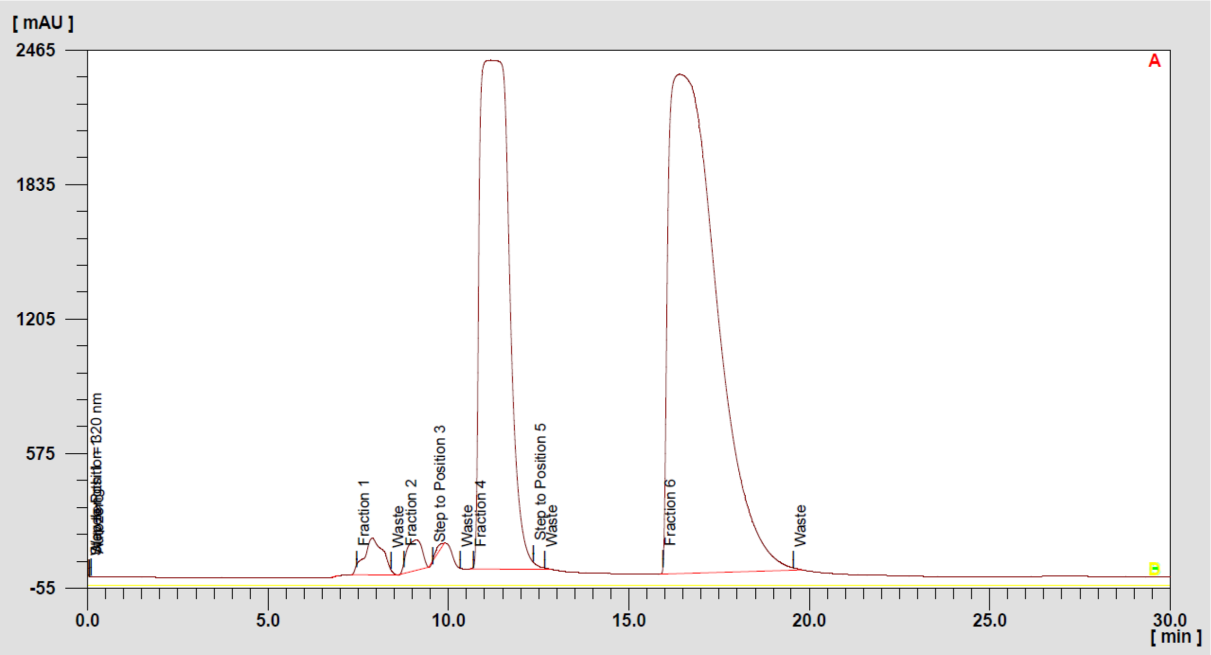

Figure S 1: HPLC chromatogram of **[0,9]** (flow rate: 5 ml/min; injection volume 1 ml; solvent: 100% CH_2Cl_2; Chiralpak IG) Fractions 1-3 are impurities.

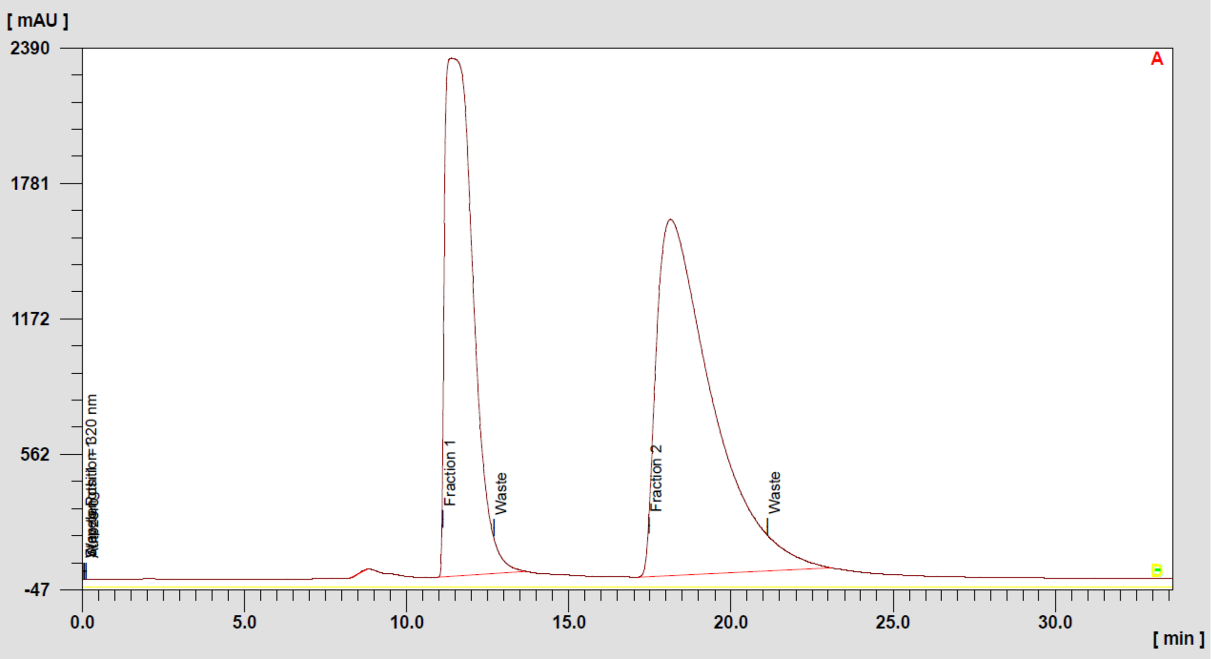

Figure S 2: HPLC chromatogram of **[9,0]** (flow rate: 5 ml/min; injection volume 1 ml; solvent: 100% CH_2Cl_2; Chiralpak IG).

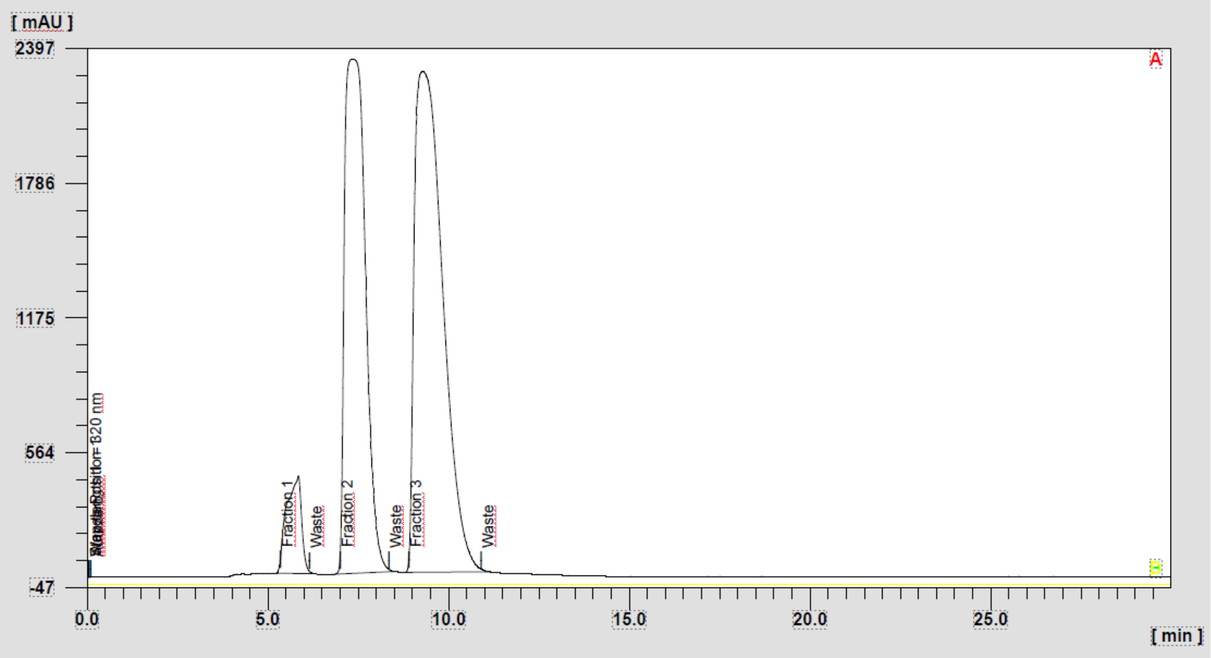

Figure S 3: HPLC chromatogram of **[0,11]** (flow rate: 5 ml/min; injection volume 1 ml; solvent: 100% CH_2Cl_2; Chiralpak IG; Fraction 1 = impurity).

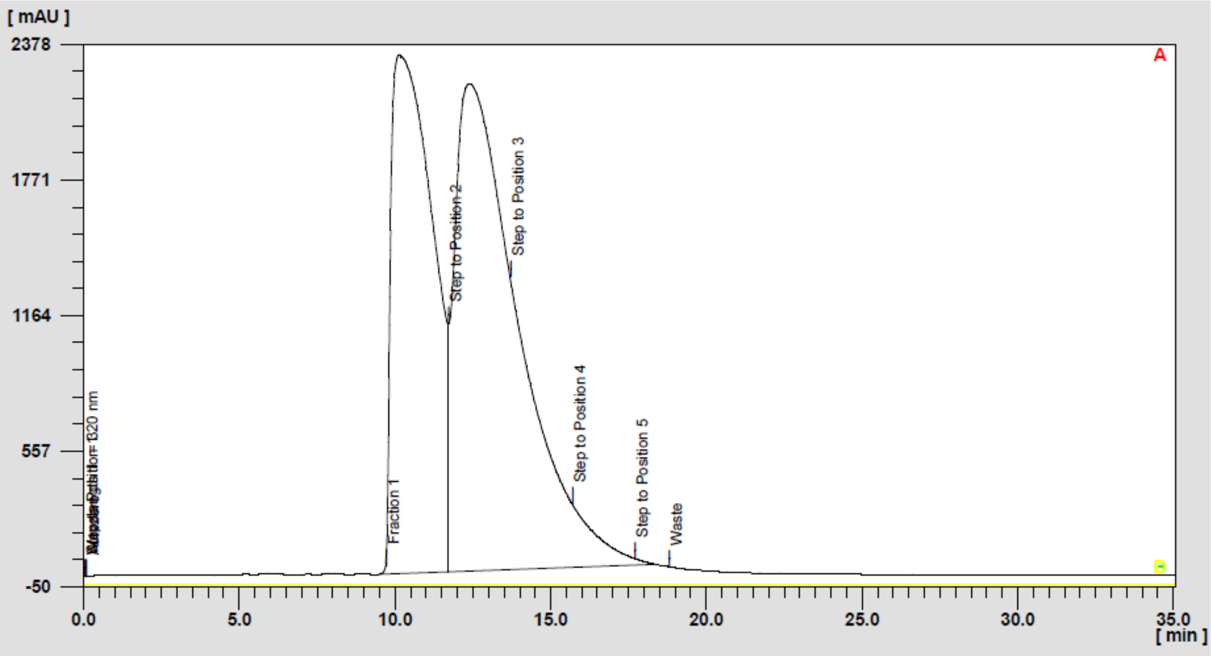

Figure S 4: First cycle of the HPLC chromatogram of **[11,0]** (flow rate: 5 ml/min; injection volume 1 ml; solvent: 100% CH_2Cl_2; Chiralpak IG). To secure baseline separation the respective fractions 1 and 2-5 were further separated afterwards.

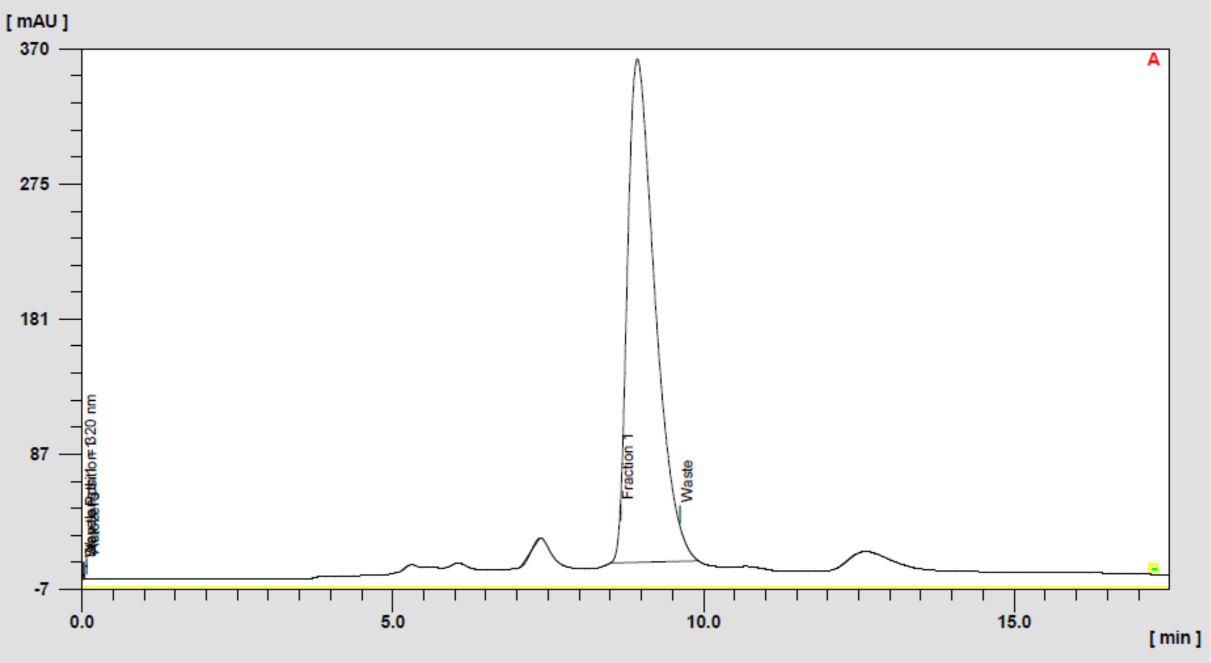

Figure S 5: HPLC chromatogram of **[11,0]** of fraction 1 after 2 more cycles (flow rate: 5 ml/min; injection volume 1 ml; solvent: 100% CH_2Cl_2).

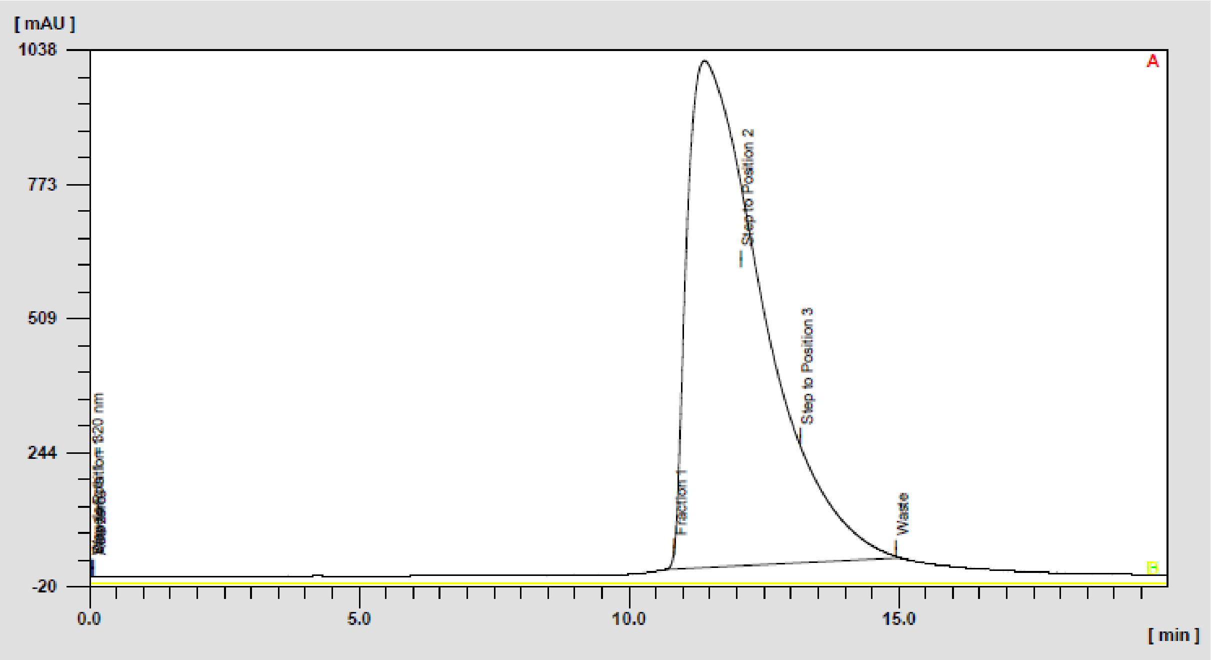

Figure S 6 HPLC chromatogram of **[11,0]** of fraction 2 after 2 more cycles (flow rate: 5 ml/min; injection volume 1 ml; solvent: 100% CH_2Cl_2).

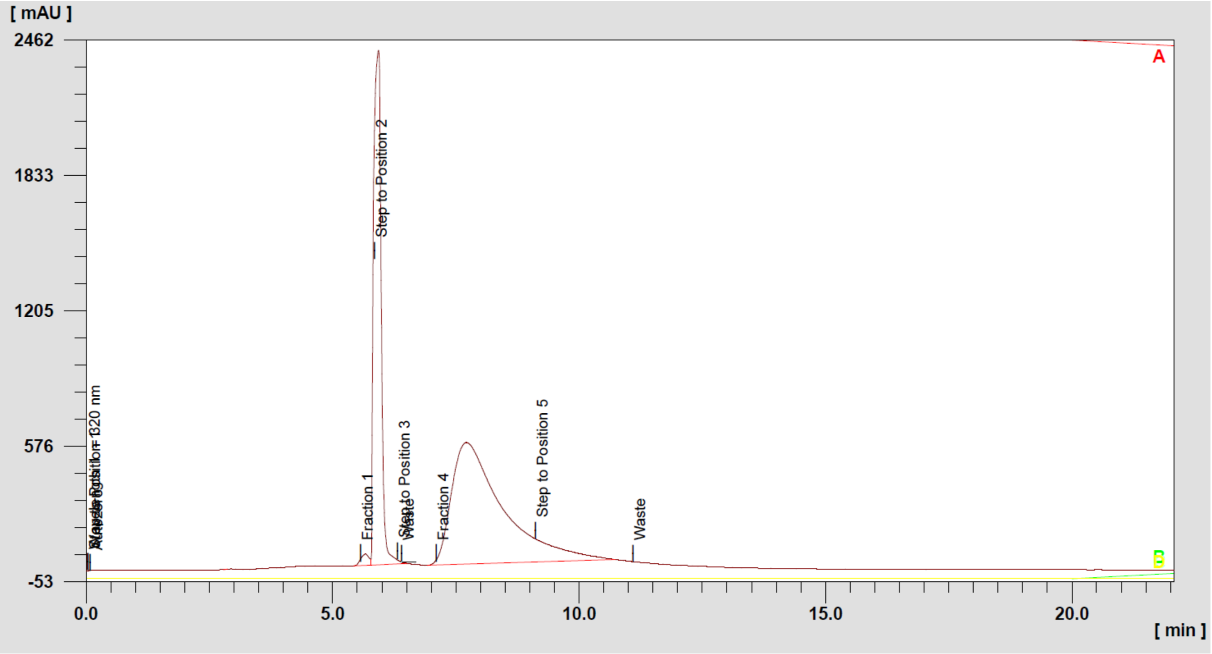

Figure S 7: HPLC Chromatogram of **[9,9]** (flow rate: 5 ml/min; injection volume 1 ml; solvent: 100% CH_2Cl_2; Chiralpak IG). Fraction 1 is a minor impurity.

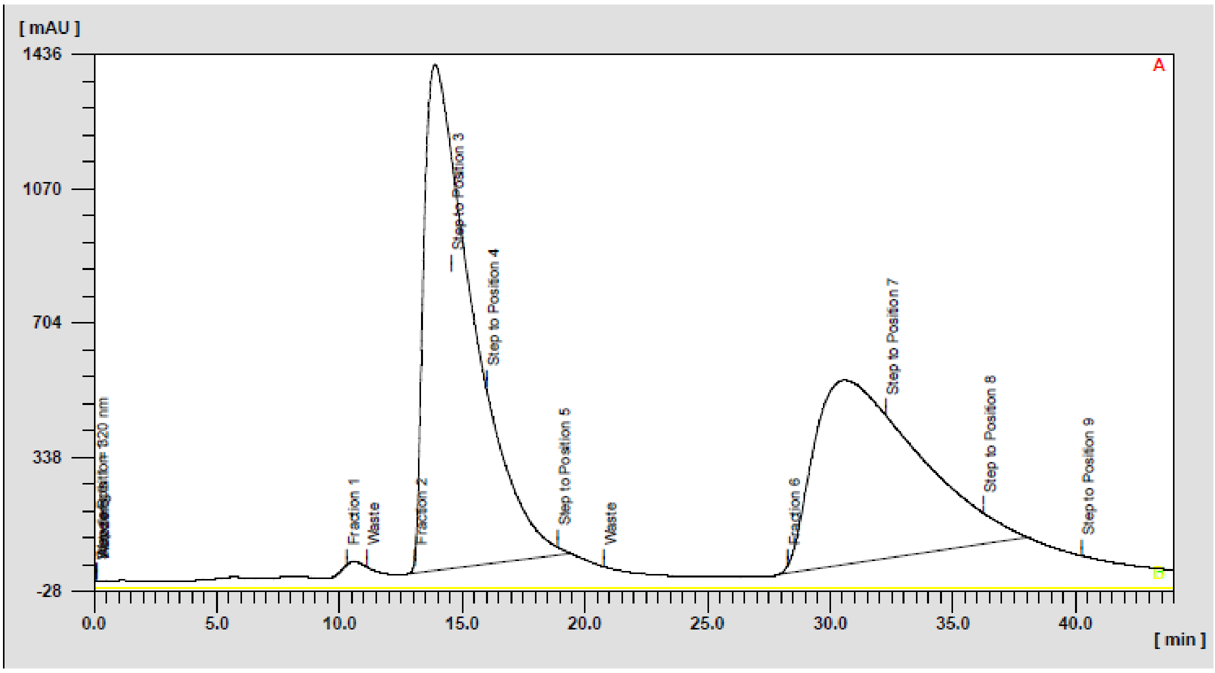

Figure S 8: HPLC Chromatogram of **[11,9]** (flow rate: 5 ml/min; injection volume 1 ml; solvent: 100% CH_2Cl_2; Chiralpak IG). Fraction 1 is a minor impurity.

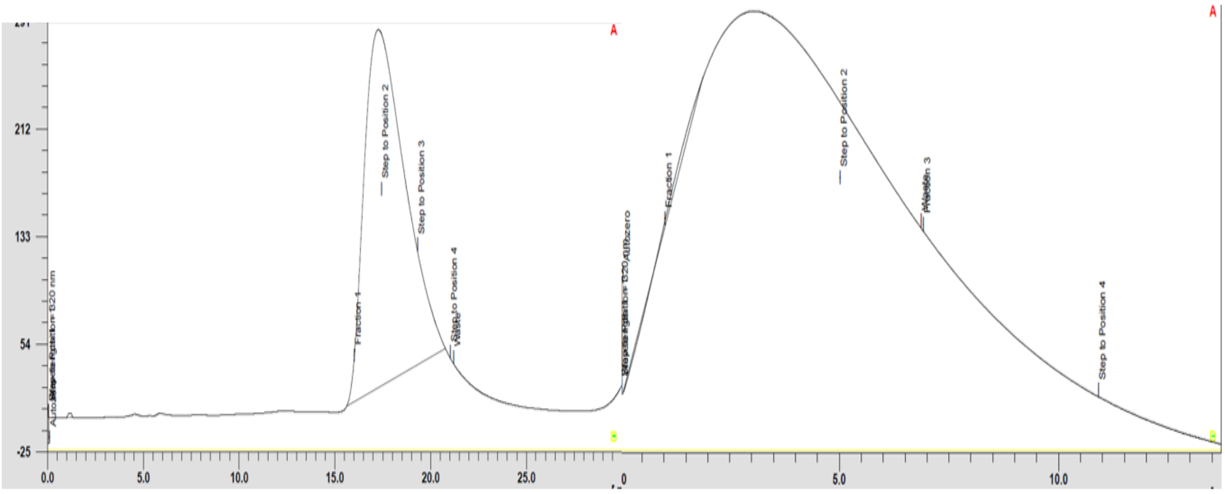

Figure S 9: HPLC Chromatogram of **[11,11]** (flow rate: 5 ml/min; injection volume 1 ml; solvent: 100% CH_2Cl_2; Chiralpak IG). Due to software issues the time scale and y-axis are not correct for the 2nd fraction.

5 Optical Properties

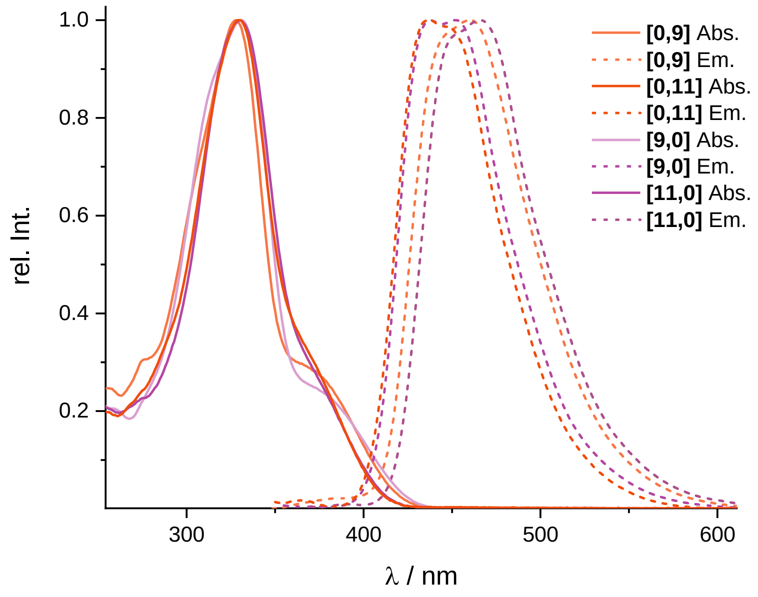

Figure S 10: Absorption (solid lines) and emission spectra (dashed lines, λ_{exc} = 334 nm) of **[0,9]**; **[0,11]; [11,0]** and **[9,0]** in CH_2Cl_2 (10^{-6} M).

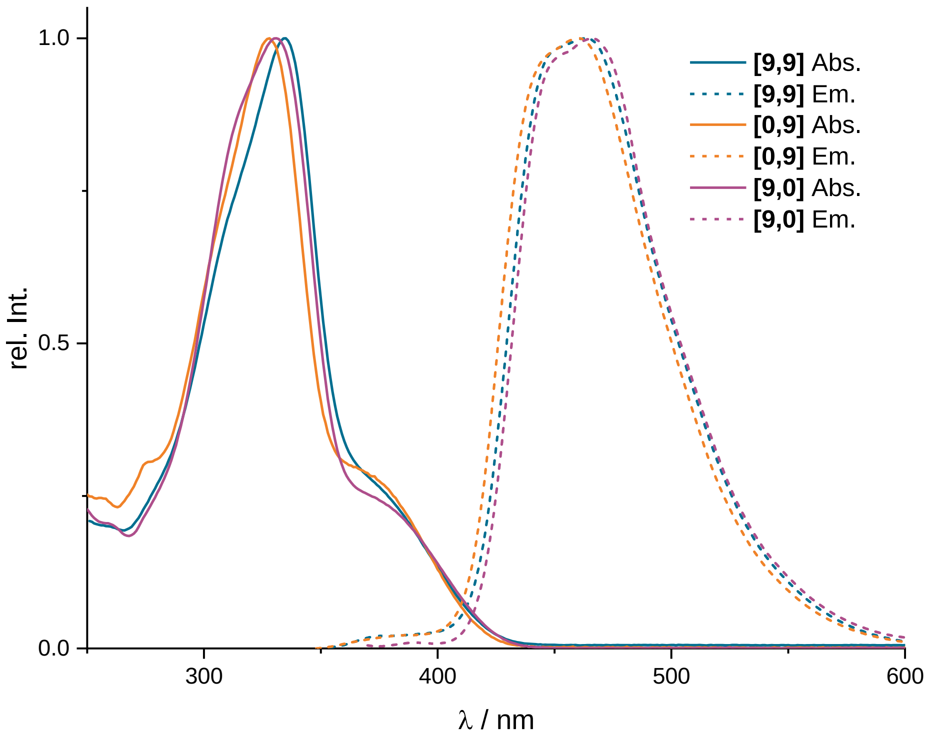

Figure S 11: Absorption (solid lines) and emission spectra (dashed lines, λ_{exc} = 334 nm) of **[9,9]**, **[9,0]** and **[0,9]** in CH_2Cl_2 (10^{-6} M).

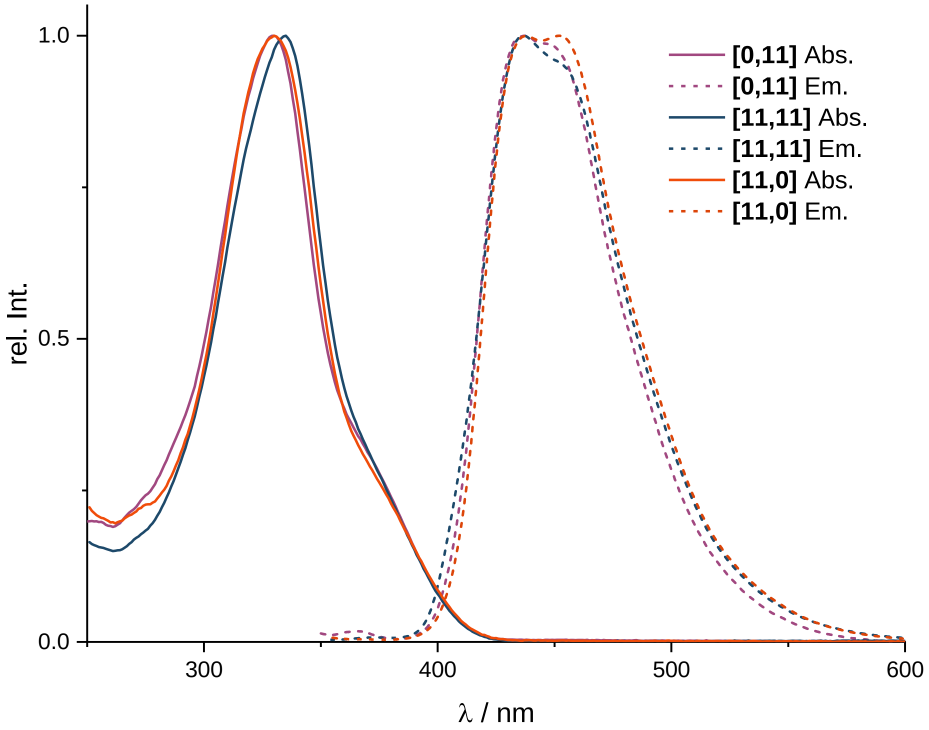

Figure S 12: Absorption (solid lines) and emission spectra (dashed lines, λ_{exc} = 334 nm) of **[11,11]**, **[11,0]** and **[0,11]** in CH_2Cl_2 (10^{-6} M).

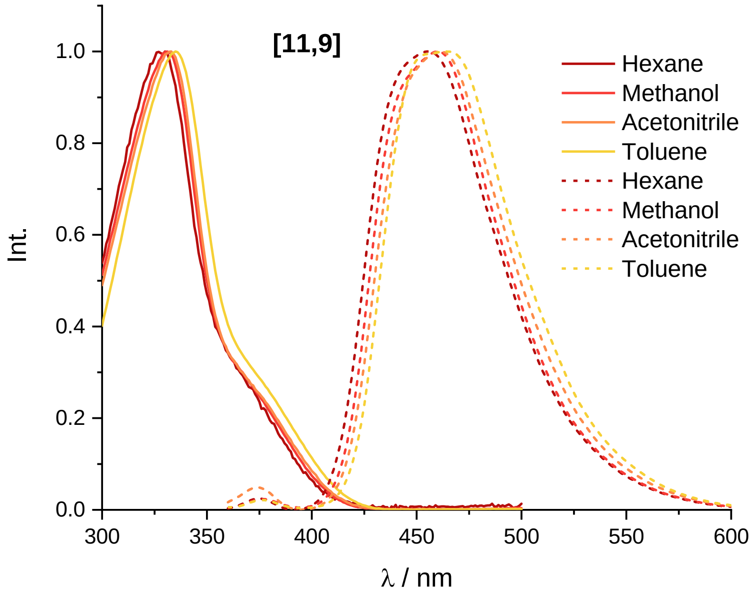

Figure S 13: Absorption (solid lines) and emission (dashed lines, λ_{exc} = 334 nm) spectra of **[11,9]** in different organic solvents (c = 10^{-5} to 10^{-6} M).

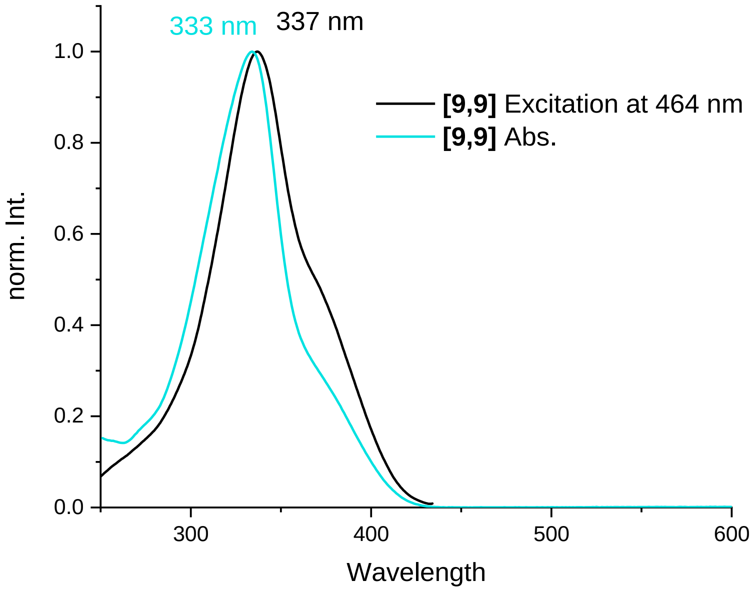

Figure S 14: Absorption (blue) and excitation spectrum (black) of **[9,9]** in CH_2Cl_2 (10^{-6} M).

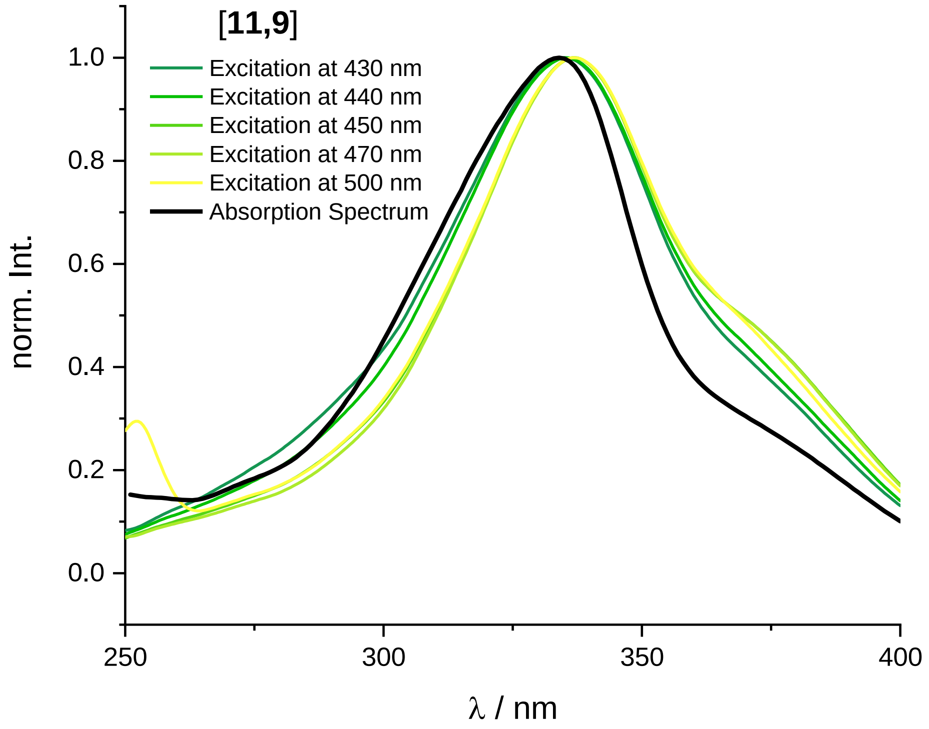

Figure S 15: Absorption (black) and excitation (colored) spectra of **[11,9]** in CH_2Cl_2 (10^{-6} M).

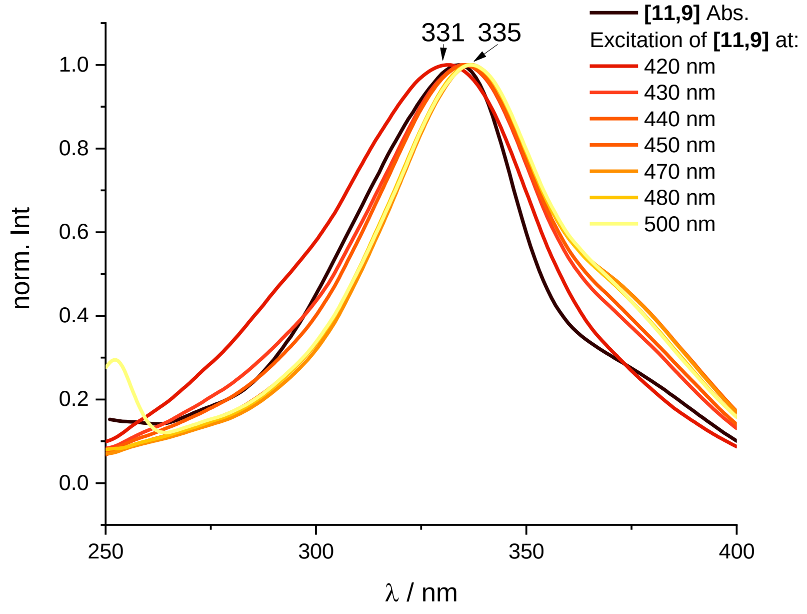

Figure S 16: Absorption (black) and excitation spectra (red to yellow) of **[11,9]** at different excitation wavelengths in CH_2Cl_2 (10^{-6} M).

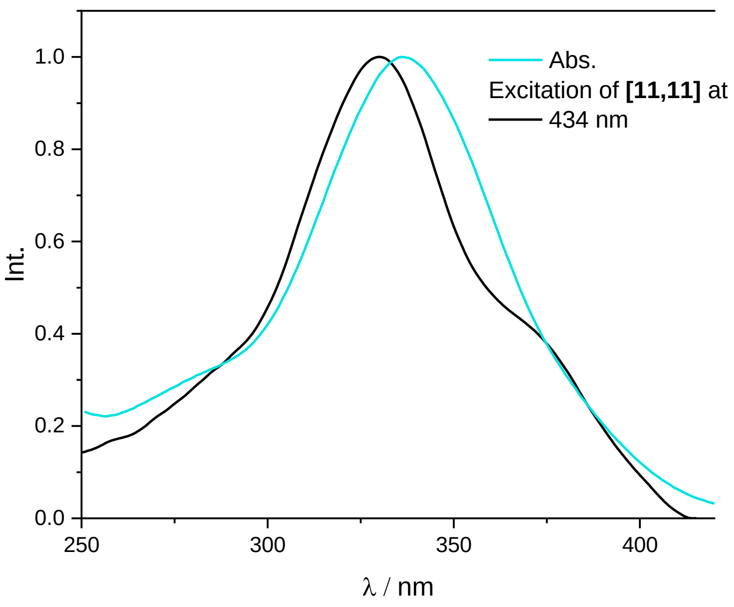

Figure S 17: Absorption (blue) and excitation spectrum (black) of **[11,11]** in CH_2Cl_2 (10^{-6} M).

6 Electronic circular dichroism Spectra and g_{abs} Plots

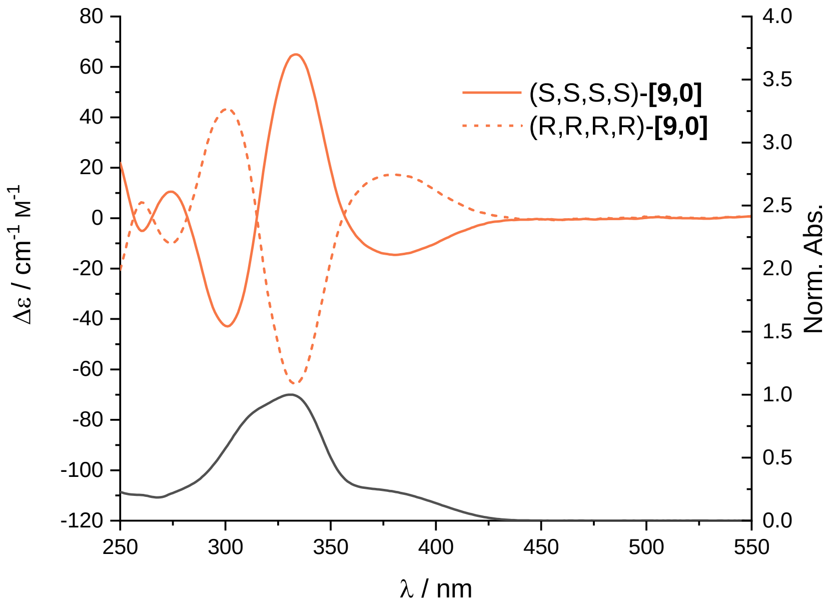

Figure S 18: CD (orange) and absorption (black) spectra of **[9,0]** in CH_2Cl_2 (10^{-6} M).

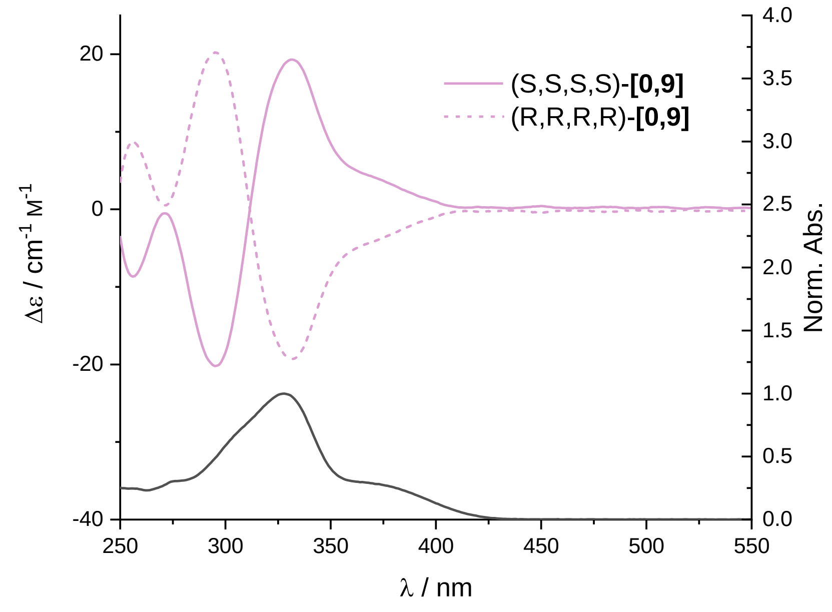

Figure S 19: ECD (purple) and absorption (black) spectra of **[0,9]** in CH_2Cl_2 (10^{-6} M).

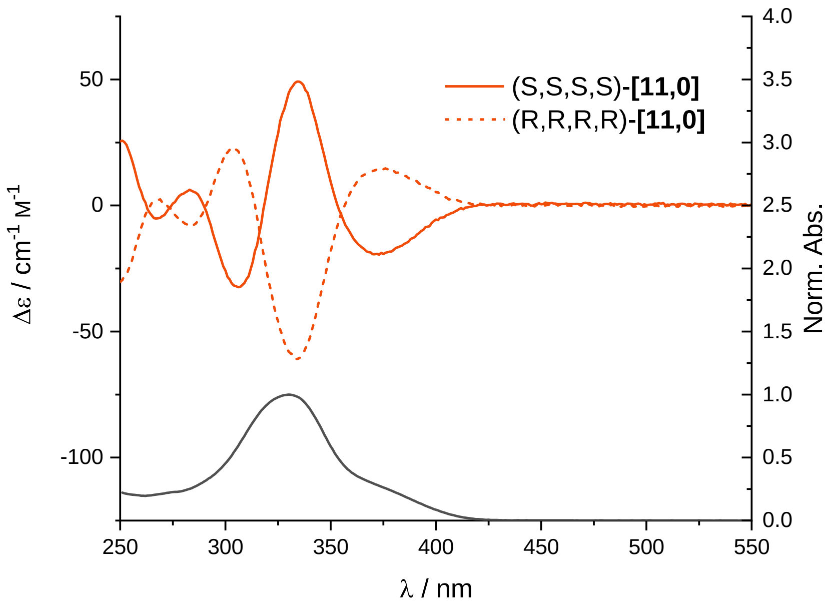

Figure S 20: ECD (orange) and absorption (black) spectra of **[11,0]** in CH_2Cl_2 (10^{-6} M).

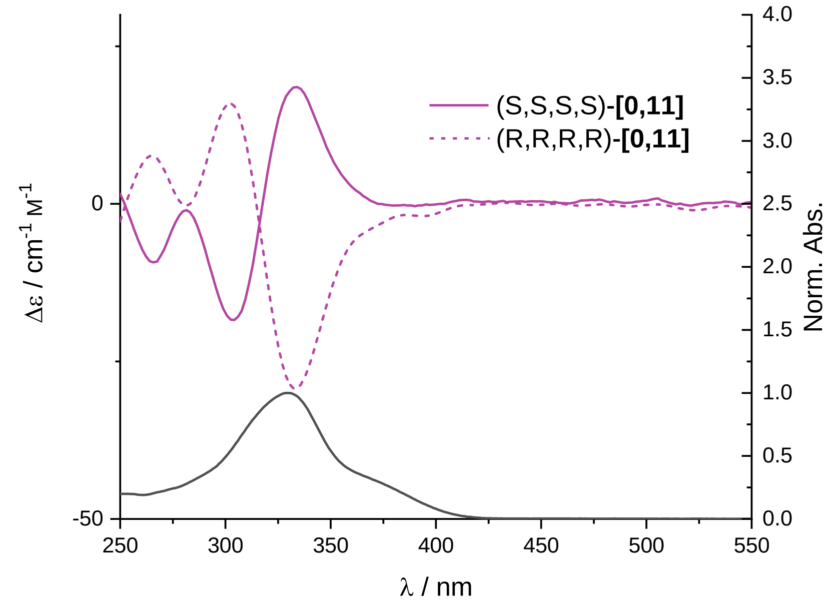

Figure S 21: ECD (purple) and absorption (black) spectra of **[0,11]** in CH_2Cl_2 (10^{-6} M).

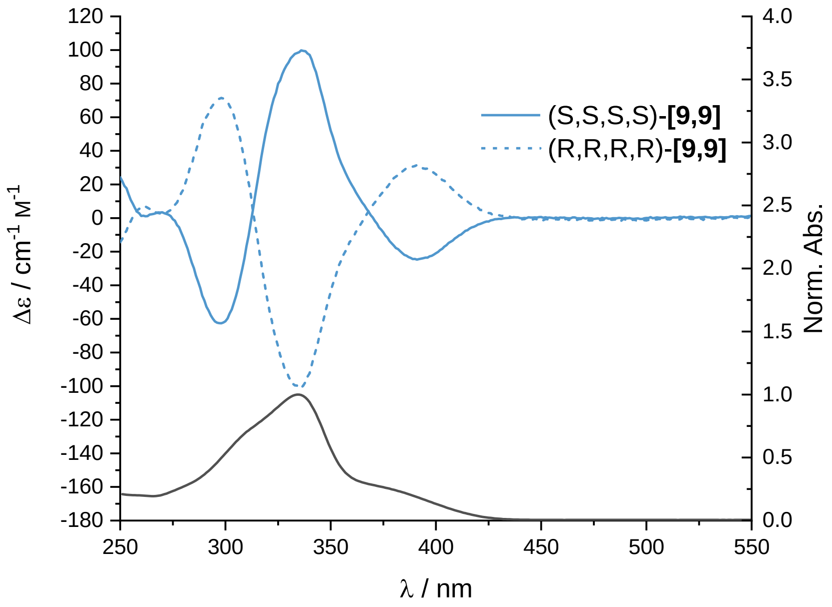

Figure S 5: ECD (blue) and absorption (black) spectra of **[9,9]** in CH_2Cl_2 (10^{-6} M).

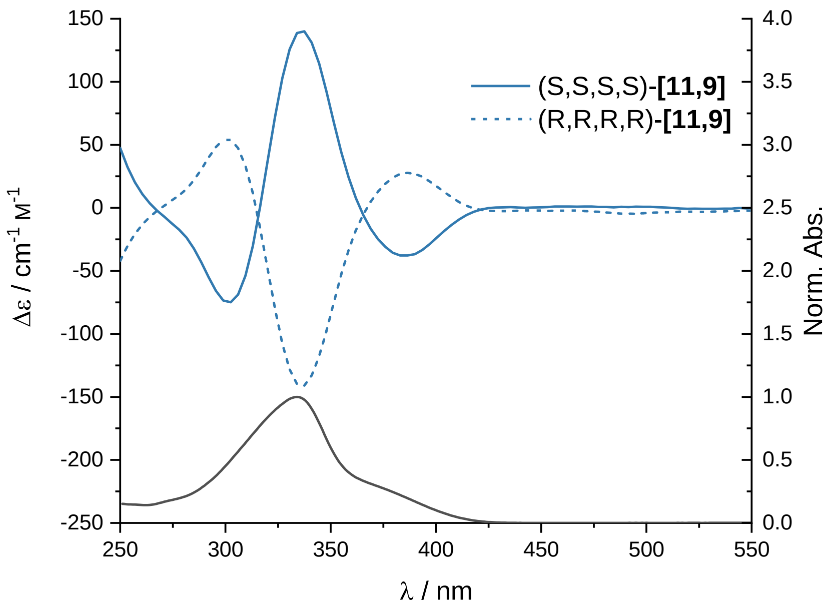

Figure S 22: ECD (blue) and absorption (black) spectra of **[11,9]** in CH_2Cl_2 (10^{-6} M).

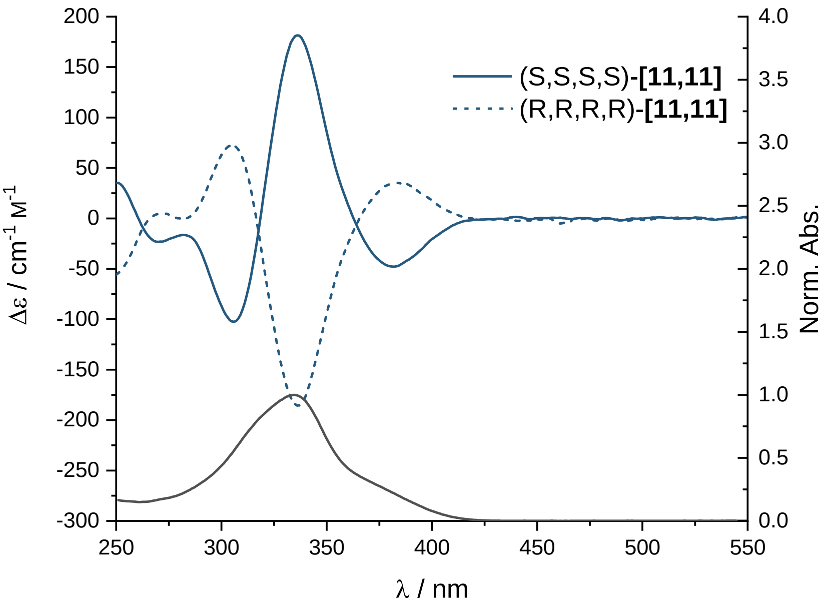

Figure S 23: ECD (blue) and absorption (black) spectra of **[11,11]** in CH_2Cl_2 (10^{-6} M).

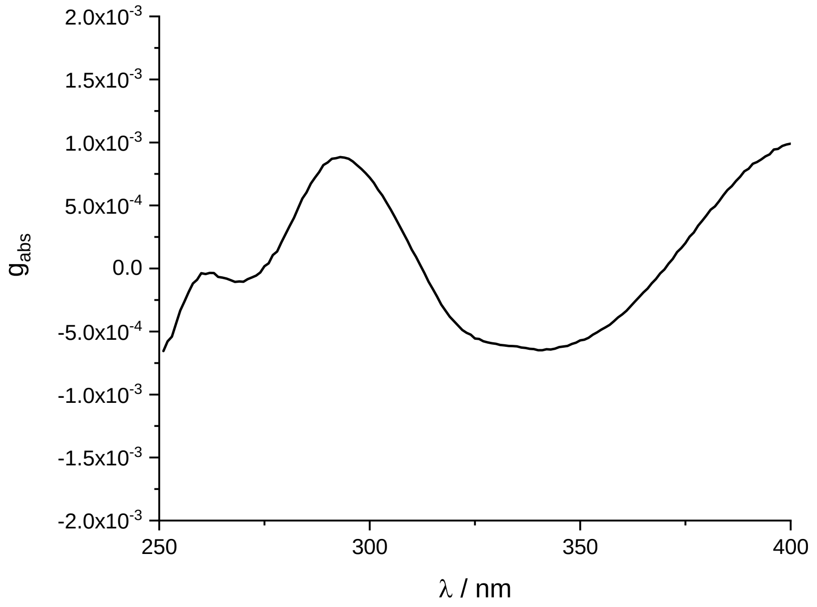

Figure S 24: Plot of g_{abs} of (*R*,*R*,*R*,*R*)-**[9,9]** in CH_2Cl_2 (10^{-6} M).

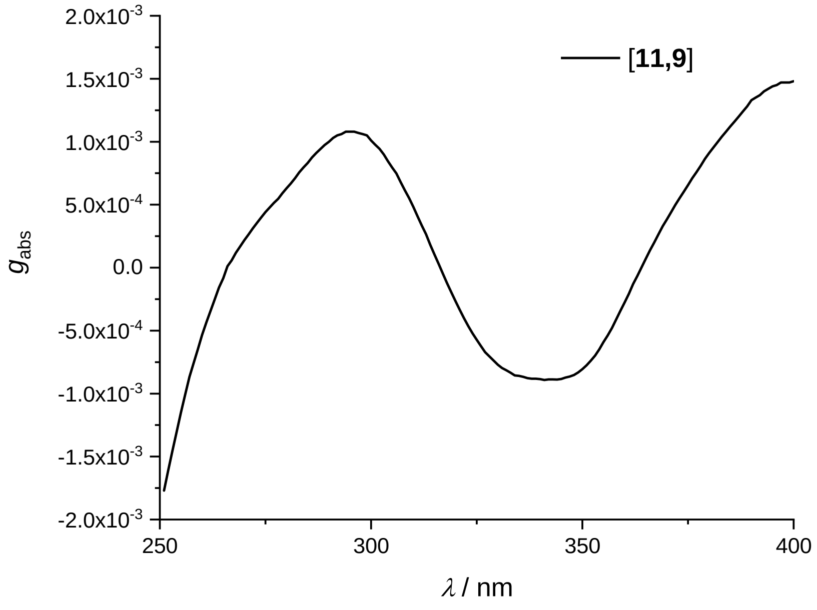

Figure S 25: Plot of g_{abs} of (*R*,*R*,*R*,*R*)-**[11,9]]** in CH_2Cl_2 (10^{-6} M).

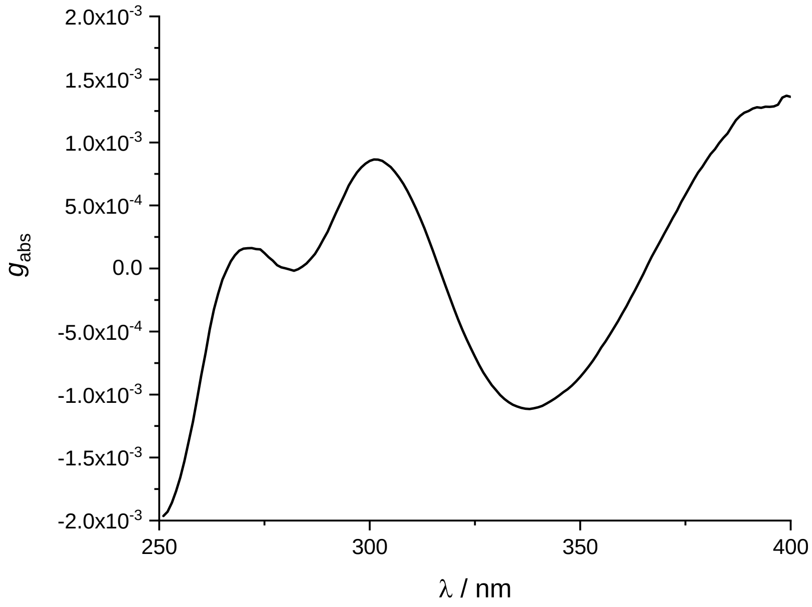

Figure S 26: Plot of g_{abs} of (*R*,*R*,*R*,*R*)-**[11,11]** in CH_2Cl_2 (10^{-6} M).

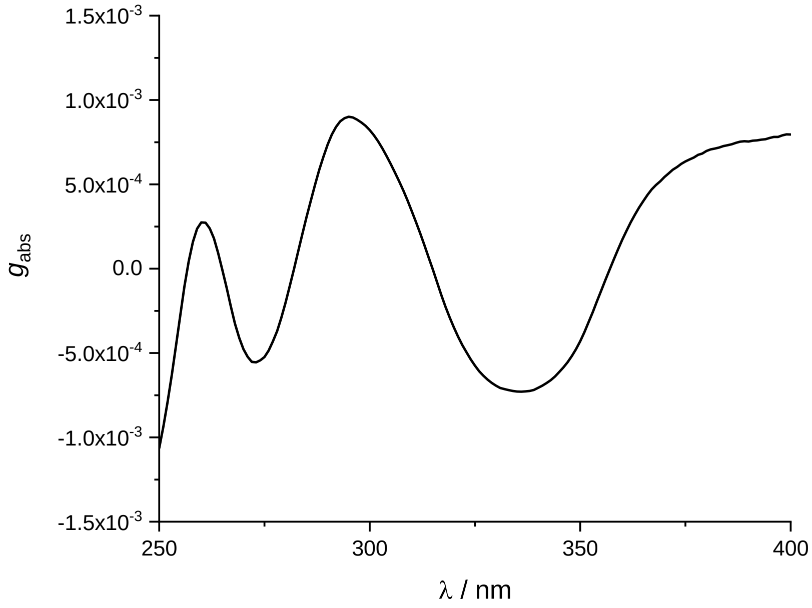

Figure S 27: g_{abs} of (*R*,*R*,*R*,*R*)-**[9,0]** in CH_2Cl_2 (10^{-6} M).

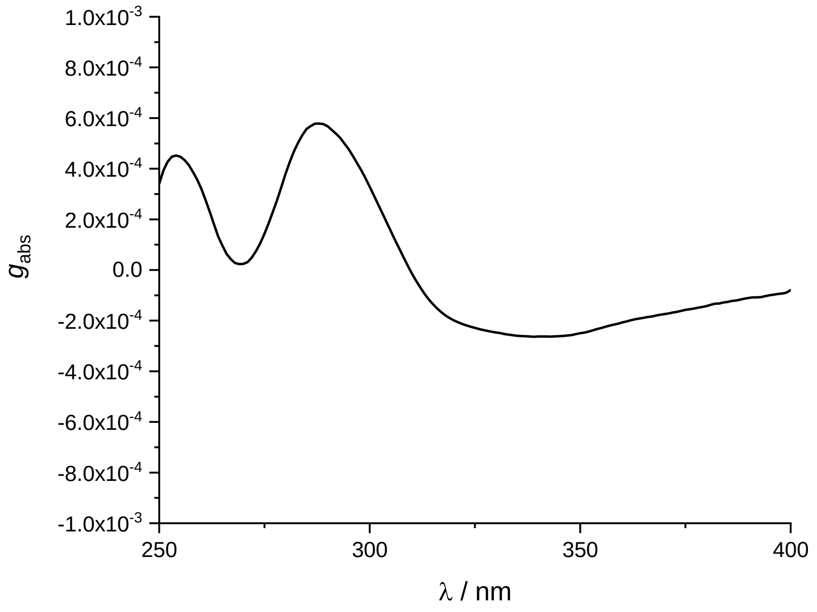

Figure S 28: g_{abs} of (*R*,*R*,*R*,*R*)-**[0,9]** in CH_2Cl_2 (10^{-6} M).

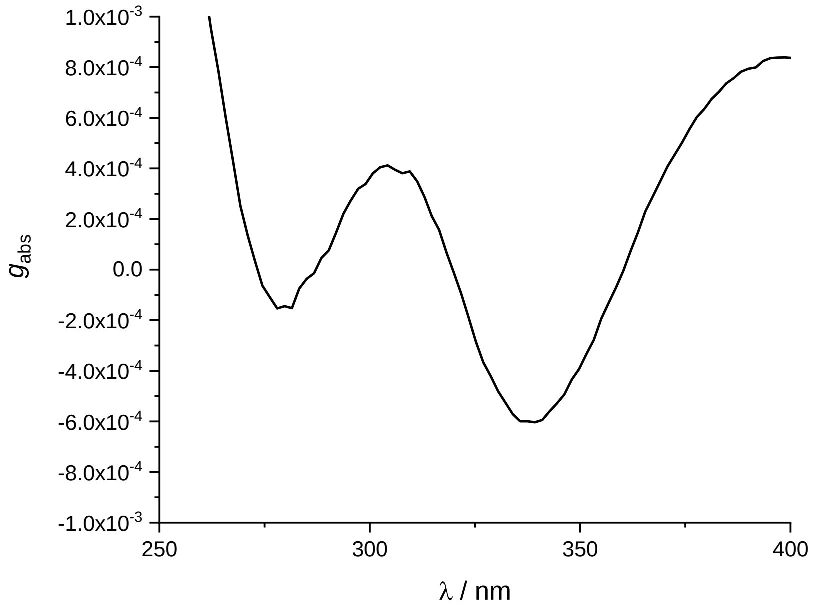

Figure S 29: g_{abs} of (*R*,*R*,*R*,*R*)-**[11,0]** in CH_2Cl_2 (10^{-6} M).

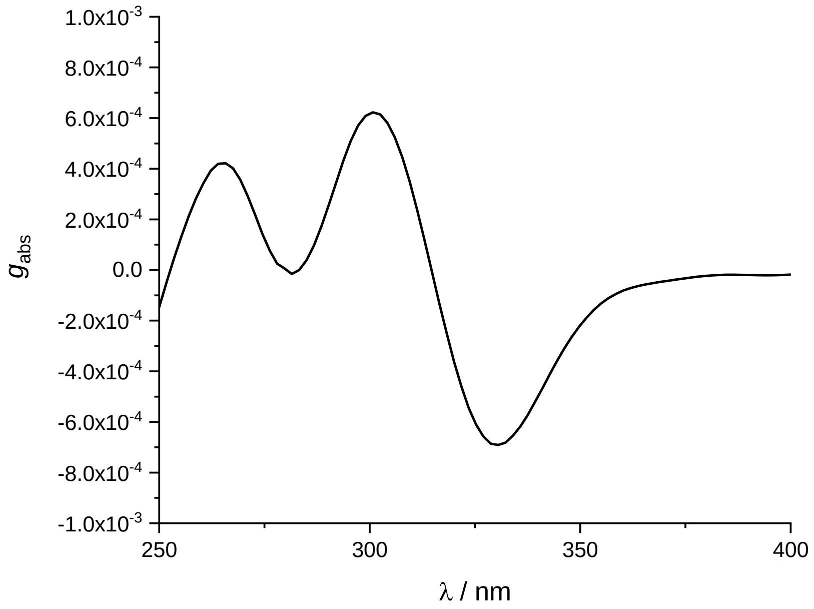

Figure S 30: g_{abs} of (*R*,*R*,*R*,*R*)-**[0,11]** in CH_2Cl_2 (10^{-6} M).

7 Circularly Polarized Luminescence Measurements

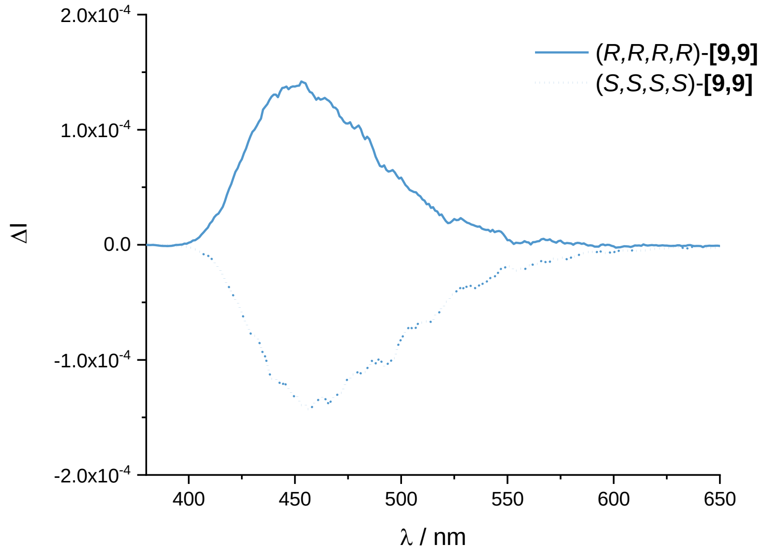

Figure S 31: CPL spectra of (*R*,*R*,*R*,*R*)- (solid) and (*S*,*S*,*S*,*S*)- (dashed) **[9,9]** in CH_2Cl_2. Concentration 10^{-5} M. $\lambda_{exc} = 320$ nm.

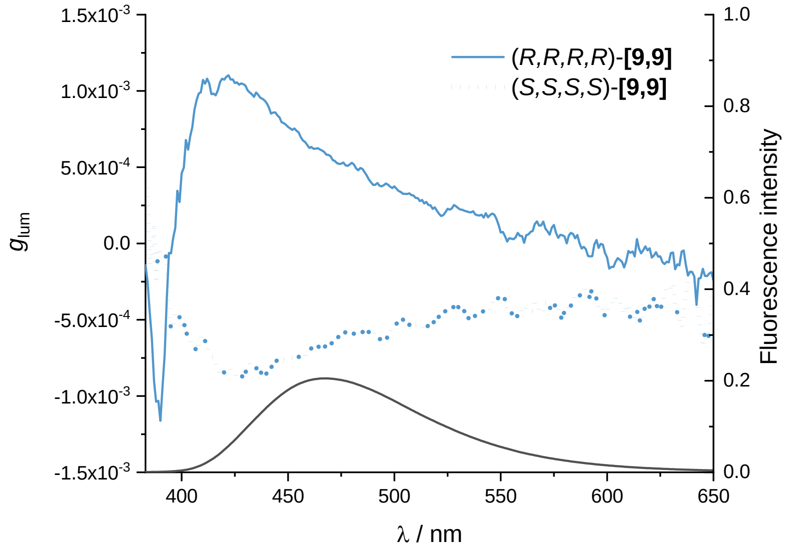

Figure S 32: Fluorescence spectra (black) and luminescence dissymmetry factors of (*R*,*R*,*R*,*R*)-(solid)- and (*S*,*S*,*S*,*S*)-(dashed)- **[9,9]** in CH_2Cl_2. Concentration 10^{-5} M. $\lambda_{exc} = 320$ nm.

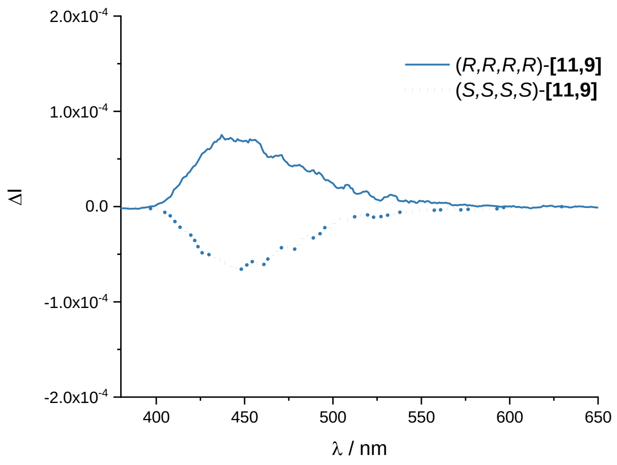

Figure S 33: CPL spectra of (R,R,R,R)-(solid) and (S,S,S,S)- (dashed)-**[11,9]** in CH_2Cl_2. Concentration 10^{-5} M. λ_{exc} = 320 nm.

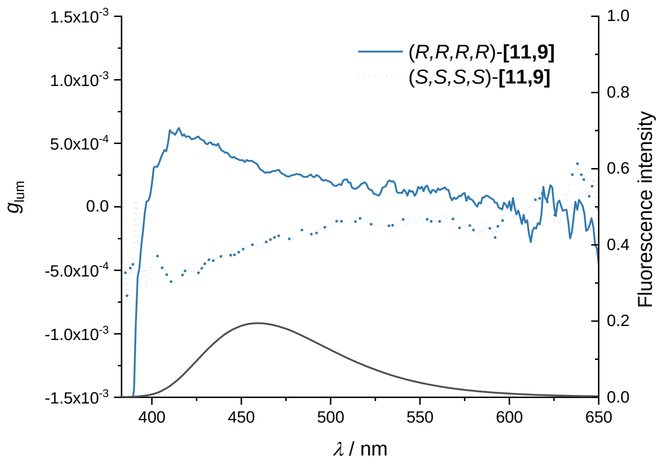

Figure S 34: Fluorescence spectra (black) and luminescence dissymmetry factors of (R,R,R,R)-(solid)- and (S,S,S,S)-(dashed)- **[11,9]** in CH_2Cl_2. Concentration 10^{-5} M. λ_{exc} = 320 nm.

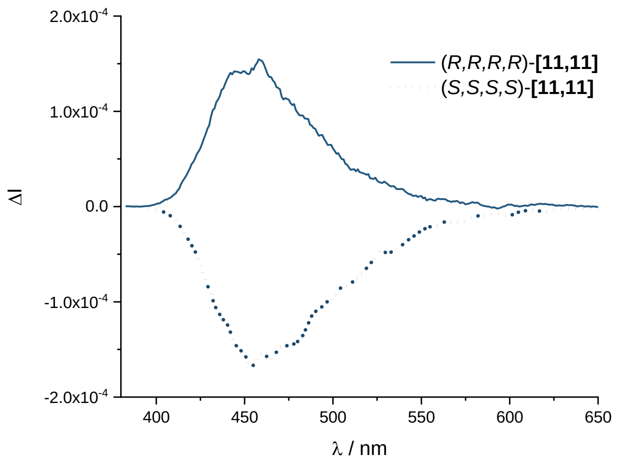

Figure S 35: CPL spectra of (*R*,*R*,*R*,*R*)-(solid)- and (*S*,*S*,*S*,*S*)-(dashed)- **[11,11]** in CH_2Cl_2. Concentration 10^{-5} M. λ_{exc} = 320 nm.

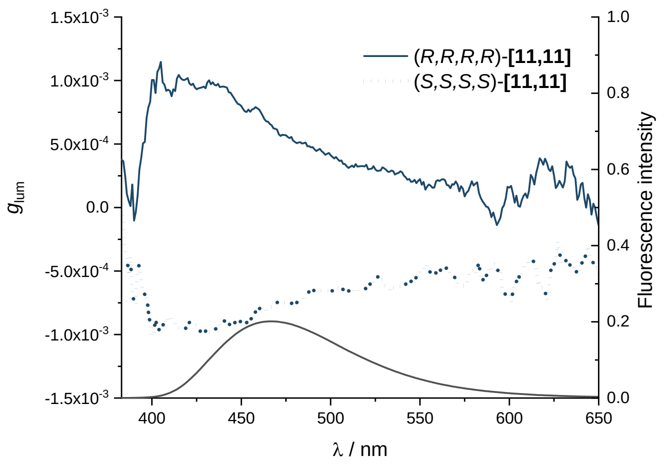

Figure S 36: Fluorescence spectra (black) and luminescence dissymmetry factors of (*R*,*R*,*R*,*R*)-(solid)- and (*S*,*S*,*S*,*S*)-(dashed)- **[11,11]** in CH_2Cl_2. Concentration 10^{-5} M. λ_{exc} = 320 nm.

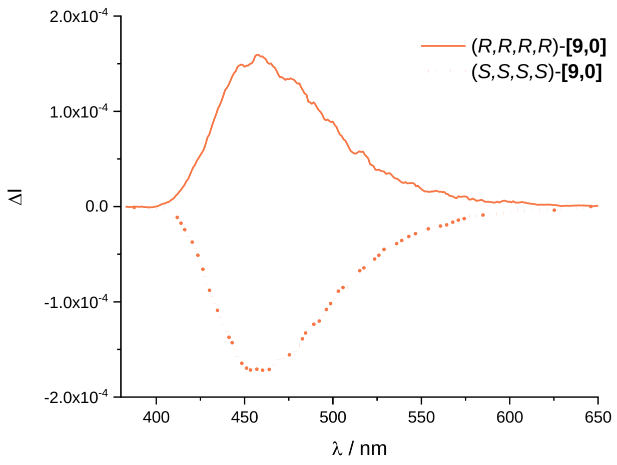

Figure S 37:CPL spectra of (*R*,*R*,*R*,*R*)-(solid)- and (*S*,*S*,*S*,*S*)-(dashed)-**[9,0]** in CH_2Cl_2. Concentration 10^{-5} M. λ_{exc} = 320 nm.

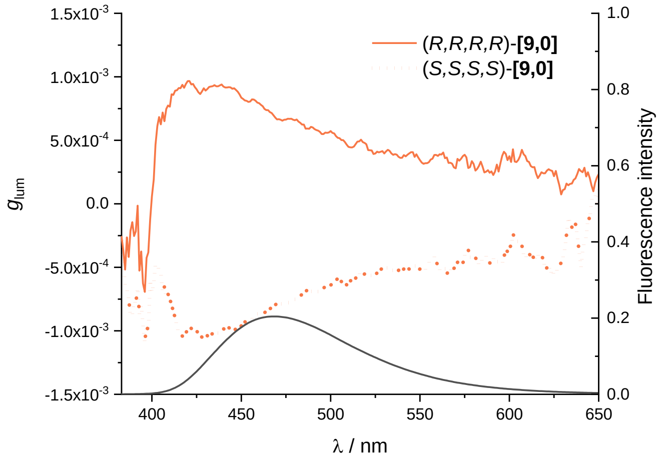

Figure S 38: Fluorescence spectra (black) and luminescence dissymmetry factors of (*R*,*R*,*R*,*R*)-(solid)- and (*S*,*S*,*S*,*S*)-(dashed)- **[9,0]** in CH_2Cl_2. Concentration 10^{-5} M. λ_{exc} = 320 nm.

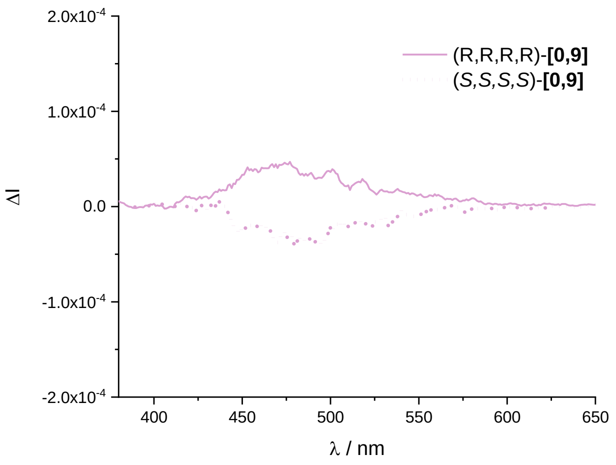

Figure S 39: CPL spectra of (*R*,*R*,*R*,*R*)-(solid)- and (*S*,*S*,*S*,*S*)-(dashed)- **[0,9]** in CH_2Cl_2. Concentration: 10^{-5} M. $\lambda_{exc} = 320$ nm.

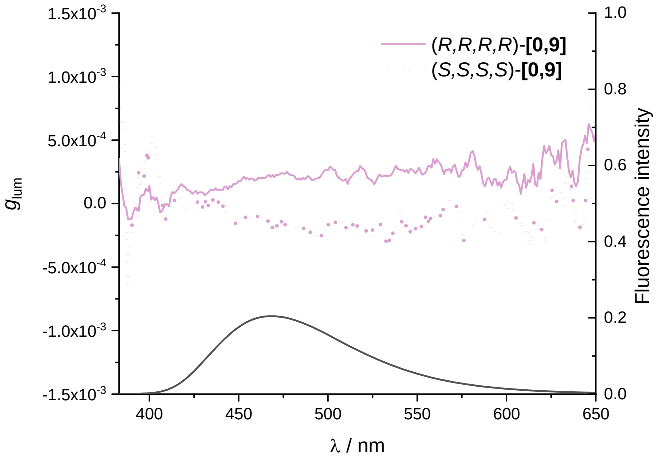

Figure S 40: Fluorescence spectra (black) and luminescence dissymmetry factors of (*R*,*R*,*R*,*R*)-(solid)- and (*S*,*S*,*S*,*S*)-(dashed)- **[0,9]** in CH_2Cl_2. Concentration: 10^{-5} M. $\lambda_{exc} = 320$ nm.

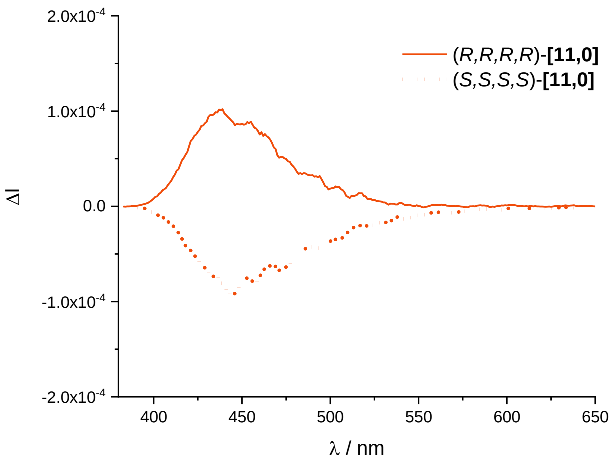

Figure S 41: CPL spectra of (*R*,*R*,*R*,*R*)-(solid)- and (*S*,*S*,*S*,*S*)-(dashed)- **[11,0]** in CH_2Cl_2. Concentration 10^{-5} M. $\lambda_{exc} = 320$ nm.

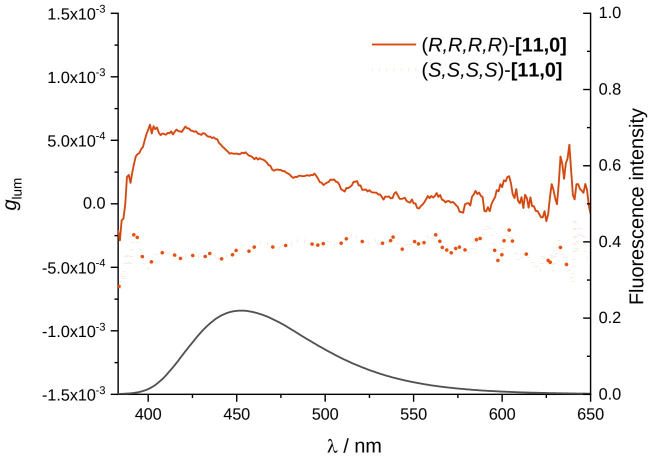

Figure S 42: Fluorescence spectra (black) and luminescence dissymmetry factors of (*R*,*R*,*R*,*R*)-(solid)- and (*S*,*S*,*S*,*S*)-(dashed)- **[11,0]** in CH_2Cl_2. Concentration 10^{-5} M. $\lambda_{exc} = 320$ nm.

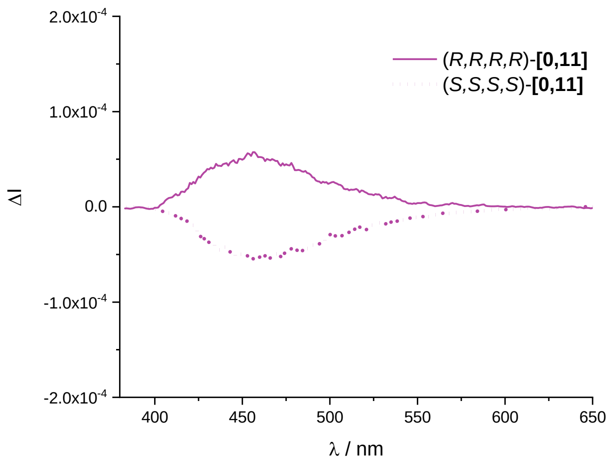

Figure S 43: CPL spectra of (*R*,*R*,*R*,*R*)-(solid)- and (*S*,*S*,*S*,*S*)-(dashed)- **[0,11]** in CH_2Cl_2. Concentration 10^{-5} M. λ_{exc} = 330 nm.

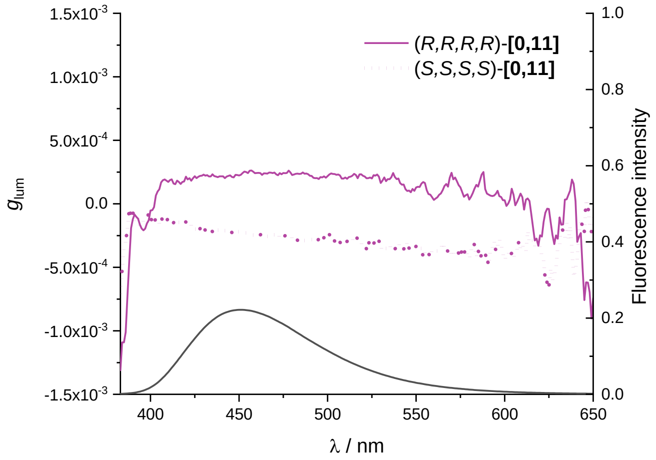

Figure S 44: Fluorescence spectra (black) and luminescence dissymmetry factors of (*R*,*R*,*R*,*R*)-(solid)- and (*S*,*S*,*S*,*S*)-(dashed)- **[0,11]** in CH_2Cl_2. Concentration 10^{-5} M. λ_{exc} = 330 nm.

8 NMR Spectra

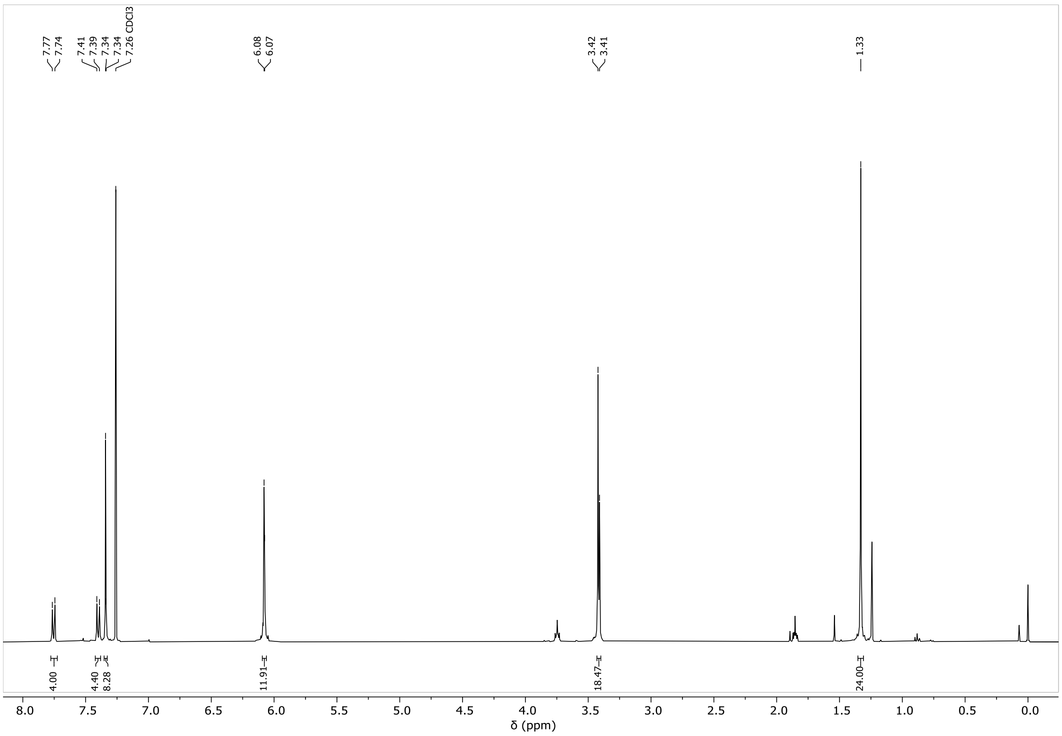

Figure S 45: ^{1}H NMR spectrum of **3** (500 MHz, $CDCl_3$).

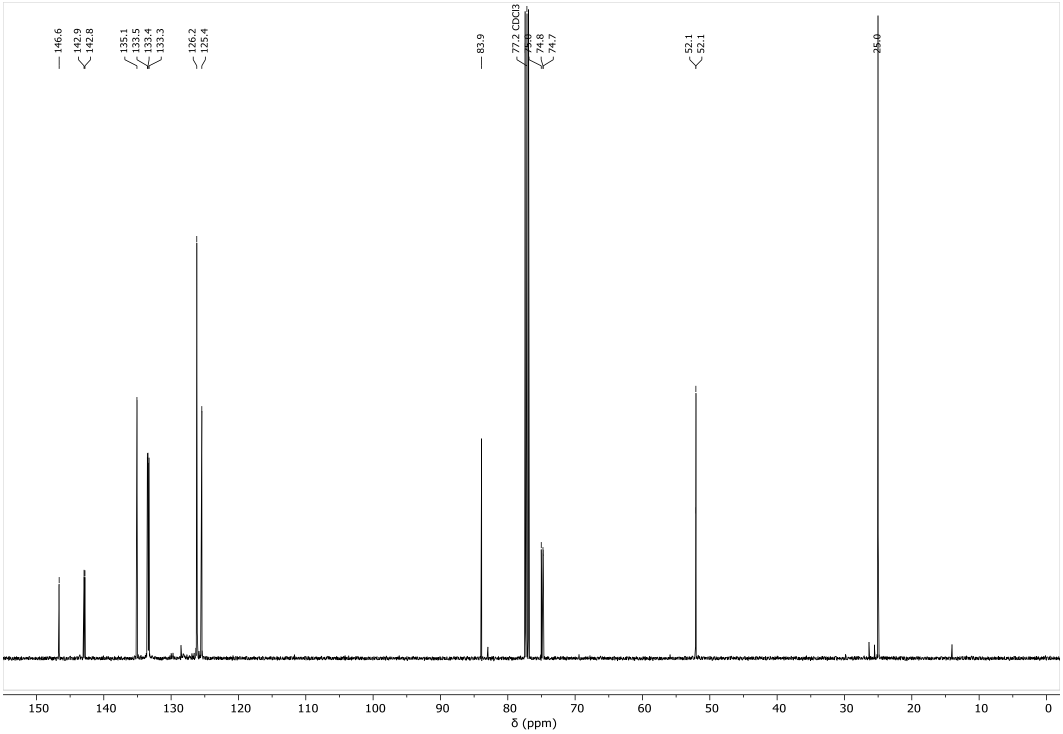

Figure S 46: ^{13}C NMR spectrum of **3** (126 MHz, $CDCl_3$).

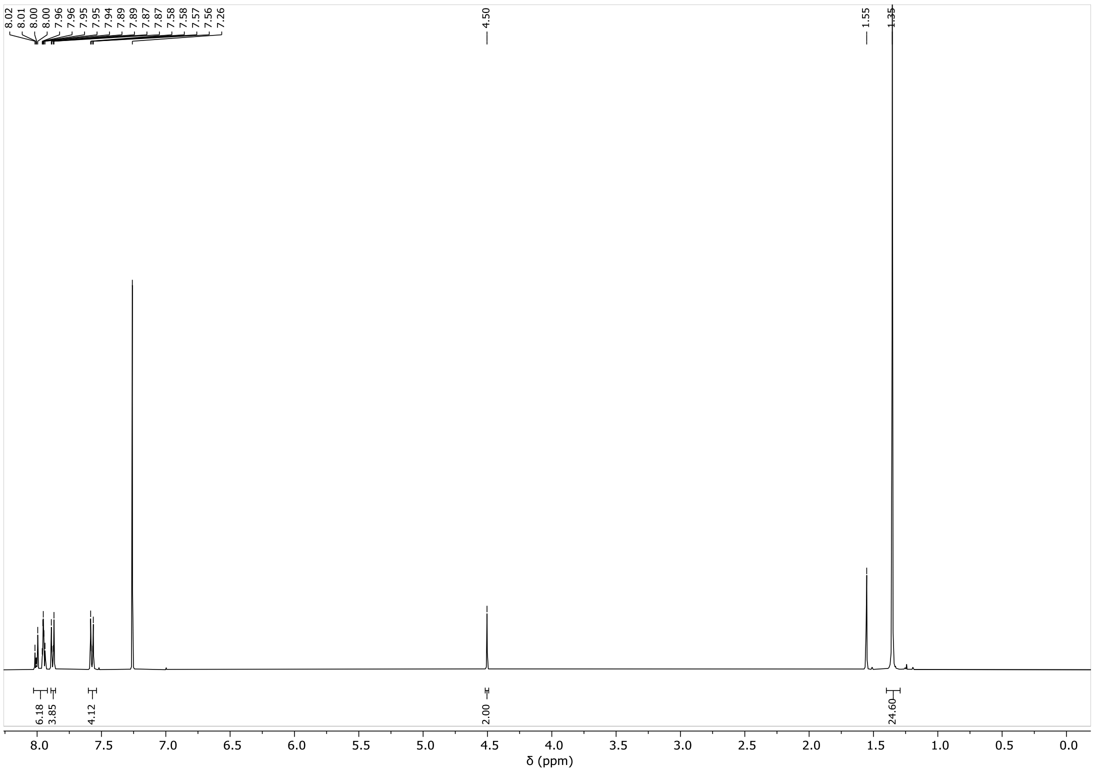

Figure S 47: ^{1}H NMR spectrum of **9** (400 MHz, $CDCl_3$).

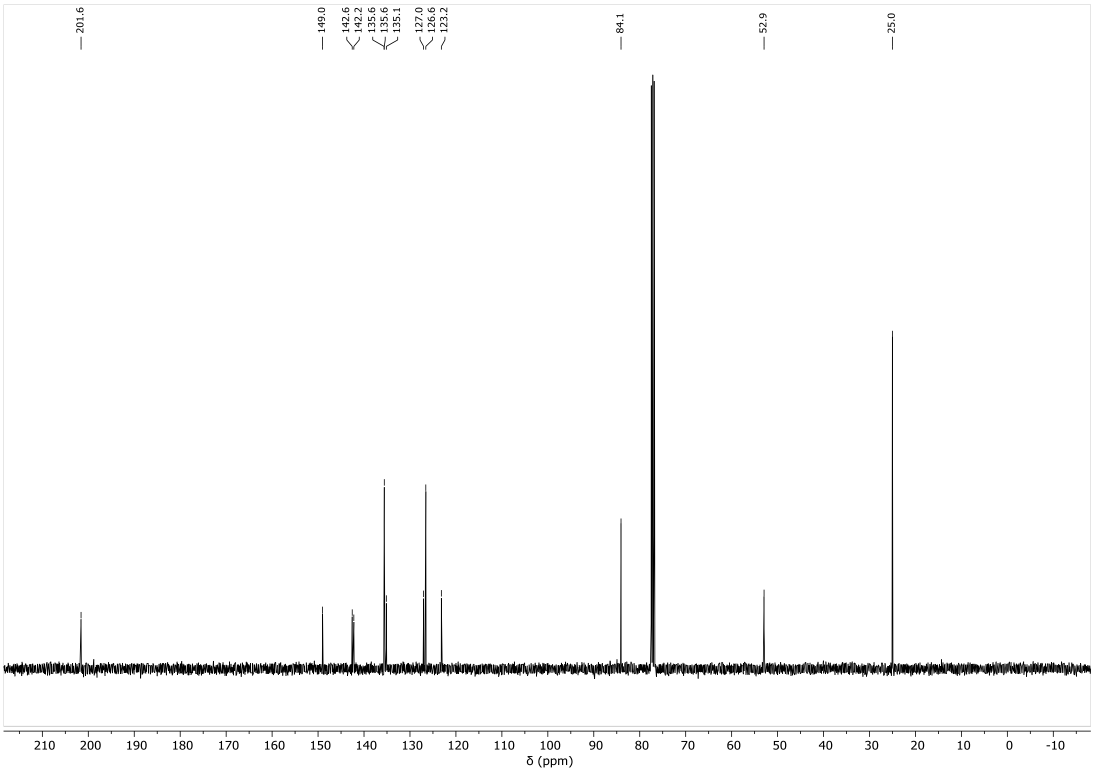

Figure S 48: ^{13}C NMR spectrum of **9** (100 MHz, $CDCl_3$).

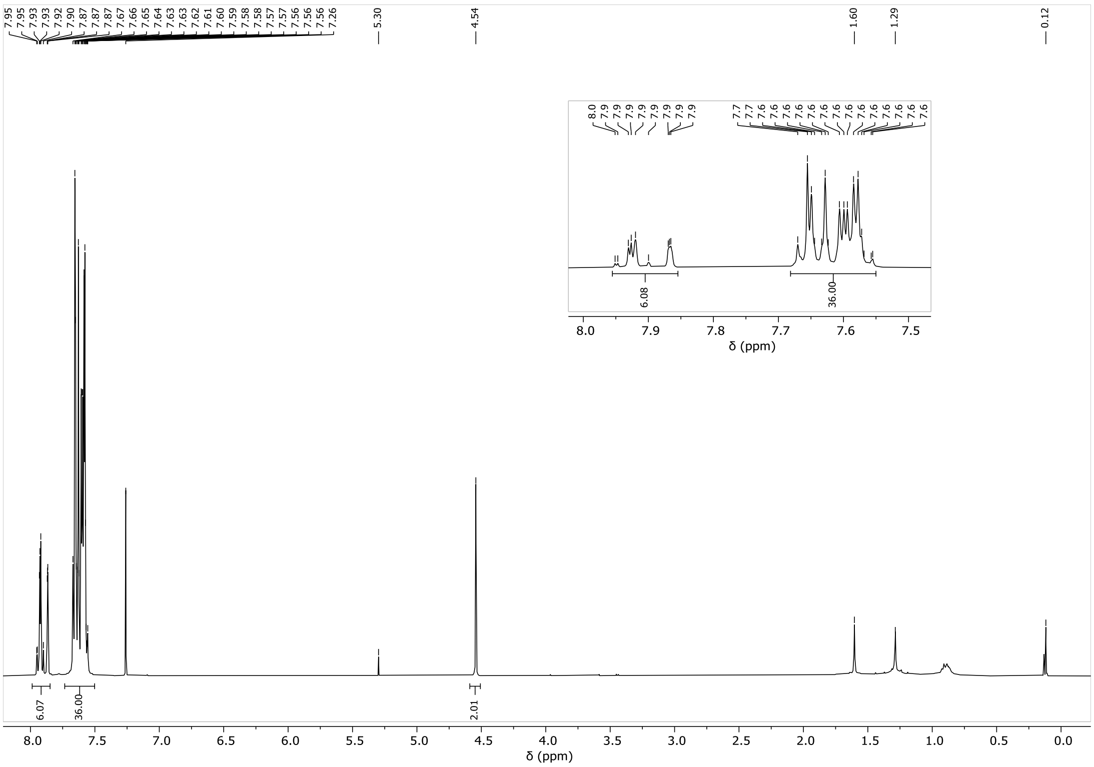

Figure S 49: ^{1}H NMR spectrum of **2** (400 MHz, $CDCl_3$).

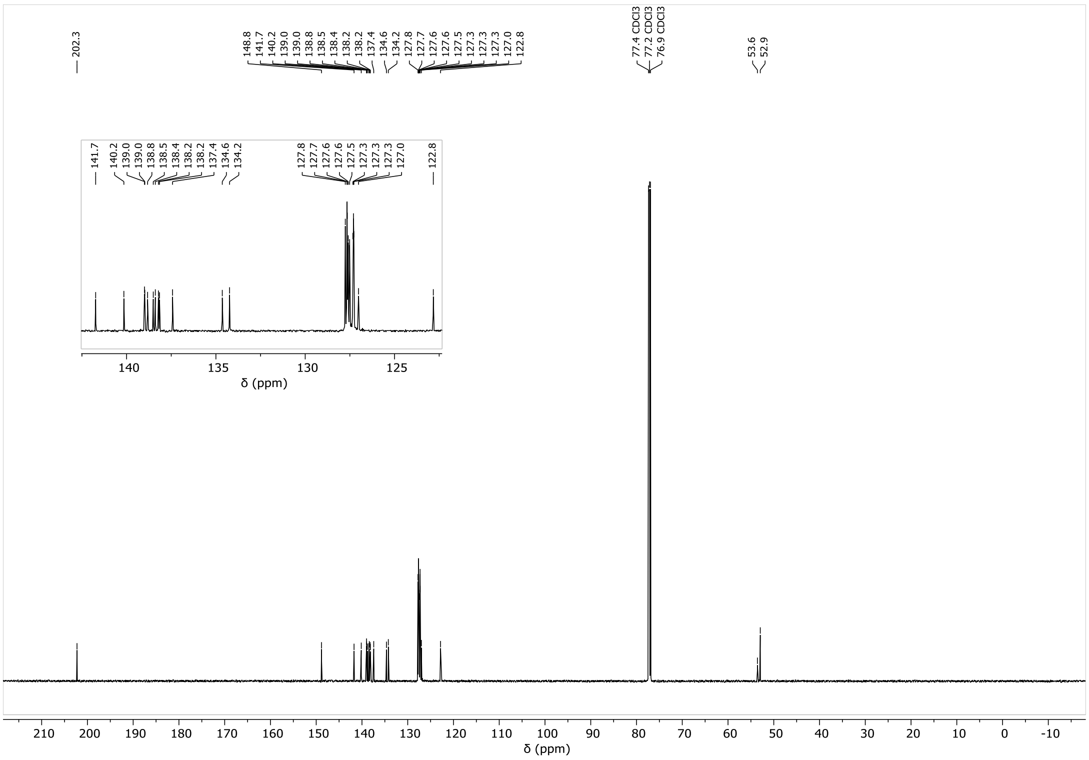

Figure S 50: ^{13}C NMR spectrum of **2** (100 MHz, $CDCl_3$).

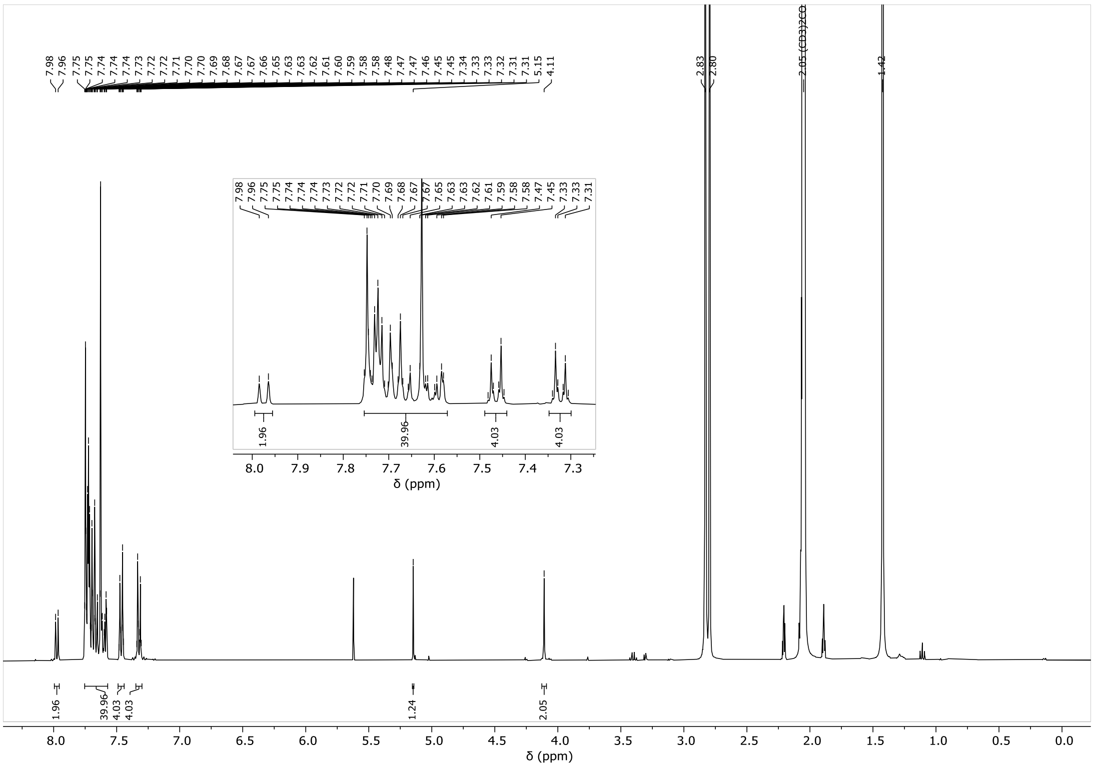

Figure S 51: ^{1}H NMR spectrum of **6** (500 MHz, acetone-*d*).

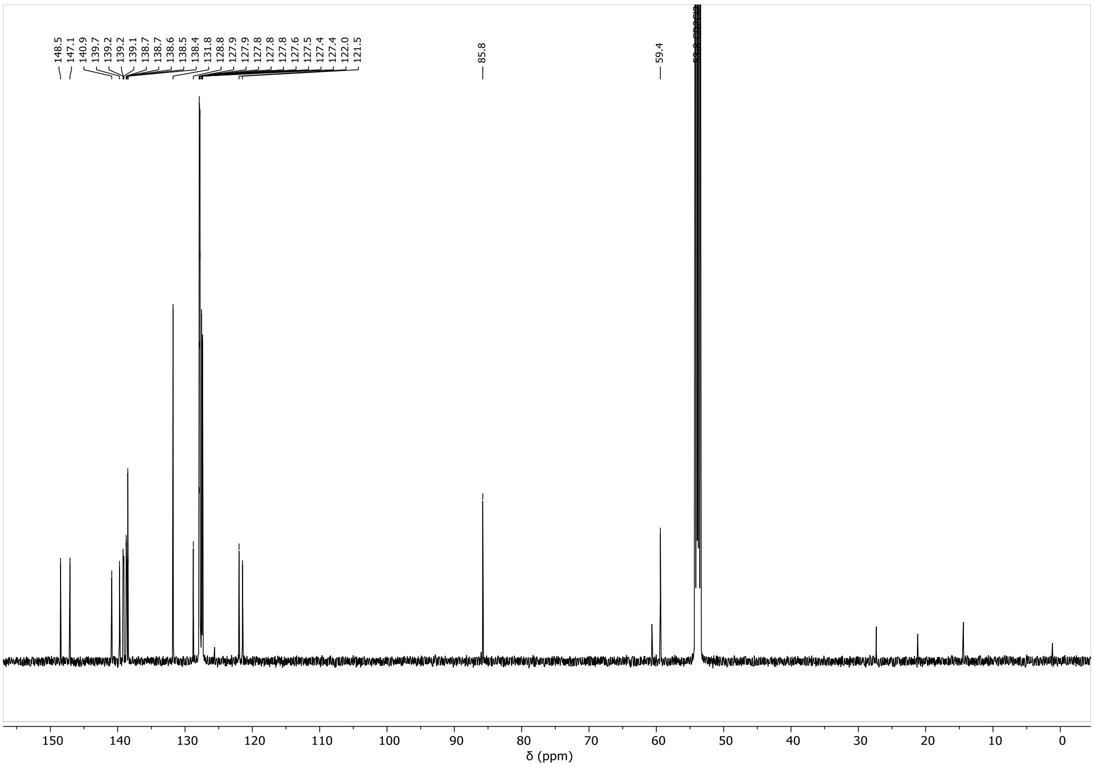

Figure S 52: ^{13}C NMR spectrum of **6** (100 MHz, CD_2Cl_2).

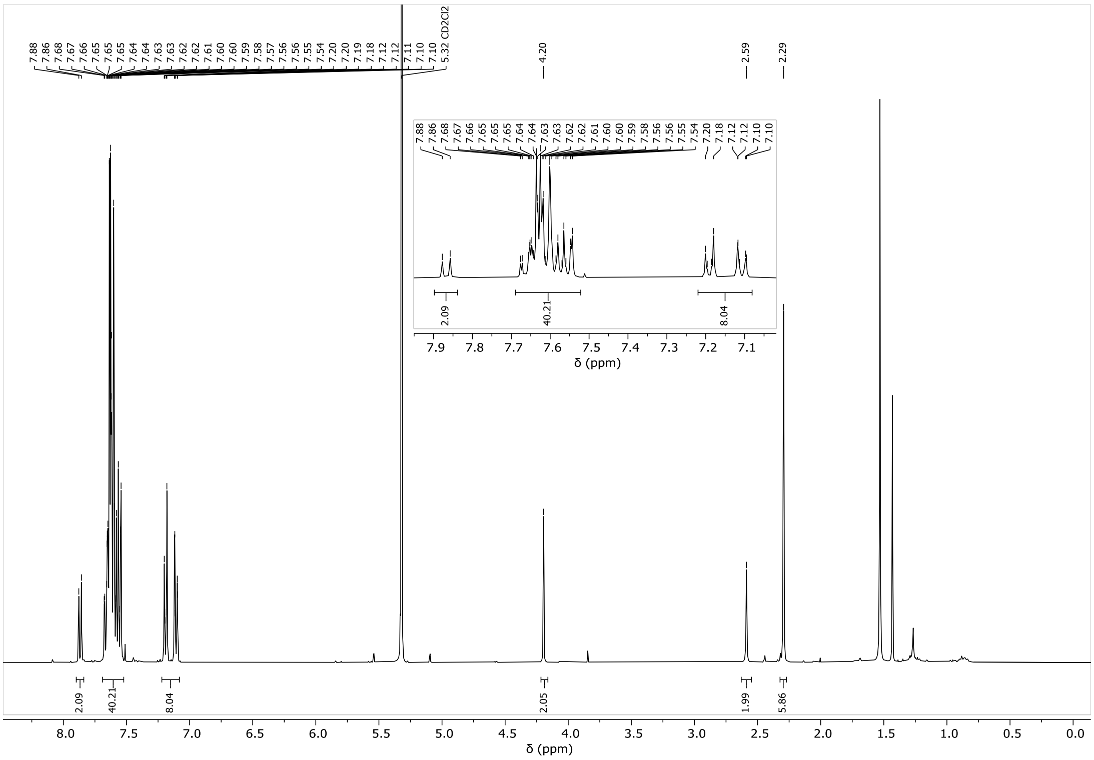

Figure S 53: ^{1}H NMR spectrum of **[11,0]** (400 MHz, CD_2Cl_2).

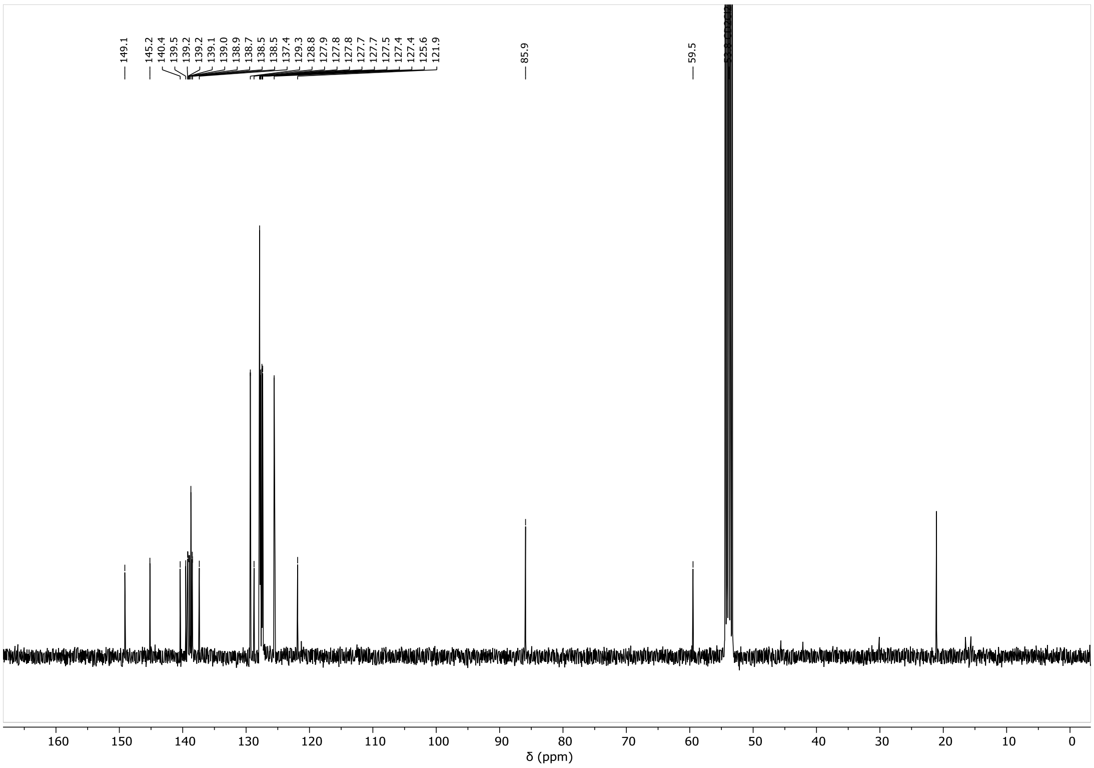

Figure S 54: ^{13}C NMR spectrum of **[11,0]** (100 MHz, CD_2Cl_2).

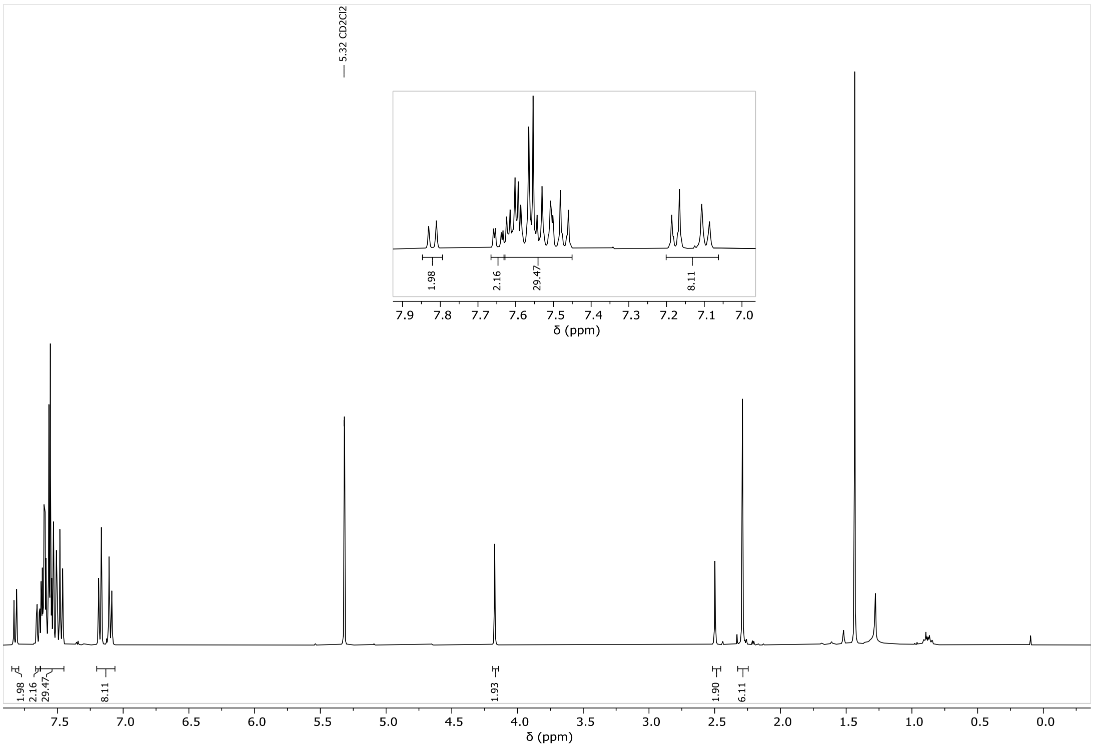

Figure S 55: ^{1}H NMR spectrum of **[9,0]** (400 MHz, CD_2Cl_2).

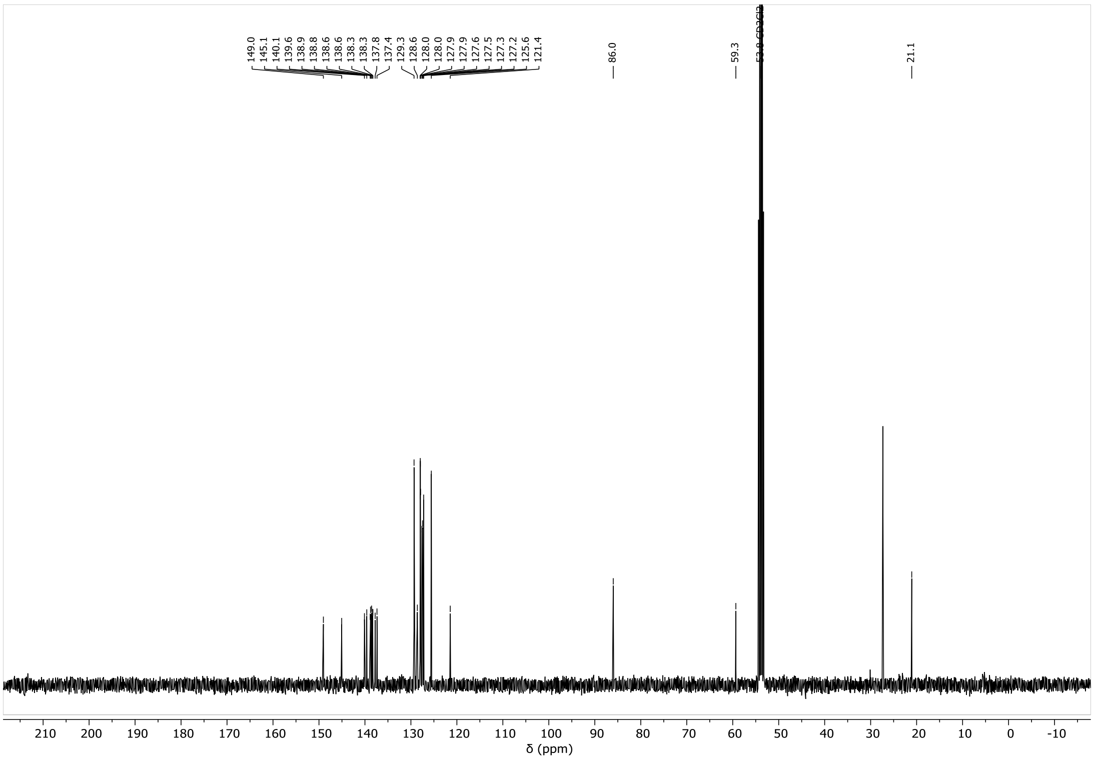

Figure S 56: ^{13}C NMR spectrum of **[9,0]** (100 MHz, CD_2Cl_2).

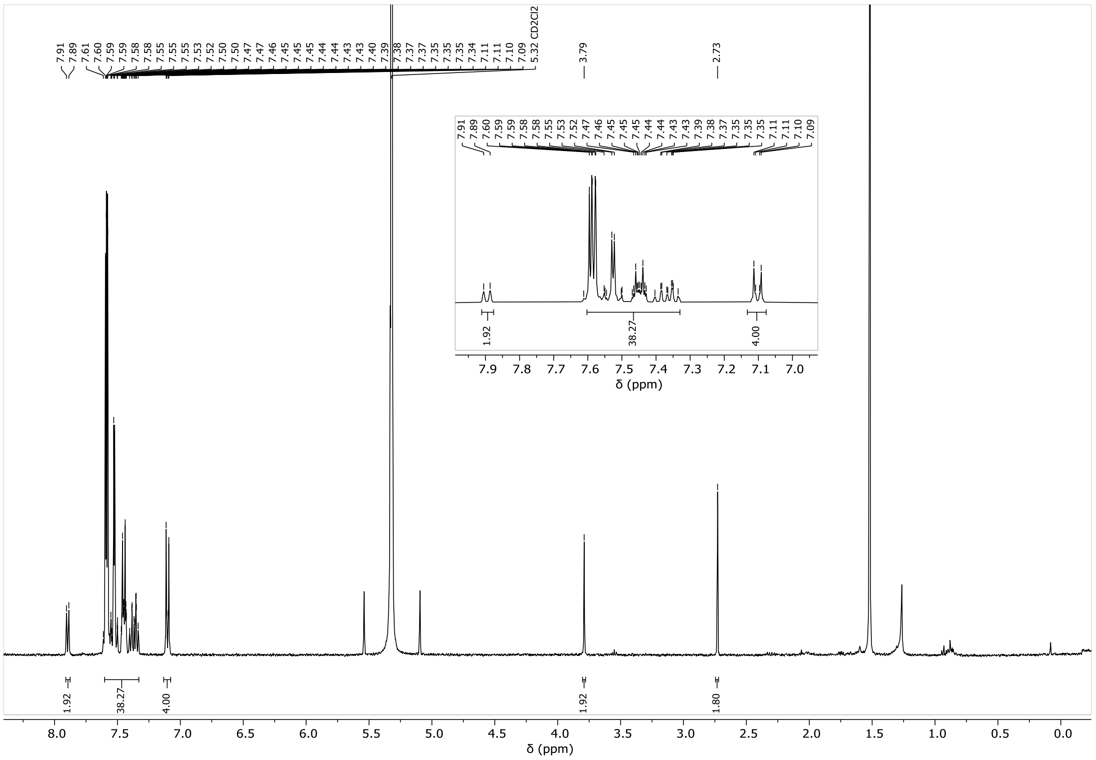

Figure S 57: ^{1}H NMR spectrum of **[0,9]** (400 MHz, CD_2Cl_2).

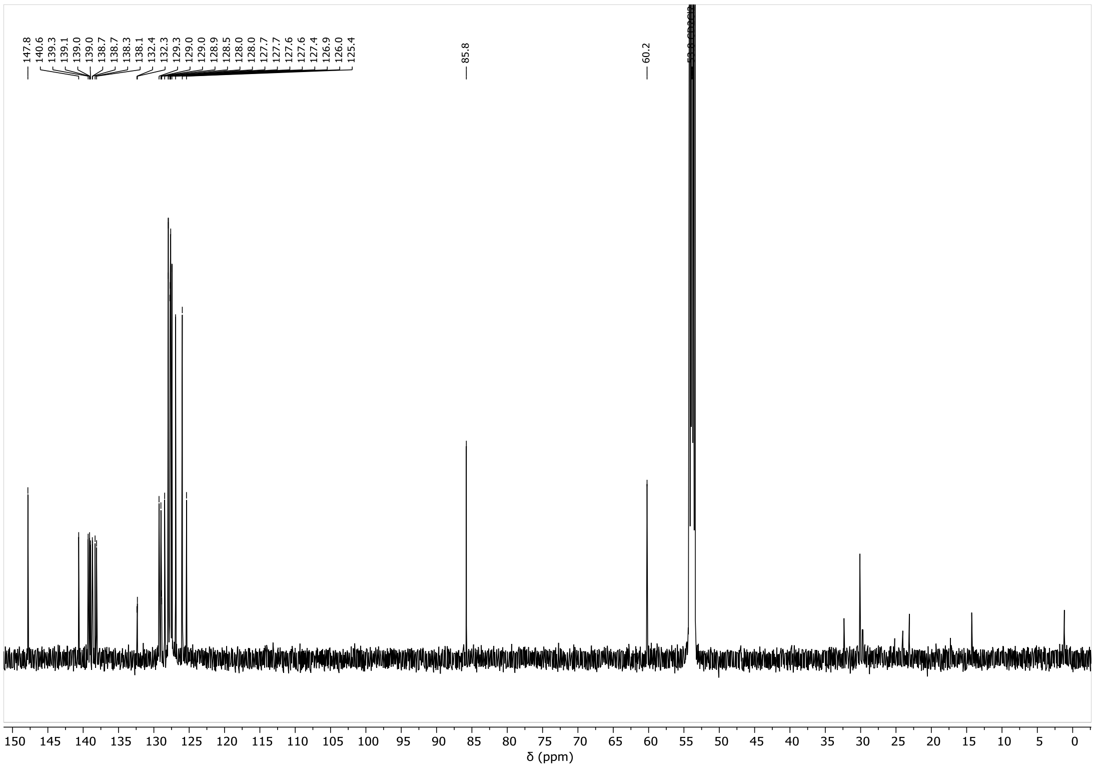

Figure S 58: ^{13}C NMR spectrum of **[0,9]** (151 MHz, CD_2Cl_2).

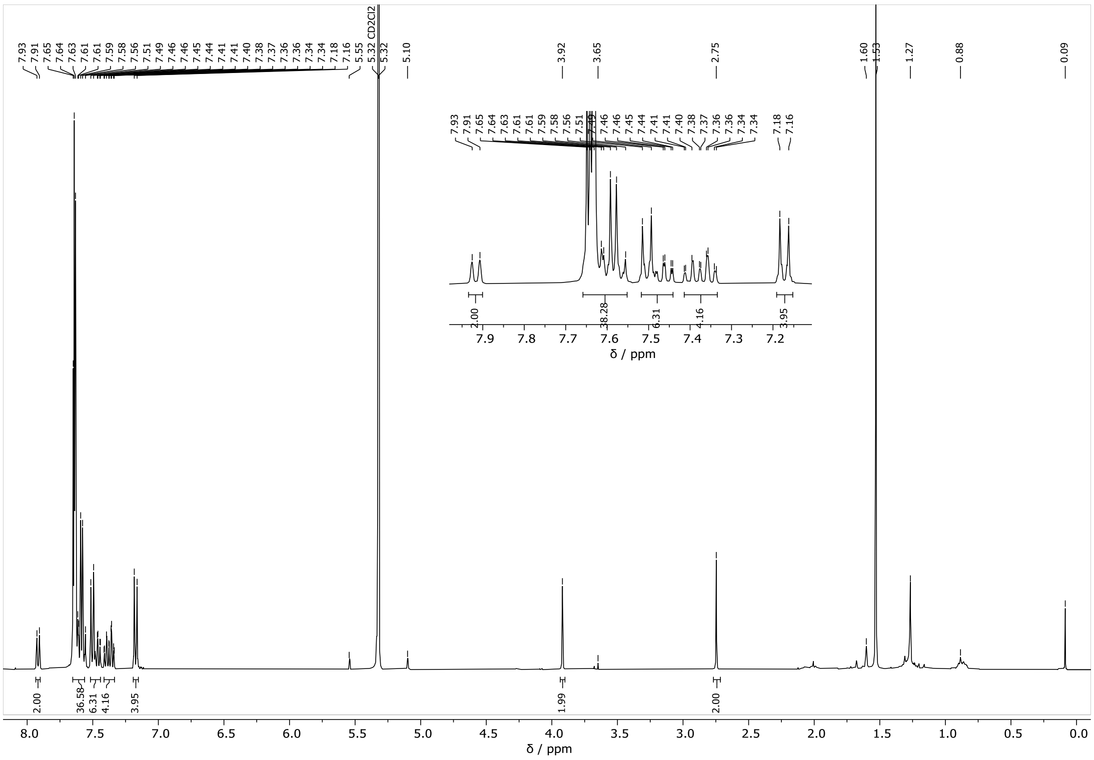

Figure S 59: ^{1}H NMR spectrum of **[0,11]** (400 MHz, CD_2Cl_2).

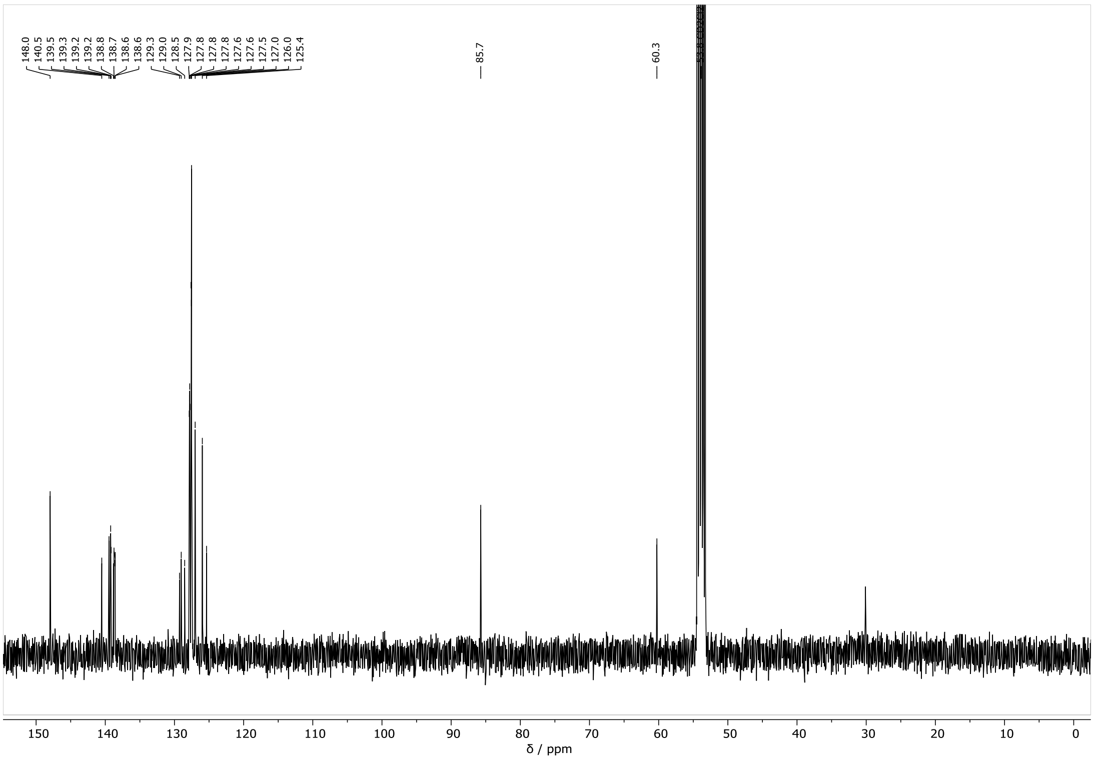

Figure S 60: ^{13}C NMR spectrum of **[0,11]** (151 MHz, CD_2Cl_2).

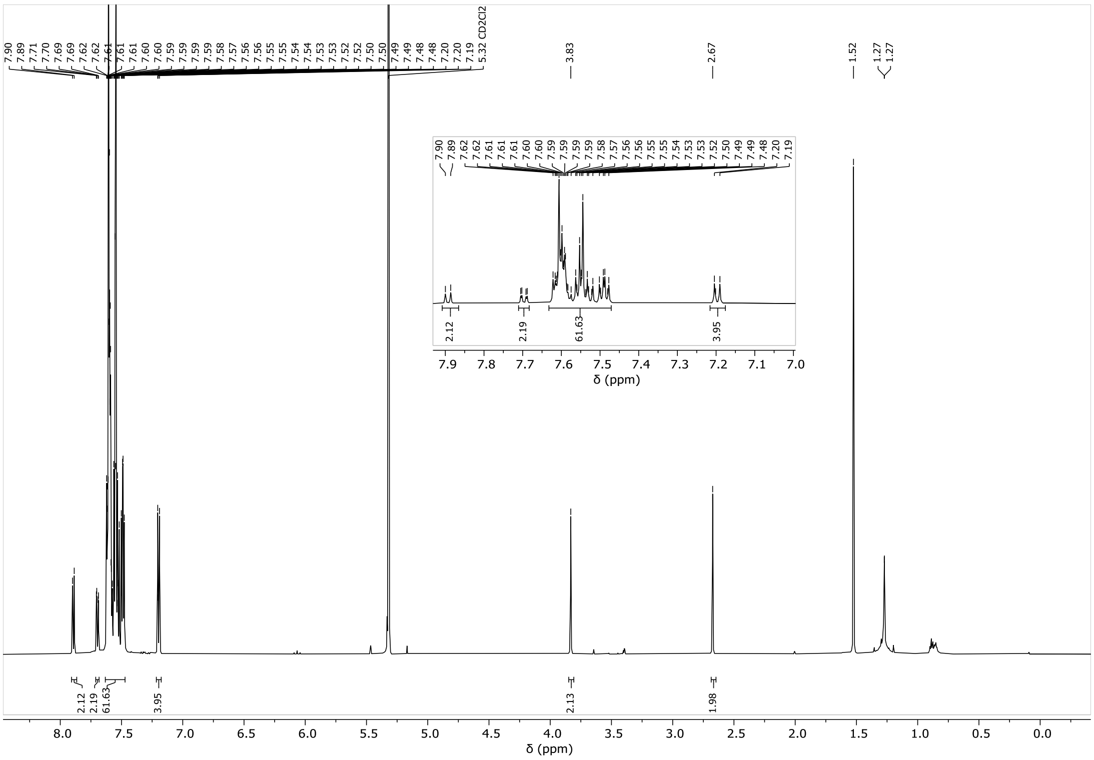

Figure S 61: ^{1}H NMR spectrum of **[9,9]** (600 MHz, CD_2Cl_2).

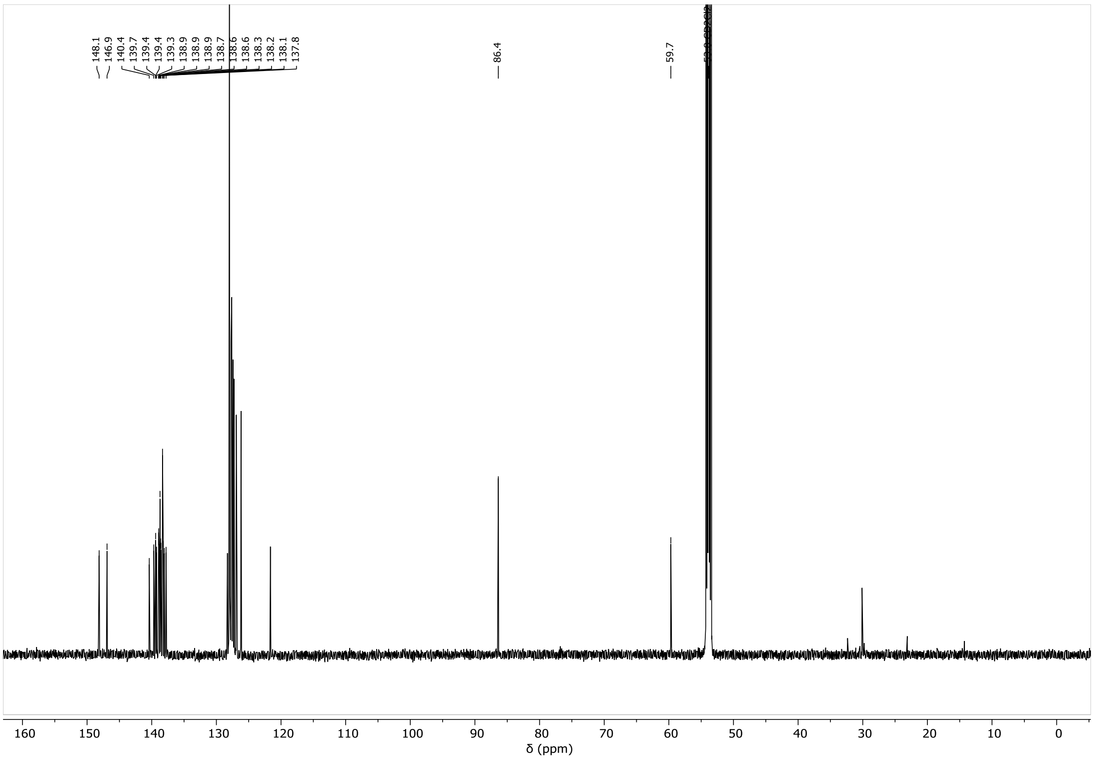

Figure S 62: ^{13}C NMR spectrum of **[9,9]** (151 MHz, CD_2Cl_2).

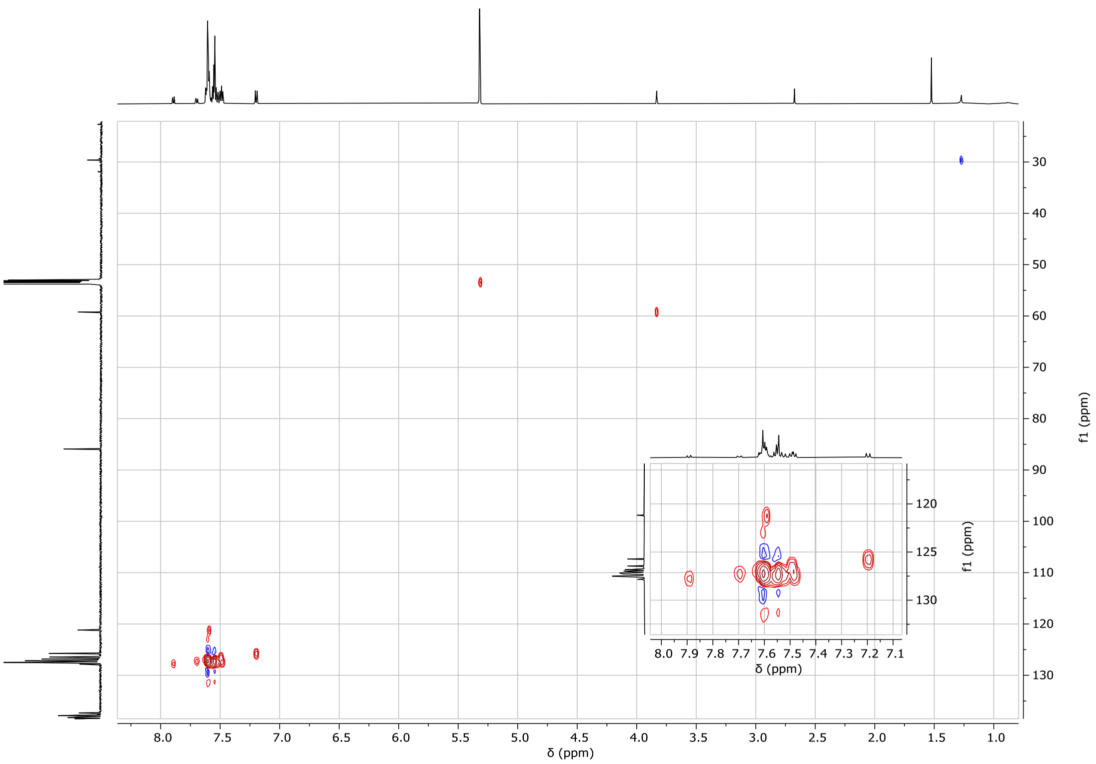

Figure S 63: Edited HSQC of **[9,9]** (600 MHz, 151 MHz, CD_2Cl_2).

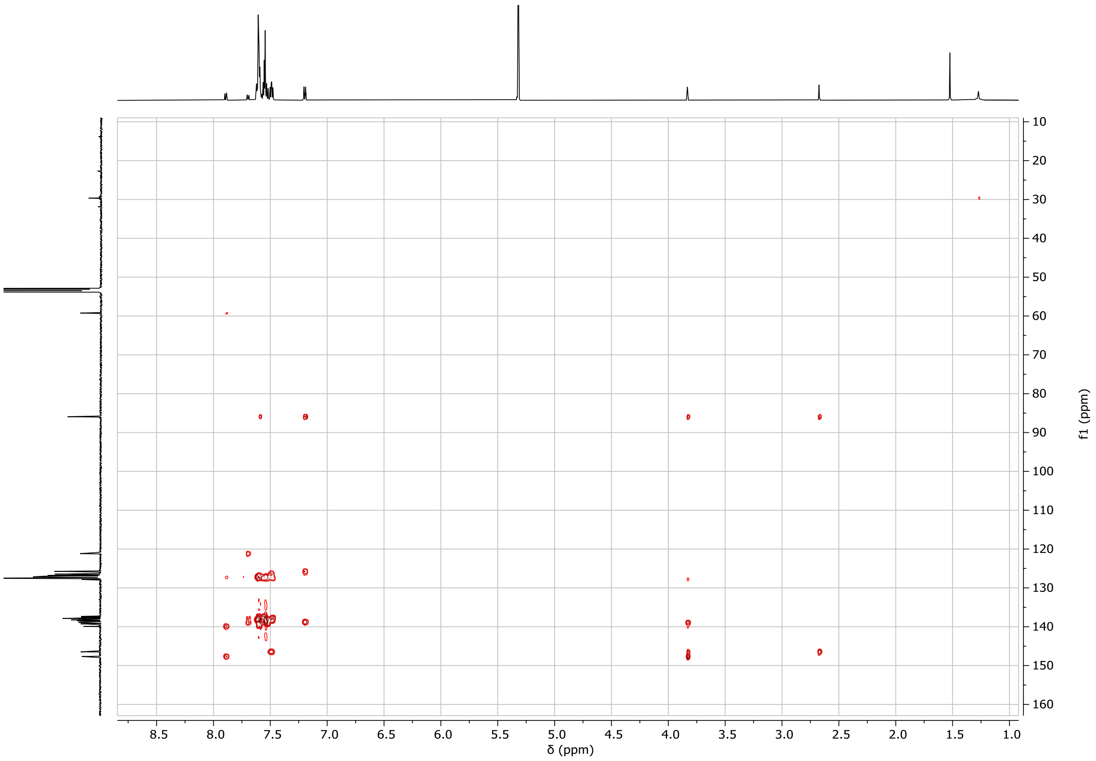

Figure S 64: HMBC spectrum of **[9,9]** (600 MHz, 151 MHz, CD_2Cl_2).

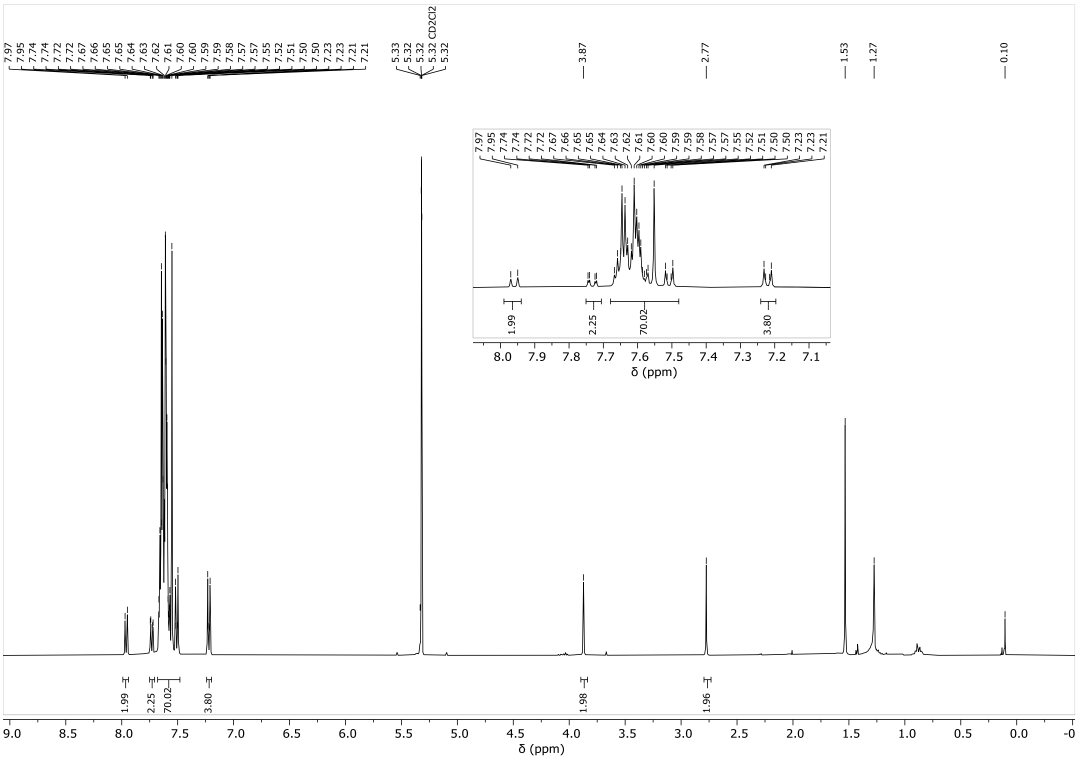

Figure S 65: ^{1}H NMR spectrum of **[11,9]** (600 MHz, CD_2Cl_2).

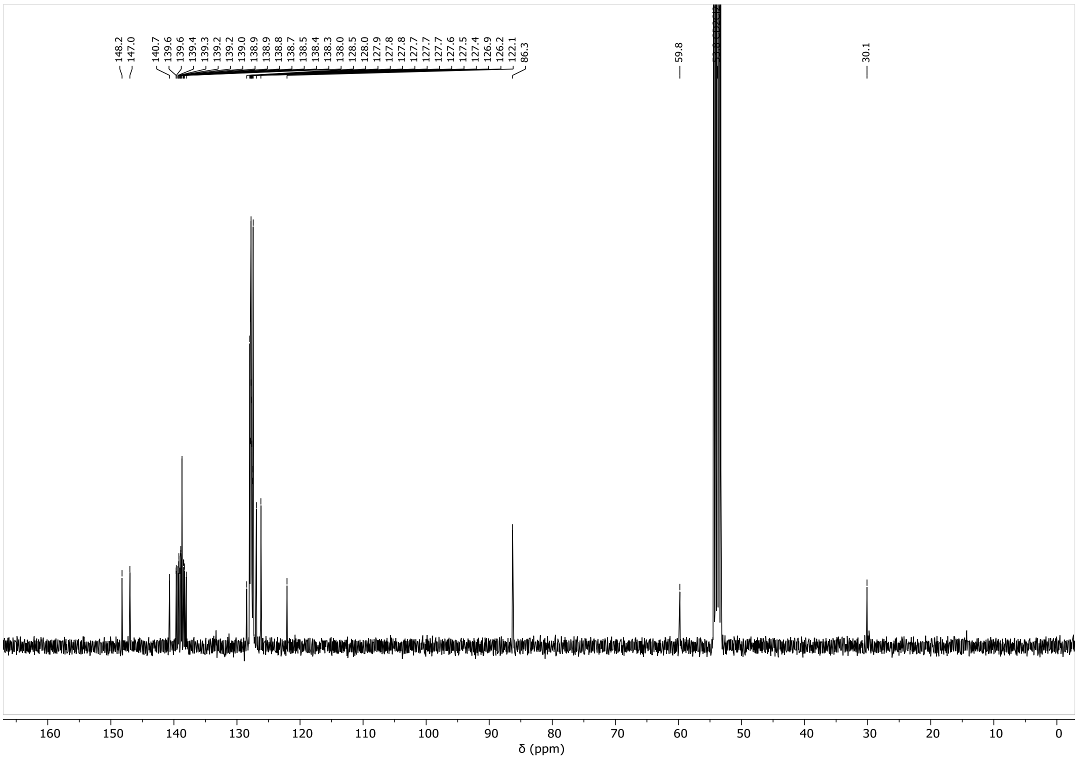

Figure S 66: ^{13}C NMR spectrum of **[11,9]** (151 MHz, CD_2Cl_2).

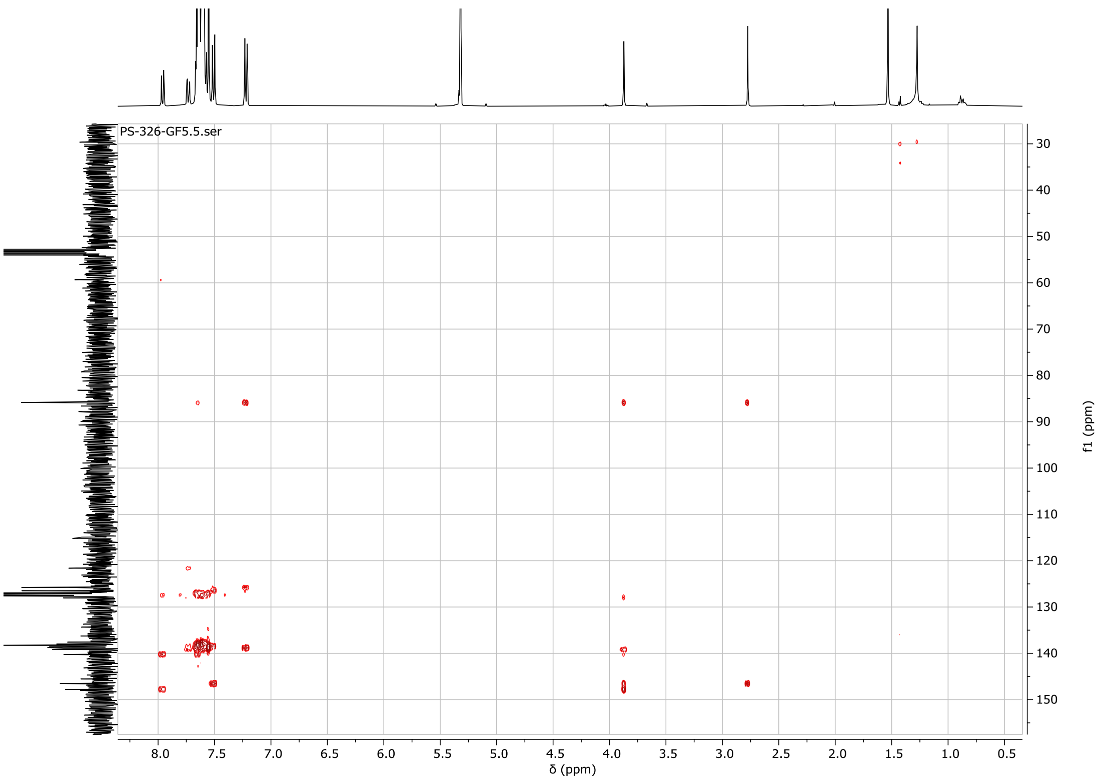

Figure S 67: HMBC spectrum of **[11,9]** (600 MHz, 151 MHz, CD_2Cl_2).

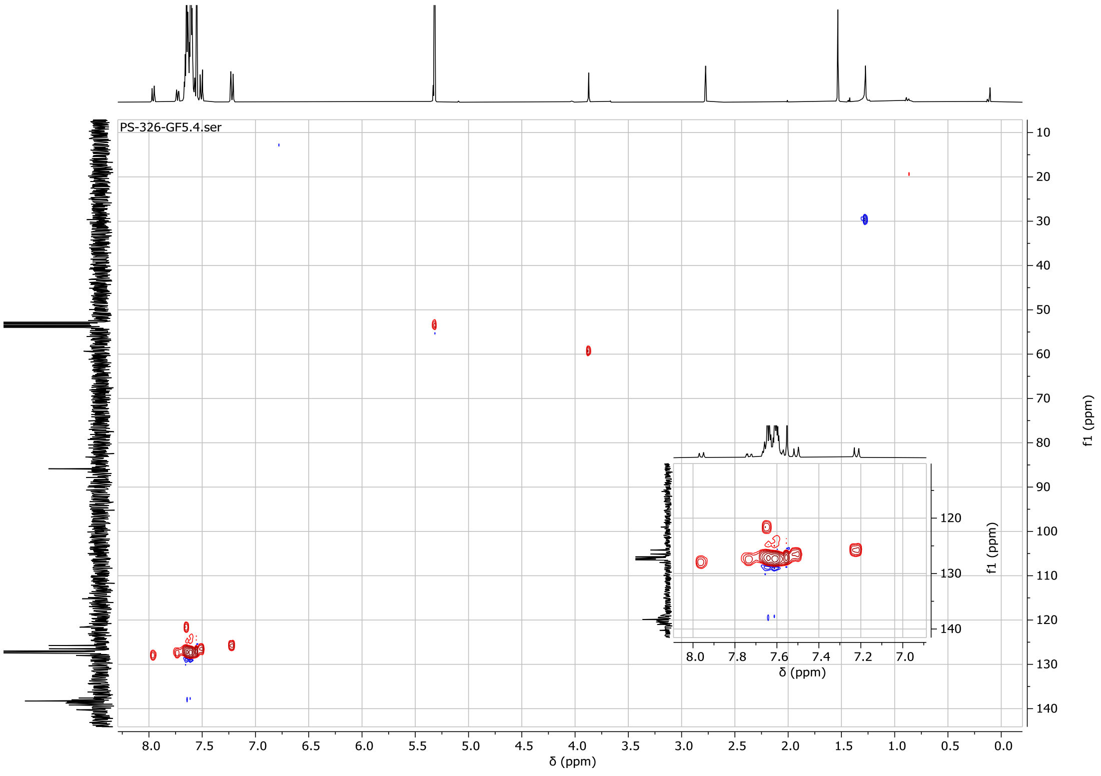

Figure S 68: edited HSQC spectrum of **[11,9]** (600 MHz, 151 MHz, CD_2Cl_2)..

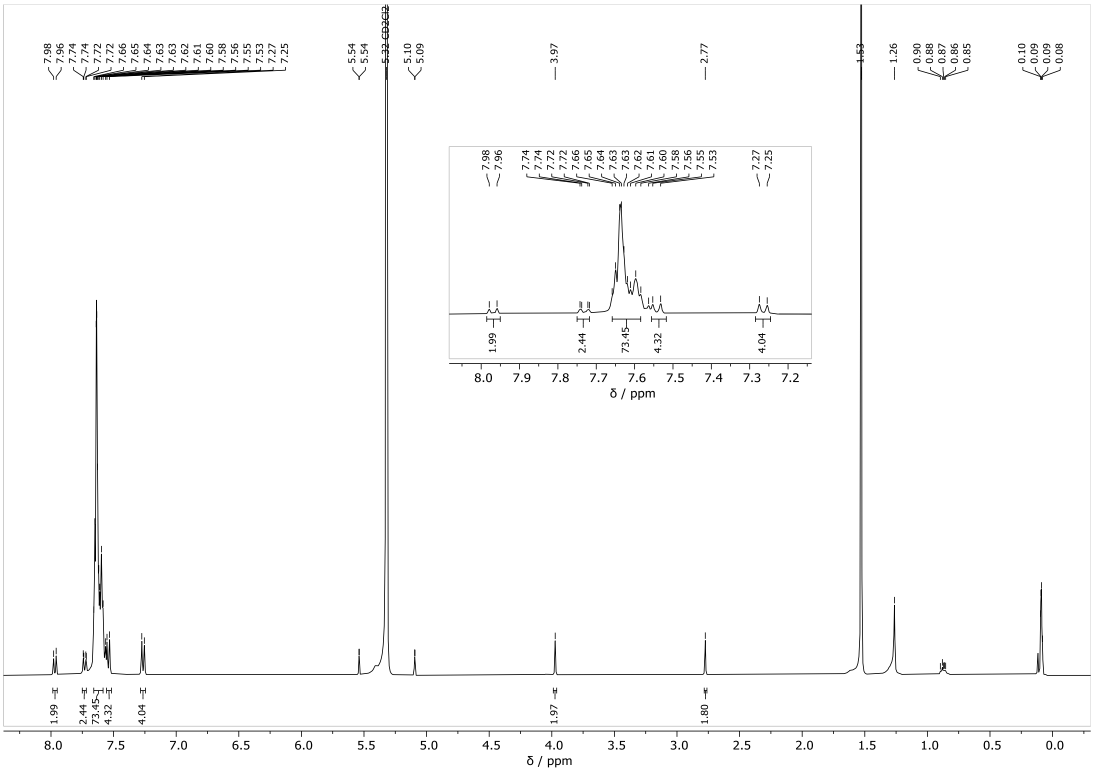

Figure S 69: ^{1}H NMR spectrum of **[11,11]** (600 MHz, CD_2Cl_2).

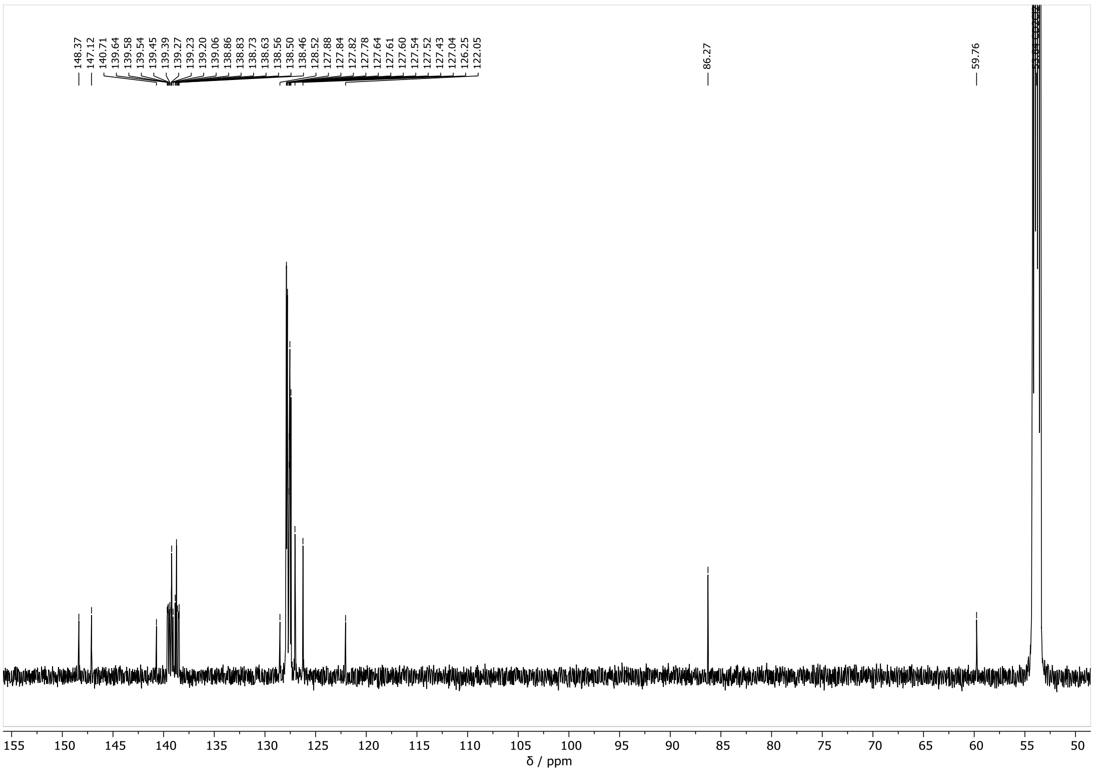

Figure S 70: ^{13}C NMR spectrum of **[11,11]** (151 MHz, CD_2Cl_2).

9 Single Crystal X-Ray Diffraction

A suitable crystal was obtained by vapor diffusion of methanol into a 1,2-dichlorobenzene solution. A yellow, black-shaped crystal was mounted on a MiTeGen MicroMount with perfluoroether oil. The diffraction data were collected at T = 105 K at the P24 beamline of the PETRA III at DESY (Hamburg, Germany) equipped with a four-circle HUBER four-circle diffractometer with Eulerian geometry, an X Spectrum LAMBDA 7.5M pixel array detector with a pixel size of 55×55 µm, and an openflow nitrogen cryosystem. The data were acquired by 360° rotation φ-scans with 0.5° scan width with 2θ = 0° and κ = −45° and an exposure time of 2 s per frame at the wavelength λ = 0.56 Å and a detector distance of 145 mm. All data were handled (importing, indexing, integration, scaling, absorption correction) using CrysAlisPro 1.171.43.66a (Rigaku OD 2024) and a multi-scan absorption correction using SCALE3 ABSPACK was applied.[48]The structure was solved by direct methods with SHELXT and refined by full-matrix least-squares methods against F2 using SHELXL-2018/3 within ShelXle.[49–51] Some atoms (10) were refined using isotropic displacement parameters. All other non-hydrogen atoms were refined with anisotropic displacement parameters. All C-bound hydrogen atoms were refined isotropic on calculated positions using a riding model with their Uiso values constrained to 1.5 times the Ueq of their pivot atoms for terminal sp3 carbon atoms and 1.2 times for all other carbon atoms. Crystallographic data for the structures reported in this paper have been deposited with the Cambridge Crystallographic Data Centre.[52] CCDC 2469367 contain the supplementary crystallographic data for this paper. These data can be obtained free of charge from The Cambridge Crystallographic Data Centre via www.ccdc.cam.ac.uk/structures. This report and the CIF file were generated using FinalCif.[53]

The structure contains large solvent-accessible voids with highly disordered solvent molecules. Explicit solvent molecules were modelled when possible. This resulted in 5 1,2-dichlorobenzene solvent molecules, modeled with the help of DSR[54,55], that are located inside the hoop cavity. The remaining undescribed electron density was therefore described using the PLATON/SQUEEZE procedure,[56] which removed approximately 4697 electrons in a volume of 1385 $Å^3$ per unit cell from the diffuse solvent regions. The reported model contains only the ordered framework and explicitly resolved solvent molecules. Since the hoop cavities form solvent accessible channels, unspecified amount of solvent molecules can be present, consisting of o-DCB and MeOH. Refinement was stable after applying SQUEEZE, with reasonable geometric parameters for the main structure. Some phenyl groups in the hoops are most likely rotatinally disordered along their para-connection over two positions. Because of

the bad data set, it was chosen not to model this disorder, but rather accept the large ADP. Due to the overall bad quality of the dataset, the atoms were restrained using RIGU on the whole carbon skeleton.

We are aware, that the presented structure does not show the best parameters and quality values. We obtained multiple datasets on different visits to the synchrotron facility from different crystals and crystallization attempts. Although the crystals looked optically fine and were large enough, we were not able to obtain any better dataset. Still, we are confident in the structure solution despite the mediocre refinement. The core structure and its connectivity is well defined and reasonable. The main issue with this structure is the large disorder caused by many solvent molecules inside of the hoop cavities which form continuous channels.

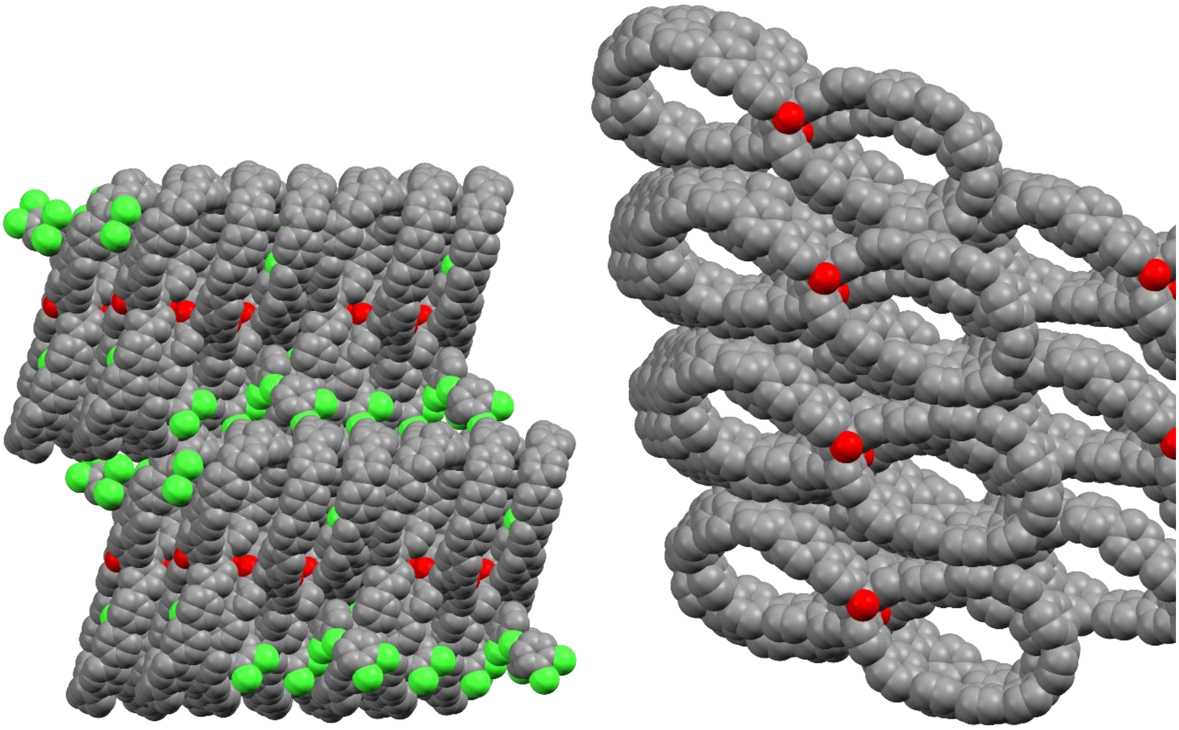

Figure S 71: Solid state molecular packing of **[11,9]** with (left) and without (right) solvent molecules. Hydrogen atoms are omitted for clarity.

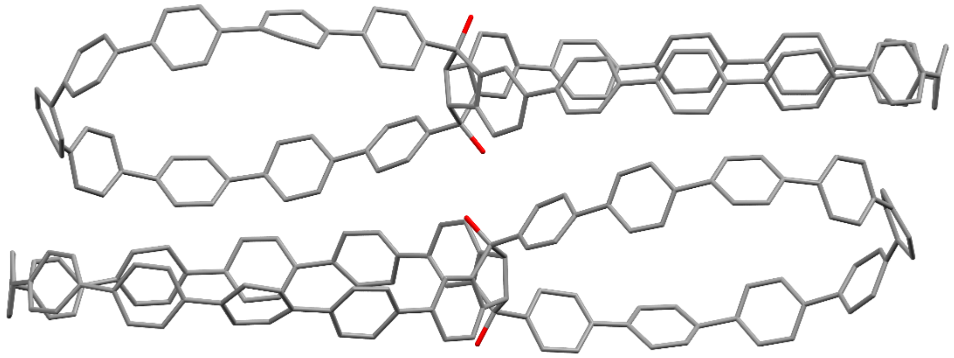

Figure S 72: Side view of molecular packing of **[11,9]**.

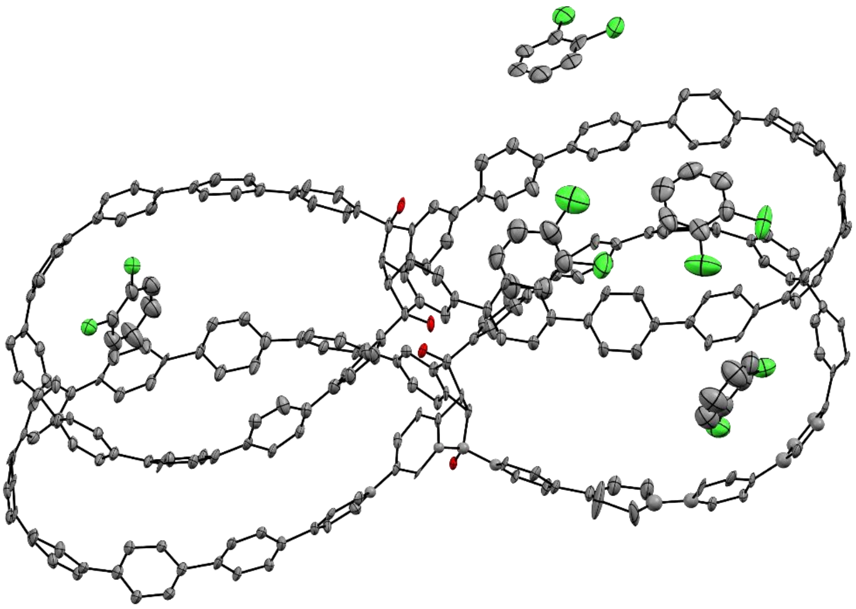

Figure S 73: Molecular Structure of (R,R,R,R)-[**11,9]** (bottom) and (S,S,S,S)-[**11,9]** (top). Displacement ellipsoids are shown at the 50% probability level; hydrogen atoms are omitted for clarity.

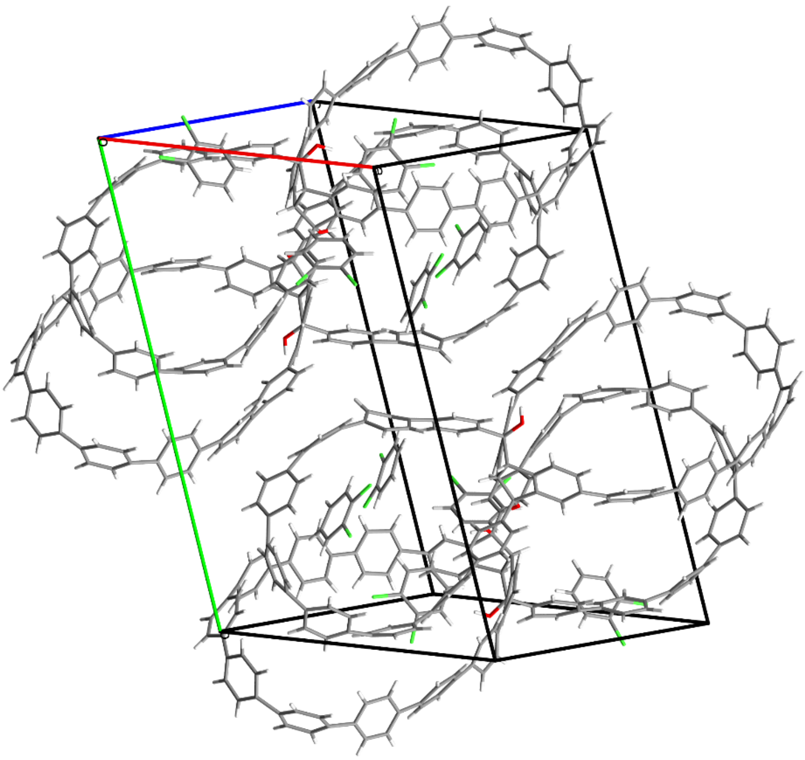

Figure S 74: Packing of **[11,9]** with unit cell shown.

Table S 1: Crystal data and structre refinement data for **[11,9]**.

CCDC number	2469367
Empirical formula	$C_{278}H_{184}Cl_{10}O_4$
Formula weight	3942.74
Temperature [K]	105(2)
Crystal system	triclinic
Space group (number)	$P\overline{1}$ (2)
a [Å]	15.5773(5)
b [Å]	29.2539(10)
c [Å]	31.7834(8)
α [°]	70.716(3)
β [°]	84.575(2)
γ [°]	77.197(3)
Volume [Å^3]	13327.5(8)
Z	2
ρ_{calc} [gcm^{-3}]	0.982
μ [mm^{-1}]	0.084
F(000)	4108
Crystal size [mm^3]	0.200×0.300×0.400
Crystal colour	yellow
Crystal shape	block
Radiation	synchrotron (λ=0.560 Å)
2θ range [°]	3.03 to 30.93 (1.05 Å)
Index ranges	$-14 \leq h \leq 14$<br>$-27 \leq k \leq 27$<br>$-30 \leq l \leq 30$
Reflections collected	66226
Independent reflections	22516<br>R_{int} = 0.0550<br>R_{sigma} = 0.0687
Completeness to<br>θ = 15.466°	93.3 %
Data / Restraints / Parameters	22516 / 3623 / 2579
Absorption correction T_{min}/T_{max} (method)	0.5503 / 1.0000<br>(multi-scan)
Goodness-of-fit on F^2	1.041
Final R indexes<br>[$I \geq 2\sigma(I)$]	R_1 = 0.2212<br>wR_2 = 0.5744
Final R indexes<br>[all data]	R_1 = 0.2629<br>wR_2 = 0.6447
Largest peak/hole [eÅ^{-3}]	1.66/−0.93

10 DFT Calculations

10.1 Optimized Geometries

The coordinates of the molecules optimized on PBEh-3c level are listed in Table S 12–Table S 25.

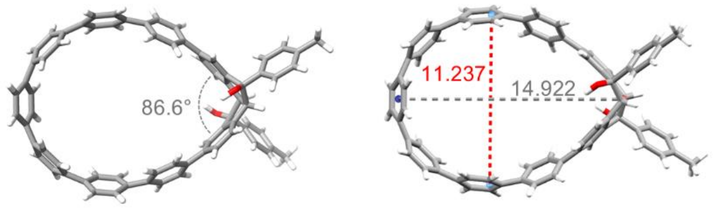

Figure S 75: Optimized geometry (PBEh-3c) of **[9,0]** shown with the angle of the tetrahydroindenoindene unit (left) and the cavity dimensions in Å (right).

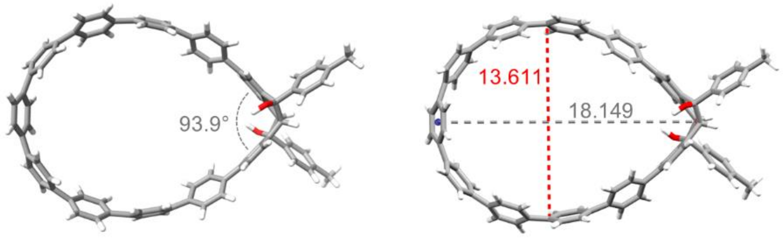

Figure S 76: Optimized geometry (PBEh-3c) of **[11,0]** shown with the angle of the tetrahydroindenoindene unit (left) and the cavity dimensions in Å (right).

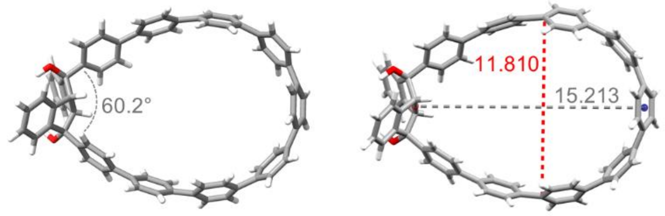

Figure S 77: Optimized geometry (PBEh-3c) of **[0,9]** shown with the angle of the loop (left) and the cavity dimensions in Å (right).

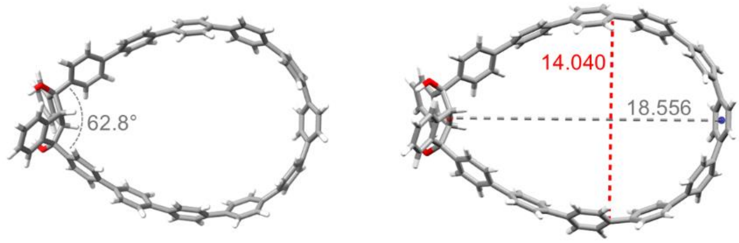

Figure S 78: Optimized geometry (PBEh-3c) of **[0,11]** shown with the angle of the loop (left) and the cavity dimensions in Å (right).

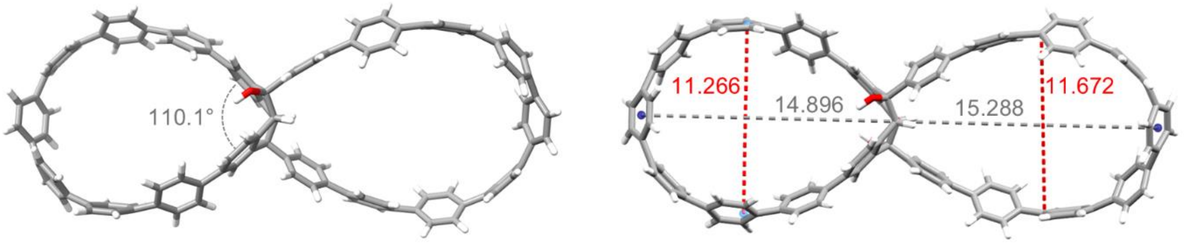

Figure S 79: Optimized geometry (PBEh-3c) of **[9,9]** shown with the angle of the tetrahydroindenoindene unit (left) and the cavity dimensions in Å (right).

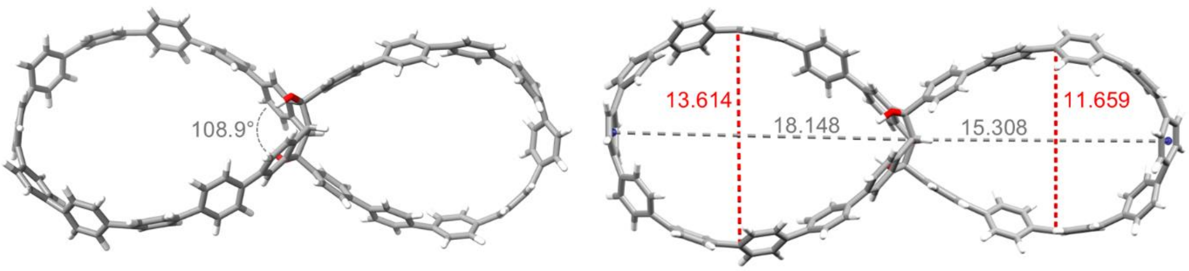

Figure S 80: Optimized geometry (PBEh-3c) of **[11,9]** shown with the angle of the tetrahydroindenoindene unit (left) and the cavity dimensions in Å (right).

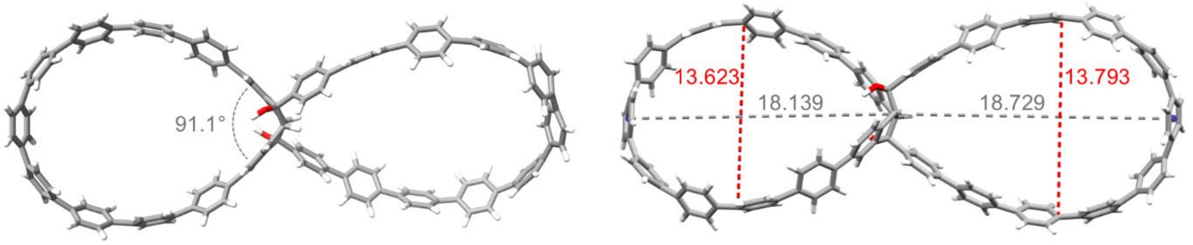

Figure S 81: Optimized geometry (PBEh-3c) of **[11,11]** shown with the angle of the tetrahydroindenoindene unit (left) and the cavity dimensions in Å (right).

10.2 Energies of Optimized Geometries

Table S 2: Calculated electronic and vibrational energies.

#	E_{SP}[a] / Hartrees	ZPVE[b] / Hartrees	E_{tot}[c] / Hartrees
**[9,0]**	−2930.7177945	1.0544667	−2929.6633278
**[11,0]**	−3393.6788501	1.2214389	−3392.4574112
**[0,9]**	−2851.9559421	0.9986304	−2850.9573117
**[0,11]**	−3314.9138724	1.1623672	−3313.7515052
**[9,9]**	−4471.0165130	1.5602922	−4469.4562208
**[11,9]**	−4933.9771618	1.7271534	−4932.2500084
**[11,11]**	−5396.9352642	1.8940983	−5395.0411659

[a] E_{SP} on PW6B95/def2-QZVP level of theory. [b] From freeh script on PBEh-3c level of theory.
[c] $E_{tot} = E_{SP}$ + ZPVE.

Table S 3: Calculated energies including thermal free energy correction $G_{free,th}$ and thermal free enthalpy correction $H_{free,th}$ at T = 298.15 K and P = 0.1 MPa.

#	$G_{free,th}$[a] / kJ·mol^{-1}	G[b] / Hartrees	$H_{free,th}$[a] / kJ·mol^{-1}	H[c] / Hartrees
**[9,0]**	2892.77	−2929.6167335	2895.25	−2929.6157895
**[11,0]**	3351.82	−3392.4030631	3354.30	−3392.4021191
**[0,9]**	2737.17	−2850.9141064	2739.64	−2850.9131663
**[0,11]**	3195.71	−3313.6975048	3198.18	−3313.6965647
**[9,9]**	4278.56	−4469.3879855	4281.04	−4469.3870415
**[11,9]**	4737.35	−4932.1740072	4739.83	−4932.1730633
**[11,11]**	5196.33	−5394.9574104	5198.81	−5394.9564664

[a] From freeh script on PBEh-3c level of theory. [b] $G = E_{SP} + G_{free}$. E_{SP} on PW6B95/def2-QZVP level of theory. [c] $H = E_{SP} + H_{free}$. E_{SP} on PW6B95/def2-QZVP level of theory.

10.3 Frontier Molecular Orbitals

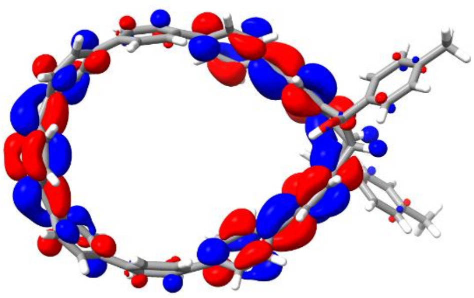

LUMO+2 E_{PW6B95} = −1.23 eV

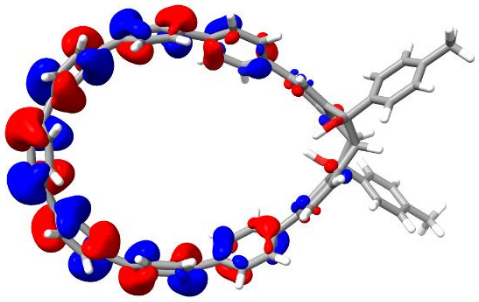

HOMO E_{PW6B95} = −5.90 eV

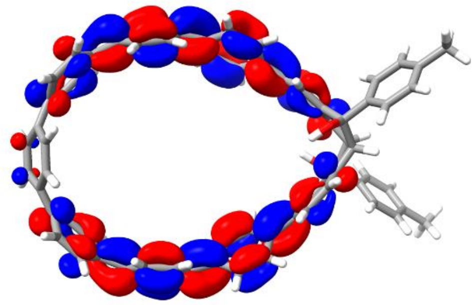

LUMO+1 E_{PW6B95} = −1.42 eV

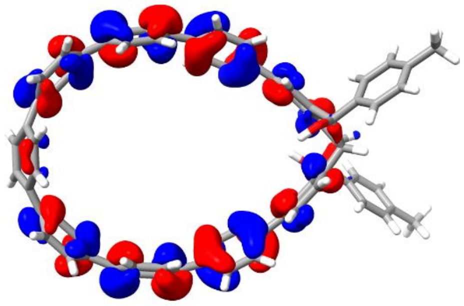

HOMO−1 E_{PW6B95} = −6.21 eV

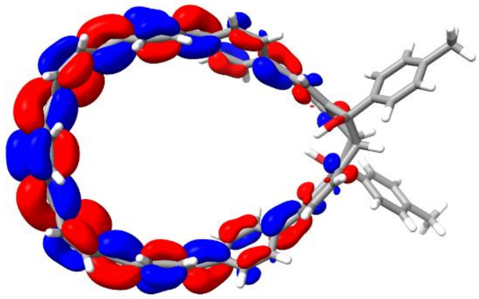

LUMO E_{PW6B95} = −1.71 eV

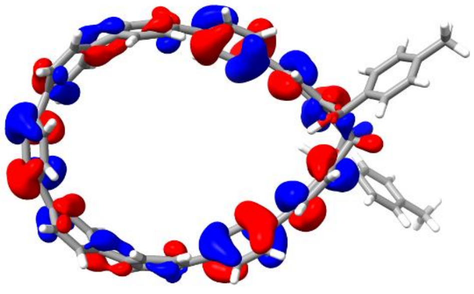

HOMO−2 E_{PW6B95} = −6.48 eV

Figure S 82: Frontier molecular orbitals of **[9,0]** with respective energies calculated on PW6B95/def2-QZVP level of theory with an isovalue of ±0.02.

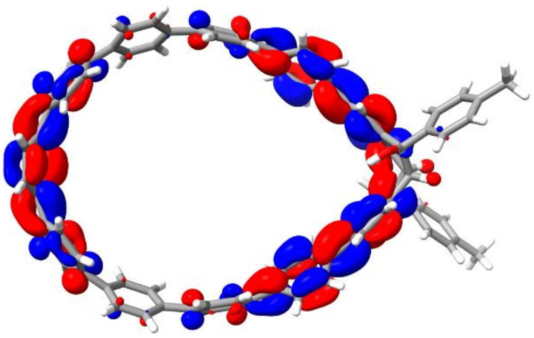

LUMO+2 E_{PW6B95} **= −1.32 eV**

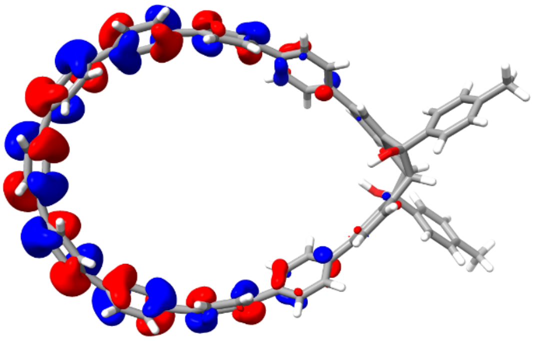

HOMO E_{PW6B95} **= −5.93 eV**

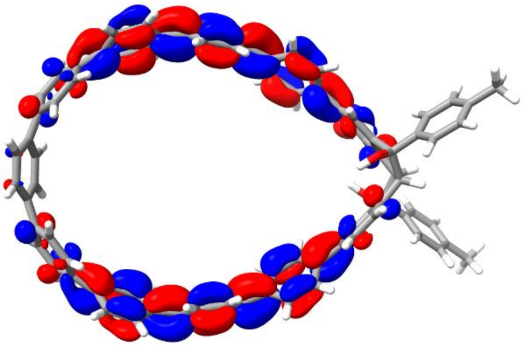

LUMO+1 E_{PW6B95} **= −1.48 eV**

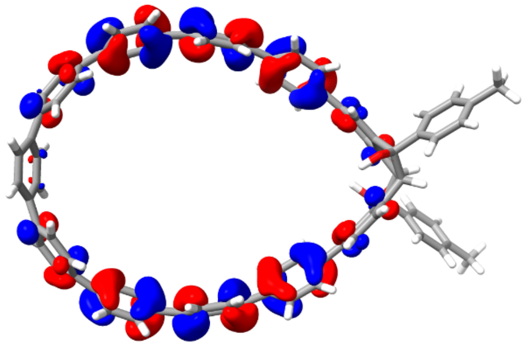

HOMO−1 E_{PW6B95} **= −6.15 eV**

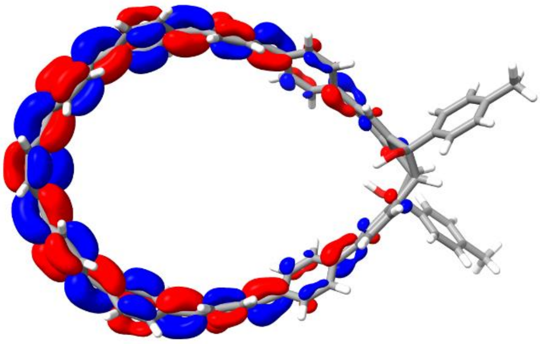

LUMO E_{PW6B95} **= −1.69 eV**

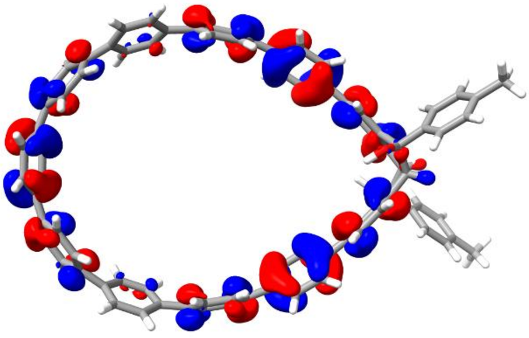

HOMO−2 E_{PW6B95} **= −6.36 eV**

Figure S 83: Frontier molecular orbitals of **[11,0]** with respective energies calculated on PW6B95/def2-QZVP level of theory with an isovalue of ±0.02.

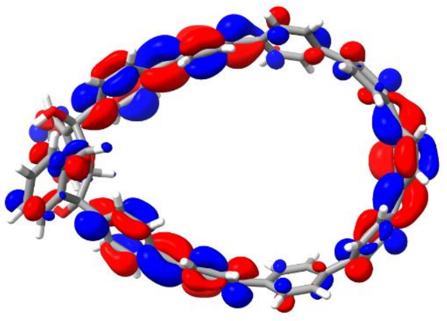

LUMO+2 $E_{PW6B95} = -1.08$ **eV**

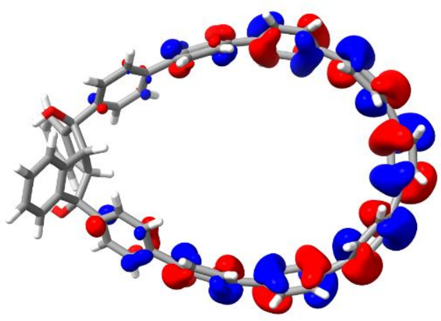

HOMO $E_{PW6B95} = -5.87$ **eV**

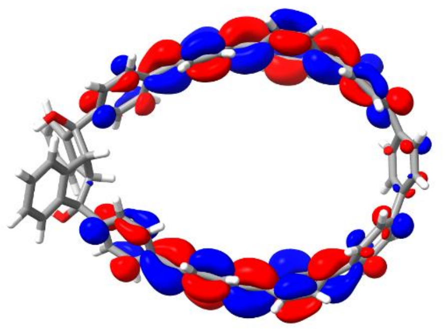

LUMO+1 $E_{PW6B95} = -1.38$ **eV**

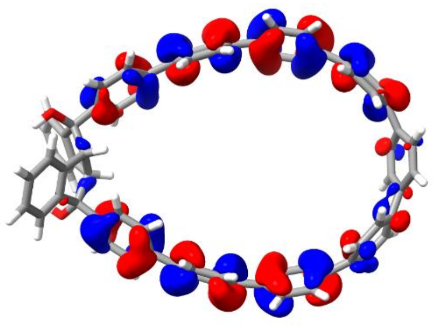

HOMO−1 $E_{PW6B95} = -6.12$ **eV**

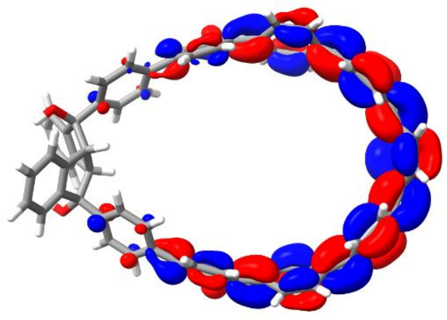

LUMO $E_{PW6B95} = -1.66$ **eV**

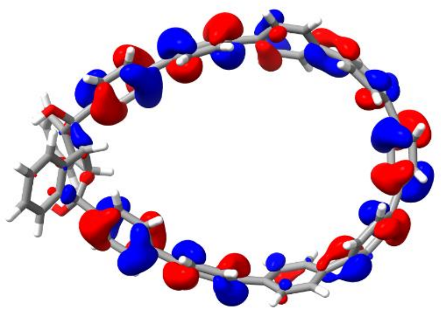

HOMO−2 $E_{PW6B95} = -6.43$ **eV**

Figure S 84: Frontier molecular orbitals of **[0,9]** with respective energies calculated on PW6B95/def2-QZVP level of theory with an isovalue of ±0.02.

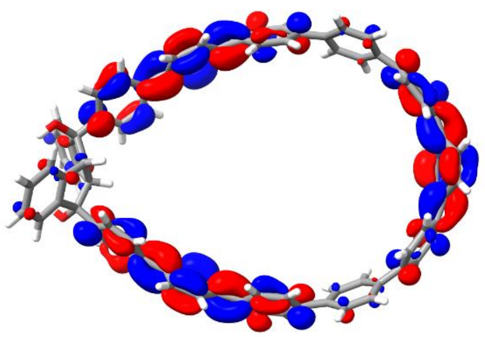

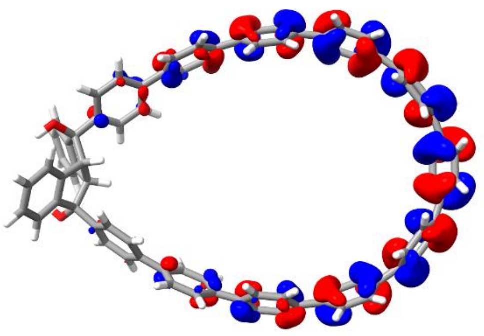

LUMO+2 $E_{\mathrm{PW6B95}} = -1.21$ **eV** **HOMO** $E_{\mathrm{PW6B95}} = -5.91$ **eV**

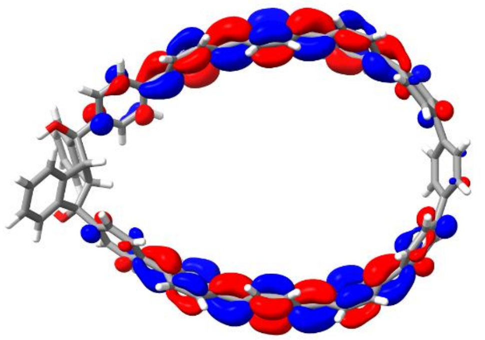

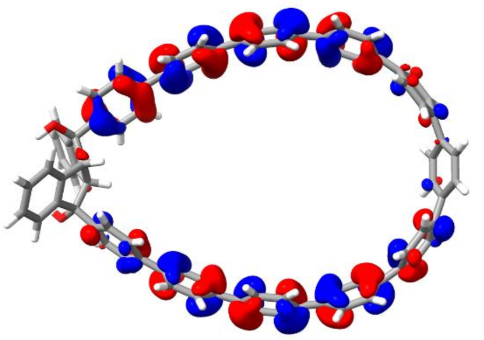

LUMO+1 $E_{\mathrm{PW6B95}} = -1.44$ **eV** **HOMO−1** $E_{\mathrm{PW6B95}} = -6.09$ **eV**

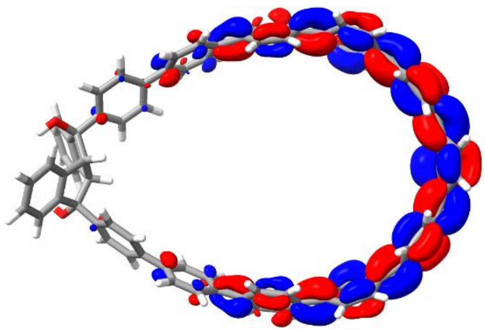

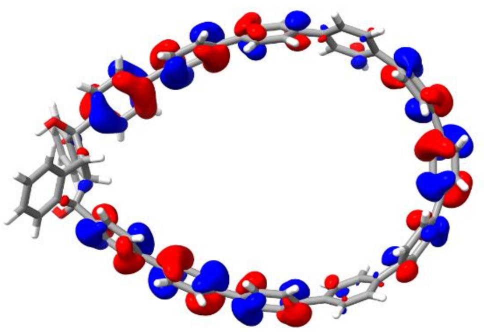

LUMO $E_{\mathrm{PW6B95}} = -1.65$ **eV** **HOMO−2** $E_{\mathrm{PW6B95}} = -6.30$ **eV**

Figure S 85: Frontier molecular orbitals of **[0,11]** with respective energies calculated on PW6B95/def2-QZVP level of theory with an isovalue of ±0.02.

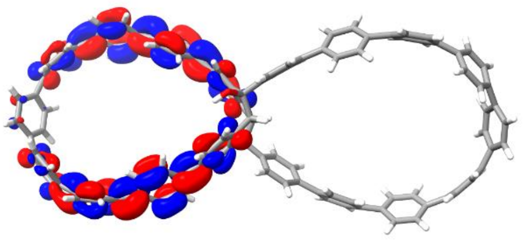

LUMO+2 $\boldsymbol{E_{PW6B95}}$ **= −1.47 eV**

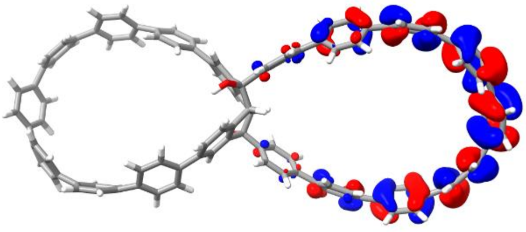

HOMO $\boldsymbol{E_{PW6B95}}$ **= −5.89 eV**

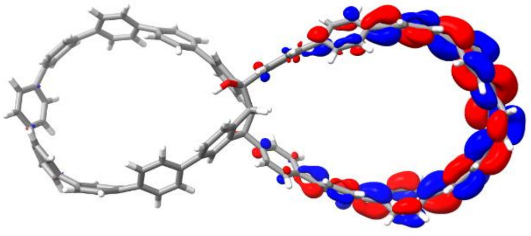

LUMO+1 $\boldsymbol{E_{PW6B95}}$ **= −1.66 eV**

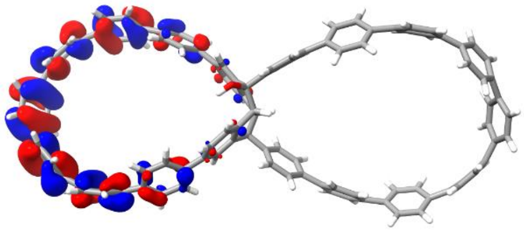

HOMO−1 $\boldsymbol{E_{PW6B95}}$ **= −5.93 eV**

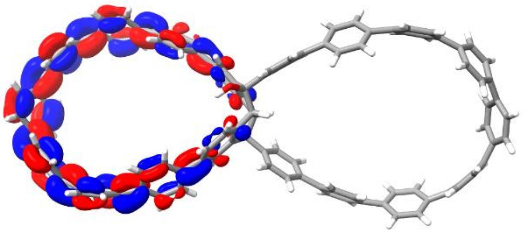

LUMO $\boldsymbol{E_{PW6B95}}$ **= −1.76 eV**

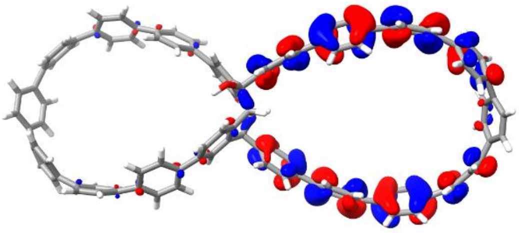

HOMO−2 $\boldsymbol{E_{PW6B95}}$ **= −6.16 eV**

Figure S 86: Frontier molecular orbitals of **[9,9]** with respective energies calculated on PW6B95/def2-QZVP level of theory with an isovalue of ±0.02.

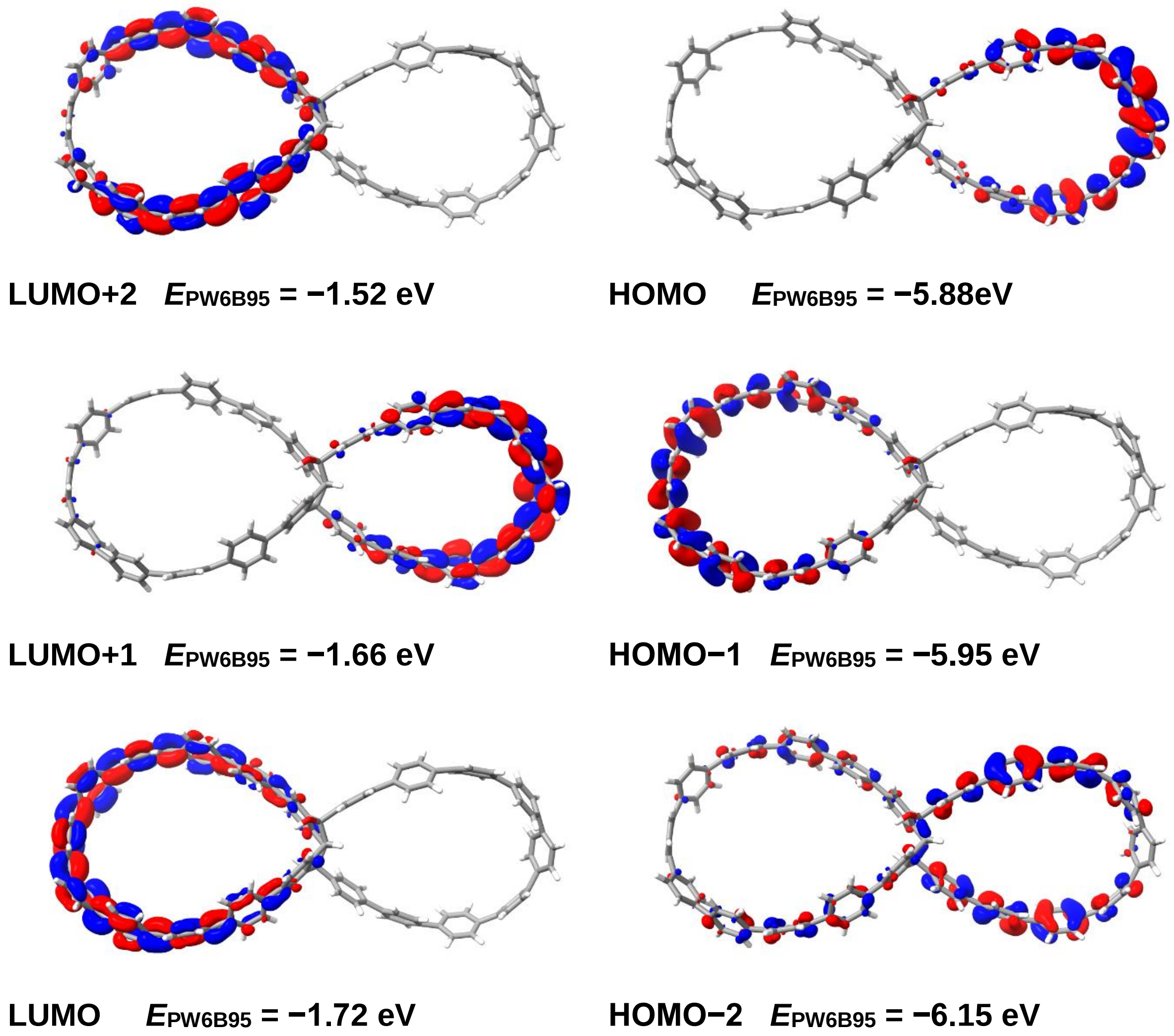

Figure S 87: Frontier molecular orbitals of **[11,9]** with respective energies calculated on PW6B95/def2-QZVP level of theory with an isovalue of ±0.02.

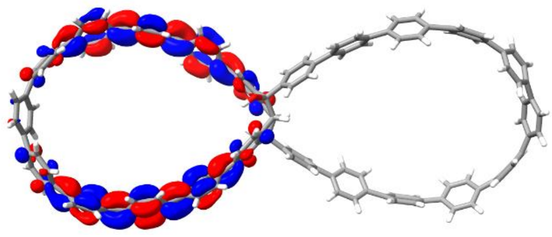

LUMO+2 ***E***$_{PW6B95}$ **= −1.52 eV**

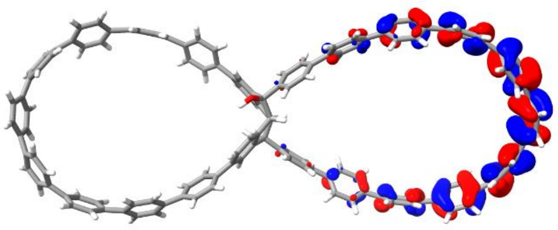

HOMO ***E***$_{PW6B95}$ **= −5.92 eV**

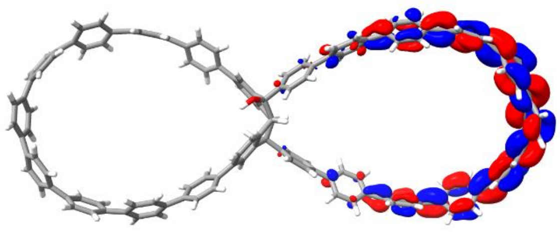

LUMO+1 ***E***$_{PW6B95}$ **= −1.65 eV**

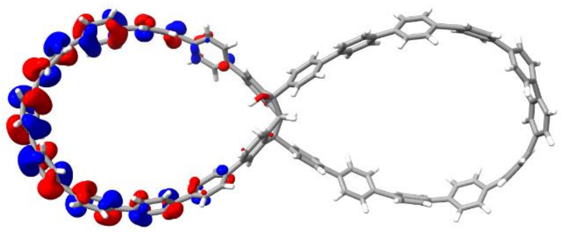

HOMO−1 ***E***$_{PW6B95}$ **= −5.95 eV**

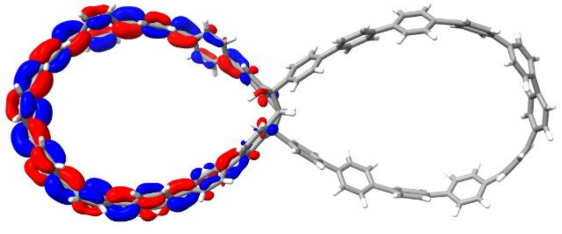

LUMO ***E***$_{PW6B95}$ **= −1.72 eV**

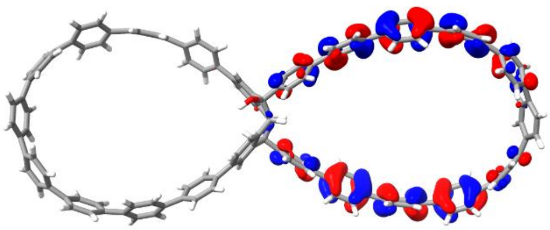

HOMO−2 ***E***$_{PW6B95}$ **= −6.11 eV**

Figure S 88: Frontier molecular orbitals of **[11,11]** with respective energies calculated on PW6B95/def2-QZVP level of theory with an isovalue of ±0.02.

10.4 StrainViz Calculations

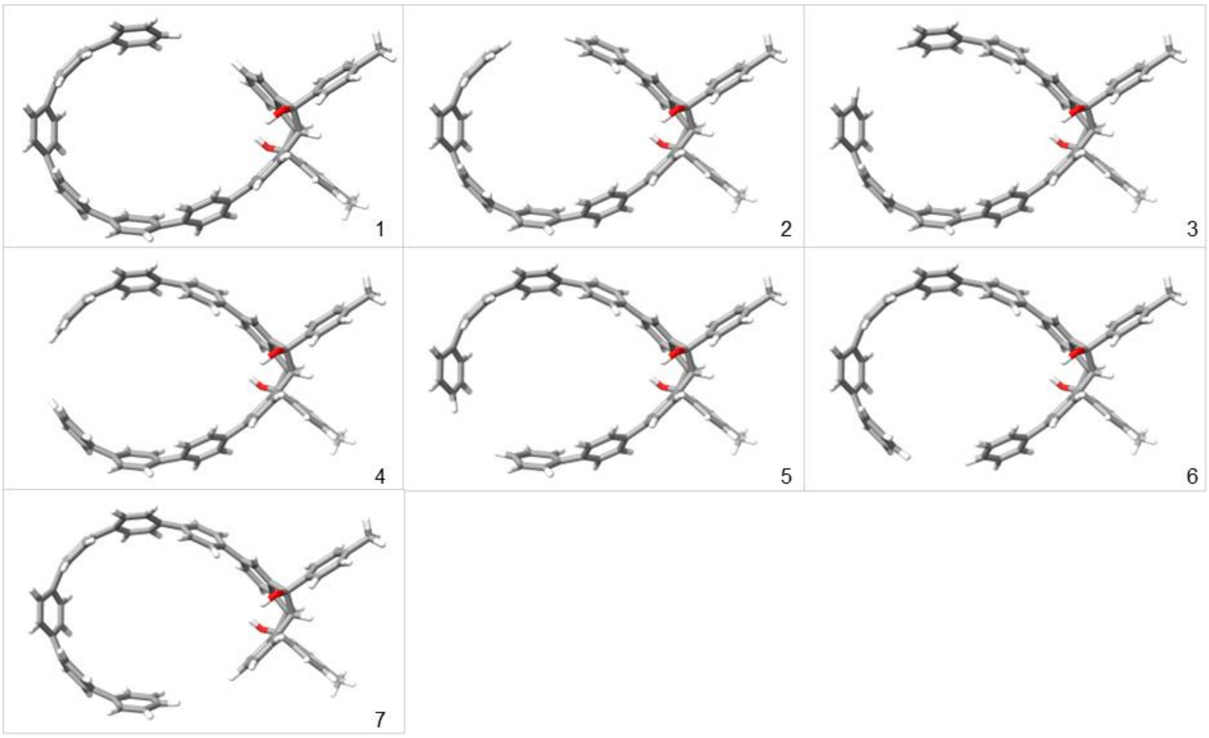

Figure S 89: StrainViz fragments of **[9,0]**.

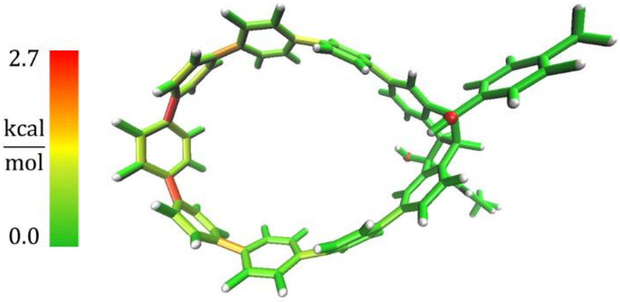

Total strain	44.4 kcal/mol
Bond strain	3.4 kcal/mol
Angle strain	3.2 kcal/mol
Dihedral strain	37.8 kcal/mol

Figure S 90: StrainViz mapping of **[9,0]** (left) with strain distribution (right).

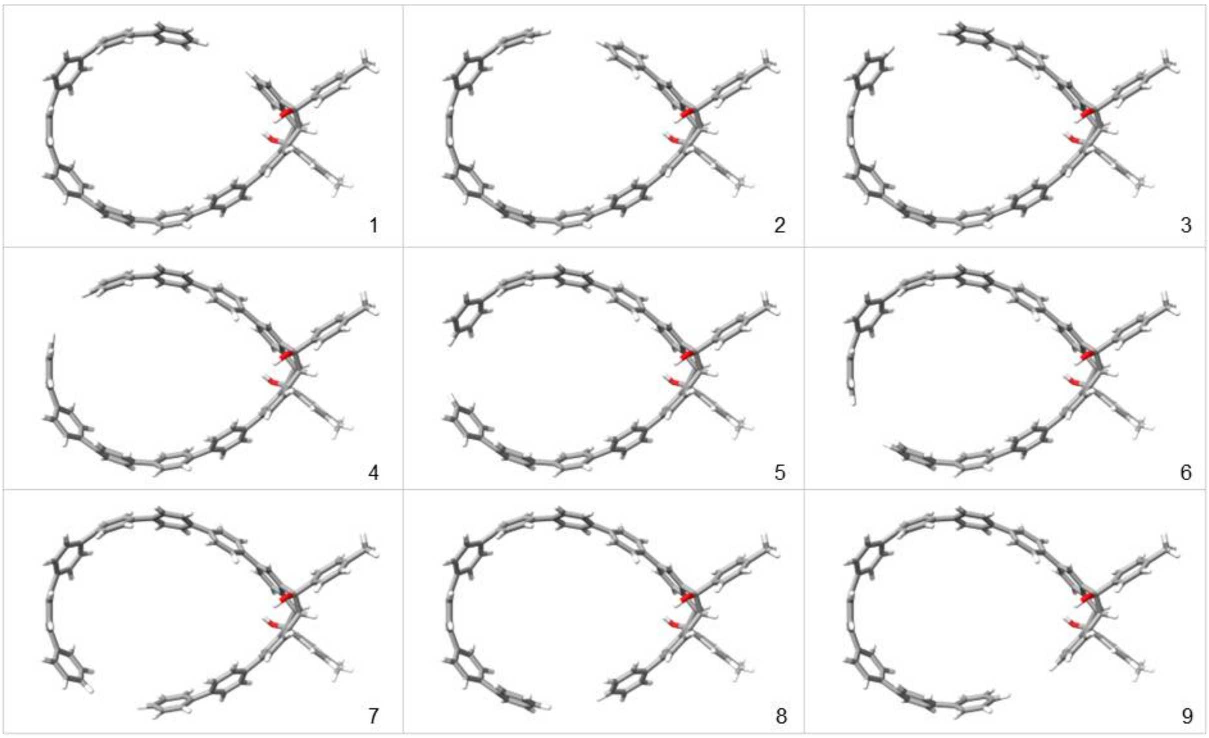

Figure S 91: StrainViz fragments of **[11,0]**.

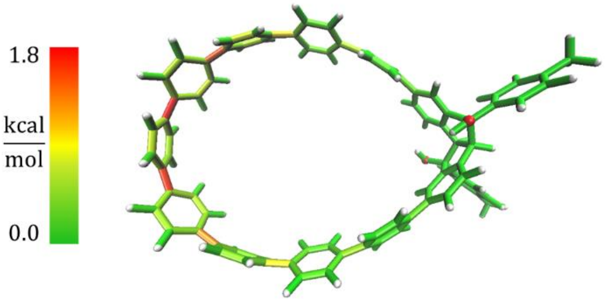

Total strain	37.4 kcal/mol
Bond strain	2.9 kcal/mol
Angle strain	2.5 kcal/mol
Dihedral strain	32.0 kcal/mol

Figure S 92: StrainViz mapping of **[11,0]** (left) with strain distribution (right).

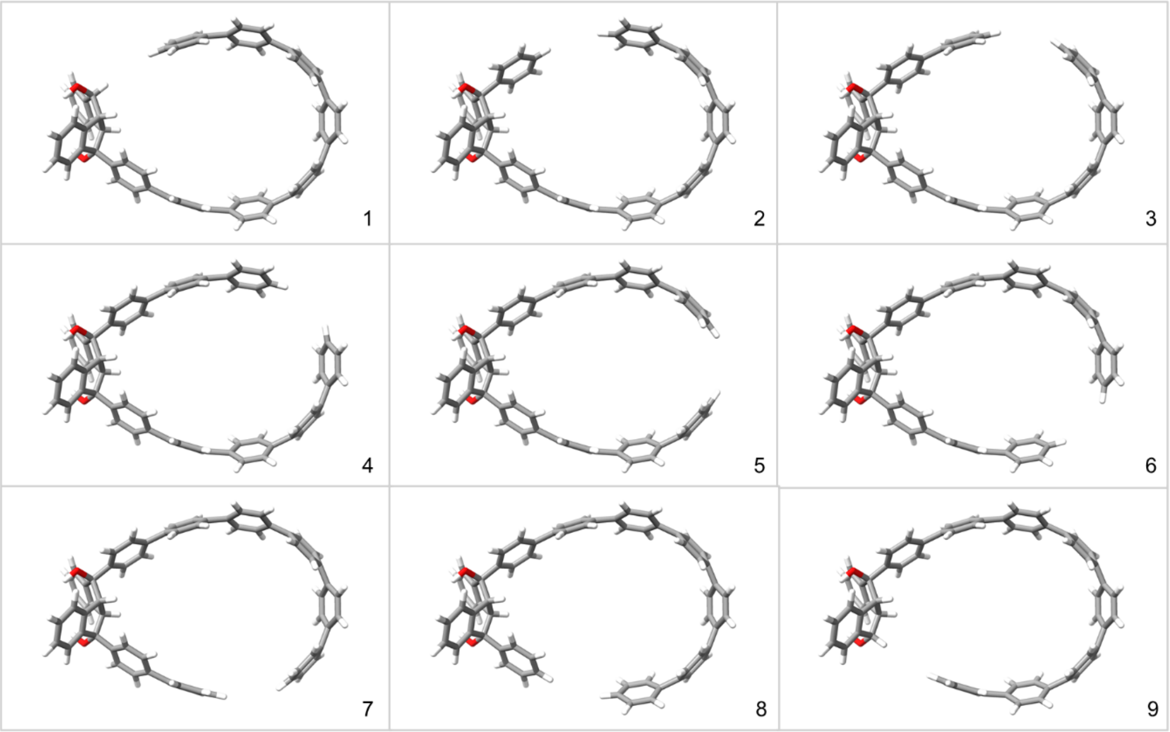

Figure S 93: StrainViz fragments of **[0,9]**.

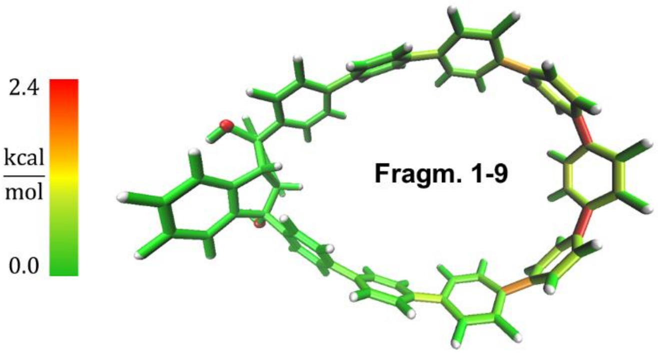

Total strain	38.4 kcal/mol
Bond strain	2.9 kcal/mol
Angle strain	2.9 kcal/mol
Dihedral strain	32.6 kcal/mol

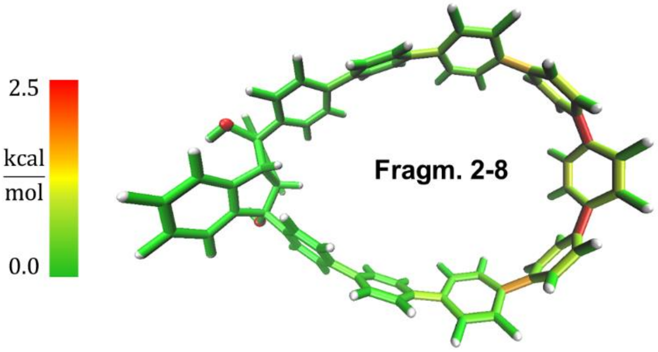

Total strain	37.6 kcal/mol
Bond strain	3.4 kcal/mol
Angle strain	2.6 kcal/mol
Dihedral strain	31.6 kcal/mol

Figure S 94: StrainViz mapping of **[0,9]** (left) with strain distribution (right). The top calculation (fragm. 1-9) serves as comparison to **[11,9]**, the bottom (fragm. 2-8) as comparison to **[9,9]**.

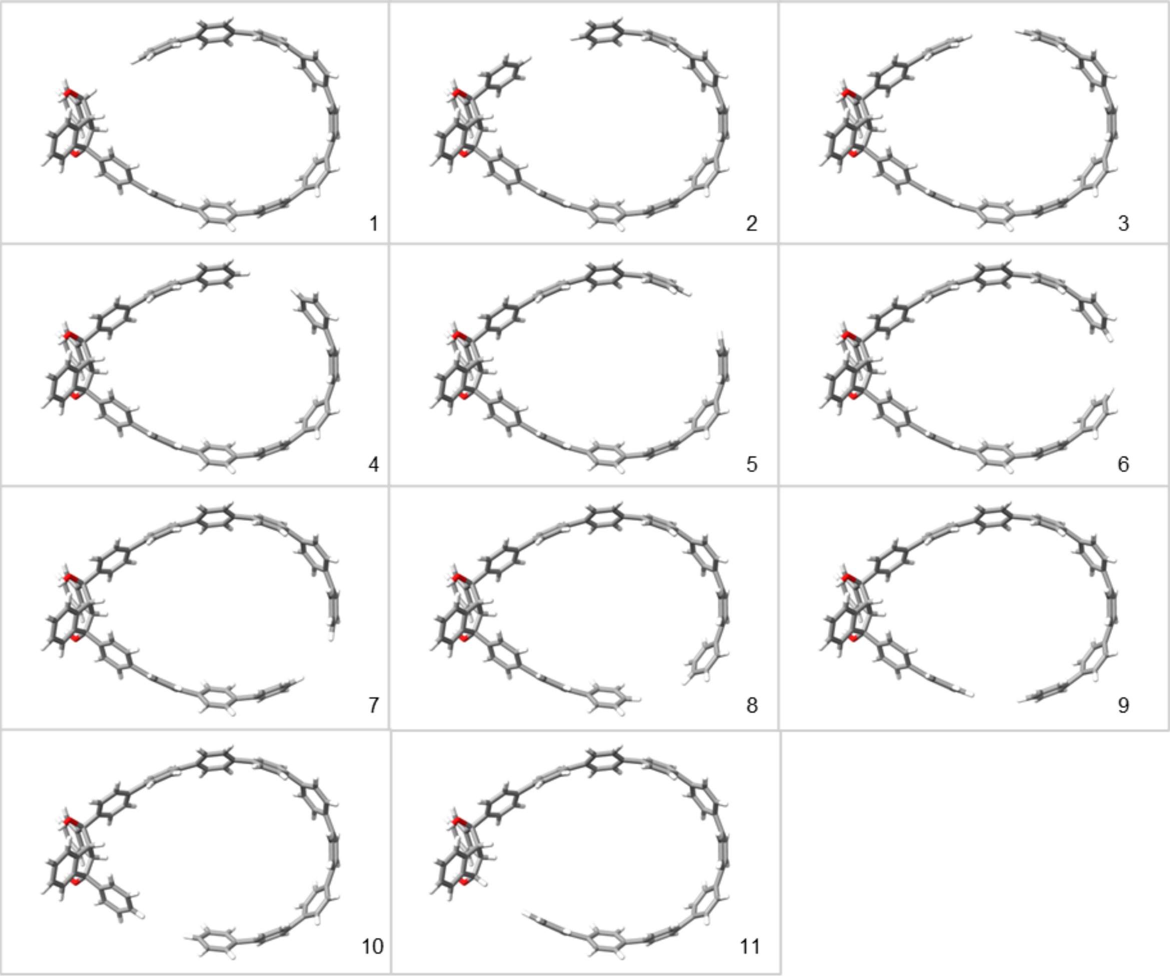

Figure S 95: StrainViz fragments of **[0,11]**.

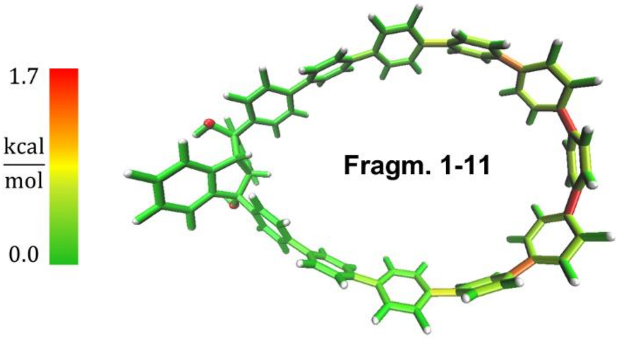

Total strain	32.8 kcal/mol
Bond strain	2.3 kcal/mol
Angle strain	2.7 kcal/mol
Dihedral strain	27.8 kcal/mol

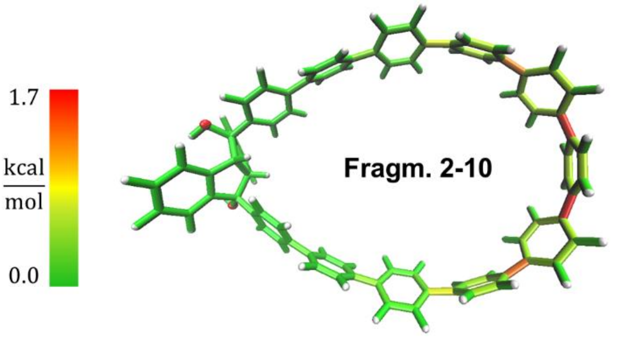

Total strain	32.4 kcal/mol
Bond strain	2.7 kcal/mol
Angle strain	2.6 kcal/mol
Dihedral strain	27.1 kcal/mol

Figure S 96: StrainViz mapping of **[0,11]** (left) with strain distribution (right). The top calculation (fragm. 1-11) serves as comparison to **[11,11]**, the bottom (fragm. 2-10) as control.

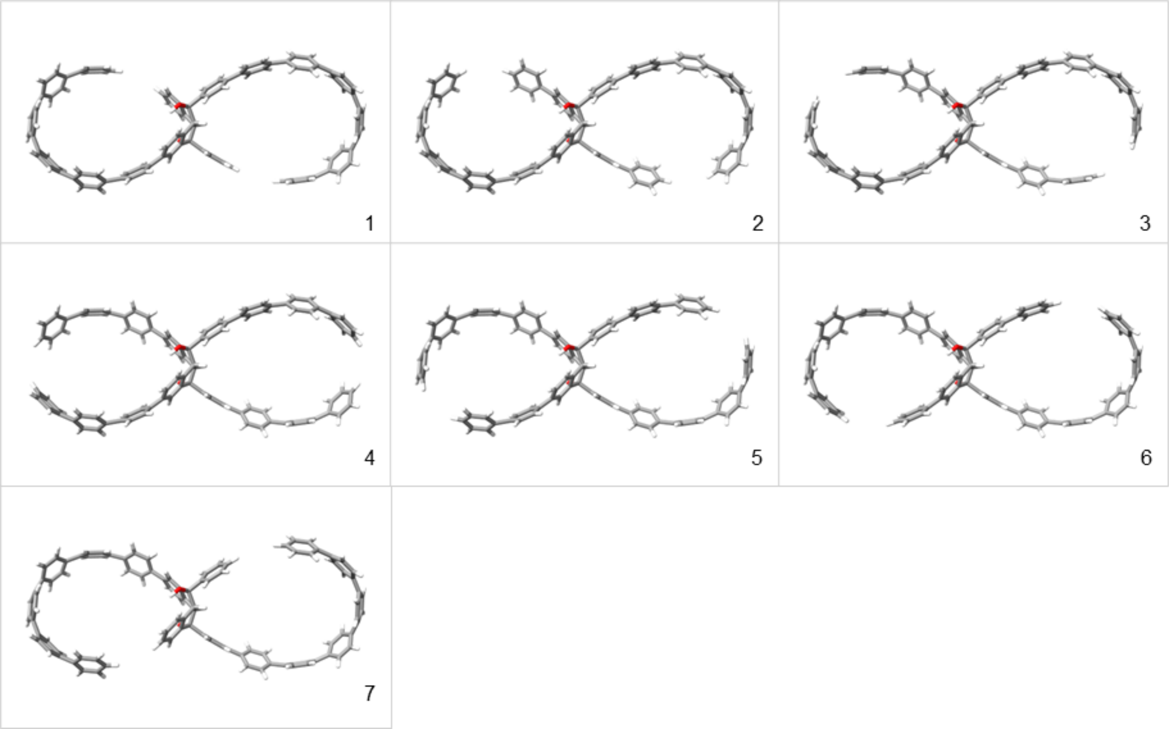

Figure S 97: StrainViz fragments of **[9,9]**.

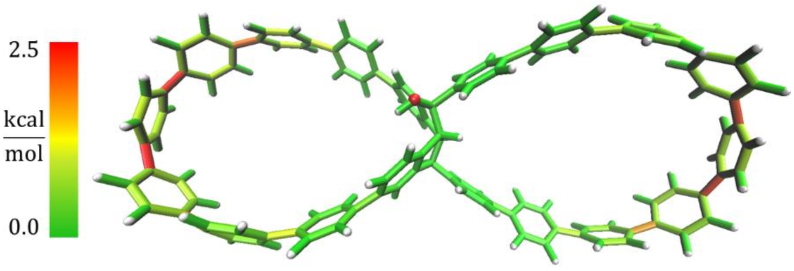

Total strain	82.3 kcal/mol
Bond strain	7.3 kcal/mol
Angle strain	4.7 kcal/mol
Dihedral strain	70.2 kcal/mol

Figure S 98: StrainViz mapping of **[9,9]** (left) with strain distribution (right).

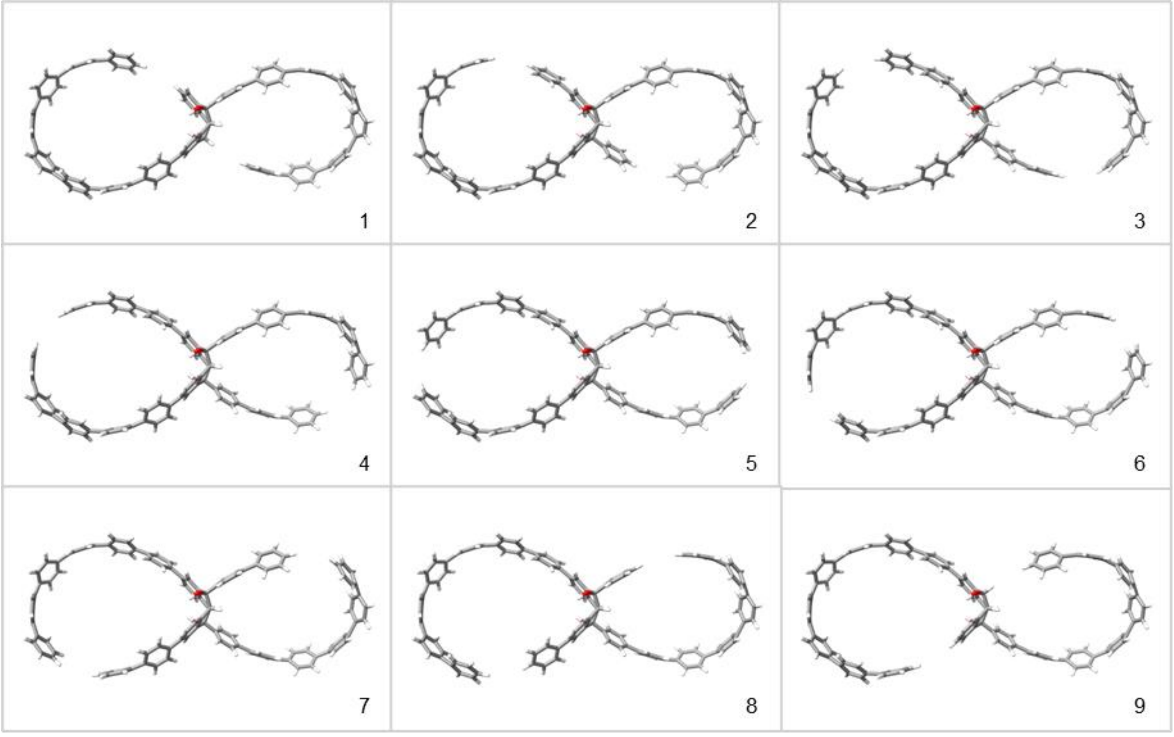

Figure S 99: StrainViz fragments of **[11,9]**.

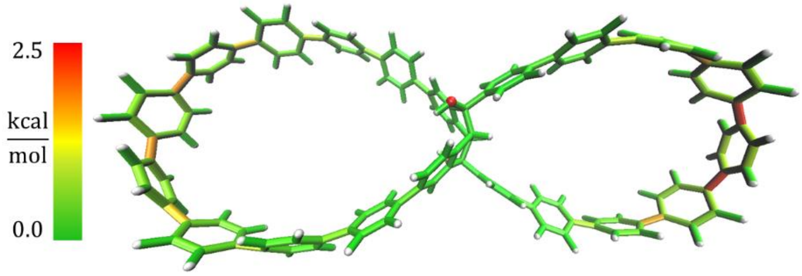

Total strain	76.1 kcal/mol
Bond strain	4.5 kcal/mol
Angle strain	6.0 kcal/mol
Dihedral strain	65.6 kcal/mol

Figure S 100: StrainViz mapping of **[11,9]** (left) with strain distribution (right).

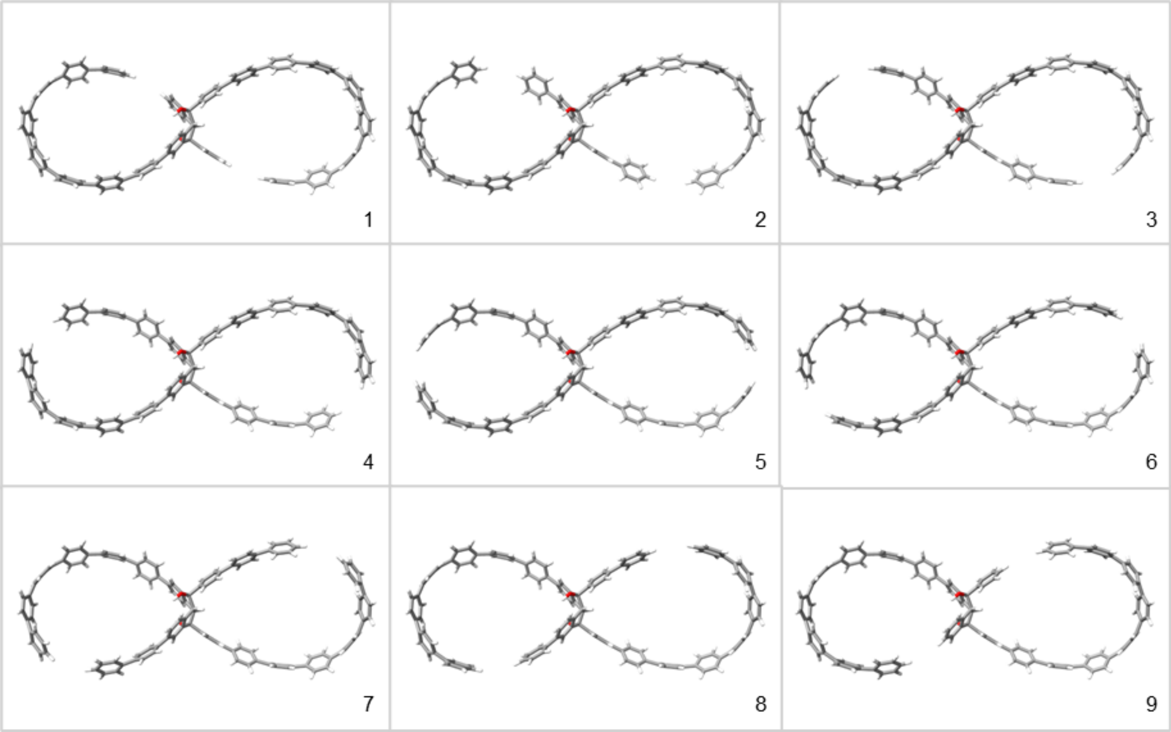

Figure S 101: StrainViz fragments of **[11,11]**.

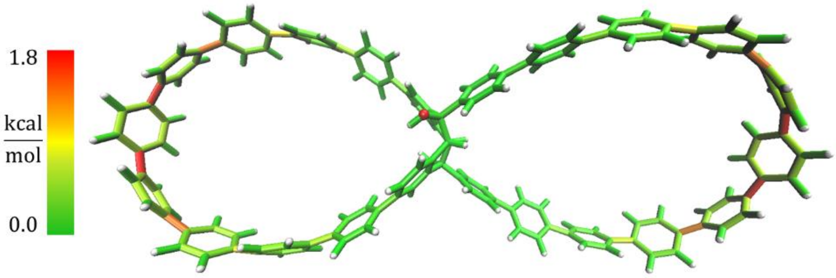

Total strain	70.2 kcal/mol
Bond strain	3.8 kcal/mol
Angle strain	5.8 kcal/mol
Dihedral strain	60.5 kcal/mol

Figure S 102: StrainViz mapping of **[11,11]** (left) with strain distribution (right).

10.5 TD-DFT results

The dissymmetry factor g_{calc}[57,58]. was calculated for each excitation using the formula

$$g_{calc.} = \frac{4R}{f} \approx \frac{\Delta\varepsilon}{\varepsilon}$$

under the assumption of dominating electronic dipole transitions for an isotropic substance, where R is the rotatory strength (length representation, dimensionless) and f is the oscillator strength (length representation, dimensionless) determined in the TD-DFT calculations on PBE0/def2-TZVP level.

Table S 4*:* Excitations calculated for **[9,0]** on PBE0/def2-TZVP level of theory. The excitation with the greatest oscillator strength (f_{L}) is highlighted in orange. The dissymmetry factor g is calculated based on the rotatory strength R and f.

Exc. #	E_{exc} / eV	λ / nm	From	→	To	Contr. (%)	f_{L}	R_{L}	$g_{\text{calc.}}$
**1**	3.376	367.3	H	→	L	90	0.2249	0.0014	0.0249
**2**	3.841	322.8	H	→	L+1	50	2.0044	−0.0076	−0.0152
			H−1	→	L	45			
**3**	3.931	315.4	H−1	→	L	51	0.0086	0	0.0000
			H	→	L+1	45			
**4**	4.083	303.6	H	→	L+2	51	0.7121	0.0054	0.0303
			H−1	→	L+1	23			
			H−2	→	L	19			
**5**	4.150	298.7	H	→	L+2	44	0.0051	0.0009	0.7059
			H−1	→	L+1	31			
			H−2	→	L	18			
**6**	4.210	294.5	H−2	→	L	59	0.0504	0.0003	0.0238
			H−1	→	L+1	35			
**7**	4.363	284.2	H−1	→	L+2	75	0.0292	−0.0004	−0.0548
**8**	4.399	281.9	H	→	L+6	51	0.0332	−0.0002	−0.0241
			H−10	→	L	12			
**9**	4.457	278.2	H−2	→	L+1	67	0.1095	−0.0012	−0.0438
			H−3	→	L	13			
**10**	4.467	277.6	H−3	→	L	28	0.0054	0.0001	0.0741
			H	→	L+3	15			
			H	→	L+7	14			
**11**	4.480	276.7	H	→	L+7	20	0.0455	−0.0003	−0.0264
			H−3	→	L	16			
**12**	4.555	272.2	H	→	L+3	22	0.1208	−0.0007	−0.0232
			H	→	L+9	14			
			H−3	→	L	14			
**13**	4.573	271.1	H−2	→	L+2	24	0.0020	−0.0001	−0.2000
			H	→	L+4	14			
**14**	4.594	269.9	H	→	L+5	25	0.0097	0.0001	0.0412
			H−2	→	L+5	10			

**15**	4.631	267.7	H−2	→	L+2	26	0.0087	0.0008	0.3678
			H	→	L+3	11			
**16**	4.646	266.9	H	→	L+3	15	0.0092	0.0004	0.1739
			H−4	→	L	14			
			H−5	→	L	11			
**17**	4.660	266.1	H−4	→	L	13	0.0131	0	0
			H	→	L+12	12			
**18**	4.684	264.7	H−4	→	L	36	0.0090	−0.0007	−0.3111
			H−2	→	L+2	20			
**19**	4.713	263.1	H	→	L+15	16	0.0227	0.0001	0.0176
			H−5	→	L	16			
			H−17	→	L	15			
			H−3	→	L+1	13			
**20**	4.726	262.3	H−3	→	L+1	52	0.0719	−0.0002	−0.0111
**21**	4.732	262.0	H−5	→	L	36	0.0336	0.0003	0.0357
**22**	4.744	261.3	H−6	→	L	10	0.0034	−0.0001	−0.0992
**23**	4.783	259.2	H	→	L+4	15	0.0292	−0.0001	−0.0348
**24**	4.838	256.3	H	→	L+4	22	0.0115	−0.0001	−0.0348
			H−8	→	L	16			
			H−1	→	L+3	12			
**25**	4.873	254.4	H−1	→	L+3	53	0.0523	0.0003	0.0229
**26**	4.890	253.5	H−4	→	L+1	33	0.0231	0	0
			H−3	→	L+2	23			
**27**	4.902	252.9	H−4	→	L+1	36	0.0086	0	0
			H	→	L+5	27			
**28**	4.940	251.0	H−8	→	L	18	0.0088	0	0
			H−6	→	L	14			
**29**	4.952	250.4	H−3	→	L+2	21	0.0258	−0.0001	−0.0155
			H−5	→	L+1	17			
			H−4	→	L+1	12			
			H−6	→	L	10			
**30**	4.961	249.9	H−5	→	L+1	60	0.0279	−0.0002	−0.0287
			H−8	→	L	11			

Table S 5*:* Excitations calculated for **[11,0]** on PBE0/def2-TZVP level of theory. The excitation with the greatest oscillator strength (f_L) is highlighted in orange. The dissymmetry factor g is calculated based on the rotatory strength R and f.

**Exc. #**	E_{exc} **/ eV**	λ **/ nm**	**From**	→	**To**	**Contr. (%)**	f_L	R_L	$g_{calc.}$
**1**	3.449	359.5	H	→	L	84	0.3012	0.0022	0.0292
			H−1	→	L+1	10			
**2**	3.797	326.5	H−1	→	L	48	2.5896	−0.0096	−0.0148
			H	→	L+1	45			
**3**	3.970	312.3	H	→	L+1	49	0.0018	0	0
			H−1	→	L	45			
**4**	3.994	310.4	H−1	→	L+1	32	0.9651	0.0079	0.0327
			H	→	L+2	29			
			H−2	→	L	27			

**5**	4.134	299.9	H	→	L+2	63	0.0220	0.0003	0.0545
			H−1	→	L+1	19			
**6**	4.174	297.0	H−2	→	L	59	0.0519	0.0003	0.0231
			H−1	→	L+1	31			
**7**	4.271	290.3	H−1	→	L+2	75	0.1655	−0.0018	−0.0435
**8**	4.327	286.6	H−2	→	L+1	80	0.0956	−0.0009	−0.0377
**9**	4.409	281.2	H−3	→	L	55	0.0019	0	0
			H	→	L+3	29			
**10**	4.449	278.7	H−2	→	L+2	71	0.0388	0.0002	0.0206
**11**	4.469	277.4	H	→	L+7	27	0.0601	−0.0002	−0.0133
			H	→	L+3	18			
			H−3	→	L	15			
**12**	4.522	274.2	H	→	L+3	31	0.0816	0	0
			H	→	L+7	29			
**13**	4.547	272.7	H	→	L+9	16	0.0006	0	0
			H	→	L+4	13			
**14**	4.569	271.3	H−3	→	L+1	55	0.1065	0.0004	0.0150
			H−1	→	L+3	13			
**15**	4.586	270.4	H	→	L+8	17	0.0238	0.0002	0.0336
			H−3	→	L+1	13			
**16**	4.604	269.3	H	→	L+4	13	0.0080	0.0001	0.0500
**17**	4.618	268.5	H−3	→	L+2	13	0.0145	−0.0001	−0.0276
**18**	4.646	266.9	H	→	L+4	16	0.0013	0.0001	0.3077
**19**	4.656	266.3	H−4	→	L	18	0.0297	0	0
			H−1	→	L+3	11			
**20**	4.672	265.4	H−5	→	L	33	0.0417	0.0004	0.0384
			H−3	→	L+2	15			
**21**	4.683	264.8	H	→	L+13	11	0.0015	0.0001	0.2667
**22**	4.702	263.7	H−1	→	L+3	49	0.0537	−0.0009	−0.0670
			H−4	→	L	17			
**23**	4.718	262.8	H−4	→	L	19	0.0050	0.0002	0.1600
			H	→	L+4	16			
**24**	4.733	261.9	H	→	L+14	16	0.0125	−0.0002	−0.0640
			H−18	→	L	13			
**25**	4.744	261.3	H	→	L+16	23	0.0022	0	0
			H−20	→	L	17			
			H−1	→	L+14	10			
**26**	4.752	260.9	H−5	→	L	17	0.0015	−0.0001	−0.2667
**27**	4.779	259.4	H−6	→	L	44	0.0241	−0.0002	−0.0332
			H	→	L+4	15			
**29**	4.813	257.6	H−3	→	L+2	19	0.0175	0.0002	0.0457
			H−4	→	L+1	14			
			H−5	→	L	12			
**30**	4.829	256.7	H−4	→	L+1	27	0.0680	−0.0009	−0.0529
			H−2	→	L+3	18			
			H−6	→	L+1	12			
			H−3	→	L+2	11			

Table S 6: Excitations calculated for **[0,9]** on PBE0/def2-TZVP level of theory. The excitation with the greatest oscillator strength (f_L) is highlighted in purple. The dissymmetry factor g is calculated based on the rotatory strength R and f.

Exc. #	E_{exc} / eV	λ / nm	From	→	To	Contr. (%)	f_L	R_L	$g_{calc.}$
**1**	3.414	363.2	H	→	L	90	0.3455	−0.0016	−0.0185
**2**	3.839	323.0	H−1	→	L	59	2.0706	0.0002	0.0004
			H	→	L+1	37			
**3**	3.944	314.4	H	→	L+1	59	0.0309	0	0
			H−1	→	L	37			
**4**	4.114	301.4	H−1	→	L+1	65	0.3724	−0.0003	−0.0032
			H−2	→	L	17			
**5**	4.218	293.9	H−2	→	L	73	0.0292	0	0
			H	→	L+2	14			
**6**	4.251	291.7	H	→	L+2	71	0.2096	0.0001	0.0019
			H−1	→	L+1	16			
**7**	4.413	281.0	H	→	L+6	32	0.0553	−0.0001	−0.0072
			H	→	L+4	13			
			H	→	L+7	12			
**8**	4.459	278.1	H−1	→	L+2	60	0.2303	−0.0001	−0.0017
**9**	4.485	276.5	H−2	→	L+1	73	0.0399	0	0
			H−1	→	L+2	16			
**10**	4.500	275.5	H	→	L+7	22	0.0275	−0.0001	−0.0145
			H−1	→	L+6	13			
			H	→	L+6	10			
**11**	4.546	272.8	H	→	L+9	17	0.0317	0.0003	0.0379
**12**	4.575	271.0	H−3	→	L	66	0.0304	0.0002	0.0263
			H	→	L+4	11			
**14**	4.661	266.0	H	→	L+3	54	0.0054	0.0001	0.0741
**15**	4.671	265.4	H	→	L+3	17	0.0099	0.0001	0.0404
			H	→	L+12	12			
**16**	4.707	263.4	H	→	L+13	11	0.0004	0	0
			H−1	→	L+12	11			
			H−16	→	L	11			
**17**	4.738	261.7	H−16	→	L	11	0.0010	0	0
			H	→	L+15	11			
**18**	4.756	260.7	H	→	L+4	51	0.0447	0.0001	0.0089
			H−3	→	L	12			
**19**	4.767	260.1	H−2	→	L+2	64	0.0314	−0.0003	−0.0382
**20**	4.788	258.9	H−1	→	L+3	59	0.0252	−0.0002	−0.0317
**22**	4.847	255.8	H−3	→	L+1	71	0.0514	0.0001	0.0078
**23**	4.882	254.0	H	→	L+5	27	0.0294	0	0
			H−1	→	L+4	17			
**24**	4.913	252.4	H	→	L+5	33	0.0001	0	0
			H−1	→	L+3	15			
**25**	4.928	251.6	H−1	→	L+4	45	0.0815	0.0003	0.0147
**26**	4.964	249.8	H−4	→	L	33	0.0037	−0.0001	−0.1081
			H−1	→	L+4	22			
			H−5	→	L	11			

**27**	5.004	247.8	H−1	→	L+5	44	0.0006	0	0
			H	→	L+6	13			
**28**	5.022	246.9	H	→	L+6	21	0.0080	−0.0001	−0.050
			H−9	→	L	18			
			H−1	→	L+5	18			
**29**	5.028	246.6	H	→	L+7	35	0.0124	0	0
			H−10	→	L	23			
			H−1	→	L+6	13			
**30**	5.037	246.2	H−5	→	L	28	0.0060	−0.0002	−0.1333
			H−4	→	L	26			

Table S 7*:* Excitations calculated for **[0,11]** on PBE0/def2-TZVP level of theory. The excitation with the greatest oscillator strength (f_L) is highlighted in purple. The dissymmetry factor *g* is calculated based on the rotatory strength *R* and *f*.

**Exc. #**	**E_{exc} / eV**	**λ / nm**	**From**	→	**To**	**Contr. (%)**	**f_L**	**R_L**	**$g_{calc.}$**
**1**	3.470	357.3	H	→	L	84	0.4209	−0.0015	−0.0143
**1**	3.470	357.3	H−1	→	L+1	11	0.4209		
**2**	3.796	326.6	H−1	→	L	52	2.6850	−0.0004	−0.0006
			H	→	L+1	40			
**3**	3.978	311.7	H	→	L+1	53	0.0164	0	0
			H−1	→	L	41			
**4**	4.009	309.2	H−1	→	L+1	47	0.6801	0.0005	0.0029
			H−2	→	L	27			
			H	→	L+2	15			
**5**	4.167	297.6	H−2	→	L	67	0.0361	−0.0001	−0.0111
			H−1	→	L+1	17			
**6**	4.205	294.8	H	→	L+2	73	0.1269	0.0001	0.0032
			H−1	→	L+1	18			
**7**	4.300	288.3	H−2	→	L+1	48	0.3304	−0.0002	−0.0024
			H−1	→	L+2	30			
			H−3	→	L	10			
**8**	4.368	283.9	H−1	→	L+2	54	0.0073	0	0
			H−2	→	L+1	37			
**9**	4.411	281.1	H−3	→	L	71	0.0586	0	0
			H	→	L+3	16			
**10**	4.483	276.6	H	→	L+7	32	0.0450	0.0001	0.0089
			H	→	L+3	22			
**11**	4.505	275.2	H−2	→	L+2	42	0.0838	0.0001	0.0048
**12**	4.542	273.0	H	→	L+3	37	0.0869	0	0
			H	→	L+7	22			
**13**	4.565	271.6	H	→	L+9	19	0.0104	0	0
			H−2	→	L+2	15			
**14**	4.586	270.4	H−1	→	L+8	21	0.0106	−0.0002	−0.0755
			H	→	L+8	19			
**15**	4.614	268.7	H−3	→	L+1	65	0.0763	0.0002	0.0105
			H−2	→	L+2	18			

**16**	4.626	268.0	H	→	L+11	24	0.0011	0	0
			H−16	→	L	11			
			H	→	L+3	10			
**17**	4.645	266.9	H−1	→	L+3	13	0.0024	0	0
**18**	4.689	264.4	H−1	→	L+3	20	0.0146	0.0001	0.0274
**19**	4.694	264.1	H−1	→	L+3	25	0.0668	−0.0001	−0.0060
			H−4	→	L	15			
			H	→	L+4	13			
**20**	4.706	263.5	H	→	L+4	38	0.0066	0	0
			H−1	→	L+3	13			
**21**	4.724	262.5	H	→	L+18	10	0.0025	0.0001	0.1600
**22**	4.740	261.6	H	→	L+19	11	0.0012	−0.0001	−0.3333
**23**	4.758	260.6	H−4	→	L	14	0.0007	0	0
**24**	4.760	260.5	H−4	→	L	39	0.0005	0	0
			H−1	→	L+4	18			
**25**	4.772	259.8	H−1	→	L+4	39	0.0016	0.0001	0.2500
			H−3	→	L+2	10			
**26**	4.838	256.3	H−3	→	L+2	53	0.1267	−0.0004	−0.0126
			H−4	→	L+1	12			
**28**	4.885	253.8	H−2	→	L+3	46	0.0371	−0.0001	−0.0108
**29**	4.889	253.6	H−2	→	L+3	21	0.0145	0	0
**30**	4.914	252.3	H−4	→	L+1	26	0.0191	−0.0002	−0.0419
			H	→	L+5	19			
			H−1	→	L+5	11			

Table S 8*: Excitations calculated for* **[9,9]** on PBE0/def2-TZVP level of theory. The excitation with the greatest oscillator strength (f_L) is highlighted in orange. The dissymmetry factor *g* is calculated based on the rotatory strength *R* and *f*.

**Exc. #**	**E_{exc} / eV**	**λ / nm**	**From**	**→**	**To**	**Contr. (%)**	**f_L**	**R_L**	**$g_{calc.}$**
**1**	3.373	367.6	H−1	→	L	87	0.1846	0.0107	0.2319
**2**	3.426	361.8	H	→	L+1	86	0.3902	−0.0091	−0.0933
**3**	3.795	326.7	H−1	→	L+2	33	4.3358	−0.0122	−0.0113
			H−3	→	L	22			
			H−2	→	L+1	13			
			H	→	L+3	12			
			H−2	→	L	11			
**4**	3.833	323.4	H	→	L	94	0.0138	−0.0002	−0.0580
**5**	3.896	318.3	H−1	→	L+2	37	0.1809	−0.0001	−0.0022
			H−2	→	L+1	31			
			H	→	L+3	22			
**6**	3.930	315.5	H−3	→	L	51	0.0054	0.0001	0.0741
			H−1	→	L+2	25			
			H−2	→	L	11			
**7**	3.966	312.6	H	→	L+3	55	0.0213	−0.0002	−0.0376
			H−2	→	L+1	34			
**8**	3.979	311.6	H−1	→	L+1	87	0.0677	0	0

**9**	4.059	305.4	H−2	→	L	56	0.1014	−0.0002	−0.0079
			H−3	→	L	16			
**10**	4.077	304.1	H−1	→	L+4	55	0.5603	0.0039	0.0278
			H−3	→	L+2	12			
**11**	4.100	302.4	H	→	L+2	87	0.0073	0	0
**12**	4.134	299.9	H−3	→	L+2	33	0.0711	0.0039	0.2194
			H−1	→	L+4	26			
			H−5	→	L	15			
			H−2	→	L+2	12			
**13**	4.140	299.5	H−2	→	L+3	46	0.3313	0.0003	0.0036
			H	→	L+4	15			
			H−4	→	L+1	14			
**14**	4.204	294.9	H	→	L+4	56	0.0104	−0.0011	−0.4231
			H−2	→	L+3	16			
**15**	4.217	294.0	H−5	→	L	49	0.0460	0.0018	0.1565
			H−3	→	L+2	16			
			H−4	→	L	11			
**16**	4.246	292.0	H−1	→	L+3	72	0.0003	0	0
			H−3	→	L+1	19		0	0
**17**	4.261	291.0	H−4	→	L+1	50	0.0246	0.0025	0.4065
			H−2	→	L+3	15			
**18**	4.270	290.4	H−3	→	L+1	52	0.0027	0.0001	0.1481
			H−1	→	L+3	23			
**19**	4.309	287.7	H−2	→	L+2	54	0.0296	−0.0021	−0.2838
			H−3	→	L+2	21			
**20**	4.347	285.2	H−2	→	L+4	22	0.0751	−0.0006	−0.0320
			H−3	→	L+4	16			
			H−4	→	L	13			
			H	→	L+5	11			
**21**	4.352	284.9	H	→	L+5	19	0.0823	−0.0004	−0.0194
			H−2	→	L+4	18			
			H−4	→	L	15			
			H−3	→	L+4	11			
**22**	4.375	283.4	H−4	→	L	36	0.0232	0.0004	0.0690
			H	→	L+5	33			
			H	→	L+4	15			
**23**	4.398	281.9	H−1	→	L+10	46	0.0491	−0.0002	−0.0163
			H−17	→	L	10			
**24**	4.420	280.5	H	→	L+12	46	0.0395	−0.0001	−0.0101
**25**	4.428	280.0	H−3	→	L+4	31	0.0361	−0.0002	−0.0222
			H−2	→	L+4	24			
			H−5	→	L+2	13			
**26**	4.461	277.9	H−5	→	L+2	25	0.0766	−0.0009	−0.0470
			H−1	→	L+11	11			
**27**	4.467	277.5	H−1	→	L+11	18	0.0790	−0.0007	−0.0354
			H−5	→	L+2	13			
**28**	4.491	276.0	H−1	→	L+6	26	0.0255	−0.0004	−0.0627
			H−6	→	L	14			

			H−7	→	L	14			
**29**	4.509	275.0	H−3	→	L+3	36	0.0138	−0.0007	−0.2029
			H−5	→	L+1	21			
**30**	4.517	274.5	H	→	L+13	26	0.0362	0.0003	0.0331
			H−4	→	L+3	10			

Table S 9*:* Excitations calculated for **[11,9]** on PBE0/def2-TZVP level of theory. The excitation with the greatest oscillator strength (f_L) is highlighted in orange. The dissymmetry factor g is calculated based on the rotatory strength R and f.

Exc. #	E_{exc} / eV	λ / nm	From	→	To	Contr. (%)	f_L	R_L	$g_{calc.}$
**1**	3.425	362	H	→	L+1	83	0.2679	0.0264	0.3942
**2**	3.446	359.8	H−1	→	L	78	0.3883	−0.0244	−0.2514
**3**	3.769	328.9	H−1	→	L+2	37	4.6798	−0.0141	−0.0121
			H−3	→	L	22			
			H−2	→	L	17			
**4**	3.880	319.5	H	→	L	88	0.0342	−0.0003	−0.0351
**5**	3.884	319.2	H−2	→	L+1	35	0.5043	−0.0002	−0.0016
			H	→	L+4	30			
			H−1	→	L+2	12			
**6**	3.965	312.7	H−1	→	L+2	27	0.0040	0	0
			H	→	L+4	25			
			H−2	→	L	17			
			H−3	→	L	15			
**7**	3.967	312.5	H	→	L+4	31	0.0171	−0.0002	−0.0468
			H−2	→	L+1	25			
			H−1	→	L+2	17			
			H−3	→	L	16			
**8**	3.980	311.6	H−1	→	L+3	35	0.8738	0.0062	0.0284
			H−4	→	L	20			
			H−1	→	L+1	16			
			H−3	→	L+2	14			
**9**	4.032	307.5	H−1	→	L+1	72	0.0868	0.0012	0.0553
**10**	4.058	305.5	H	→	L+2	93	0.0004	0	0
**11**	4.112	301.5	H−2	→	L	40	0.0088	0.0008	0.3636
			H−3	→	L	22			
			H−1	→	L+3	13			
**12**	4.114	301.3	H−1	→	L+3	42	0.0186	−0.0014	−0.3011
			H−3	→	L+2	11			
**13**	4.130	300.2	H	→	L+3	43	0.3065	0.0031	0.0405
			H−2	→	L+4	20			
			H	→	L+5	11			
**14**	4.170	297.3	H	→	L+3	39	0.0552	0.0007	0.0507
			H−2	→	L+4	33			
			H−5	→	L+1	10			
**15**	4.183	296.4	H−4	→	L	60	0.0556	0.0008	0.0576
			H−3	→	L+2	16			
			H−2	→	L+2	13			

**16**	4.211	294.4	H−3	→	L+1	53	0.0046	−0.0001	−0.0870
			H−3	→	L+3	13			
			H−2	→	L+1	13			
**17**	4.260	291.1	H−5	→	L+1	51	0.0199	0.0014	0.2814
**18**	4.273	290.1	H−2	→	L+3	27	0.1508	−0.0014	−0.0371
			H−3	→	L+3	21			
			H−3	→	L+1	18			
**19**	4.283	289.5	H−2	→	L+2	48	0.0200	−0.0015	−0.3000
			H−3	→	L+2	31			
**20**	4.303	288.1	H−1	→	L+4	94	0.0007	0	0
**21**	4.326	286.6	H	→	L+5	59	0.1183	−0.0005	−0.0169
**22**	4.330	286.3	H−4	→	L+2	66	0.1325	−0.0003	−0.0091
**23**	4.371	283.6	H−3	→	L+3	41	0.0021	0	0
			H−2	→	L+3	33			
**24**	4.399	281.8	H−4	→	L+1	41	0.0019	−0.0003	−0.6316
			H−4	→	L+3	22			
			H−5	→	L	11			
**25**	4.406	281.4	H−5	→	L	58	0.0013	−0.0003	−0.9231
			H−4	→	L+1	17			
**26**	4.418	280.6	H−1	→	L+6	26	0.0384	−0.0001	−0.0104
			H	→	L+12	23			
			H−6	→	L	14			
**27**	4.420	280.5	H	→	L+12	31	0.0013	−0.0001	−0.3077
			H−1	→	L+6	20			
			H−6	→	L	12			
**28**	4.468	277.5	H−4	→	L+3	42	0.0284	0	0
			H−4	→	L+1	24		0	0
**29**	4.477	276.9	H−6	→	L	29	0.0280	−0.0001	−0.0143
			H−1	→	L+11	24			
**30**	4.488	276.2	H−3	→	L+4	73	0.0091	−0.0003	−0.1319
			H−2	→	L+4	15			

Table S 10: Excitations calculated for **[11,11]** on PBE0/def2-TZVP level of theory. The excitation with the greatest oscillator strength (f_L) is highlighted in orange. The dissymmetry factor g is calculated based on the rotatory strength R and f.

**Exc. #**	**E_{exc} / eV**	**λ / nm**	**From**	**→**	**To**	**Contr. (%)**	**f_L**	**R_L**	**$g_{calc.}$**
**1**	3.447	359.7	H−1	→	L	80	0.2271	0.0235	0.4139
**2**	3.473	357.0	H	→	L+1	81	0.5067	−0.0213	−0.1681
**3**	3.759	329.8	H−1	→	L+2	28	5.7362	−0.0155	−0.0108
			H−3	→	L	24			
			H−2	→	L+1	15			
			H	→	L+3	14			
**4**	3.840	322.8	H−2	→	L+1	31	0.1650	−0.0002	−0.0048
			H	→	L+3	26			
			H−1	→	L+2	20			
			H−3	→	L	16			
**5**	3.932	315.3	H	→	L	93	0.0414	−0.0004	−0.0386

**6**	3.967	312.5	H−1	→	L+2	46	0.0049	0	0
			H−3	→	L	40			
**7**	3.984	311.2	H−1	→	L+4	35	0.9258	0.0061	0.0264
			H−3	→	L+2	21			
			H−5	→	L	21			
**8**	3.987	311.0	H	→	L+3	51	0.0228	0.0006	0.1053
			H−2	→	L+1	38			
**9**	4.034	307.3	H−2	→	L+3	44	0.5414	0.0033	0.0244
			H−4	→	L+1	22			
			H	→	L+5	11			
**10**	4.048	306.3	H−1	→	L+1	80	0.0557	0.0013	0.0934
**11**	4.082	303.7	H−2	→	L	68	0.0072	0.0001	0.0556
**12**	4.113	301.5	H−1	→	L+4	51	0.0077	0.0005	0.2597
			H−3	→	L+2	18			
**13**	4.120	300.9	H	→	L+2	90	0.0003	0	0
**14**	4.180	296.6	H	→	L+4	36	0.0566	−0.0034	−0.2403
			H−5	→	L	19			
			H	→	L+5	11			
**15**	4.184	296.3	H−5	→	L	35	0.0209	0.003	0.5742
			H	→	L+4	19			
			H−3	→	L+2	15			
**16**	4.201	295.2	H−4	→	L+1	53	0.0379	0.0033	0.3483
			H−2	→	L+3	24			
**17**	4.217	294.0	H−3	→	L+1	34	0.0815	−0.0009	−0.0442
			H−3	→	L+4	28			
			H−2	→	L+4	13			
**18**	4.242	292.3	H−1	→	L+3	90	0.0266	−0.0002	−0.0301
**19**	4.254	291.5	H−2	→	L+2	56	0.0355	−0.0023	−0.2592
			H−3	→	L+2	12			
**20**	4.270	290.3	H−3	→	L+1	53	0.1889	−0.0013	−0.0275
			H−2	→	L+4	11			
**21**	4.287	289.2	H	→	L+5	31	0.0342	0	0
			H−2	→	L+2	21			
			H−4	→	L	19			
			H	→	L+4	11			
**22**	4.315	287.3	H−3	→	L+4	32	0.1352	−0.0003	−0.0089
			H−5	→	L+2	23			
**23**	4.317	287.2	H−4	→	L	44	0.0223	0.0001	0.0179
			H	→	L+4	18			
			H	→	L+5	17			
**24**	4.338	285.8	H−5	→	L+2	33	0.0203	−0.0004	−0.0788
			H−2	→	L+4	29			
**25**	4.366	284.0	H−4	→	L+3	53	0.0348	0	0
			H−2	→	L+4	14			
			H−5	→	L+2	12			
**26**	4.408	281.3	H−5	→	L+1	25	0.0011	−0.0003	−1.0909
			H−5	→	L+4	21			
			H−3	→	L+3	15			

**27**	4.415	280.8	H−1	→	L+6	35	0.0036	−0.0003	−0.3333
			H−7	→	L	18			
			H−6	→	L	16			
**28**	4.430	279.9	H−6	→	L+1	48	0.0430	−0.0001	−0.0093
			H	→	L+7	16			
**29**	4.436	279.5	H−3	→	L+3	60	0.0291	0.0004	0.0550
			H−5	→	L+4	13			
**30**	4.444	279.0	H−2	→	L+5	39	0.0218	−0.0003	−0.0550
			H−4	→	L+2	17			
			H−4		L+3	11			

10.6 Calculated Absorption Spectra

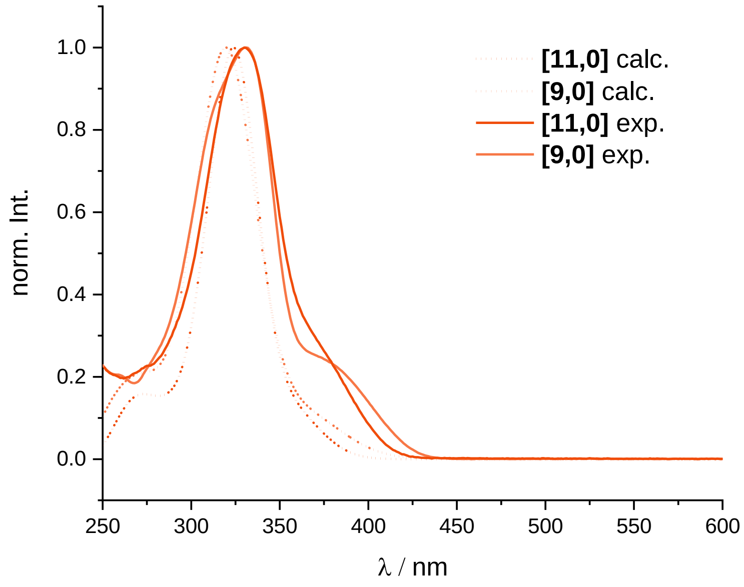

Figure S 103: Calculated (dashed line, PBE0/def2.TZVP//PBEh-3c level of theory) and experimental (solid) absorption spectra (normed) of **[11,0]** and **[9,0]**.

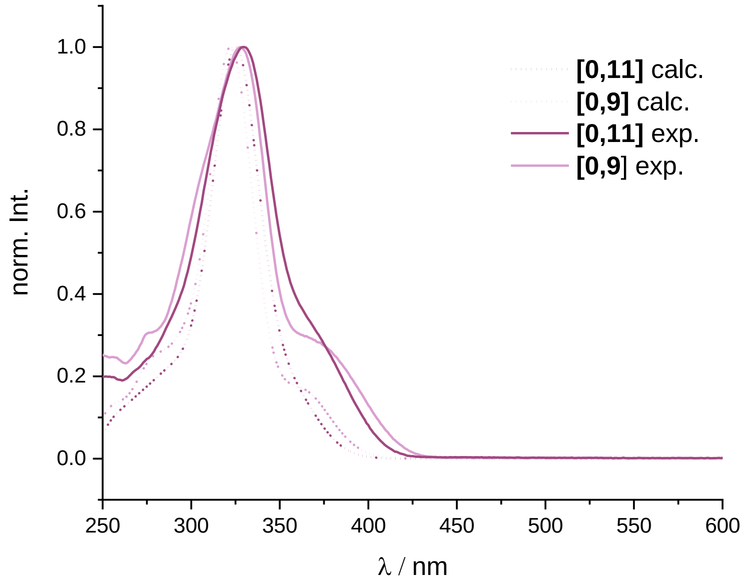

Figure S 104: Calculated (dashed line, PBE0/def2.TZVP//PBEh-3c level of theory) and experimental (solid) absorption spectra (normed) of **[0,11]** and **[0,9]**.

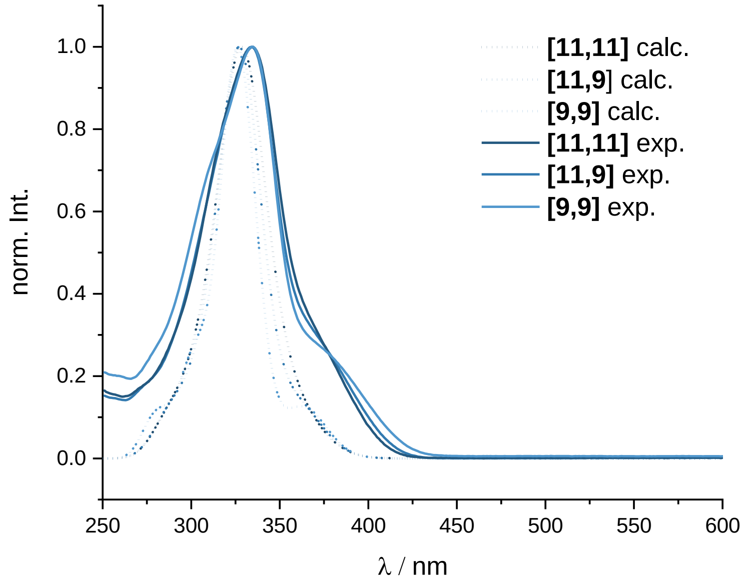

Figure S 105: Calculated (dashed line, PBE0/def2.TZVP//PBEh-3c level of theory) and experimental (solid) absorption spectra (normed) of **[9,9]**; **[11,9]** and **[11,11]**.

10.7 Calculated ECD Spectra

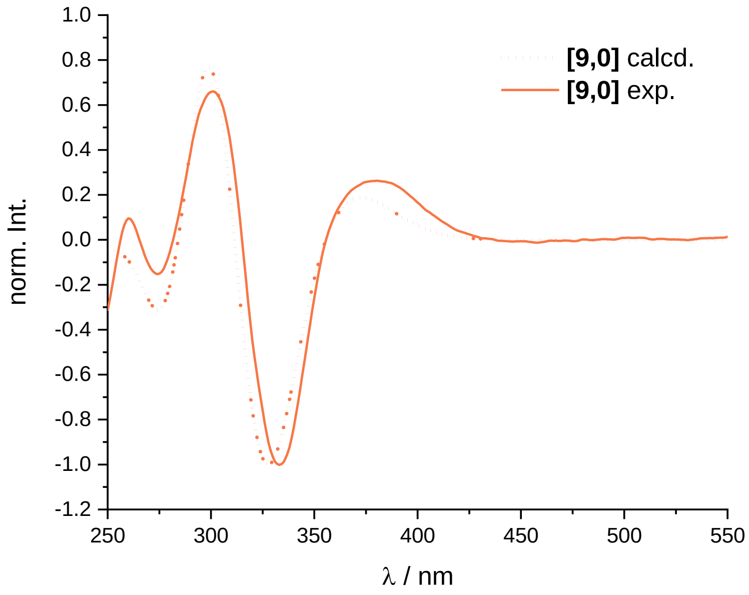

Figure S 106: Calculated (dashed line, PBE0/def2.TZVP//PBEh-3c level of theory) and experimental (solid) ECD spectra (normed) of (*R*,*R*,*R*,*R*)-**[9,0])**.

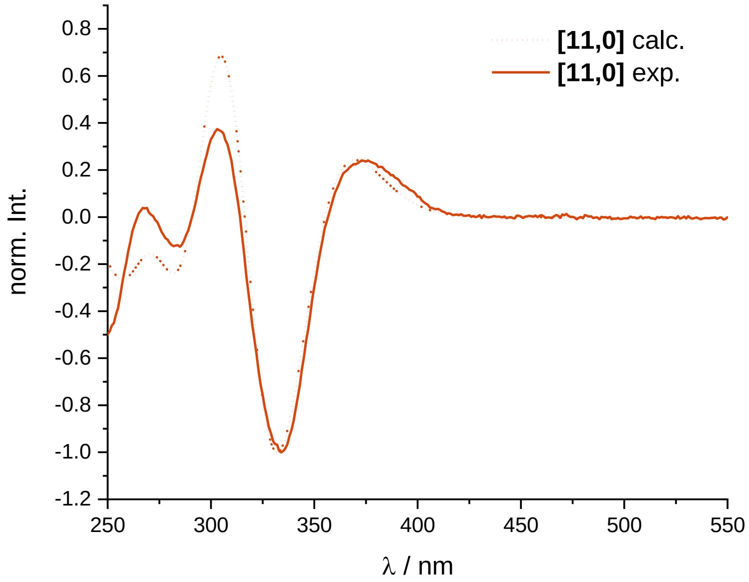

Figure S 107: Calculated (dashed line, PBE0/def2.TZVP//PBEh-3c level of theory) and experimental (solid) ECD spectra (normed) of (*R*,*R*,*R*,*R*)-**([11,0])**.

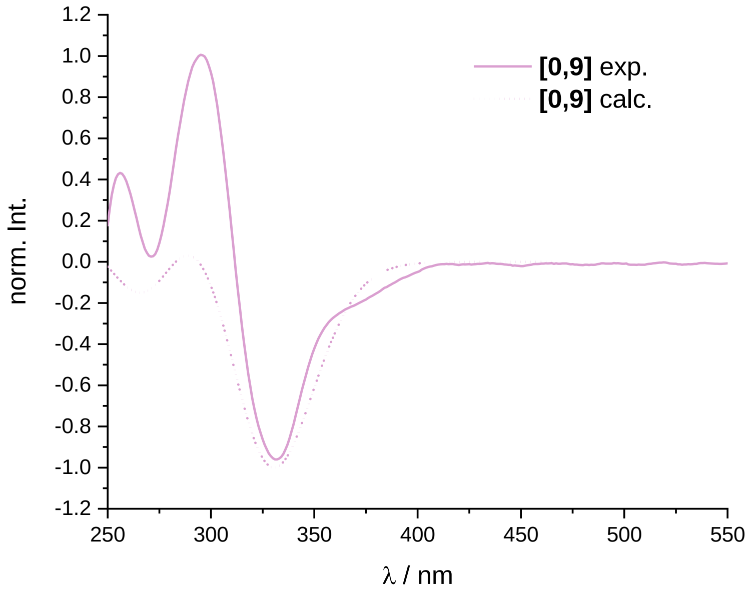

Figure S 108: Calculated (dashed line, PBE0/def2.TZVP//PBEh-3c level of theory, shifted by +0.33 eV) and experimental (solid) ECD spectra (normed) of (*R*,*R*,*R*,*R*)-**[0,11]**.

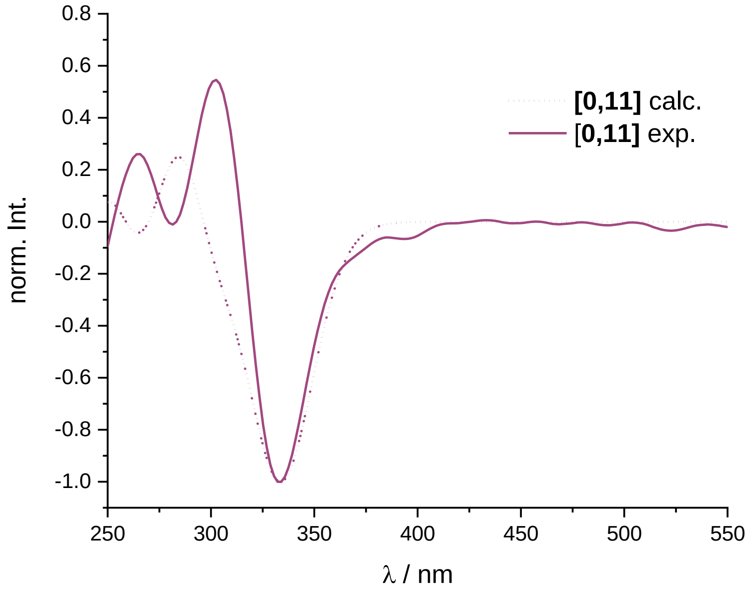

Figure S 109: Calculated (dashed line, PBE0/def2.TZVP//PBEh-3c level of theory, shifted by +0.24 eV) and experimental (solid) ECD spectra (normed) of (*R*,*R*,*R*,*R*)-**[0,11])**.

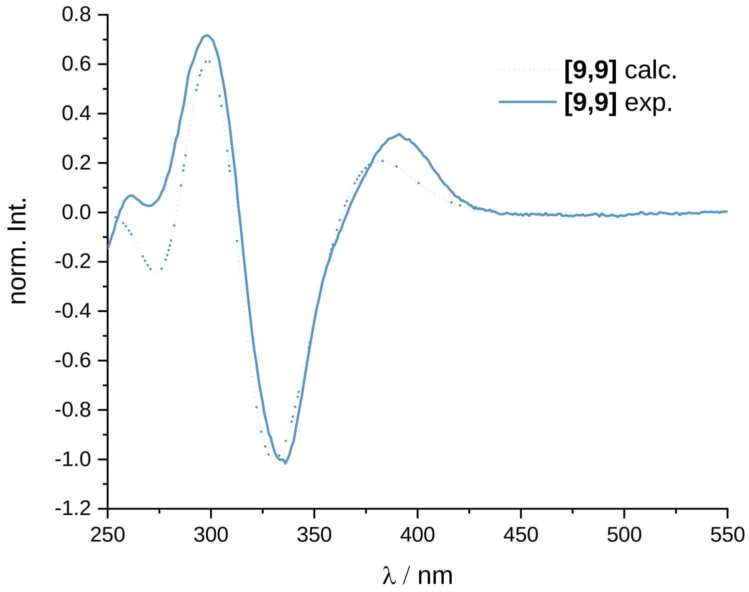

Figure S 110: Calculated (dashed line, PBE0/def2.TZVP//PBEh-3c level of theory) and experimental (solid) ECD spectra (normed) of (*R*,*R*,*R*,*R*)-**[9,9])**.

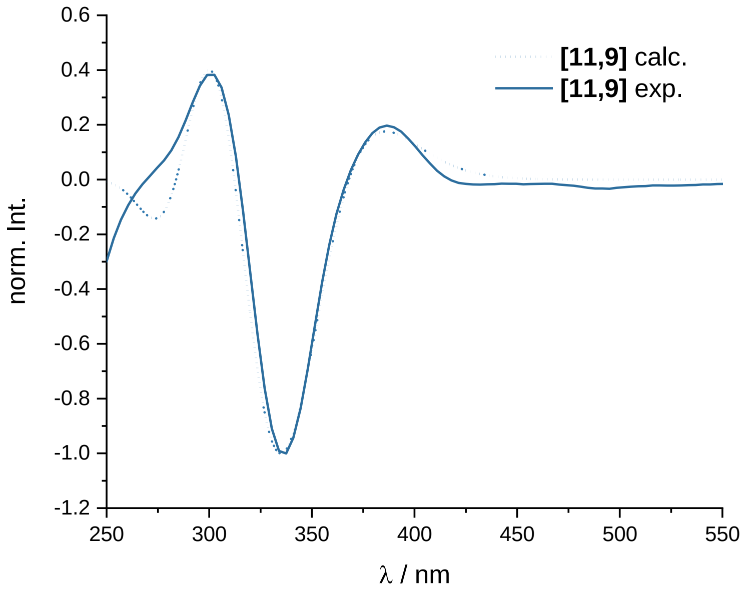

Figure S 111: Calculated (dashed line, PBE0/def2.TZVP//PBEh-3c level of theory) and experimental (solid) ECD spectra (normed) of (*R*,*R*,*R*,*R*)-**[11,9])**.

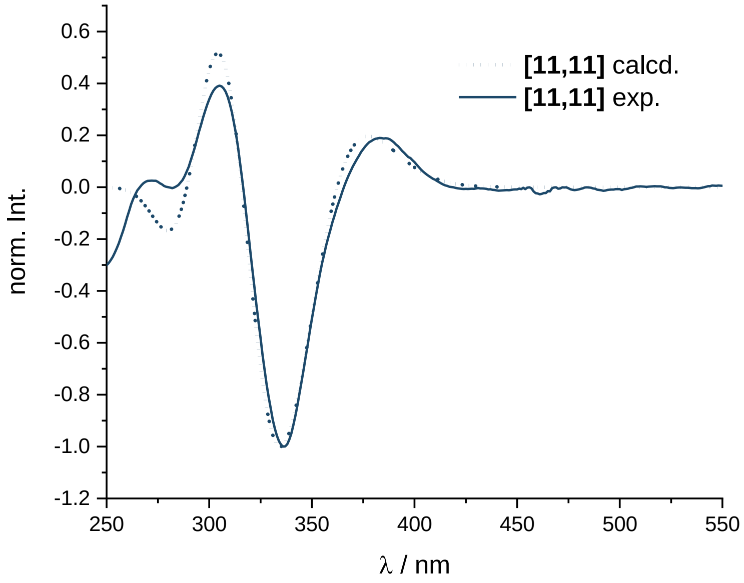

Figure S 112: Calculated (dashed line, PBE0/def2.TZVP//PBEh-3c level of theory) and experimental (solid) ECD spectra (normed) of (*R*,*R*,*R*,*R*)-**[11,11])**.

10.8 Calculations on the Tetrahydroindenoindene unit

To evaluate the bend of the tetrahydroindenoindene (THII) unit incorporated in the hoop structures, a simplified workflow was used (Figure S 113), in which Single Point energies (E_{SP}) on PW6B95/def2-QZVP level and bend angles were compared before (**[n,m]-THII-1**) and after geometry optimization with PBEh-3c (**[n,m]-THII-2**). Angles of the 2,7-connection (grey) and the 5,10-connection (blue) were determined between planes of six-memebered rings using MERCURY.

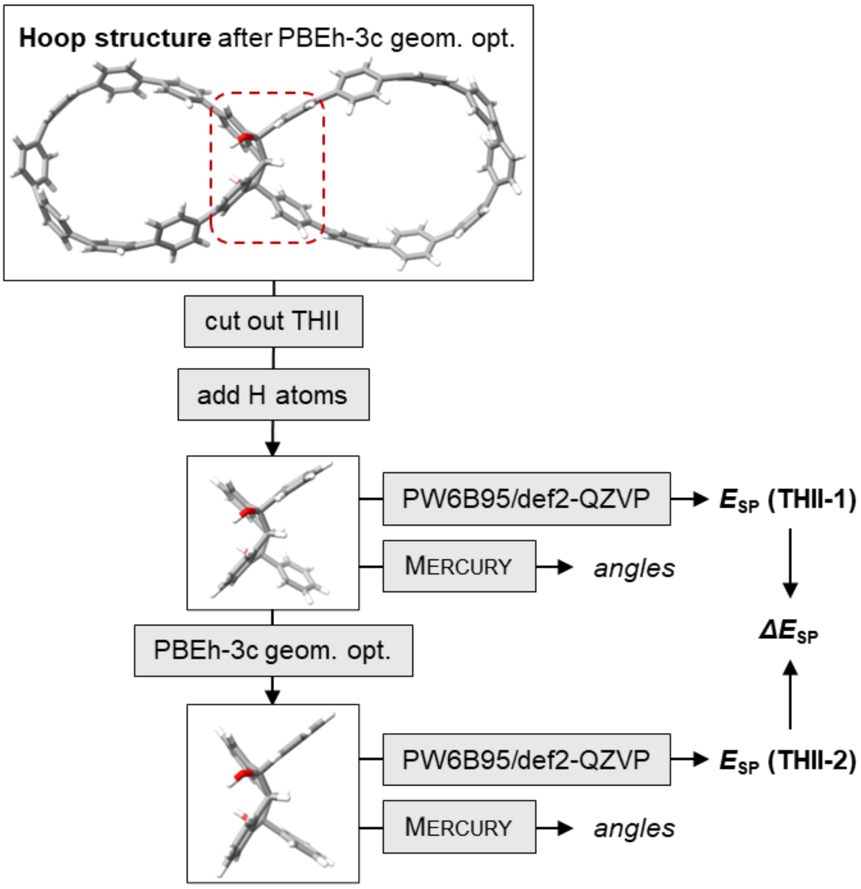

Figure S 113: Workflow for DFT calculations on the tetrahydroindenoindene unit shown on the example of **[9,9]**.

Table S 11: Calculated energies (PW6B95/def2-QZVP) and angles determined for tetrahydroindenoindene units before and after relaxation.

	$E_{SP,THII-1}$ / Hartrees	$E_{SP,THII-2}$ / Hartrees	∠THII-1 (2,7)	∠THII-2 (2,7)	∠THII-1 (5,10)	∠THII-2 (5,10)
**[9,0][a]**	−1311.6530862	−1311.6567924	87°	109°	70°	73°
**[11,0][a]**	−1311.6541972	−1311.6568908	94°	109°	70°	72°
**[0,9]**	−1232.8822275	−1232.8841500	110°	110°	60°	72°
**[0,11]**	−1232.8827904	−1232.8842041	112°	111°	63°	72°
**[9,9]**	−1232.8784574	−1232.8840319	110°	110°	75°	72°
**[11,9]**	−1232.8793089	−1232.8840219	109°	110°	72°	72°
**[11,11]**	−1232.8797983	−1232.8840798	109°	110°	72°	72°

[a] Note that these structures have tolyl groups in 5,10-position.

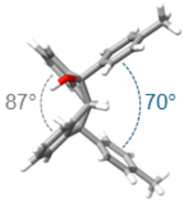

[9,0]-THII-1

$$\Delta E_{SP} = -2.33\,\frac{\text{kcal}}{\text{mol}}$$

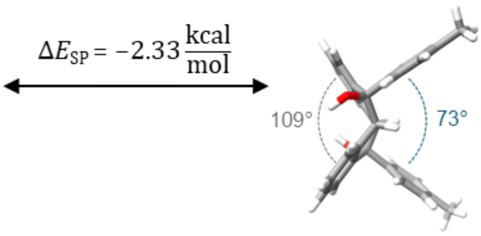

[9,0]-THII-2

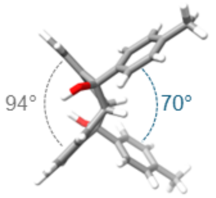

[11,0]-THII-1

$$\Delta E_{SP} = -1.69\,\frac{\text{kcal}}{\text{mol}}$$

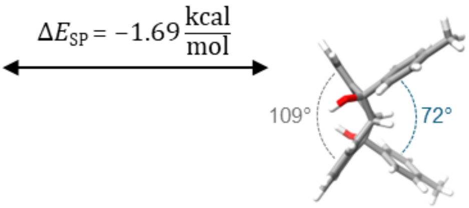

[11,0]-THII-2

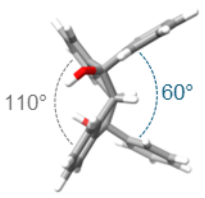

[0,9]-THII-1

$$\Delta E_{SP} = -1.21\,\frac{\text{kcal}}{\text{mol}}$$

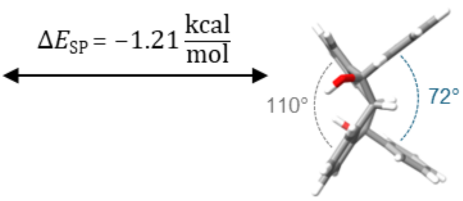

[0,9]-THII-2

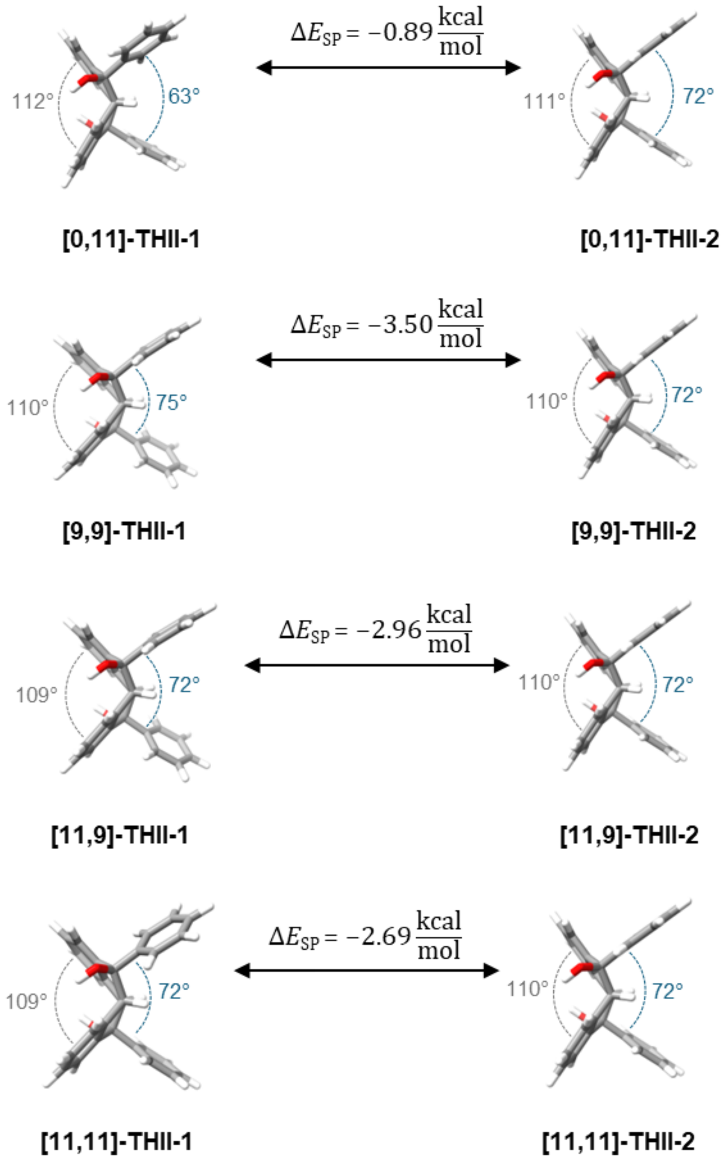

Figure S 114: Geometries, angles and energy differences of calc. **[n,m]-THII** units.

10.9 Coordinates of Optimized Geometries

Table S 12: Coordinates of the optimized geometry of **[9,0]** (PBEh-3c/def2-mSVP).

	x	y	z
C	0.643868	3.6401447	1.87857
C	1.7123259	2.7461874	1.97738
C	1.6818207	1.7943255	3.0008614
C	0.5864448	1.6579549	3.8400156
C	−0.5254034	2.4674892	3.6398039
C	−0.4536947	3.4871684	2.7050762
C	−1.8777697	2.4093717	4.2966089
C	−2.5946271	1.0189801	4.1241192
C	−3.8475164	0.2240251	2.0119237
C	−4.5638655	0.5724532	0.866082
C	−4.8500518	1.9252524	0.6555156
C	−4.3352773	2.9160587	1.4785144
C	−3.5135159	2.5579159	2.5425074
C	−3.349663	1.2150914	2.8362014
C	−2.7209395	3.4296332	3.4804699
C	−1.6824279	4.3595095	2.7466414
C	−4.81192	−0.1204852	−1.5038082
C	−4.8860999	−0.4479536	−0.1505052
C	−5.1187225	−1.7853921	0.1753054
C	−5.160755	−2.7645297	−0.8029287
C	−4.9810198	−2.4446451	−2.1486414
C	−4.8627225	−1.0958744	−2.4810765
C	4.9528663	1.9056241	0.2318231
C	4.5999595	2.0622636	−1.1081716
C	3.3685756	2.6534565	−1.3871539
C	2.4758391	2.9561134	−0.3761138
C	2.7792062	2.6905913	0.9584925
C	4.0626646	2.2204709	1.2440383
C	6.0248737	−0.1106637	−4.4767506
C	6.0721486	−0.6537126	−3.1926758
C	5.7449959	0.1030614	−2.0827323
C	5.3461471	1.4316904	−2.2131484
C	5.462257	2.0193617	−3.4733786
C	5.8044857	1.2648318	−4.5817376
C	−4.5039186	−4.0174465	−5.5299441
C	−5.1010937	−3.2974291	−4.5105599
C	−4.6912958	−3.4528674	−3.1853176
C	−3.7843127	−4.473299	−2.9068575
C	−3.1811703	−5.186604	−3.9267722
C	−3.4655111	−4.9126913	−5.2645806
C	5.925102	−3.2991472	−6.4553412
C	6.4447985	−2.310892	−5.6371639
C	5.9045228	−1.0221456	−5.6315949
C	4.9794297	−0.717057	−6.629293
C	4.4645175	−1.7014372	−7.4505171
C	4.8489286	−3.0340631	−7.3048506
C	−0.0350134	−5.5665901	−7.660701
C	−0.9014303	−4.5044856	−7.9188249
C	−2.1046593	−4.3859322	−7.2505839
C	−2.4927276	−5.3241536	−6.2951223
C	−1.7269381	−6.4881296	−6.1959616
C	−0.5222641	−6.608076	−6.8672333
C	3.5371077	−5.0597368	−6.8378628
C	2.3117172	−5.6934064	−6.9238488
C	1.4026362	−5.3656093	−7.9291867
C	1.8683039	−4.5594027	−8.9714088
C	3.0930776	−3.9214876	−8.8834368
C	3.8990354	−4.0713728	−7.7523427
O	−2.0787692	4.7886209	1.4737886
C	−1.4637487	5.6115798	3.5797646
O	−1.7373098	−0.08735	4.0824767
C	−3.5315897	0.7960673	5.3005173
C	−3.0367309	0.2327654	6.4763267
C	−3.8509607	0.0727091	7.5821303
C	−5.1875535	0.4667935	7.5613932
C	−5.674517	1.0266762	6.3896435
C	−4.8621423	1.1882524	5.274335
C	−0.4776927	5.6889798	4.5528802
C	−0.3449569	6.8229698	5.3438791
C	−1.1863713	7.9144388	5.1862481
C	−2.1737904	7.8325846	4.2064564
C	−2.3128971	6.7055397	3.4175578
C	−1.0382855	9.1494619	6.0254368
C	−6.0617073	0.2786522	8.7663478
H	0.6643787	4.4459092	1.1544647
H	2.5047177	1.0990927	3.1059168
H	0.5980788	0.9019167	4.6138977

**H**	−1.8148114	2.6741291	5.3544156
**H**	−3.632511	−0.8142239	2.2336337
**H**	−5.475273	2.2129902	−0.1804101
**H**	−4.5699175	3.9531682	1.2770156
**H**	−3.3790977	4.0272643	4.114903
**H**	−4.6169285	0.9017324	−1.8011212
**H**	−5.2444393	−2.0729857	1.2117006
**H**	−5.3084066	−3.7973667	−0.5120671
**H**	−4.7043921	−0.8121848	−3.5136686
**H**	5.9254497	1.502608	0.4865485
**H**	3.0587353	2.7805267	−2.4166386
**H**	1.4903352	3.3150647	−0.6431876
**H**	4.3641566	2.068663	2.2729354
**H**	6.2392982	−1.7149889	−3.0629419
**H**	5.6741543	−0.3845101	−1.118663
**H**	5.2314474	3.0703486	−3.5976044
**H**	5.8388705	1.7439357	−5.5523581
**H**	−4.809029	−3.8350089	−6.5530407
**H**	−5.8613477	−2.5652499	−4.7537542
**H**	−3.4470986	−4.6275402	−1.8896555
**H**	−2.3830415	−5.8738566	−3.6775767
**H**	6.3121337	−4.3077494	−6.3754457
**H**	7.2332301	−2.5679373	−4.9404864
**H**	4.540366	0.2708729	−6.6743545
**H**	3.6406307	−1.4495343	−8.1054724
**H**	−0.5707723	−3.6781705	−8.5342982
**H**	−2.6734625	−3.4731245	−7.3704883
**H**	−2.0399185	−7.2831995	−5.5302546
**H**	0.0815556	−7.4933819	−6.7094294
**H**	4.142049	−5.2218786	−5.9551434
**H**	2.0021191	−6.3287143	−6.1042505
**H**	1.229816	−4.3568643	−9.8226395
**H**	3.3858855	−3.2311092	−9.6651746
**H**	−2.2111569	4.0107575	0.9215784
**H**	−1.1536853	0.0104184	3.32249
**H**	−2.0051369	−0.0879357	6.530782
**H**	−3.439262	−0.3707747	8.4815753
**H**	−6.7101332	1.3407347	6.3364406
**H**	−5.2885589	1.6191698	4.377883
**H**	0.2094645	4.866371	4.7021814
**H**	0.4367764	6.8535468	6.0934821
**H**	−2.8466606	8.6690829	4.0558665
**H**	−3.0904855	6.6809663	2.6661743
**H**	−0.7135908	10.0003218	5.4239475
**H**	−1.9823055	9.429103	6.4949459
**H**	−0.3043654	9.0105958	6.8182158
**H**	−6.1888311	−0.7790256	9.0031798
**H**	−5.6332643	0.7564409	9.648533
**H**	−7.0537711	0.7012355	8.612514

Table S 13: Coordinates of the optimized geometry of **[11,0]** (PBEh-3c/def2-mSVP).

	x	y	z
**C**	4.9937094	−3.1865442	−0.2324173
**C**	4.3932716	−3.6957508	0.9200671
**C**	4.8469645	−3.2365892	2.1601257
**C**	5.8072482	−2.2420625	2.2635581
**C**	6.313366	−1.6649589	1.1045497
**C**	5.9402569	−2.1846697	−0.1237003
**C**	7.2579074	−0.5038837	0.9527321
**C**	6.7429825	0.8208049	1.6279502
**C**	5.0509034	2.4857996	0.6183286
**C**	4.470839	3.0254486	−0.5297734
**C**	4.9309708	2.5802961	−1.7732462
**C**	5.8590432	1.5557451	−1.8828429
**C**	6.3458545	0.9520759	−0.727675
**C**	5.9850576	1.4739095	0.5025418
**C**	7.2598719	−0.2348915	−0.5782952
**C**	6.7106879	−1.5480997	−1.2519166
**C**	2.2882671	3.8962772	−1.338529
**C**	3.339291	3.9665184	−0.4249951
**C**	3.2149929	4.8667616	0.6341347
**C**	2.0562337	5.601933	0.8168625
**C**	0.9781351	5.473751	−0.058093
**C**	1.1348768	4.6368614	−1.1617349
**C**	1.0940419	−5.1631916	−0.1201755
**C**	0.806913	−6.0468285	0.9188976
**C**	1.7975143	−6.2676267	1.8749158
**C**	2.9912027	−5.5683927	1.8388019
**C**	3.2388354	−4.6122229	0.8527917
**C**	2.2829339	−4.4580713	−0.15021
**C**	−3.3437803	−7.0020775	1.2931495
**C**	−2.5668913	−6.6917001	2.4089933
**C**	−1.2041459	−6.4854763	2.2977488

C	−0.5630903	−6.5717493	1.0635718
C	−1.316929	−6.9976957	−0.0299711
C	−2.6786394	−7.2151047	0.0844147
C	−2.5015827	6.8255771	−0.561558
C	−1.1644869	6.5547963	−0.7906734
C	−0.3491193	6.0506014	0.2228017
C	−0.8952169	5.9423777	1.5000704
C	−2.234308	6.2046655	1.726612
C	−3.0794403	6.592911	0.6874641
C	−9.4806109	−3.2297538	3.237481
C	−8.6663491	−4.3420711	3.1345267
C	−8.6335894	−5.1019093	1.9665875
C	−9.5818651	−4.813368	0.9837541
C	−10.4018757	−3.703907	1.0902665
C	−10.2986191	−2.8354874	2.1791667
C	−10.1626617	2.7639575	2.0646583
C	−10.2700957	3.6083708	0.9579327
C	−9.4039464	4.6741871	0.7895754
C	−8.3987401	4.9374984	1.7221575
C	−8.4165172	4.2070268	2.9091488
C	−9.2809754	3.1413223	3.0768066
C	−11.0436357	−0.6853449	3.2660025
C	−11.0086337	0.6980613	3.2393921
C	−10.689417	1.3860169	2.0670789
C	−10.5953106	0.640687	0.8926187
C	−10.6272497	−0.7403445	0.9199766
C	−10.757002	−1.4342029	2.1224661
O	5.9181807	−1.3352087	−2.387105
C	7.883831	−2.4072261	−1.6934091
O	5.952784	0.6329417	2.7687199
C	7.9385448	1.6543799	2.0602047
C	8.536441	2.5804449	1.2169997
C	9.6683825	3.2797048	1.6150002
C	10.2357541	3.0814964	2.8654002
C	9.6313986	2.1514897	3.7085085
C	8.5055598	1.4499977	3.3172812
C	8.4442364	−2.2187545	−2.9558707
C	9.5520245	−2.9447345	−3.3538465
C	10.1442908	−3.8838581	−2.5122754
C	9.5834347	−4.0661423	−1.2565276
C	8.4694144	−3.342515	−0.851857
C	11.3353161	−4.6768986	−2.963818
C	11.4457445	3.8494098	3.3099251
C	−7.4574101	−5.9541318	1.7107196
C	−6.8198924	−6.6876385	2.7119512

C	−5.5239485	−7.1441869	2.5446367
C	−4.8114438	−6.879317	1.3733352
C	−5.5038702	−6.2772208	0.3237035
C	−6.8000189	−5.8269215	0.4877909
C	−6.63229	5.5797949	0.1251561
C	−5.3315009	5.9721187	−0.1261341
C	−4.5407433	6.5315365	0.8764692
C	−5.1554365	6.8147684	2.0974114
C	−6.4580042	6.4202305	2.3500917
C	−7.19841	5.7278626	1.3907848
H	4.7266813	−3.5728039	−1.2089233
H	4.4006568	−3.6254368	3.066479
H	6.129295	−1.910382	3.2417389
H	8.2512809	−0.757159	1.3297262
H	4.7450839	2.8229537	1.6013227
H	4.5504362	3.0406211	−2.6764425
H	6.189749	1.2366921	−2.8625754
H	8.2586981	−0.0081912	−0.9577326
H	2.3361533	3.194388	−2.161136
H	4.0317683	4.9894726	1.3344045
H	1.9849386	6.2786592	1.6594436
H	0.3092136	4.4979247	−1.8480485
H	0.3404683	−4.9576114	−0.8698539
H	1.6268627	−6.9865514	2.6668026
H	3.7367292	−5.7616277	2.5999959
H	2.4318969	−3.7171181	−0.9251381
H	−3.0453846	−6.5062705	3.3619643
H	−0.6499808	−6.1514777	3.165883
H	−0.8360836	−7.1344193	−0.9907842
H	−3.2380361	−7.5220032	−0.7906024
H	−3.1132464	7.1912539	−1.3770302
H	−0.7531313	6.7108309	−1.7803782
H	−0.2916304	5.5494107	2.3085337
H	−2.6469269	6.0036073	2.706963
H	−9.3731666	−2.5874779	4.101923
H	−7.9498709	−4.5385676	3.9218708
H	−9.6390707	−5.4307498	0.0955888
H	−11.0873452	−3.4760882	0.2832043
H	−10.9993103	3.3933482	0.1863929
H	−9.4726149	5.2739983	−0.1096955
H	−7.6566466	4.3837744	3.6595474
H	−9.1696478	2.5147781	3.952365
H	−11.2332031	−1.1892033	4.2058638
H	−11.1695116	1.247902	4.1586588
H	−10.3531276	1.1353913	−0.0390371

**H**	−10.4082525	−1.2806328	0.0082319
**H**	5.1585699	−0.8026625	−2.1286791
**H**	5.1686136	0.1344442	2.5154432
**H**	8.121329	2.7763675	0.2368667
**H**	10.1105487	3.9972409	0.9340801
**H**	10.0490426	1.9732111	4.692846
**H**	8.0647102	0.7376479	4.0013467
**H**	8.0121821	−1.5000446	−3.6388374
**H**	9.9645924	−2.7790684	−4.3425203
**H**	10.0160275	−4.7908568	−0.5770905
**H**	8.0573595	−3.527174	0.1317608
**H**	11.0652256	−5.3822349	−3.7517981
**H**	11.7687847	−5.2512935	−2.1461183
**H**	12.1174562	−4.0309378	−3.3645698
**H**	11.1911648	4.57426	4.0853639
**H**	11.8978746	4.399098	2.4853632
**H**	12.2085479	3.1894169	3.7246235
**H**	−7.3262407	−6.8645174	3.6530081
**H**	−5.0445429	−7.6765633	3.3568247
**H**	−4.9885495	−6.0493417	−0.6003336
**H**	−7.2604638	−5.2591476	−0.310528
**H**	−7.1715777	5.0412356	−0.643371
**H**	−4.8920631	5.7312946	−1.0854921
**H**	−4.594814	7.3139676	2.8781383
**H**	−6.8891788	6.614128	3.3246161

Table S 14: Coordinates of the optimized geometry of **[0,9]** (PBEh-3c/def2-mSVP).

	x	y	z
**C**	5.1341777	0.3588005	0.768148
**C**	4.8992064	0.9182832	2.0227899
**C**	5.1677899	2.2774875	2.1853807
**C**	5.5621763	3.0613519	1.1149422
**C**	5.6999799	2.5187444	−0.1630661
**C**	5.5244973	1.1422661	−0.3018959
**C**	−5.7991219	0.6319538	−0.6854828
**C**	−5.4825592	0.0987389	−1.9353875
**C**	−4.8688393	−1.1539098	−1.9581592
**C**	−4.4911729	−1.7898597	−0.7901034
**C**	−4.6915828	−1.194379	0.4550334
**C**	−5.404082	0.004849	0.48215
**C**	−5.0895384	2.3780333	−5.5254556
**C**	−5.4729037	1.0379476	−5.5888077
**C**	−5.7210936	0.312671	−4.4357415
**C**	−5.587024	0.8942149	−3.1729636
**C**	−5.3466821	2.2669239	−3.1239861
**C**	−5.107354	2.9931901	−4.2741976
**C**	4.8983807	5.1672047	−2.6783768
**C**	5.0853478	4.5364874	−1.4626824
**C**	5.8217722	3.3568232	−1.3701851
**C**	6.488284	2.9223001	−2.5174215
**C**	6.3009848	3.5532339	−3.7348581
**C**	5.439234	4.6446801	−3.852875
**C**	−2.4311204	3.0856901	−8.0182404
**C**	−3.4213775	2.3977675	−7.3418044
**C**	−4.4072396	3.0783786	−6.6298629
**C**	−4.4850448	4.4618731	−6.802348
**C**	−3.4981175	5.1495628	−7.485902
**C**	−2.3914564	4.4799039	−8.013506
**C**	2.9595754	5.6296662	−7.1438304
**C**	3.4970094	6.6536383	−6.360342
**C**	4.4305091	6.380706	−5.3759416
**C**	4.8670522	5.0749801	−5.1425279
**C**	4.4850036	4.0983013	−6.0606532
**C**	3.5489936	4.3699204	−7.0406139
**C**	−0.0982638	4.6197905	−9.0597732
**C**	1.2380978	4.9067554	−8.8418258
**C**	1.6327733	5.7277154	−7.7825683
**C**	0.6292042	6.4187907	−7.104801
**C**	−0.7045548	6.1320223	−7.3220571
**C**	−1.0899249	5.1426845	−8.226172
**C**	1.8474183	−1.3275823	7.9991825
**C**	1.120651	−0.6112939	8.9371683
**C**	−0.2013196	−0.2617025	8.6803427
**C**	−0.8201765	−0.6430475	7.498421
**C**	−0.1026832	−1.395186	6.5725909
**C**	1.2246561	−1.7046163	6.8198223
**C**	−0.5450106	−1.8952016	5.2240905
**C**	−1.8255038	−2.8142367	5.2337106
**C**	−1.9249382	−5.3924276	5.3196327
**C**	−1.2380859	−6.5862501	5.1664469
**C**	0.1153069	−6.5792463	4.8437916
**C**	0.8027805	−5.3835715	4.6937254
**C**	0.1224325	−4.1841145	4.8848556

C	−1.2329563	−4.2010708	5.1685606
C	0.6406147	−2.7783902	4.7495526
C	1.8736496	−2.4156769	5.6603444
O	2.6384276	−3.5131865	6.0731152
O	−2.6510204	−2.6482165	6.3506999
C	−2.8141083	−2.35427	1.5981627
C	−4.0618435	−1.7331492	1.6749193
C	−4.6134884	−1.563848	2.9420854
C	−3.9269871	−1.9411211	4.0877092
C	−2.664765	−2.5170299	4.0030101
C	−2.1369695	−2.7476313	2.735712
C	3.8661395	−2.0260919	4.1497037
C	2.7919161	−1.5033369	4.8650339
C	2.5069818	−0.1499594	4.7231363
C	3.2283169	0.6412036	3.8443429
C	4.2648103	0.1099136	3.0802996
C	4.588551	−1.2316843	3.2753512
H	−0.7090806	−1.04716	4.5554922
H	0.8962013	−2.5816659	3.7058686
H	4.9244817	−0.6902927	0.6021201
H	5.0516679	2.7329339	3.1610599
H	5.7445477	4.1172435	1.2726372
H	5.6034503	0.6882905	−1.2814094
H	−6.3222834	1.5776284	−0.6195182
H	−4.6112696	−1.6101445	−2.9053494
H	−3.9930299	−2.7488556	−0.8544057
H	−5.595804	0.4954007	1.4279332
H	−5.5328876	0.5411056	−6.5495097
H	−5.9813574	−0.7351125	−4.5208279
H	−5.2284532	2.7600461	−2.1681008
H	−4.8069155	4.0290668	−4.1833391
H	4.2194273	6.0086117	−2.7296685
H	4.5517508	4.9058093	−0.5962694
H	7.1297888	2.0510878	−2.4674679
H	6.7943823	3.1602958	−4.6154186
H	−1.5990364	2.5257294	−8.4248207
H	−3.3356669	1.3229579	−7.245209
H	−5.2894097	5.0164962	−6.3346246
H	−3.5505766	6.2300697	−7.5405701
H	3.1273182	7.6659109	−6.4694514
H	4.7733636	7.1842666	−4.7356128
H	4.809562	3.0750024	−5.9221606
H	3.1683695	3.5484361	−7.6334658
H	−0.3649953	3.9160227	−9.8388141
H	1.9874023	4.4211358	−9.4550294
H	0.8879152	7.0761666	−6.2850528
H	−1.4400925	6.5756334	−6.6639046
H	2.8867668	−1.5758983	8.1734722
H	1.5863611	−0.3069333	9.8652837
H	−0.7528999	0.3181698	9.4089002
H	−1.8426501	−0.3494597	7.3026526
H	−2.9857054	−5.3866284	5.5366813
H	−1.7583965	−7.5281935	5.2800669
H	0.6371369	−7.5168906	4.70326
H	1.8499162	−5.391794	4.4223156
H	2.0686903	−4.1142019	6.5638982
H	−2.1444251	−2.8646889	7.1397121
H	−2.3383154	−2.4923086	0.6360085
H	−5.5981558	−1.1249847	3.0460043
H	−4.3833349	−1.7701281	5.0518584
H	−1.1701918	−3.2228441	2.62461
H	4.1363975	−3.0668991	4.2595451
H	1.6949464	0.2974162	5.2825052
H	2.9422121	1.677303	3.7137782
H	5.4173346	−1.666824	2.7303286

Table S 15: Coordinates of the optimized geometry of **[0,11]** (PBEh-3c/def2-mSVP).

	x	y	z
C	1.8564745	5.2181941	−3.887293
C	2.6912837	5.3233014	−2.7748146
C	2.1728134	5.9451308	−1.6394162
C	0.8549945	6.3605821	−1.5882704
C	−0.0012232	6.1864234	−2.6754009
C	0.5422248	5.6464013	−3.8409816
C	2.7643299	−4.1804478	3.1332317
C	2.1230532	−5.2145185	2.4522359
C	2.7133211	−5.6799001	1.2772815
C	3.8579211	−5.0899035	0.7701173
C	4.4605115	−4.011872	1.4181388
C	3.9089917	−3.592532	2.6275725
C	−1.8701613	−6.0917506	3.7262699
C	−1.5205902	−6.1106896	2.3766119
C	−0.2135246	−5.9084608	1.9736359

C	0.7992587	−5.6799779	2.9035749
C	0.4673486	−5.7678869	4.2561666
C	−0.8408797	−5.9688233	4.6597307
C	−3.3115547	7.4762546	−1.3939174
C	−1.9615471	7.4206268	−1.6954311
C	−1.4484111	6.4285302	−2.5326595
C	−2.358726	5.5620833	−3.1367316
C	−3.7075323	5.6225081	−2.842925
C	−4.2051073	6.5470127	−1.9257198
C	−5.0165576	−4.7058853	5.19545
C	−3.6878669	−5.058955	5.051414
C	−3.2927436	−5.9995414	4.1018858
C	−4.2958981	−6.6751994	3.4057116
C	−5.6264661	−6.324751	3.5531374
C	−6.005926	−5.2808659	4.3985421
C	−7.9771156	5.3636022	−0.3306688
C	−6.9788543	5.8873126	0.4903305
C	−5.8039987	6.3860715	−0.0418808
C	−5.5778831	6.3748025	−1.4167521
C	−6.6342524	5.9894353	−2.2430703
C	−7.8127078	5.4980261	−1.7104438
C	−7.8734068	−4.4167124	3.0314377
C	−8.8590787	−3.4702887	2.8254961
C	−9.3280335	−2.6790095	3.8734512
C	−8.8949471	−2.9962977	5.1620845
C	−7.908158	−3.9445252	5.3681744
C	−7.3160437	−4.6118655	4.2941253
C	9.3877615	4.1015703	−1.5025225
C	10.1124843	4.337889	−0.3454282
C	10.1005578	3.4092483	0.6908781
C	9.3857517	2.2261347	0.57479
C	8.6867351	1.9715858	−0.602043
C	8.6743814	2.9181067	−1.6123473
C	7.8303502	0.7840313	−0.9452171
C	8.5557514	−0.6136238	−0.8822007
C	9.6597009	−1.9176579	−2.8147399
C	9.7891026	−2.0702823	−4.1860491
C	9.1201123	−1.2112078	−5.0526213
C	8.3357599	−0.1772421	−4.5623126
C	8.2346002	−0.0011944	−3.1850579
C	8.8728989	−0.8842394	−2.3305213
C	7.4331271	1.0219281	−2.4276679
C	7.7802939	2.5227106	−2.7628231
O	8.4052811	2.7101562	−4.0002258
O	9.7005803	−0.6406326	−0.076923

C	5.5923057	−2.9774754	−0.5448688
C	5.5826599	−3.2680032	0.8175082
C	6.6223927	−2.7602479	1.5939793
C	7.6054324	−1.9553238	1.0429824
C	7.583913	−1.6315315	−0.3105965
C	6.5765357	−2.1765407	−1.0993645
C	6.041028	3.9823248	−3.9172781
C	6.5084628	3.3526945	−2.7700787
C	5.7407548	3.4494145	−1.6125261
C	4.52607	4.1067098	−1.6141736
C	4.0338587	4.7141845	−2.7702746
C	4.8273782	4.6551397	−3.9123549
H	6.9544808	0.7621842	−0.2927779
H	6.3674039	0.848517	−2.5923997
H	2.2216046	4.7450149	−4.7899714
H	2.801542	6.0862571	−0.7694161
H	0.4728093	6.7745975	−0.6639038
H	−0.0780822	5.5319175	−4.721046
H	2.320855	−3.7770219	4.0346705
H	2.2691041	−6.5134744	0.7474473
H	4.2901892	−5.4730743	−0.1459076
H	4.3394767	−2.7456077	3.1468107
H	−2.2953021	−6.1877991	1.6244142
H	0.0035925	−5.835657	0.9155441
H	1.2408597	−5.6568917	5.0059627
H	−1.0688796	−6.0066979	5.7178569
H	−1.2935126	8.1504262	−1.2551575
H	−2.0006002	4.7673955	−3.7782936
H	−4.3698062	4.8748251	−3.2604371
H	−5.2692648	−3.8874784	5.8571863
H	−2.9377792	−4.5112739	5.6076128
H	−4.0292728	−7.4608368	2.7094583
H	−6.3766762	−6.8423121	2.9680128
H	−7.4446387	−4.915674	2.1719563
H	−9.1696293	−3.2559418	1.8110775
H	−9.2924281	−2.4544349	6.0115626
H	−7.5549909	−4.128206	6.3754765
H	9.3628499	4.8328156	−2.3006643
H	10.676165	5.2552923	−0.2380645
H	10.6513638	3.6139447	1.5996805
H	9.3696966	1.5203444	1.3944412
H	10.148624	−2.600355	−2.1312461
H	10.3981247	−2.8700995	−4.5864303
H	9.2096432	−1.352905	−6.1218125
H	7.8080196	0.474452	−5.2455095

**H**	9.2318195	2.2174182	−4.0034566
**H**	10.3224859	0.0153033	−0.4079535
**H**	4.794567	−3.3455606	−1.1777901
**H**	6.6686639	−3.0006646	2.6490731
**H**	8.3892376	−1.5719417	1.6808045
**H**	6.5403853	−1.9637241	−2.1604873
**H**	6.6182146	3.9493596	−4.829784
**H**	6.0823438	2.9940366	−0.6910262
**H**	3.9338336	4.1126371	−0.7081696
**H**	4.5000919	5.1472697	−4.8199011
**C**	−9.0015787	4.4818112	0.2594013
**C**	−9.3043322	3.282131	−0.383006
**C**	−9.9240196	2.2477942	0.2915441
**C**	−10.2660014	2.368668	1.6378121
**C**	−10.1211111	3.6259793	2.2269864
**C**	−9.5013718	4.6624181	1.5505831
**C**	−10.4705778	1.1358232	2.4213284
**C**	−9.8033215	1.001167	3.6379608
**C**	−9.5714263	−0.2449327	4.1892739
**C**	−9.9974594	−1.4061149	3.5460273
**C**	−10.8277295	−1.2566518	2.4334113
**C**	−11.0600371	−0.0091834	1.8816055
**H**	−3.6727707	8.2412144	−0.7174777
**H**	−7.0661873	5.7973412	1.5654253
**H**	−5.0065083	6.6786214	0.6293912
**H**	−6.5166754	6.0351078	−3.3188077
**H**	−8.5975796	5.1688134	−2.3802015
**H**	−8.936409	3.1009429	−1.3845596
**H**	−10.0197191	1.289523	−0.2023376
**H**	−10.4445488	3.7813629	3.2490037
**H**	−9.3554209	5.6091295	2.0560899
**H**	−9.3358919	1.8652156	4.0923739
**H**	−8.9298858	−0.3171567	5.0580341
**H**	−11.2460283	−2.132837	1.9532691
**H**	−11.6556471	0.0668709	0.9800931

Table S 16: Coordinates of the optimized geometry of **[9,9]** (PBEh-3c/def2-mSVP).

	x	y	z
**C**	8.0300295	−4.2774557	−0.9593465
**C**	4.9330187	6.2135223	−4.0262294
**C**	7.5095213	−3.3580506	−0.0494822
**C**	6.1466488	−3.2447692	0.1521273
**C**	5.2455583	−4.0516915	−0.5407852
**C**	5.7729263	−5.0380571	−1.3757587
**C**	7.1369427	−5.1462121	−1.5851799
**C**	3.8275895	5.9137223	−4.8221047
**C**	2.7078341	5.3015296	−4.2862996
**C**	2.6485805	4.9612379	−2.9343335
**C**	3.7155468	5.3462544	−2.1244048
**C**	4.8331436	5.9601322	−2.6586156
**C**	−3.2742579	1.0137827	−2.0317399
**C**	−4.0009423	0.2551284	−2.9517603
**C**	−3.4029471	−0.8944801	−3.4764483
**C**	−2.1665424	−1.3410754	−3.0363897
**C**	−1.5167782	−0.6465407	−2.0229431
**C**	−2.0507964	0.553276	−1.5814525
**C**	−0.2576854	−1.0022401	−1.2803447
**C**	−0.3177849	−2.4062855	−0.5709319
**C**	−1.5052985	−2.9024495	1.6665118
**C**	−1.949193	−2.417536	2.8979109
**C**	−1.6420374	−1.0947096	3.2322563
**C**	−1.0329395	−0.2362752	2.3286094
**C**	−0.7155961	−0.7008439	1.0566201
**C**	−0.8842232	−2.0467809	0.7766466
**C**	−0.1983048	0.0527298	−0.1403988
**C**	−1.134845	1.2377106	−0.5974332
**C**	−3.8764136	−2.6364676	4.4451414
**C**	−2.8298103	−3.2406554	3.7496379
**C**	−2.7616175	−4.6348337	3.7747222
**C**	−3.757398	−5.3904094	4.37047
**C**	−4.8581698	−4.7818796	4.973552
**C**	−4.8656792	−3.3897078	5.0474938
**C**	−7.6238324	1.0804035	−2.4416805
**C**	−8.2156604	0.599679	−3.6088777
**C**	−7.371788	0.2117552	−4.6491904
**C**	−5.9978335	0.1943195	−4.4823951
**C**	−5.4125982	0.5510034	−3.2658944
**C**	−6.2521935	1.0520394	−2.272182
**C**	−12.214857	−0.6442861	−2.8669565
**C**	−11.3418754	−1.4274122	−3.6223141
**C**	−10.092604	−0.9595277	−3.9855668
**C**	−9.6612321	0.307557	−3.5984691
**C**	−10.5945962	1.149932	−2.9932386

**C**	−11.8503316	0.685678	−2.6431891
**C**	−8.2100092	−5.5541233	6.4907008
**C**	−6.8925507	−5.1402127	6.4065014
**C**	−6.0903676	−5.5115726	5.3262198
**C**	−6.6077336	−6.4445928	4.4300238
**C**	−7.927328	−6.8509119	4.5094206
**C**	−8.779696	−6.3458232	5.4912193
**C**	−14.5010926	−3.3517106	−1.6292938
**C**	−13.8980324	−2.4931662	−2.5322126
**C**	−13.2899587	−1.3096836	−2.1052027
**C**	−13.5063798	−0.9256792	−0.7823301
**C**	−14.1135888	−1.7792793	0.1183237
**C**	−14.5251277	−3.0554819	−0.2648317
**C**	−12.7340962	−6.0546212	4.0023893
**C**	−12.2115536	−5.080216	4.8525817
**C**	−10.9891263	−5.2544033	5.4719144
**C**	−10.2367811	−6.4102172	5.2672218
**C**	−10.8558301	−7.4632321	4.5897783
**C**	−12.0817743	−7.2895974	3.9709906
**C**	−13.912515	−5.2811742	0.580396
**C**	−13.4600206	−6.0322388	1.6489333
**C**	−13.6887954	−5.6232239	2.9622892
**C**	−14.5859595	−4.5692249	3.1548058
**C**	−15.035231	−3.8143982	2.0857175
**C**	−14.6077057	−4.0899518	0.7847104
**O**	−1.8345719	1.862605	0.4399145
**C**	−0.2755855	2.3014238	−1.261628
**O**	−1.0751612	−3.3765949	−1.2389486
**C**	1.0960544	−2.9571535	−0.4775248
**C**	1.8643585	−2.8799248	0.6760425
**C**	3.1914439	−3.2861308	0.6768887
**C**	3.7987497	−3.7730496	−0.476625
**C**	3.0074537	−3.8897838	−1.6211933
**C**	1.6835424	−3.4958714	−1.6207512
**C**	0.1499166	2.1681731	−2.5785368
**C**	1.0593325	3.0565393	−3.1276606
**C**	1.5697793	4.1189732	−2.3858293
**C**	1.0960605	4.28148	−1.0847977
**C**	0.1939926	3.3895604	−0.5300801
**C**	9.7629304	−4.1227946	−2.7190761
**C**	10.9931955	−3.6598045	−3.1477611
**C**	11.9568803	−3.222776	−2.2389129
**C**	11.6941106	−3.4175021	−0.8812943
**C**	10.4589105	−3.8694447	−0.451791
**C**	9.4430068	−4.1588768	−1.3635321

**C**	13.0391854	−2.3412389	−2.7169577
**C**	6.7119164	5.9282374	−5.7248764
**C**	8.0459514	5.9895639	−6.082536
**C**	8.9629289	6.7190694	−5.3260306
**C**	8.459117	7.5080705	−4.2903497
**C**	7.1250346	7.4422194	−3.9288841
**C**	6.2407214	6.5908004	−4.5931402
**C**	10.3985764	6.4080767	−5.4614416
**C**	11.1491178	6.2124378	−4.3032045
**C**	12.3229076	5.4842989	−4.329696
**C**	12.7955327	4.9223288	−5.5154468
**C**	12.1554652	5.295545	−6.6994153
**C**	10.9785024	6.0232679	−6.6724977
**C**	13.3483851	−1.2013065	−1.9762743
**C**	13.9821563	−0.1216149	−2.5603457
**C**	14.3303699	−0.1352976	−3.9106874
**C**	14.2067955	−1.3485713	−4.5909588
**C**	13.5744784	−2.4311229	−4.0042198
**C**	14.9751981	2.3016043	−3.9722624
**C**	14.5908921	3.5681955	−4.3757124
**C**	13.6765842	3.7423279	−5.4177688
**C**	13.3563854	2.6188191	−6.1790296
**C**	13.7419461	1.3538022	−5.7776156
**C**	14.4617929	1.163522	−4.5987927
**H**	8.173591	−2.6561234	0.4387351
**H**	5.7773492	−2.4572034	0.7963562
**H**	5.1063143	−5.7236285	−1.8839291
**H**	7.5118559	−5.9055028	−2.2605132
**H**	3.849755	6.1446583	−5.8800685
**H**	1.8709254	5.0711202	−4.9337494
**H**	3.7119776	5.0868457	−1.0733796
**H**	5.6796129	6.1615081	−2.0145001
**H**	−3.6663076	1.9536626	−1.66163
**H**	−3.9424952	−1.491719	−4.2000751
**H**	−1.7418094	−2.2403595	−3.4625159
**H**	0.6128442	−0.9478219	−1.9380019
**H**	−1.6909453	−3.9302447	1.3792353
**H**	−1.901177	−0.7204344	4.2146694
**H**	−0.8232417	0.7846825	2.6196101
**H**	0.8153357	0.4203801	0.0336075
**H**	−3.9768147	−1.5588937	4.4392355
**H**	−1.931151	−5.1423145	3.2999061
**H**	−3.6908504	−6.4710514	4.3413148
**H**	−5.7118294	−2.8830768	5.493652
**H**	−8.2493931	1.3809549	−1.6108783

H	−7.7962801	−0.1117456	−5.5916698
H	−5.3738886	−0.131682	−5.3051913
H	−5.8410319	1.3347154	−1.3117191
H	−11.5827491	−2.4636166	−3.8205278
H	−9.3977225	−1.6386134	−4.4632135
H	−10.3170879	2.1672709	−2.7457802
H	−12.5327159	1.3530844	−2.1315682
H	−8.8203817	−5.2058082	7.3148189
H	−6.4962789	−4.4759452	7.1647459
H	−6.0127039	−6.7609373	3.5826341
H	−8.3285838	−7.4684245	3.7162702
H	−14.8899929	−4.2994463	−1.9813564
H	−13.8361171	−2.7859853	−3.5731125
H	−13.0662023	−0.011374	−0.4067463
H	−14.124337	−1.5040645	1.1649276
H	−12.6871931	−4.1103394	4.9155788
H	−10.554573	−4.4159394	6.0003638
H	−10.3423312	−8.4110172	4.4838098
H	−12.4986523	−8.1047628	3.3922363
H	−13.5764658	−5.5427961	−0.414628
H	−12.7852422	−6.8548294	1.451017
H	−14.8689426	−4.2791	4.159307
H	−15.6570352	−2.9479744	2.2756897
H	−2.3973066	1.2057418	0.8629762
H	−1.9862814	−3.067598	−1.290205
H	1.4368532	−2.493418	1.5918257
H	3.7583846	−3.2186875	1.5969543
H	3.4473533	−4.2475361	−2.5434386
H	1.1100843	−3.580884	−2.5345938
H	−0.213253	1.3528721	−3.1907179
H	1.4102027	2.8889783	−4.1380677
H	1.4368628	5.1208677	−0.4912459
H	−0.1372105	3.5431641	0.4872435
H	9.0001712	−4.3437207	−3.4550064
H	11.1543917	−3.5228969	−4.2091405
H	12.4456886	−3.1608682	−0.1449454
H	10.2657843	−3.9568765	0.6103742
H	6.0554441	5.2590405	−6.2663691
H	8.3971942	5.3600404	−6.8898482
H	9.1284533	8.1440558	−3.7241158
H	6.7743692	8.0293619	−3.0890284
H	10.7428736	6.5042202	−3.3434279
H	12.7922752	5.2276313	−3.389033
H	12.5398492	4.9506306	−7.6515531
H	10.4684655	6.2352212	−7.60424
H	12.9634068	−1.0910343	−0.9709805
H	14.0679463	0.796254	−1.99346
H	14.5422539	−1.4265904	−5.6179564
H	13.4348055	−3.3356155	−4.5834449
H	15.6165208	2.1958547	−3.1056949
H	14.9410603	4.4276653	−3.8171693
H	12.6638298	2.7086845	−7.0057311
H	13.3378647	0.4989279	−6.3042052

Table S 17: Coordinates of the optimized geometry of **[11,9]** (PBEh-3c/def2-mSVP).

	x	y	z
C	9.1421108	−5.979708	0.1772713
C	9.8884466	5.3383364	−0.0495981
C	8.8419164	−5.1874286	1.2846138
C	7.5714903	−4.6773949	1.4754633
C	6.5434149	−4.9450733	0.5727708
C	6.8210832	−5.8176716	−0.4806457
C	8.0954951	−6.3208364	−0.6782914
C	8.8468334	5.6274913	−0.9309705
C	7.5362625	5.3153894	−0.6134609
C	7.2138127	4.6992952	0.5963314
C	8.2444838	4.4962866	1.5123409
C	9.5533475	4.8099404	1.1965971
C	0.3087553	2.8879298	0.2131679
C	−0.494353	2.6675435	−0.9068401
C	−0.2583918	1.5239476	−1.6754827
C	0.6876964	0.579777	−1.3080346
C	1.4012329	0.7581948	−0.128412
C	1.2397922	1.9353091	0.5840492
C	2.3899823	−0.1614132	0.5351361
C	1.8177254	−1.5908121	0.8591989
C	0.3399508	−2.2036158	2.8837823
C	−0.0384584	−1.9163745	4.1958059
C	0.6067332	−0.8636616	4.8524401
C	1.5191147	−0.0472127	4.2008366
C	1.8025118	−0.2790973	2.8591157
C	1.2613156	−1.3959246	2.2443278
C	2.6541402	0.5182495	1.9070897

C	2.2001371	2.0197418	1.743993
C	−2.0411755	−1.9655694	5.6653347
C	−1.1540279	−2.6439637	4.8305455
C	−1.4344552	−3.9797162	4.5396039
C	−2.5932487	−4.5840942	4.9963674
C	−3.5140732	−3.879549	5.7708847
C	−3.193326	−2.5712379	6.1292762
C	−3.2594788	4.3988736	−2.8084927
C	−4.0689241	4.87021	−1.7755916
C	−3.5927702	4.7479672	−0.4712347
C	−2.4031633	4.0962413	−0.2032764
C	−1.6356548	3.5456708	−1.2285121
C	−2.064776	3.754221	−2.5400126
C	−8.2404699	5.7722573	−2.0310794
C	−7.6564789	4.8015763	−2.8445664
C	−6.290859	4.5850347	−2.8333155
C	−5.4532458	5.3244743	−2.00071
C	−6.020367	6.3732107	−1.2767878
C	−7.3857522	6.596671	−1.2971533
C	−6.965892	−5.2415692	5.2535265
C	−5.6124241	−5.0118533	5.0835785
C	−4.8582269	−4.3988313	6.0819115
C	−5.4827362	−4.1441674	7.3030602
C	−6.8343297	−4.3825448	7.4756818
C	−7.6176615	−4.8748414	6.430568
C	−14.5444003	2.1088209	−0.4468462
C	−13.7460725	2.998348	−1.1413164
C	−13.5305751	4.2909644	−0.6666577
C	−14.2929451	4.711855	0.4242833
C	−15.0973817	3.8229984	1.1152048
C	−15.1634335	2.4773708	0.7471133
C	−14.9066369	−1.987095	4.1221448
C	−14.8050697	−2.0096741	5.5145228
C	−13.9009992	−2.8434895	6.1483345
C	−13.0656027	−3.688107	5.4146682
C	−13.3020238	−3.7939639	4.0451424
C	−14.2045721	−2.9600242	3.4115804
C	−16.0423335	0.1702462	1.2628439
C	−15.9781474	−0.9337712	2.0952593
C	−15.4516663	−0.8316718	3.3843924
C	−15.1723001	0.4478778	3.8625053
C	−15.2336739	1.5491741	3.0306169
C	−15.5790915	1.4179906	1.6860947
O	1.6131549	2.5799026	2.8836887
C	3.4231507	2.8592794	1.412397

O	0.8635927	−2.0669	−0.0491256
C	2.9674524	−2.5852132	0.8332447
C	3.4690841	−3.0018802	−0.3984945
C	4.5939622	−3.8000145	−0.4747072
C	5.2597818	−4.2262101	0.676384
C	4.7215904	−3.8531067	1.9042493
C	3.5984408	−3.0418152	1.9823357
C	4.1225555	3.5192434	2.419788
C	5.3192222	4.160886	2.1492887
C	5.867302	4.1631183	0.8674622
C	5.1387034	3.5427448	−0.1444762
C	3.9370532	2.9077504	0.1214193
C	11.4992074	−6.5523285	0.8385566
C	12.854769	−6.4513394	0.5802742
C	13.320037	−6.0268084	−0.6660521
C	12.3786911	−5.8747913	−1.684186
C	11.0242811	−5.9878219	−1.4296793
C	10.5559689	−6.2480634	−0.1436521
C	14.6736475	−5.47193	−0.8580465
C	11.6757336	4.8990406	−1.7065427
C	12.9941634	4.5972262	−1.9929403
C	13.9975267	4.7707454	−1.0398096
C	13.6478248	5.403854	0.1542625
C	12.3275205	5.7008172	0.443008
C	11.3057828	5.3830729	−0.4532845
C	15.2685924	4.0416828	−1.2100357
C	15.7768567	3.3280152	−0.125863
C	16.6571749	2.2796432	−0.3129007
C	17.0659146	1.9017048	−1.5916914
C	16.7204097	2.746407	−2.6490543
C	15.8371359	3.795347	−2.4617338
C	15.242077	−4.7245864	0.1725334
C	16.2485939	−3.8123232	−0.0792149
C	16.7269142	−3.607063	−1.3729992
C	16.3034352	−4.4991479	−2.3604399
C	15.2969749	−5.4148099	−2.1069878
C	18.1104134	−1.592107	−0.752915
C	18.1953024	−0.2121377	−0.8087448
C	17.512373	0.5092194	−1.7912548
C	16.946233	−0.2179669	−2.8376952
C	16.863073	−1.5963614	−2.7831642
C	17.341437	−2.300809	−1.6791454
C	−12.3446953	5.0291011	−1.1397384
C	−11.4893524	5.5883425	−0.1912797
C	−10.1931141	5.9388794	−0.5176904

C	−9.6982387	5.7505093	−1.8073521
C	−10.6018986	5.3372212	−2.7883267
C	−11.8983924	4.9801018	−2.4609066
C	−11.040715	−3.4215648	6.7972969
C	−9.7093915	−3.7028027	7.0370118
C	−9.0906493	−4.8095193	6.4574788
C	−9.8983628	−5.6994784	5.7473979
C	−11.2319276	−5.4171593	5.5057514
C	−11.8110859	−4.2335951	5.96579
H	9.6356334	−4.8880482	1.9572558
H	7.4017384	−3.9918361	2.295701
H	6.0324515	−6.0973979	−1.1679504
H	8.2836003	−6.9743632	−1.5213256
H	9.0650852	6.0862511	−1.8875269
H	6.7517972	5.5422167	−1.3247336
H	8.035315	4.0072173	2.455218
H	10.3379508	4.5538818	1.8971642
H	0.208916	3.799512	0.7903565
H	−0.8651069	1.3391688	−2.5527153
H	0.8395692	−0.2930833	−1.9291163
H	3.3001621	−0.2463528	−0.0629591
H	−0.1130121	−3.0286766	2.3474617
H	0.385621	−0.6727724	5.8950534
H	1.9953528	0.7601135	4.7414912
H	3.7065874	0.4736926	2.1958469
H	−1.872104	−0.9217161	5.8963021
H	−0.7426526	−4.5565536	3.938531
H	−2.7897976	−5.6172878	4.737889
H	−3.8967742	−1.9893357	6.7111252
H	−3.5753453	4.5253195	−3.836697
H	−4.2038201	5.0899021	0.3544683
H	−2.1105158	3.9449019	0.82786
H	−1.4633872	3.3958821	−3.3661606
H	−8.2847627	4.1337028	−3.4195634
H	−5.8845513	3.7594088	−3.403895
H	−5.3876512	7.0065665	−0.6671787
H	−7.7957996	7.4048092	−0.7042783
H	−7.5407289	−5.6172442	4.4167818
H	−5.1599764	−5.218855	4.1219101
H	−4.9084685	−3.7266999	8.1209815
H	−7.2941307	−4.1490845	8.4279869
H	−14.5729073	1.0765405	−0.7707331
H	−13.177343	2.636781	−1.9884657
H	−14.2110166	5.7337219	0.7740242
H	−15.6316732	4.1680175	1.9918882

H	−15.3950233	−1.3218779	6.1078806
H	−13.8021816	−2.7941585	7.2258287
H	−12.6790971	−4.4414346	3.4414103
H	−14.2583522	−2.9780873	2.3306831
H	−16.3983845	0.0443271	0.2476932
H	−16.2831928	−1.9006357	1.7139798
H	−14.7659329	0.5720286	4.8578927
H	−14.8726067	2.4990135	3.4028593
H	0.8255054	2.0698299	3.0993065
H	0.1073687	−1.4701838	−0.0348416
H	2.9950759	−2.673514	−1.3143212
H	4.9906672	−4.0542913	−1.4494063
H	5.1826321	−4.1969817	2.8216469
H	3.2239039	−2.7630306	2.9584489
H	3.738864	3.5286687	3.4302037
H	5.8354741	4.6702496	2.9537264
H	5.5356293	3.5126786	−1.1513051
H	3.4095783	2.4315833	−0.6950696
H	11.1672979	−6.8291703	1.8317157
H	13.5589114	−6.6534963	1.3779276
H	12.696001	−5.5419291	−2.6638828
H	10.3206546	−5.7501389	−2.2174673
H	10.9114237	4.6411194	−2.4285841
H	13.2230865	4.1042528	−2.9288984
H	14.4109364	5.6211298	0.8915613
H	12.0821909	6.1472483	1.3988252
H	15.3719854	3.493486	0.864118
H	16.9073187	1.6579157	0.5367647
H	17.0867053	2.5380204	−3.6468848
H	15.5334116	4.3877317	−3.3161767
H	14.7994279	−4.7442702	1.1598169
H	16.5555303	−3.1480415	0.7180844
H	16.7127965	−4.428805	−3.3608649
H	14.9448937	−6.045801	−2.9138301
H	18.5790807	−2.1164929	0.0709438
H	18.7302536	0.3145919	−0.0279162
H	16.4200384	0.299441	−3.6292655
H	16.2752645	−2.1086443	−3.5337728
H	−11.7982787	5.6372971	0.8449724
H	−9.5257927	6.2537231	0.2740673
H	−10.2758845	5.2499641	−3.8173953
H	−12.5587552	4.6138764	−3.2374562
H	−11.4512641	−2.4908994	7.1671347
H	−9.1177837	−2.9848836	7.590215
H	−9.4696093	−6.6073696	5.3413069

**H**	−11.8187908	−6.1071986	4.9119495

Table S 18: Coordinates of the optimized geometry of **[11,11]** (PBEh-3c/def2-mSVP).

	x	y	z
**C**	6.7805812	−4.9407262	−3.5761681
**C**	6.6175829	4.8734207	3.8497332
**C**	6.3746608	−3.7163048	−4.1052458
**C**	5.1064937	−3.2194447	−3.8668632
**C**	4.1894687	−3.9268686	−3.0912781
**C**	4.5737823	−5.1808329	−2.6160323
**C**	5.8438679	−5.677137	−2.8519065
**C**	5.6923234	4.5107086	4.8271469
**C**	4.4362392	4.0438431	4.4811421
**C**	4.0575896	3.9108751	3.1447909
**C**	4.9642268	4.32829	2.1714859
**C**	6.2158079	4.8036473	2.5166573
**C**	−2.45357	2.7360549	0.487459
**C**	−3.0354558	3.2976448	−0.6495414
**C**	−2.5515167	2.9033629	−1.9010058
**C**	−1.5979068	1.9049758	−2.0328455
**C**	−1.1077366	1.2773739	−0.892387
**C**	−1.4927055	1.7521848	0.3500747
**C**	−0.1699434	0.1056011	−0.7706837
**C**	−0.6819471	−1.1988334	−1.4936114
**C**	−2.3646018	−2.9147571	−0.5458925
**C**	−2.9707988	−3.4700093	0.5819291
**C**	−2.5520031	−3.0313443	1.8415555
**C**	−1.6206468	−2.0152806	1.9883438
**C**	−1.109731	−1.3946877	0.8541338
**C**	−1.4478145	−1.8908766	−0.3940865
**C**	−0.186279	−0.2115572	0.7505055
**C**	−0.728803	1.0831552	1.461187
**C**	−4.3335097	−5.405008	1.426323
**C**	−4.0983655	−4.4151809	0.471018
**C**	−5.0439863	−4.2578752	−0.5410794
**C**	−6.2143433	−4.9940033	−0.5473053
**C**	−6.4921302	−5.9133246	0.462944
**C**	−5.5085695	−6.1358637	1.4258774
**C**	−5.5274995	5.7703164	0.7495594
**C**	−6.5920056	5.644447	−0.1422278
**C**	−6.4023367	4.8472812	−1.2698693
**C**	−5.2293461	4.1406867	−1.4560541
**C**	−4.1909059	4.2070133	−0.5279764
**C**	−4.349247	5.0692197	0.5578715
**C**	−10.6838753	6.641949	0.5865355
**C**	−10.0969997	6.9279619	−0.6471223
**C**	−8.751083	6.6967572	−0.8675961
**C**	−7.9359043	6.1806051	0.1399677
**C**	−8.4942575	6.0190535	1.4062247
**C**	−9.8419943	6.2417106	1.6237775
**C**	−9.8654135	−6.6832884	1.8926609
**C**	−8.5069965	−6.4410517	1.8037546
**C**	−7.850143	−6.4759533	0.5753746
**C**	−8.5808059	−6.8880249	−0.5389941
**C**	−9.9380166	−7.1414741	−0.4472391
**C**	−10.6217911	−6.9796591	0.7590601
**C**	−17.7990875	3.4724718	0.6717807
**C**	−16.9584051	4.564421	0.5476978
**C**	−15.9714691	4.8234041	1.5007567
**C**	−15.9846472	4.0556294	2.6640367
**C**	−16.8235667	2.9637913	2.7873815
**C**	−17.6832995	2.5965578	1.7528837
**C**	−17.6822078	−3.0057911	1.6907462
**C**	−16.8665542	−3.4133807	2.7458253
**C**	−16.0250736	−4.5026378	2.6181736
**C**	−15.9617291	−5.2250023	1.4279184
**C**	−16.9057763	−4.9283019	0.4434335
**C**	−17.7529034	−3.8420687	0.5744501
**C**	−18.4907828	0.4744533	2.8520274
**C**	−18.491833	−0.9095412	2.8350058
**C**	−18.1736489	−1.6147394	1.6722458
**C**	−18.0471103	−0.8804449	0.4936932
**C**	−18.0489908	0.5011345	0.5098373
**C**	−18.1751704	1.2065673	1.7057604
**O**	−1.5149534	0.8497293	2.5968005
**C**	0.4557394	1.9146975	1.9261379
**O**	−1.4678343	−0.9676563	−2.6278626
**C**	0.5209299	−2.0115949	−1.9440372
**C**	1.015105	−1.8839678	−3.2396943
**C**	2.1785056	−2.5298103	−3.6218347
**C**	2.895611	−3.3218197	−2.7265637
**C**	2.3795083	−3.4703691	−1.4417743
**C**	1.2119576	−2.8315349	−1.058982
**C**	0.9409922	3.0012659	1.2112251
**C**	2.0829156	3.6729615	1.6234732

C	2.783754	3.2769418	2.7583114
C	2.2749584	2.2011114	3.4881579
C	1.1330187	1.5368773	3.0840759
C	8.9469301	−5.1236391	−4.8297901
C	10.3196148	−5.2970547	−4.8298707
C	10.9941656	−5.7002677	−3.6769673
C	10.2260915	−6.025658	−2.5595103
C	8.8537056	−5.8564506	−2.5606827
C	8.1925208	−5.3510335	−3.6784864
C	12.4592986	−5.6001968	−3.5466519
C	8.7247283	6.1897596	3.5005919
C	10.095067	6.3155067	3.6435644
C	10.8182526	5.4394182	4.4542873
C	10.0974163	4.5089373	5.2023374
C	8.7262118	4.3896094	5.0675147
C	8.0205246	5.1903646	4.1719202
C	12.2879321	5.3402278	4.3868283
C	13.0609093	4.9084068	5.4662106
C	14.3740429	4.5076581	5.2902228
C	14.9677628	4.5274418	4.0277079
C	14.2400208	5.0939899	2.9820242
C	12.927747	5.4898515	3.1573472
C	13.1986278	−6.4533101	−2.725736
C	14.4974974	−6.144851	−2.3607006
C	15.1084178	−4.9704087	−2.802316
C	14.4178402	−4.1961572	−3.7340289
C	13.1208227	−4.5044139	−4.0983832
C	18.4826113	−1.4781179	0.020185
C	18.8753227	−1.5464874	1.3584009
C	18.8609325	−0.4204194	2.1629171
C	18.4518374	0.8165967	1.6603435
C	18.2477352	0.9125762	0.2844168
C	18.2634021	−0.2113215	−0.5191352
C	−14.7877525	5.6536387	1.2094946
C	−14.2028311	5.5594037	−0.052714
C	−12.9094785	5.9917877	−0.2749592
C	−12.1453726	6.5389559	0.7546868
C	−12.7817533	6.7686634	1.975753
C	−14.0768939	6.3340714	2.199233
C	−14.1194527	−6.7900372	2.146037
C	−12.8113754	−7.2110286	1.9801345
C	−12.0927971	−6.8945157	0.8255006
C	−12.7878536	−6.2775552	−0.21368
C	−14.0959352	−5.8625454	−0.0511078
C	−14.7632149	−6.041636	1.1599034

C	16.1849869	3.7608626	3.7032987
C	16.3274863	2.4762058	4.2245742
C	17.1892151	1.5642625	3.6444177
C	17.9456872	1.9013573	2.5225065
C	17.9385098	3.238867	2.1212598
C	17.0721869	4.1504367	2.6984152
C	18.0058935	−2.6526309	−0.7341875
C	17.2354761	−3.6039504	−0.0665409
C	16.3993695	−4.4589903	−0.7596623
C	16.2991022	−4.3963591	−2.1484949
C	17.2015054	−3.5712101	−2.8223354
C	18.0399749	−2.716584	−2.1285406
H	7.084908	−3.1079412	−4.6508307
H	4.8500467	−2.2337842	−4.2341096
H	3.8680862	−5.7795599	−2.053775
H	6.1118294	−6.6528305	−2.4656304
H	5.9585348	4.594062	5.8736777
H	3.7382279	3.7786721	5.2653461
H	4.7156858	4.2179691	1.123666
H	6.9198051	5.0548318	1.7333319
H	−2.7779038	3.0341317	1.4771639
H	−2.9340839	3.3825019	−2.793459
H	−1.2506504	1.6242743	−3.0185023
H	0.8282703	0.3662545	−1.1292214
H	−2.605331	−3.2819481	−1.5365021
H	−3.0037283	−3.4556947	2.7290482
H	−1.3244406	−1.7022117	2.9807747
H	0.8074742	−0.461261	1.1292817
H	−3.5933464	−5.6001892	2.1922524
H	−4.9041745	−3.491852	−1.2930413
H	−6.9629666	−4.7870122	−1.301518
H	−5.6706874	−6.8812694	2.1947117
H	−5.6247783	6.4180017	1.6120969
H	−7.2164021	4.7107947	−1.9703511
H	−5.1551467	3.4669219	−2.2999487
H	−3.5440195	5.1891808	1.2718989
H	−10.7076542	7.3043637	−1.4584413
H	−8.3318949	6.893955	−1.8466135
H	−7.890968	5.6155455	2.2097147
H	−10.2612614	6.0000958	2.5920689
H	−10.3590549	−6.537293	2.8447415
H	−7.9711594	−6.1190616	2.6877708
H	−8.0859806	−6.9852968	−1.4975118
H	−10.4798413	−7.4363675	−1.3373099
H	−18.5134831	3.26372	−0.1151844

H	−17.0316926	5.1903853	−0.3332015
H	−15.2385894	4.2277618	3.4292178
H	−16.7067982	2.3130206	3.644365
H	−16.7839029	−2.7959153	3.6308278
H	−15.3128324	−4.7067183	3.4074324
H	−16.9388496	−5.5191298	−0.4638158
H	−18.4348031	−3.6053807	−0.2330767
H	−18.6760425	0.9909827	3.7859099
H	−18.6798297	−1.4472679	3.756229
H	−17.8043785	−1.3863256	−0.4316789
H	−17.8085887	1.0307187	−0.4029205
H	−2.2919857	0.3467831	2.3296307
H	−2.2462983	−0.468667	−2.3593785
H	0.4916987	−1.2745039	−3.9627926
H	2.5284989	−2.4225551	−4.6411488
H	2.9151834	−4.0654882	−0.7129777
H	0.8487308	−2.9749791	−0.0495149
H	0.43076	3.3402651	0.319291
H	2.4232126	4.526744	1.0510171
H	2.8037278	1.8499757	4.3653152
H	0.7790878	0.6975821	3.6683452
H	8.4549707	−4.7877796	−5.7342586
H	10.8781593	−5.091904	−5.7348077
H	10.7156603	−6.3256892	−1.6418296
H	8.3012556	−6.0335704	−1.6465532
H	8.1948484	6.8666689	2.8418069
H	10.6135045	7.0915909	3.0943242
H	10.6239361	3.801391	5.8299101
H	8.2111038	3.5983987	5.5976221
H	12.6187468	4.8466866	6.4529233
H	14.9318507	4.1339097	6.1401629
H	14.6641888	5.1182161	1.9864546
H	12.3628772	5.8152513	2.2933296
H	12.7393379	−7.3527365	−2.3345079
H	15.0286789	−6.806388	−1.6873624
H	14.8548379	−3.2731151	−4.0926378
H	12.5802648	−3.8160234	−4.7352599
H	19.1336164	−2.5036058	1.7948049
H	19.1091598	−0.5193006	3.2125776
H	17.9332904	1.8535516	−0.1479374
H	17.9612314	−0.1131388	−1.5537565
H	−14.718962	5.0318211	−0.8444007
H	−12.4525712	5.7923095	−1.2356396
H	−12.2435417	7.257262	2.7786565
H	−14.5247153	6.4867026	3.1735727
H	−14.6316086	−7.0068384	3.0755308
H	−12.3283344	−7.7562941	2.7815382
H	−12.2681349	−6.0102519	−1.1246186
H	−14.5609948	−5.2818903	−0.8373936
H	15.6610711	2.1384979	5.0079428
H	17.1679826	0.5386518	3.989454
H	18.5731876	3.5588974	1.3040073
H	17.0445151	5.1657719	2.3221369
H	17.1826275	−3.5889551	1.0144149
H	15.7200327	−5.0893703	−0.2003774
H	17.2082551	−3.5544625	−3.9053233
H	18.6858181	−2.0459609	−2.6820288

Table S 19: Coordinates of **[9,0]-THII-2** (PBEh-3c/def2-mSVP).

	x	y	z
C	0.8466065	3.7167818	2.1496038
C	1.9239802	2.8854133	2.4127907
C	1.7981254	1.845371	3.3283107
C	0.5907481	1.6057464	3.9689728
C	−0.5031165	2.4151067	3.6743012
C	−0.3557438	3.4737072	2.7943741
C	−1.8945332	2.3607689	4.2445736
C	−2.6424524	0.9915473	4.0362736
C	−4.2011901	0.3199996	2.092871
C	−5.000879	0.7518399	1.0463505
C	−5.0949293	2.1081645	0.7509934
C	−4.3717621	3.0444548	1.4762724
C	−3.5366385	2.6074808	2.5009815
C	−3.4833365	1.2601715	2.8148147
C	−2.6750697	3.4243137	3.4253618
C	−1.5989794	4.3210666	2.7075008
O	−1.913054	4.662528	1.3857236
C	−1.4397371	5.6233162	3.4739615
O	−1.7984127	−0.1130194	3.8630373
C	−3.5048561	0.7013112	5.2531852
C	−3.0324991	−0.1117932	6.2794483
C	−3.7883923	−0.3199264	7.4214506
C	−5.0367676	0.2724982	7.5836332
C	−5.5014243	1.0856195	6.5574653
C	−4.7518226	1.294864	5.4098069
C	−0.6559081	5.6926809	4.619567
C	−0.5746569	6.8638898	5.3574687

C	−1.265756	8.0065183	4.9744522
C	−2.0481488	7.9320254	3.8263853
C	−2.1371628	6.7641005	3.0869859
C	−1.1507041	9.2854458	5.7509033
C	−5.862338	0.0152949	8.8100466
H	0.9432083	4.552843	1.468491
H	2.6523673	1.2176837	3.5465762
H	0.5119138	0.8057699	4.6929278
H	−1.8695331	2.6068505	5.3084489
H	−4.1499962	−0.7287382	2.357025
H	−5.7422112	2.4379494	−0.0512635
H	−4.4688963	4.0975821	1.2486189
H	−3.3038717	4.0473061	4.0652023
H	−2.0182029	3.8507476	0.8791186
H	−1.2409464	0.0483234	3.0949451
H	−2.0672224	−0.5905841	6.1926062
H	−3.3961203	−0.9597141	8.2036446
H	−6.4700962	1.5626443	6.6490808
H	−5.1583999	1.924262	4.6281592
H	−0.0889588	4.8297601	4.9457846
H	0.0454811	6.8857261	6.2458588
H	−2.6008158	8.8056086	3.5001477
H	−2.7564245	6.7478044	2.2010971
H	−0.4118629	9.9530175	5.3029019
H	−2.0974206	9.8250548	5.7788792
H	−0.8420716	9.1045561	6.7800446
H	−6.422148	−0.9177785	8.7192188
H	−5.2416158	−0.0668424	9.7022902
H	−6.5858176	0.8116509	8.9820005
H	2.8724159	3.0560827	1.9205212
H	−5.5699735	0.0353866	0.4685772

Table S 20: Coordinates of **[11,0]-THII-2** (PBEh-3c/def2-mSVP).

	x	y	z
C	5.1669548	−3.3744162	−0.0892598
C	4.7047495	−3.9143217	1.1006558
C	5.0792994	−3.3515875	2.3167497
C	5.8944838	−2.2294343	2.3585741
C	6.3259294	−1.6624324	1.1623795
C	5.9826008	−2.2550411	−0.0407151
C	7.2170345	−0.4679055	0.9554004
C	6.6864665	0.8715099	1.5899288
C	5.2408302	2.6908405	0.4685456
C	4.7869842	3.2417194	−0.7195539
C	5.1429789	2.6701296	−1.9370909
C	5.9307876	1.5287162	−1.9820834
C	6.3533325	0.9515281	−0.7875794
C	6.0291332	1.552181	0.4168327
C	7.2163969	−0.2639979	−0.5840638
C	6.6515589	−1.5903628	−1.2163791
O	5.7669341	−1.3954265	−2.2848751
C	7.8054259	−2.4256477	−1.7450422
O	5.8014136	0.6975974	2.6616846
C	7.8618362	1.6790701	2.1142868
C	8.649005	2.440435	1.2581969
C	9.7650745	3.1156639	1.7280218
C	10.1297158	3.0608464	3.067613
C	9.3379723	2.2983842	3.9202762
C	8.223948	1.6176827	3.4567045
C	8.1653195	−2.3707934	−3.0884376
C	9.2610348	−3.0776401	−3.5562005
C	10.0363201	−3.8606706	−2.7069156
C	9.6740978	−3.9088952	−1.3665113
C	8.5760578	−3.2073934	−0.8924358
C	11.2021282	−4.647973	−3.2292157
C	11.3156229	3.8209035	3.5850023
H	4.9137824	−3.8263662	−1.0399806
H	4.7341274	−3.7957863	3.2413534
H	6.1935978	−1.8131027	3.3112068
H	8.2185378	−0.6817234	1.3352091
H	5.0023606	3.1487087	1.4202365
H	4.8048872	3.1225169	−2.8603381
H	6.2159832	1.1053283	−2.9358726
H	8.2212233	−0.0741617	−0.9678581
H	5.0250522	−0.8673628	−1.9726951
H	5.0459427	0.187505	2.3522326
H	8.3921243	2.5226198	0.2094693
H	10.3580801	3.7009324	1.0351311
H	9.5950761	2.2340093	4.9713524
H	7.6353254	1.0360978	4.1522093
H	7.5891593	−1.773787	−3.7813212
H	9.5166907	−3.0178608	−4.6079097
H	10.2545051	−4.5096769	−0.6762463
H	8.3201556	−3.285074	0.1568671
H	10.8748716	−5.5945591	−3.6641332
H	11.9144941	−4.883614	−2.4391863
H	11.7379666	−4.1040975	−4.0071936
H	11.014346	4.7834467	4.003374
H	12.0391429	4.0231147	2.7958021
H	11.8305483	3.2733579	4.3743798
H	4.0663586	−4.7879653	1.0868307
H	4.169873	4.1304817	−0.7032431

Table S 21: Coordinates of **[0,9]-THII-2** (PBEh-3c/def2-mSVP).

	x	y	z
C	1.8454523	−1.3508248	7.9442847
C	1.1286552	−0.6331371	8.8885853
C	−0.1893078	−0.2651336	8.6361356
C	−0.8144042	−0.6318501	7.453033
C	−0.1071573	−1.3864421	6.5208769
C	1.2163289	−1.7116763	6.7631733
C	−0.5619297	−1.8836677	5.1759619
C	−1.8305682	−2.8162843	5.2113438
C	−1.9033867	−5.3932101	5.3153864
C	−1.21312	−6.5807399	5.1312884
C	0.1267208	−6.5610306	4.756251
C	0.7989732	−5.3590897	4.5877707
C	0.1164695	−4.1661821	4.8107524
C	−1.2270271	−4.1957988	5.1428753
C	0.6228178	−2.755836	4.6782661
C	1.8583367	−2.4095922	5.5915201
O	2.6155232	−3.5177539	5.9905463
O	−2.641471	−2.6529081	6.3411012
C	−3.1061948	−2.7126085	1.6229741
C	−4.2135938	−1.8866021	1.7342209
C	−4.5627782	−1.3841291	2.9780026
C	−3.8120844	−1.6997539	4.1009094
C	−2.6984488	−2.5281813	3.996961
C	−2.3576239	−3.0325436	2.7453706
C	3.9277155	−1.9796219	4.1990055
C	2.795299	−1.480818	4.8362353
C	2.5025749	−0.1259298	4.7109951
C	3.3166416	0.710131	3.9621832
C	4.4423696	0.2060608	3.3299827
C	4.7437951	−1.1412034	3.453912
H	−0.7484086	−1.0374817	4.5110528
H	0.8689372	−2.5463034	3.6349536
H	2.8827847	−1.6105626	8.1137283
H	1.5995307	−0.3405073	9.8178865
H	−0.7325705	0.3169057	9.3692027
H	−1.8336933	−0.3244047	7.2613038
H	−2.9555425	−5.3959758	5.5706531
H	−1.7209297	−7.5275207	5.260455
H	0.6501915	−7.4936718	4.590536
H	1.8357099	−5.3571476	4.2789379
H	2.0406849	−4.1227669	6.4702129
H	−2.1109113	−2.8281205	7.1249247
H	−2.8249939	−3.1186046	0.660057
H	−5.4284353	−0.7423094	3.0790876
H	−4.1024571	−1.2988477	5.0615532
H	−1.5071747	−3.6941892	2.6384623
H	4.1815755	−3.0265849	4.2843345
H	1.6383012	0.2925819	5.2113112
H	3.0719229	1.7611362	3.8808533
H	5.6229473	−1.5461305	2.9695079
H	−4.8016655	−1.6400013	0.8600498
H	5.0812792	0.858224	2.749211

Table S 22: Coordinates of **[0,11]-THII-2** (PBEh-3c/def2-mSVP).

	x	y	z
C	9.4089881	4.0913529	−1.4918015
C	10.1164694	4.3267764	−0.3237163
C	10.0633795	3.4122444	0.7238209
C	9.3274476	2.2419868	0.6074226
C	8.6477188	1.9867442	−0.5806737
C	8.672963	2.9218828	−1.6008719
C	7.7812944	0.808105	−0.930619
C	8.5068239	−0.5883593	−0.8572755
C	9.6501155	−1.8870249	−2.7711918
C	9.7956458	−2.0445851	−4.1402581
C	9.128375	−1.195499	−5.0179411
C	8.3314649	−0.1652049	−4.5403377
C	8.2155309	0.0172347	−3.1648309
C	8.850364	−0.8575395	−2.3002173
C	7.4082512	1.0445182	−2.4196734
C	7.7760376	2.5402768	−2.750899
O	8.4033036	2.7245477	−3.9889809
O	9.6396514	−0.6125174	−0.0348646
C	5.6147303	−3.0549997	−0.6172931
C	5.6747693	−3.4431204	0.7120984
C	6.6693129	−2.9239806	1.5258642
C	7.5951433	−2.0233505	1.0194803
C	7.5410714	−1.6289608	−0.3139623
C	6.5416568	−2.1582189	−1.1255703
C	5.8962192	3.7225755	−3.967945
C	6.5077465	3.3780518	−2.7663687
C	5.8980853	3.7599497	−1.5746078
C	4.7025357	4.4615758	−1.5816021
C	4.0982287	4.7996511	−2.7824995
C	4.7024497	4.4294855	−3.9735734
H	6.8968849	0.7940985	−0.2898089
H	6.3433861	0.8821035	−2.5999857
H	9.4131755	4.8129536	−2.2989317
H	10.6983949	5.2327919	−0.2167446
H	10.5995643	3.6172212	1.6413061
H	9.2799119	1.5472008	1.4352221
H	10.1350617	−2.5640776	−2.0793266
H	10.4143121	−2.8420296	−4.5304894
H	9.2292126	−1.3421366	−6.0854654
H	7.8045011	0.4777633	−5.2325663
H	9.2064378	2.1942508	−4.006145
H	10.2633277	0.0470643	−0.3550206
H	4.8481582	−3.45661	−1.2670581

H	6.729541	−3.2222122	2.5645931
H	8.3638133	−1.6320991	1.6704728
H	6.4870283	−1.8823162	−2.1712627
H	6.3509467	3.4441737	−4.9078623
H	6.3627131	3.52294	−0.6256206
H	4.2473225	4.7507616	−0.6433868
H	4.2447828	4.6941717	−4.9180377
H	4.955363	−4.1472011	1.1090099
H	3.1664465	5.3496992	−2.7896832

Table S 23: Coordinates of **[9,9]-THII-2** (PBEh-3c/def2-mSVP).

	x	y	z
C	−3.1153968	1.0730576	−2.360071
C	−3.6794741	0.3618777	−3.4073173
C	−3.1190969	−0.8441208	−3.8167817
C	−2.00858	−1.3667107	−3.1697051
C	−1.4652756	−0.6720696	−2.0922851
C	−2.0045411	0.5471092	−1.7195189
C	−0.2681351	−1.027083	−1.2534589
C	−0.3650009	−2.4167923	−0.5189702
C	−1.2078812	−2.891756	1.8727963
C	−1.496208	−2.3720833	3.1248017
C	−1.3567751	−1.0088431	3.3661702
C	−0.9555145	−0.146572	2.3559452
C	−0.7036776	−0.6625688	1.0875409
C	−0.8048647	−2.0256628	0.8685957
C	−0.2478492	0.0639101	−0.148378
C	−1.2077931	1.2160896	−0.6288849
O	−2.0298112	1.7448428	0.3738216
C	−0.3760982	2.3665141	−1.1728925
O	−1.2465269	−3.3285707	−1.11254
C	1.0066957	−3.0719688	−0.5040145
C	1.978039	−2.6662241	0.4063037
C	3.2526006	−3.2113873	0.3748731
C	3.57606	−4.1773278	−0.564876
C	2.612867	−4.5904827	−1.4721524
C	1.3389056	−4.042646	−1.4447015
C	0.1761627	2.3027879	−2.4487534
C	0.9946173	3.3181772	−2.9197492
C	1.2700687	4.4190012	−2.1240467
C	0.7200025	4.4925036	−0.8537603
C	−0.0950918	3.4752791	−0.3796738
H	−3.5200153	2.0305315	−2.0571181
H	−3.5518259	−1.3795155	−4.6518822
H	−1.5717913	−2.2963014	−3.509259
H	0.6357512	−0.999062	−1.8660442
H	−1.2765815	−3.9555776	1.6835399
H	−1.5601441	−0.6163501	4.3540425
H	−0.8337744	0.9085209	2.5613053
H	0.7564658	0.4646365	0.0059089
H	−2.5652113	1.0317218	0.7362671
H	−2.1239081	−2.9332584	−1.1354241
H	1.7417599	−1.9260443	1.1604515
H	3.9915057	−2.8834623	1.0943019
H	2.8513881	−5.3478484	−2.2077022
H	0.6021967	−4.3798648	−2.159854
H	−0.0403744	1.4606705	−3.0940208
H	1.4109504	3.2495217	−3.9162713
H	0.9231957	5.3494126	−0.2245308
H	−0.5151664	3.5527594	0.6130251
H	−4.5470612	0.7533561	−3.9220873
H	−1.8124727	−3.0295574	3.9239517
H	4.5687633	−4.6072806	−0.5874863
H	1.9045228	5.2145216	−2.4920705

Table S 24: Coordinates of **[11,9]-THII-2** (PBEh-3c/def2-mSVP).

	x	y	z
C	0.4917659	2.9448067	−0.0425493
C	−0.1491219	2.7328098	−1.2529539
C	0.0429936	1.5427555	−1.9479619
C	0.8530925	0.5418376	−1.4312516
C	1.4659159	0.7385314	−0.1966436
C	1.3018108	1.942222	0.4670163
C	2.389682	−0.1838866	0.5509185
C	1.7785917	−1.593937	0.8950257
C	0.5696249	−2.3139974	3.0575752
C	0.323549	−2.0410128	4.3938541
C	0.8587783	−0.9011368	4.9857517
C	1.6209892	−0.0084773	4.245988
C	1.8356758	−0.2620321	2.8939233
C	1.3339414	−1.4197989	2.3246596
C	2.6314107	0.5421189	1.9023651
C	2.1438683	2.0277834	1.7142224
O	1.4258478	2.5465622	2.7987279
C	3.3511108	2.9288523	1.5100826
O	0.7257481	−1.991093	0.0615891
C	2.86295	−2.6525818	0.7745915
C	2.9735631	−3.4262966	−0.3772065
C	4.0044834	−4.3442162	−0.513166
C	4.9426287	−4.5030197	0.4949393
C	4.8388541	−3.7363227	1.6448161
C	3.8060681	−2.8216157	1.783934
C	3.8797713	3.6569623	2.5721199
C	5.0215958	4.4254129	2.4004625
C	5.6558546	4.4776711	1.1689498
C	5.1351229	3.7556751	0.1067338

**C**	3.991076	2.990641	0.2758367
**H**	0.3797169	3.8814155	0.4888904
**H**	−0.4420683	1.396884	−2.904374
**H**	1.0082972	−0.3713974	−1.9899182
**H**	3.3178202	−0.3141334	−0.009905
**H**	0.1899377	−3.2166951	2.5958587
**H**	0.6818008	−0.7097806	6.0361602
**H**	2.0462595	0.8646753	4.7224124
**H**	3.6873228	0.53597	2.1815901
**H**	0.655909	1.9887295	2.9490571
**H**	0.0259065	−1.3327838	0.120317
**H**	2.2517364	−3.319102	−1.1743122
**H**	4.0713551	−4.9407086	−1.4138623
**H**	5.5594544	−3.8536055	2.4436176
**H**	3.7337867	−2.2460383	2.6981867
**H**	3.3996219	3.6311984	3.5399743
**H**	5.4152417	4.9889136	3.236546
**H**	5.6147958	3.7918282	−0.8626578
**H**	3.5913245	2.4488707	−0.5721358
**H**	−0.7862347	3.501662	−1.6699681
**H**	−0.2705302	−2.7250944	4.9855027
**H**	5.7450676	−5.2208394	0.387058
**H**	6.5457755	5.0789438	1.0372818

Table S 25: Coordinates of **[11,11]-THII-2** (PBEh-3c/def2-mSVP).

	x	y	z
**C**	−2.2635379	2.9383403	0.3929852
**C**	−2.7239178	3.529397	−0.7729971
**C**	−2.3478622	3.0199	−2.0119474
**C**	−1.532479	1.9007966	−2.1014439
**C**	−1.1023147	1.2827441	−0.9303638
**C**	−1.4474935	1.8222495	0.2968874
**C**	−0.2108754	0.080902	−0.7748514
**C**	−0.7429979	−1.2302217	−1.4654615
**C**	−2.16992	−3.106918	−0.4174615
**C**	−2.6144856	−3.7118273	0.7475645
**C**	−2.2567631	−3.1911388	1.9872816
**C**	−1.4762838	−2.0475417	2.078493
**C**	−1.0624548	−1.4167463	0.9083209
**C**	−1.3885656	−1.9664047	−0.319661
**C**	−0.208184	−0.1879339	0.7546156
**C**	−0.7822367	1.1060448	1.4442095
**O**	−1.6720642	0.8589609	2.496843
**C**	0.3671488	1.9195661	2.0171443
**O**	−1.6373279	−1.0108833	−2.5204695
**C**	0.4326198	−2.0074181	−2.035687
**C**	0.7680314	−1.8962517	−3.3821748
**C**	1.881246	−2.551122	−3.8875002
**C**	2.6789666	−3.324604	−3.0584939
**C**	2.3511979	−3.4403213	−1.7169897
**C**	1.2359454	−2.7894575	−1.2113465
**C**	1.152762	2.7190878	1.1922986
**C**	2.2457168	3.4049816	1.7000404
**C**	2.5684298	3.3075748	3.044311
**C**	1.7881197	2.5170991	3.8737086
**C**	0.6973894	1.8268485	3.3661817
**H**	−2.5184404	3.3485736	1.3619893
**H**	−2.6920841	3.5036997	−2.9167904
**H**	−1.2322515	1.5260126	−3.0708034
**H**	0.7899781	0.3107238	−1.146989
**H**	−2.410137	−3.5246791	−1.3870029
**H**	−2.5876405	−3.6854643	2.8914109
**H**	−1.1902874	−1.6633965	3.048481
**H**	0.798562	−0.3867031	1.1287706
**H**	−2.4103222	0.3433211	2.156684
**H**	−2.3919333	−0.5180776	−2.1822981
**H**	0.1581881	−1.2998246	−4.0457584
**H**	2.1237407	−2.4567238	−4.9381249
**H**	2.9600227	−4.0460752	−1.058582
**H**	0.9884764	−2.9080682	−0.1639263
**H**	0.9082935	2.8237829	0.1426796
**H**	2.8409961	4.0235686	1.041177
**H**	2.0263211	2.4371526	4.926511
**H**	0.1012722	1.2170753	4.0300743
**H**	−3.3624733	4.4015171	−0.7221069
**H**	−3.22597	−4.6030604	0.6953311
**H**	3.5461097	−3.8357581	−3.4554922
**H**	3.4181095	3.8460309	3.4429797

11 List of References

[1] D. B. G. Williams, M. Lawton, *J. Org. Chem.* **2010**, *75*, 8351–8354.

[2] G. R. Fulmer, A. J. M. Miller, N. H. Sherden, H. E. Gottlieb, A. Nudelman, B. M. Stoltz, J. E. Bercaw, K. I. Goldberg, *Organometallics* **2010**, *29*, 2176–2179.

[3] C. Bannwarth, S. Ehlert, S. Grimme, *J. Chem. Theory Comput.* **2019**, *15*, 1652–1671.

[4] C. Bannwarth, E. Caldeweyher, S. Ehlert, A. Hansen, P. Pracht, J. Seibert, S. Spicher, S. Grimme, *Wiley Interdiscip. Rev. Comput. Mol. Sci.* **2021**, *11*, 1–49.

[5] F. Neese, *Wiley Interdiscip. Rev. Comput. Mol. Sci.* **2012**, *2*, 73–78.

[6] F. Neese, *Wiley Interdiscip. Rev. Comput. Mol. Sci.* **2025**, *15*, e70019.

[7] F. Neese, F. Wennmohs, U. Becker, C. Riplinger, *J. Chem. Phys.* **2020**, *152*, 224108.

[8] F. Neese, *Chem. Phys. Lett.* **2000**, *325*, 93–98.

[9] R. Ahlrichs, Turbomole, *7.7.1* **2022**, Turbomole GmbH, since 2007.

[10] S. Grimme, J. G. Brandenburg, C. Bannwarth, A. Hansen, *J. Chem. Phys.* **2015**, *143*, 054107.

[11] S. Grimme, J. Antony, S. Ehrlich, H. Krieg, *J. Chem. Phys.* **2010**, *132*, 154104.

[12] S. Grimme, S. Ehrlich, L. Goerigk, *J. Comput. Chem.* **2011**, *32*, 1456–1465.

[13] A. D. Becke, E. R. Johnson, *J. Chem. Phys.* **2005**, *123*, 154101.

[14] E. R. Johnson, A. D. Becke, *J. Chem. Phys.* **2006**, *124*, 174104.

[15] K. Eichkorn, O. Treutler, H. Öhm, M. Häser, R. Ahlrichs, *Chem. Phys. Lett.* **1995**, *242*, 652–660.

[16] K. Eichkorn, F. Weigend, O. Treutler, R. Ahlrichs, *Theor. Chem. Acc.* **1997**, *97*, 119–124.

[17] F. Weigend, *Phys. Chem. Chem. Phys.* **2006**, *8*, 1057.

[18] C. F. MacRae, I. Sovago, S. J. Cottrell, P. T. A. Galek, P. McCabe, E. Pidcock, M. Platings, G. P. Shields, J. S. Stevens, M. Towler, P. A. Wood, *J. Appl. Crystallogr.* **2020**, *53*, 226–235.

[19] Y. Zhao, D. G. Truhlar, *J. Phys. Chem. A* **2005**, *109*, 5656–5667.

[20] F. Weigend, F. Furche, R. Ahlrichs, *J. Chem. Phys.* **2003**, *119*, 12753–12762.

[21] F. Weigend, R. Ahlrichs, *Phys. Chem. Chem. Phys.* **2005**, *7*, 3297.

[22] E. Caldeweyher, C. Bannwarth, S. Grimme, *J. Chem. Phys.* **2017**, *147*, 034112.

[23] E. Caldeweyher, S. Ehlert, A. Hansen, H. Neugebauer, S. Spicher, C. Bannwarth, S. Grimme, *J. Chem. Phys.* **2019**, *150*, 154122.

[24] E. Caldeweyher, J. M. Mewes, S. Ehlert, S. Grimme, *Phys. Chem. Chem. Phys.* **2020**, *22*, 8499–8512.

[25] E. F. Pettersen, T. D. Goddard, C. C. Huang, G. S. Couch, D. M. Greenblatt, E. C. Meng, T. E. Ferrin, UCSF Chimera - A Visualization System for Exploratory Research and Analysis, *J. Comput. Chem.* **2004**, *25*, 1605–1612.

[26] T. D. Goddard, C. C. Huang, E. C. Meng, E. F. Pettersen, G. S. Couch, J. H. Morris, T. E. Ferrin, *Protein Sci.* **2017**, *27*, 14–25.

[27] E. F. Pettersen, T. D. Goddard, C. C. Huang, E. C. Meng, G. S. Couch, T. I. Croll, J. H. Morris, T. E. Ferrin, *Protein Sci.* **2021**, *30*, 70–82.

[28] A. J. Schaefer, V. M. Ingman, S. E. Wheeler, *J. Comput. Chem.* **2021**, *42*, 1750–1754.

[29] V. M. Ingman, A. J. Schaefer, L. R. Andreola, S. E. Wheeler, *Wiley Interdiscip. Rev. Comput. Mol. Sci.* **2021**, *11*, e1510.

[30] C. Adamo, V. Barone, *J. Chem. Phys.* **1999**, *110*, 6158–6170.

[31] C. Ochsenfeld, J. Gauss, R. Ahlrichs, *J. Chem. Phys.* **1995**, *103*, 7401–7407.

[32] O. Treutler, R. Ahlrichs, *J. Chem. Phys.* **1995**, *102*, 346–354.

[33] F. Furche, D. Rappoport, in *Theor. Comput. Chem.*, Elsevier, Amsterdam, **2005**, pp. 93–128.

[34] T. Bruhn, A. Schaumlöffel, Y. Hemberger, G. Bringmann, *Chirality* **2013**, *25*, 243–249.

[35] T. Bruhn, A. Schaumlöffel, Y. Hemberger, G. Pescitelli, **2017**, SpecDis version 1.71, Berlin, Germany.

[36] OriginPro, **2023**, OriginLab Corporation, Northampton, MA, USA.

[37] C. E. Colwell, T. W. Price, T. Stauch, R. Jasti, *Chem. Sci.* **2020**, *11*, 3923–3930.

[38] M. J. Frisch, G. W. Trucks, H. B. Schlegel, G. E. Scuseria, M. A. Robb, J. R. Cheeseman, G. Scalmani, V. Barone, G. A. Petersson, H. Nakatsuji, X. Li, M. Caricato, A. V. Marenich, J. Bloino, B. G. Janesko, R. Gomperts, B. Mennucci, H. P. Hratchian, J. V. Ortiz, A. F. Izmaylov, J. L. Sonnenberg, D. Williams-Young, F. Ding, F. Lipparini, F. Egidi, J. Goings, B. Peng, A. Petrone, T. Henderson, D. Ranasinghe, V. G. Zakrzewski, J. Gao, N. Rega, G. Zheng, W. Liang, M. Hada, M. Ehara, K. Toyota, R. Fukuda, J. Hasegawa, M. Ishida, T. Nakajima, Y. Honda, O. Kitao, H. Nakai, T. Vreven, K. Throssell, J. A. J. Montgomery, J. E. Peralta, F. Ogliaro, M. J. Bearpark, J. J. Heyd, E. N. Brothers, K. N. Kudin, V. N. Staroverov, T. A. Keith, R. Kobayashi, J. Normand, K. Raghavachari, A. P. Rendell, J. C. Burant, S. S. Iyengar, J. Tomasi, M. Cossi, J. M. Millam, M. Klene, C. Adamo, R. Cammi, J. W. Ochterski, R. L. Martin, K. Morokuma, O. Farkas, J. B. Foresman, D. J. Fox, **2016**, Gaussian, Inc. Wallingford CT.

[39] A. D. Becke, *J. Chem. Phys.* **1993**, *98*, 5648–5652.

[40] P. J. Stephens, F. J. Devlin, C. F. Chabalowski, M. J. Frisch, *J. Phys. Chem.* **1994**, *98*, 11623–11627.

[41] R. Ditchfield, W. J. Hehre, J. A. Pople, *J. Chem. Phys.* **1971**, *54*, 724–728.

[42] W. J. Hehre, R. Ditchfield, J. A. Pople, *J. Chem. Phys.* **1972**, *56*, 2257−2261.

[43] P. C. Hariharan, J. A. Pople, *Theor. Chim. Acta* **1973**, *28*, 213–222.

[44] P. C. Hariharan, J. A. Pople, *Mol. Phys.* **1974**, *27*, 209–214.

[45] M. M. Francl, W. J. Pietro, W. J. Hehre, J. S. Binkley, M. S. Gordon, D. J. Defrees, J. A. Pople, J. S. Binkley, M. S. Gordon, *J. Chem. Phys.* **1982**, *77*, 3654–3665.

[46] W. Humphrey, A. Dalke, K. Schulten, *J. Mol. Graph.* **1996**, *14*, 33–38, http://www.ks.uiuc.edu/Research/vmd/.

[47] P. Seitz, M. Bhosale, L. Rzesny, A. Uhlmann, J. S. Wössner, R. Wessling, B. Esser, *Angew. Chemie Int. Ed.* **2023**, *62*, e202306184.

[48] R. O. Diffraction, **2023**.

[49] G. M. Sheldrick, *Acta Crystallogr. Sect. A Found. Adv.* **2015**, *71*, 3–8.

[50] G. M. Sheldrick, *Acta Crystallogr. Sect. C Struct. Chem.* **2015**, *71*, 3–8.

[51] C. B. Hübschle, G. M. Sheldrick, B. Dittrich, *J. Appl. Crystallogr.* **2011**, *44*, 1281–1284.

[52] C. R. Groom, I. J. Bruno, M. P. Lightfoot, S. C. Ward, *Acta Crystallogr. Sect. B Struct. Sci. Cryst. Eng. Mater.* **2016**, *72*, 171–179.

[53] D. Kratzert, "https://dkratzert.de/finalcif.html," **n.d.**

[54] D. Kratzert, I. Krossing, *J. Appl. Crystallogr.* **2018**, *51*, 928–934.

[55] D. Kratzert, J. J. Holstein, I. Krossing, *J. Appl. Crystallogr.* **2015**, *48*, 933–938.

[56] A. L. Spek, *Acta Crystallogr. Sect. C Struct. Chem.* **2015**, *71*, 9–18.

[57] L. D. Barron, *Mol. Light Scatt. Opt. Act.* **2004**, DOI 10.1017/cbo9780511535468.

[58] P. l. Polavarapu, *Chiroptical Spectroscopy, Fundamentals and Applications*, **2017**.

[59] D. Wassy, M. Hermann, J. S. Wössner, L. Frédéric, G. Pieters, B. Esser, *Chem. Sci.* **2021**, *12*, 10150–10158.

[60] J. S. Wössner, J. Kohn, D. Wassy, M. Hermann, S. Grimme, B. Esser, *Org. Lett.* **2022**, *24*, 983–988.
